# Home-Based Prehabilitation for Older Surgical Patients With Frailty: A Randomized Clinical Trial

**DOI:** 10.1001/jamasurg.2025.5288

**Published:** 2025-12-03

**Authors:** Daniel I. McIsaac, Susan Lee, Dean Fergusson, Chelsia Gillis, Rachel G. Khadaroo, Amanda Meliambro, John Muscedere, Antoine Eskander, Husein Moloo, Gregg Nelson, Tarit Saha, Rosaleen Chun, Pablo E. Serrano, Duminda N. Wijeysundera, Monica Taljaard, and the PREPARE Trial Investigator Group, Keely Barnes, Sylvain Boet, Laura Boland, Karina Branje, Rodney Breau, Gregory L. Bryson, Irfan Dhalla, Elijah Dixon, Gary Dobson, Mary Farnand, Alan Forster, Sylvain Gagne, Emily Hladkowicz, Jayna Holroyd-Leduc, Allen Huang, Joanne Hutton, Eric Jacobsohn, John Joanisse, Ana Johnson, Stephanie Johnson, Noha Khalil, Gurlie Kidd, Manoj Lalu, Luke T. Lavallée, Tien Le, Max Levine, Cameron Love, Colin McCartney, Michael McMullen, Lucas Mellaci Bergamascki, Ronald Moore, Michelle Mozel, Sudhir Nagpal, Julie Nantel, Barbara Power, Celena Scheede-Bergdahl, Laura Tamblyn-Watts, Kednapa Thavorn, Daniel Trottier, Carl van Walraven, Ilun Yang

**Affiliations:** 1 Department of Anesthesiology and Pain Medicine, University of Ottawa, Ottawa, Ontario, Canada; 2 Acute Care Research, Ottawa Hospital Research Institute, Ottawa, Ontario, Canada; 3 School of Epidemiology and Public Health, University of Ottawa, Ottawa, Ontario, Canada; 4 Aims Research, Department of Anesthesiology and Perioperative Medicine, Royal Columbian Hospital, New Westminster, British Columbia, Canada; 5 Department of Anesthesiology, Pharmacology and Therapeutics, University of British Columbia, Vancouver, British Columbia, Canada; 6 Methods and Implementation Research, Ottawa Hospital Research Institute, Ottawa, Ontario, Canada; 7 School of Human Nutrition, McGill University, Montreal, Quebec, Canada; 8 Department of Surgery, University of Alberta, Edmonton, Alberta, Canada; 9 Department of Medicine, Memorial University, St John’s, Newfoundland and Labrador, Canada; 10 Department of Critical Care Medicine, Queen’s University, Kingston, Ontario, Canada; 11 Department of Otolaryngology—Head and Neck Surgery, Sunnybrook Health Sciences Centre, Toronto, Ontario, Canada; 12 Department of Otolaryngology—Head and Neck Surgery, University of Toronto, Toronto, Ontario, Canada; 13 Department of Surgery, University of Ottawa, Ottawa, Ontario, Canada; 14 Department of Obstetrics and Gynaecology, University of Calgary, Calgary, Alberta, Canada; 15 Department of Anesthesiology and Perioperative Medicine, Queen’s University, Kingston, Ontario, Canada; 16 Department of Anesthesiology, Perioperative and Pain Medicine, University of Calgary, Calgary, Alberta, Canada; 17 Department of Surgery, McMaster University, Hamilton, Ontario, Canada; 18 Li Ka Shing Knowledge Institute, Unity Health Toronto, Toronto, Ontario, Canada; 19 St Michael’s Hospital, Unity Health Toronto, Toronto, Ontario, Canada; 20 Division of Urology, University of Ottawa, Ottawa, Ontario, Canada; 21 Clinical Programs, Quality, Equity and Medical Affairs, Unity Health Toronto, Toronto, Ontario, Canada; 22 Department of Medicine, University of Toronto, Toronto, Ontario, Canada; 23 Department of Surgery, Oncology, and Community Health Sciences, University of Calgary, Calgary, Alberta, Canada; 24 Cancer Assessment Clinic, The Ottawa Hospital, Ottawa, Ontario, Canada; 25 Department of Medicine, McGill University, Montreal, Quebec, Canada; 26 Department of Medicine, University of Calgary, Calgary, Alberta, Canada; 27 Division of Geriatric Medicine, University of Ottawa, Ottawa, Ontario, Canada; 28 Department of Anesthesiology, Perioperative and Pain Medicine, University of Manitoba, Winnipeg, Manitoba, Canada; 29 Institut du Savoir Montfort, Hôpital Montfort, Ottawa, Ontario, Canada; 30 Department of Public Health Sciences, Queen’s University, Kingston, Ontario, Canada; 31 Department of Otolaryngology—Head and Neck Surgery, University of Ottawa, Ottawa, Ontario, Canada; 32 Division of Gynecologic Oncology, The Ottawa Hospital, Ottawa, Ontario, Canada; 33 Office of the President, The Ottawa Hospital, Ottawa, Ontario, Canada; 34 Department of Anesthesiology and Pain Medicine, University of Toronto, Toronto, Ontario, Canada; 35 Division of Vascular Surgery, University of Ottawa, Ottawa, Ontario, Canada; 36 School of Human Kinetics, Faculty of Health Sciences, University of Ottawa, Ottawa, Ontario, Canada; 37 Department of Kinesiology and Physical Education, McGill University, Montreal, Quebec, Canada; 38 Factor-Inwentash Faculty of Social Work, University of Toronto, Toronto, Ontario, Canada; 39 Department of Surgery, Hôpital Montfort, Ottawa, Ontario, Canada; 40 Department of Medicine, University of Ottawa, Ottawa, Ontario, Canada; 41 Division of General Surgery, McMaster University, Hamilton, Ontario, Canada

## Abstract

**Importance:**

Explanatory trials suggest that prehabilitation has efficacy in improving surgical outcomes. The effectiveness of offering home-based prehabilitation across multiple centers and for older adults with frailty remains unknown.

**Objective:**

To evaluate the effectiveness of offering coach-supported, home-based prehabilitation to older surgical patients with frailty.

**Design, Setting, and Participants:**

This study is a pragmatic, parallel-arm, multicenter randomized clinical trial with embedded qualitative assessment. Clinicians and assessors were fully blinded; participants were partially blinded in that the control arm received publicly available activity and nutritional guidelines. From March 2, 2020, to February 8, 2024, participants aged 60 years and older with frailty (Clinical Frailty Scale score ≥4) scheduled for elective, inpatient noncardiac surgery were recruited from surgeon’s offices at 13 centers in Canada. Data analysis was completed from October 3, 2024, to December 5, 2024.

**Intervention:**

Assignment to a home-based, multimodal program of exercise and personalized nutritional recommendations, remotely supported by coaches using a theory-based approach to enhance adherence.

**Main Outcomes and Measures:**

The coprimary outcomes were patient-reported disability 30 days after surgery using the World Health Organization Disability Assessment Schedule 2.0 and the incidence of any postoperative complication during the surgical hospitalization. Barriers to adherence were identified using the Theoretical Domains Framework. Secondary outcomes were intervention-attributable safety events, health-related quality of life, survival, falls, complication severity, activities of daily living, length of stay, discharge disposition, lower limb function, and readmission. Analysis was by mixed-effects regression, adjusting for stratification and prespecified prognostic factors.

**Results:**

Of 992 eligible participants, 847 (85.4%) were randomized (423 to prehabilitation and 424 to usual care), and 705 participants had their planned surgery (353 in the prehabilitation group and 352 in the usual care group). A total of 452 participants (53.4%) were female, and mean (SD) participant age was 71.7 (7.1) years. A median (IQR) of 4 weeks (3-7) of prehabilitation enrollment was achieved. Preoperative safety outcomes did not differ between groups. Participants assigned to prehabilitation reported a mean (SD) postoperative disability score of 23.5 (21.8) compared to 24.7 (23.8) for usual care (adjusted mean difference, −1.4; 97.5% CI, −4.9 to 2.0; *P* = .36). Complications occurred in 177 participants (50.1%) assigned to prehabilitation and 168 control participants (47.7%) (adjusted odds ratio, 1.05; 97.5% CI, 0.73-1.49; *P* = .78). Participants completing more than 75% of prescribed exercises reported significantly lower disability scores with prehabilitation (mean difference, −4.9; 97.5% CI, −9.8 to −0.01; *P* = .02), but there was no significant difference in complications (odds ratio, 1.06; 97.5% CI, 0.67-1.67; *P* = .79). Primary barriers to adherence were competing priorities and motivation.

**Conclusions and Relevance:**

In this randomized clinical trial among older adults with frailty scheduled for surgery, assignment to home-based prehabilitation before surgery did not improve postoperative disability scores or reduce complications.

**Trial Registration:**

ClinicalTrials.gov Identifier: NCT04221295

## Key Points

**Question:**

Can home-based prehabilitation improve patient-centered outcomes after surgery for older adults living with frailty?

**Findings:**

In this pragmatic randomized clinical trial that included 847 older adults living with frailty, assignment to home-based prehabilitation before surgery did not improve postoperative disability scores or reduce complications. However, participants completing more than 75% of prescribed exercises reported significantly lower disability scores with prehabilitation.

**Meaning:**

Assignment to home-based prehabilitation with structured coaching did not improve patient-centered outcomes across all participants; given promising findings in adherent participants, intervention design and delivery require further optimization to overcome barriers to adherence.

## Teaser

This multicenter randomized clinical trial evaluates the effectiveness of offering coach-supported, home-based prehabilitation to older patients with frailty scheduled for surgery.

## Introduction

Among the millions of people who have major surgery each year,^1^ more than 20% experience a complication,^2^ with a similar proportion reporting worsened postoperative disability.^3,4^ A growing number of surgical patients are older adults with frailty, a state of multidimensional loss of reserve and vulnerability to adverse health outcomes.^5,6^ Older adults with frailty are at least twice as likely to experience a postoperative complication or report worsened disability after surgery compared to those without frailty.^3,6^

Prehabilitation, an intervention consisting of physical, nutritional, and/or psychological optimization before surgery,^7^ may prevent complications and improve recovery after surgery. However, the generalizability of efficacy data across multiple centers or real-world settings for a complex intervention like prehabilitation remains a crucial evidence gap. Current evidence is low certainty, as most trials have been explanatory, small, single center, and have a high risk of bias.^8,9^

Translating promising efficacy data into practice is further impeded by the design of most prehabilitation programs, which predominantly require participants to prehabilitate in hospitals or other facilities.^9^ This approach reduces access, limits generalizability, and may not scale at a health system level, especially in countries like the US, Canada, and Australia where long travel distances for hospital care are common.^10,11,12,13^ A related gap is optimizing prehabilitation for older adults with frailty, who may plausibly derive greater benefit from prehabilitation than older adults without frailty but prioritize receipt of health care interventions at home.^14^ Achieving the uptake and adherence required for populations with frailty to benefit from prehabilitation, especially when enrolled in home-based programs, is a challenge.^15,16,17^

We conducted a pragmatic, multicenter randomized clinical trial to evaluate whether assignment to a home-based, multimodal prehabilitation program supported by coaches using a theory-informed approach to optimize adherence improves patient-reported disability and reduces the risk of postoperative complications compared to usual care in older adults with frailty scheduled for elective, noncardiac surgery. We concurrently assessed patient-reported barriers to adherence using the Theoretical Domains Framework (TDF) to inform optimization of current and future prehabilitation programs.^18^

## Methods

### Trial Design

The Preoperative Exercise to Decrease Postoperative Complication Rates and Disability Scores (PREPARE) study was a randomized clinical trial comparing assignment to multimodal prehabilitation to usual care at 13 centers in Canada (eFigure 1 in Supplement 3),^19^ with an embedded study-within-a-trial qualitative assessment of barriers to adherence.^20^ A patient partner contributed to design, conduct, and reporting as a member of the trial executive. The protocol for our trial was published and is available in Supplement 1^21^; the statistical analysis plan was registered and is available in Supplement 2.^22^ Reporting guidelines, including the Consolidated Standards of Reporting Trials (CONSORT) reporting guidelines, follow best practices for trials, intervention description, and patient involvement (eTables 1 and 2 and eMethods 1 in Supplement 3).^23,24,25,26,27^ Ethical approval was granted by Clinical Trials Ontario (project identifier: 1785), the Ottawa Health Science Network—Research Ethics Board (20190409-01T), the Fraser Health Research Ethics Board (2020105), the Conjoint Health Research Ethics Board at the University of Calgary (REB20-0297_REN4), and the University of Alberta Health Research Ethics Board (Pro00104713).

### Participants

All participants provided informed oral consent. Individuals were eligible if aged 60 years or older, living with frailty based on the guideline-recommended Clinical Frailty Scale (CFS)^28,29^ score of 4 or higher (a threshold where frailty-related risk substantially increases in surgical patients,^3,30^ assigned by a trained assessor^31^), and scheduled for elective, inpatient head and neck, thoracic, abdominal, pelvic, or vascular surgery in the subsequent 3 to 12 weeks. Participants were excluded who were unable to speak English or French, cognitively unable to complete assessments, not contactable by telephone, unwilling to participate in exercise, having palliative surgery, or with cardiovascular disease precluding safe participation (severe valvular heart disease, severe cardiac dysrhythmias, or myocardial infarction ≤6 weeks prior to enrollment).

### Randomization and Blinding

Participants were randomly assigned in a 1:1 ratio to prehabilitation or usual care via a web-based allocation system using permuted blocks of randomly varying length, stratified by center and whether surgery was for treatment of cancer or not. The randomization sequence was generated by an independent biostatistician. Clinicians and outcome assessors were blinded. Participants were told that they were participating in a trial to evaluate strategies to improve exercise and nutrition before surgery; control participants received publicly available, paper-based guidelines to provide partial blinding.^32,33^

### Intervention

Intervention participants were enrolled in a home-based multimodal prehabilitation program from the day of randomization to surgery. The program, described in detail in the eMethods 2 in Supplement 3, consisted of exercise (aerobics, strength, and flexibility) with personalized nutritional recommendations based on programs demonstrating efficacy in smaller randomized trials^15,34^ and consistent with contemporary best evidence.^9^ The exercise component involved three 1-hour sessions per week, consisting of strength training (upper body, lower body, and abdominal movements), 20 minutes of moderate-intensity aerobic exercise (guided by a Borg scale^35^), and flexibility. Exercises were personalized based on individual limitations. Structured nutritional advice and discount coupons for protein supplements were provided. Participants identified at risk of malnutrition using the Canadian Nutrition Screening Tool^36^ were provided personalized advice regarding energy and protein requirements from a nutrition science–trained coach.

Participants were supported centrally by the same coach throughout the program (trained in exercise or nutrition science and structured prehabilitation coaching), with a minimum of a weekly coaching call. Participants could additionally connect with their coach using a toll-free line or email. At calls, coaches advised participants on aerobic, strength, and flexibility exercise progressions and monitored adherence. Where adherence deficits were identified, coaches identified the main barrier(s) (categorized as medical, behavioral, or other) and then mapped the barrier(s) to relevant TDF domains, which were linked to evidence-informed strategies to support participants in improving adherence (eMethods 3 in Supplement 3).^17,18,37^

### Control

Control participants were provided paper copies of publicly available guidelines for physical activity and healthy eating.^32,33^ No contact or support was provided during the preoperative period. All other aspects of perioperative care for intervention and control participants were at the discretion of treating physicians, who were blinded to allocation.

### Measures

The trial had 2 coprimary outcomes. The first outcome was patient-reported disability 30 days after surgery, measured using the 12-item World Health Organization Disability Assessment Schedule 2.0 (WHODAS disability score; range, 0-48, expressed on a 0- to 100-point scale, with higher scores indicating more severe disability), which has a minimally important difference of 5 points in surgical patients.^4,38,39^ The second outcome was any postoperative complication during the surgical hospitalization, using validated organ system definitions, with severity based on the Clavien-Dindo Classification.^40,41^

Secondary outcomes were health-related quality of life using the EuroQual Visual Analog Scale (EQ-VAS; range, 0-100)^42^ and the EuroQuol 5-dimension, 5-level health utility index (EQ-5D-5L; range, −0.59 to 0.95)^43^; lower limb function using the 5-times sit to stand test^44^; the incidence of any falls; overall survival; activities of daily living (Katz Index^45^); length of stay; discharge disposition; hospital readmissions; and complication severity.^41^

All patient-reported outcomes were measured at baseline and 30 days after surgery. On the day before surgery, safety outcomes (falls, musculoskeletal injuries, head injuries, or unplanned hospitalizations) were collected by phone. During the surgical hospitalization, participants were followed up in person, including medical record review (postoperative days 3, 5, and 7 and at discharge) to identify complications. On the date of discharge, activities of daily living, length of stay, 5-times sit to stand test time, and discharge disposition were recorded. If discharge was on or before day 3, only a single in-hospital visit occurred. Thirty days after surgery, falls, readmissions, and deaths were ascertained. For participants who did not have their planned surgery, 30-day outcomes were collected 114 days postrandomization (maximum 12-week program enrollment plus 30 days). Any outcomes not assessed during the hospitalization were collected by telephone.

### Sample Size

A Bonferroni correction (2-sided α = .025) was used to maintain the overall type I error rate across coprimary outcomes at 0.05. With 850 participants, the trial was designed to have 90% power to detect a minimum clinically important absolute mean difference of 5 points on the 100-point WHODAS disability score^39^ using an analysis of covariance (ANCOVA), assuming a common SD of 20 and correlation between baseline and follow-up of 0.4, with 5% attrition. For the coprimary outcome of any complication, we had 90% power to detect an absolute difference of 0.15 in complications using a pooled *z* test, assuming a control arm complication rate of 0.55 and accounting for 15% nonadherence to the exercise intervention and 15% attrition (including participants not having surgery). Rationale for increasing the sample size from the initial estimate of 750^21^ based on the impacts of the COVID-19 pandemic is reported in the eMethods 4 in Supplement 3. This decision was made without any knowledge of outcome data.

### Adherence

Adherence was monitored via weekly coaching calls, supplemented by participant logbooks. Adherence was expressed as a proportion, where the numerator was the number of prescribed exercises completed and the denominator was the total number of prescribed exercises. Two measures of adherence were calculated. Overall adherence reflected a participant’s behavior while enrolled, as it was calculated as the number of completed exercises divided by the number of prescribed exercises during each participant’s enrollment period, regardless of duration. Per-protocol adherence reflected a participant’s achievement of the 3 weeks of prehabilitation that our protocol defined as the minimum required dose. Therefore, per-protocol adherence was calculated as the number of exercises completed in the 3 weeks prior to surgery with a fixed denominator (the number of prescribed exercises in 3 weeks), which acknowledged that participants enrolled for less than 3 weeks were unlikely to achieve the required dose.^9^

On the day prior to surgery, participants in both arms were asked to report self-rated change in physical activity compared to enrollment (5-point scale, with 1 indicating no change, 3 indicating moderate change, and 5 indicating the largest change imaginable). For prehabilitation participants, barriers to prehabilitation adherence were collected using a validated TDF survey tool^46^ (eMethods 5 in Supplement 3).

### Statistical Analysis

The prespecified primary analysis population differed between the coprimary outcomes.^19^ For the WHODAS disability score, the primary analysis population was all randomized participants, which was in keeping with our pragmatic design,^19^ as prehabilitation could improve disability scores for participants who did not ultimately have surgery. For postoperative complications, the primary analysis population was all randomized participants who had their planned surgery, as a postoperative complication can only occur among those undergoing surgery. Likewise, for secondary nonhospitalization outcomes, the primary analysis population was all randomized participants, and for secondary in-hospital outcomes, it was all randomized participants who had their planned surgery. The per-protocol population included intervention participants who completed more than 75% of prescribed exercises in the final 3 weeks and had their planned surgery.

Statistical analyses were conducted by an independent biostatistician according to the prespecified statistical analysis plan.^22^ We used multiple imputation under a missing-at-random assumption to account for any missing data, with 25 imputations generated using the method of fully conditional specification. The imputation model included all primary and secondary outcomes, the allocation and stratification variables, and all available baseline characteristics.

The WHODAS disability score was analyzed using ANCOVA via mixed-effects linear regression, with the baseline WHODAS disability score entered as a covariate along with prespecified adjustment for stratification and baseline prognostic factors (cancer surgery [vs not], age, sex, surgery type, malnutrition risk, and frailty score), with a random intercept for each study site.^47,48^ The intervention effect was expressed as an adjusted mean difference (MD) with 97.5% confidence interval to account for multiplicity due to 2 coprimary outcomes. The incidence of any postoperative complication was analyzed using mixed-effects binary logistic regression, adjusted for stratification and the same prespecified factors, along with a random intercept for each site. The intervention effect was expressed as an adjusted odds ratio (OR) with 97.5% confidence interval. Analyses of secondary outcomes are described in the eMethods 6 in Supplement 3. All random effects assumed a normal distribution.

We conducted prespecified subgroup analyses of coprimary outcomes by age (<75 years vs ≥75 years), sex (male vs female), cancer surgery (vs not), depression (vs no depression), and frailty score (CFS = 4 vs CFS ≥5) using allocation by subgroup interactions. A post hoc analysis by surgery type was also conducted.

Overall and per-protocol adherence were described using medians and interquartile ranges. Weekly reasons for nonadherence were described using counts and proportions. Self-reported changes in activity from enrollment were compared between groups using ordinal regression. Likert scale responses from the TDF barrier survey were described using medians and interquartile ranges (eMethods 7 in Supplement 3).

All analyses were performed using SAS software version 9.4 (SAS Institute). A data safety monitoring board reviewed trial safety data throughout the conduct of the trial.

## Results

### Characteristics of Participants

**Figure 1.**
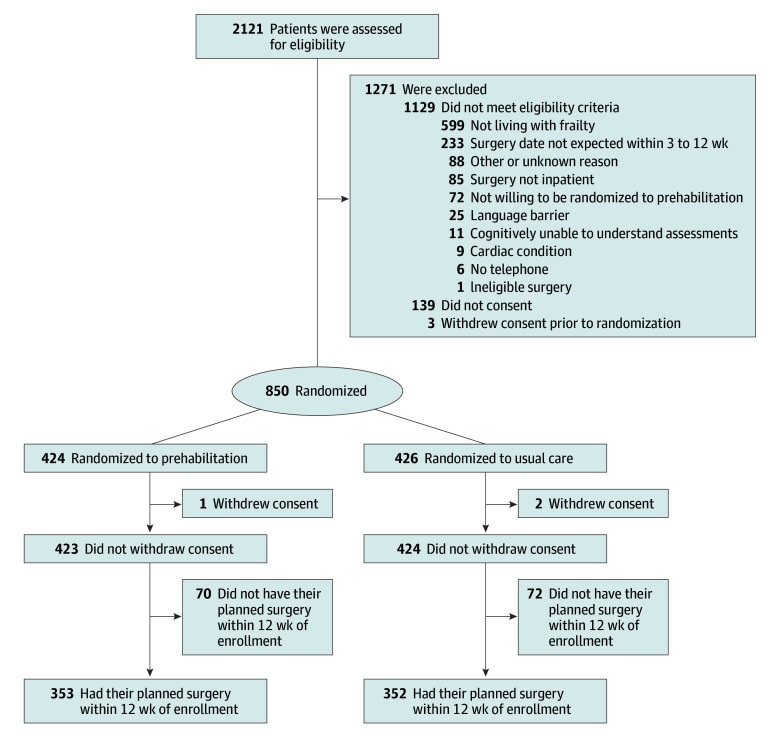
Screening, Enrollment, Randomization, and Follow-Up The World Health Organization Disability Assessment Schedule (WHODAS) score, 5-times sit to stand test, health-related quality of life, health utility, overall survival, and postoperative falls outcomes were analyzed among the 847 participants who underwent randomization and did not withdraw consent. The incidence of any postoperative medical or surgical complication, activities of daily living, discharge disposition, time to hospital discharge, postoperative complication severity, and hospital readmission outcomes were analyzed among the 705 participants who underwent randomization and had their planned surgery within 12 weeks of enrollment.

Participants were enrolled from March 2, 2020, to February 8, 2024. Of 992 eligible participants, 847 (85.4%) were randomized (423 to prehabilitation, 424 to usual care); 705 participants had their planned surgery (353 in the prehabilitation group and 352 in the usual care group) (Figure 1). A total of 452 participants (53.4%) were female, and mean (SD) participant age was 71.7 (7.1) years. Participants in both arms were enrolled in the trial for a median (IQR) of 4 weeks (3-7) before surgery. Most participants had a CFS score of 4 (Table 1). Colorectal, urologic, and gynecologic surgeries were most common. Approximately 75% of participants had cancer surgery, of whom 20% had neoadjuvant therapy. Baseline characteristics for participants who had surgery, by missing data and outcome status, and who completed more than 75% of prescribed exercises per protocol are presented in eTables 3 to 5 in Supplement 3.

**Table 1.** Characteristics of the Patients at Baseline^a^

Characteristic	No. (%)	
	Prehabilitation (n = 423)	Usual care (n = 424)
Age at randomization, mean (SD), y	71.7 (7.0)	71.7 (7.2)
Sex		
Female	235 (55.6)	217 (51.2)
Male	188 (44.4)	207 (48.8)
Days from enrollment to surgery, median (IQR)	38 (24-63)	37 (24-62)
Frailty and functional indicators		
Clinical Frailty Scale score, median (IQR)	4 (4-4)	4 (4-4)
WHODAS disability score, mean (SD)^b^	13.5 (11.9)	14.0 (11.9)
Duke Activity Status Index score, mean (SD)	32.3 (13.6)	32.4 (13.5)
Katz Index, median (IQR)	6 (6-6)	6 (6-6)
Surgery type		
Colorectal	92 (21.8)	89 (21.0)
Head and neck	11 (2.6)	18 (4.3)
Hepatobiliary	57 (13.5)	49 (11.6)
Thoracic	68 (16.1)	71 (16.8)
Vascular	55 (13.0)	53 (12.5)
Urologic or gynecologic	94 (22.2)	103 (24.3)
Other general surgery	46 (10.9)	41 (9.7)
Coexisting conditions		
History of myocardial infarction	35 (8.3)	41 (9.7)
Congestive heart failure	17 (4.0)	18 (4.3)
History of stroke or transient ischemic attack	38 (9.0)	46 (10.9)
Chronic pulmonary disease	65 (15.4)	72 (17.0)
Diabetes with complications	42 (9.9)	54 (12.7)
Liver disease	40 (9.5)	38 (9.0)
Kidney disease	65 (15.4)	68 (16.0)
Current smoker	49 (11.6)	48 (11.3)
At risk of malnutrition	88 (20.8)	78 (18.4)
Oncologic variables		
Cancer	324 (76.6)	317 (75.7)
Receipt of radiation in the last 6 mo	27 (6.4)	28 (6.6)
History of chemotherapy in last 6 mo	54 (12.8)	49 (11.6)

Abbreviation: WHODAS, World Health Organization Disability Assessment Schedule score.

^a^ Participants described in this Table represent the primary analysis population for the WHODAS score.

^b^ Expressed on a 0- to 100-point scale, with higher scores denoting greater disability.

### Safety and Adherence

There were no significant between-group differences in safety during the preoperative period (eTable 6 in Supplement 3). Participants completed a median (IQR) of 78% (44%-96%) of overall prescribed exercises while enrolled. As 23% of participants’ surgeries unexpectedly occurred less than 3 weeks from randomization, median (IQR) per-protocol adherence was 67% (33%-94%) during the 3 weeks before surgery. Prehabilitation participants reported an 8-fold increase in the odds that their self-reported activity levels were higher vs baseline compared to control (generalized OR, 8.24; 95% CI, 6.15-11.03; *P* < .001) (eMethods 7 in Supplement 3). Primary reasons for nonadherence were behavioral (53.3% aerobic, 59.7% strength) and medical (45.1% aerobic, 38.3% strength). Prehabilitation participants reported strong barriers in the TDF domains of Goals (competing priorities), Reinforcement (a lack of acknowledgment from others), and Emotion (lack of inspiration) (eMethods 8 and eFigure 2 in Supplement 3).

### Primary Outcomes

**Figure 2.**
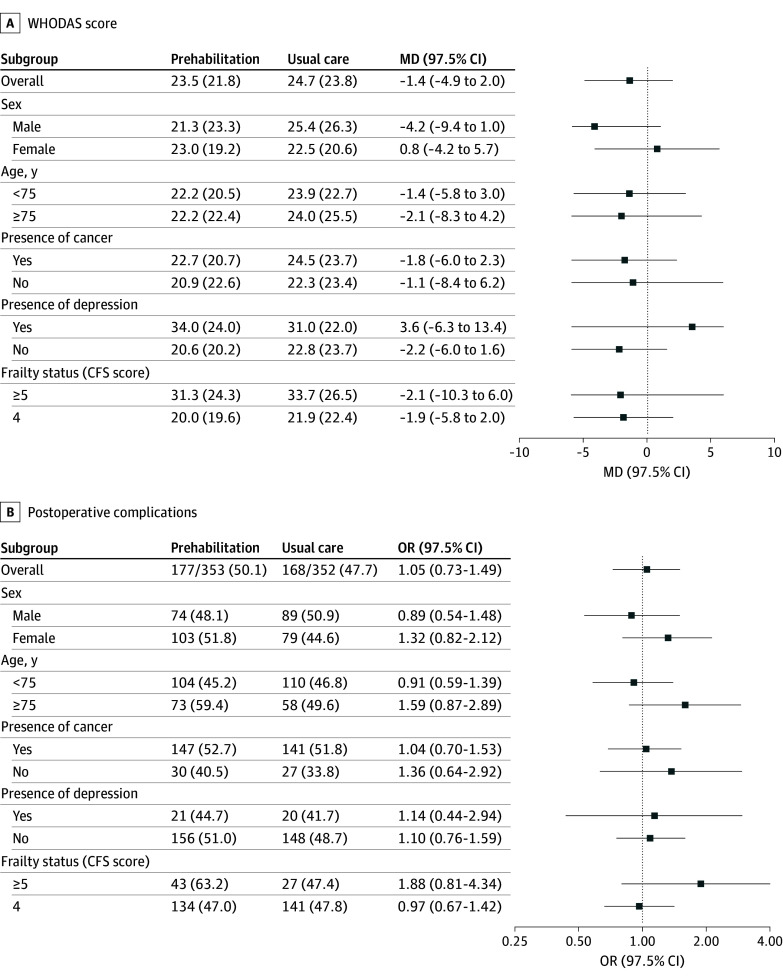
Subgroup Analyses of the Primary Outcomes The trial had 2 primary outcomes. Panel A presents results for the World Health Organization Disability Assessment Schedule (WHODAS) score on the mean difference (MD) scale. Panel B presents results for the incidence of in-hospital, postoperative complications on the odds ratio (OR) scale. The dotted vertical line denotes the null value. Boxes represent the effect estimate, and horizontal lines represent the 97.5% confidence interval. Presence of depression was defined as a positive screen on the Patient Health Questionnaire 2. Frailty status was assigned based on a trained assessor’s rating of frailty using the Clinical Frailty Scale (CFS).

Thirty days after surgery, WHODAS disability scores were complete for 780 participants (92.1%). The mean (SD) disability score was 23.5 (21.8) in the intervention arm compared to 24.7 (23.8) in the control arm (adjusted MD, −1.4; 97.5% CI, −4.9 to 2.0; *P* = .36). Postoperative complication status was ascertained for all 705 participants who had surgery (100%). At least 1 postoperative complication was identified in 177 intervention participants (50.1%) compared to 168 control participants (47.7%) (adjusted OR, 1.05; 97.5% CI, 0.73-1.49; *P* = .78). There was no evidence of effect modification of the intervention by any subgroup for the WHODAS disability score or complications at the 5% level of significance (Figure 2; eTable 7 in Supplement 3). Complication subtypes are reported in eTable 8 in Supplement 3, and WHODAS domains are provided in eFigure 3 in Supplement 3.

### Secondary Outcomes

Intervention participants reported significantly higher health-related quality of life (EQ-VAS: MD, 3.2; 95% CI, 0.19-6.20; *P* = .04), but there were no significant differences in other secondary outcomes (Table 2 and Table 3).

**Table 2.** Primary Outcome and Prespecified Secondary Outcomes at 30-Day Follow-Up^a^

Outcome	Prehabilitation (n = 423)	Usual care (n = 424)	Intervention effect (95% CI)	*P* value
Primary outcome				
WHODAS disability score (0-100), mean (SD)	23.5 (21.8)	24.7 (23.8)	−1.4 (−4.9 to 2.0)^b^	.36
Secondary outcomes				
EuroQol visual analog scale (0-100), mean (SD)	65.7 (21.8)	63.1 (24.4)	3.2 (0.19 to 6.20)^c^	.04
EuroQol utility index (−1 to 1.59 scale), mean (SD)	0.8 (0.2)	0.8 (0.3)	0.03 (−0.007 to 0.06)^c^	.11
Deaths, No./total No. (%)	6/423 (1.4)	7/424 (1.7)	0.87 (0.29 to 2.63)^d^	.81
Falls, No./total No. (%)	31/400 (7.8)	31/410 (7.6)	0.74 (0.42 to 1.32)^e^	.31

Abbreviation: WHODAS, World Health Organization Disability Assessment Schedule score.

^a^ Results described in this Table represent the primary analysis population for the WHODAS score.

^b^ Represents a mean difference with 97.5% confidence interval.

^c^ Represents a mean difference with 95% confidence interval.

^d^ Represents a hazard ratio with 95% confidence interval.

^e^ Represents an odds ratio from mixed-effects binary logistic regression with 95% confidence interval.

**Table 3.** Primary Outcome and Prespecified Secondary Outcomes at Hospital Discharge^a^

Outcome	Prehabilitation (n = 353)	Usual care (n = 352)	Intervention effect (95% CI)	*P* value
Primary outcome				
Any postoperative complication, No./total No. (%)	177/353 (50.1)	168/353 (47.7)	1.05 (0.73 to 1.49)^b^	.78
Secondary outcomes				
5-Times sit to stand time, mean (SD), s	43.4 (19.4)	42.4 (19.8)	0.91 (−1.7 to 3.5)^c^	.49
Katz Index of Activities of Daily Living (0-6), median (IQR)	6 (5 to 6)	6 (5 to 6)	0.91 (0.68 to 1.21)^d^	.52
Discharge disposition (range, died in hospital to home without support), median (IQR)	4 (4 to 4)	4 (4 to 4)	1.11 (0.75 to 1.62)^e^	.61
Time to discharge, mean (SD), d	5.5 (9.4)	5.6 (8.5)	1.06 (0.93 to 1.20)^f^	.39
Postoperative complication severity (range, 0-5), median (IQR)	0 (0 to 2)	0 (0 to 2)	0.96 (0.72 to 1.27)^d^	.76
Readmission, No./total No. (%)	34/350 (9.7)	40/347 (11.7)	0.80 (0.49 to 1.31)^g^	.39

^a^ Results described in this Table represent the primary analysis population for the any complications outcome.

^b^ Represents an odds ratio from mixed-effects binary logistic regression with 97.5% confidence interval. The corresponding absolute risk difference was 1.7% (97.5% CI, −6.5% to 10.0%), which was estimated using marginal standardization based on predicted probabilities from the mixed-effects binary logistic regression, with the confidence interval based on 1000 bootstrap samples.

^c^ Represents a mean difference with 95% confidence interval.

^d^ Represents a generalized odds ratio from mixed-effects ordinal logistic regression with 95% confidence interval, where values <1 denote better outcome with intervention.

^e^ Represents a generalized odds ratio from mixed-effects ordinal logistic regression with 95% confidence interval, where values >1 denote better outcome with intervention.

^f^ Represents a subdistributional hazard ratio with 95% confidence interval, accounting for death as a competing risk, where values >1 denote shorter time to discharge with intervention.

^g^ Represents an odds ratio from mixed-effects binary logistic regression with 95% confidence interval.

### Per-Protocol Results

Among the 152 participants (43.1%) who had their planned surgery and completed more than 75% of prescribed exercises in the 3 weeks before surgery, there was a significant improvement in WHODAS disability score compared to control (MD, −4.9; 97.5% CI, −9.8 to −0.01; *P* = .02) and significant improvements in health utility and health-related quality of life (secondary per-protocol results are presented in eTables 9 and 10 in Supplement 3).

## Discussion

Across 847 randomized older adults with frailty preparing for elective, noncardiac surgery at 13 centers in Canada, assignment to a home-based, coach-supported, multimodal prehabilitation program did not result in significant differences in patient-reported disability scores or postoperative complications. Despite theory-informed, coach-led adherence optimization, low adherence reduces certainty in understanding whether a lack of effectiveness reflects causality or the implementation challenges of delivering home-based prehabilitation on a large scale to older adults with frailty. As adherent participants may experience benefit, further optimization of intervention design and program delivery is required to support effective delivery of prehabilitation services.

A recent systematic review of 186 mostly single-center, explanatory trials with important risks of bias suggests that prehabilitation may meaningfully improve complication rates, length of stay, and patient-centered recovery.^9^ Most trials used facility-based programs,^9^ which can act as a barrier to access, and may not scale in jurisdictions serving geographically distributed populations.^10,11,12,13^ Understanding how prehabilitation programs can effectively generalize across multiple centers or real-world settings remains a key question for patients, clinicians, administrators, and researchers.

Despite the large number of prehabilitation trials published to date, multicenter randomized trials with large sample sizes are rare. A recent 7-center, 251-participant trial of relatively healthy patients with colorectal cancer reported a 12% absolute decrease in the incidence of major complications^49^; however, the trial delivered a hospital-based intervention, enrolled only 37% of eligible participants, closed early, and changed its primary outcome, limiting internal and external validity. In contrast, a 6-center, 668-participant trial using a 2-week home-based program combined with a 4-week postoperative rehabilitation program did not report a significant improvement in patient-reported recovery or complications.^50^ This home-based program was short in duration, had minimal coaching support, and included no nutritional component.

In comparison, our trial provides novel and generalizable data regarding delivery of coach-supported, multimodal prehabilitation that are unavailable from current evidence. First, as patients with frailty live with multidimensional vulnerability, prehabilitation could be especially beneficial. We enrolled 85% of eligible older patients with frailty into a home-based program, a design that was a priority for this vulnerable population.^14^ Home-based prehabilitation also met the realities of delivering care during the COVID-19 pandemic^51^ and supported accessibility for geographically distributed participants.^13^ As prior home-based prehabilitation trials for patients with^15,16^ and without frailty^50^ demonstrate low levels of adherence, we integrated coaching support based on a leading behavior change theory^18^ and evaluated barriers to adherence. However, as our primary results do not support effectiveness among all participants assigned to prehabilitation, despite using a structured approach to supporting adherence, current plans for prehabilitation implementation should cautiously consider our results, which highlight possible gaps in intervention design, as well as barriers to effective delivery. In contrast, signs of effectiveness among adherent participants suggest that individuals meaningfully engaging in prehabilitation may derive benefit.

Delivering effective prehabilitation in the short window before surgery to patients with frailty is challenging. Disease-related symptoms and fatigue are common, while frailty emerges from poorer health behaviors across the lifespan.^17^ These realities were reflected in a high proportion of participants endorsing behavioral and medical barriers to adherence. Structural barriers were also identified; 23% of participants did not have the expected 3-week minimum enrollment before surgery, highlighting the need to integrate prehabilitation and surgical scheduling. That our results, consistent with prior smaller trials in frailty and meta-analyses,^9,15,16^ suggest possible effectiveness among adherent participants without negative safety signals supports the need to address identified gaps and barriers to develop approaches to prehabilitation that can effectively serve the growing population of older surgical patients with frailty.

Our multimodal program included moderate-intensity exercise and nutritional advice, consistent with programs demonstrating efficacy in smaller, home-based trials and contemporary best evidence.^9,34,52^ Median adherence was 78% while enrolled, and prehabilitation participants were 8-fold more likely to report increasing physical activity levels prior to surgery than controls. However, opportunities remain to improve or modify prehabilitation programming for older adults with frailty. For example, explicit protein supplementation, in addition to advice, could enhance effectiveness.^53^ Additionally, for some patients with frailty, more intensive exercise, including supervision at a facility, may be required and could help to overcome participant-reported barriers around goals and reinforcement. A recent trial of a 3-year structured rehabilitation program after surgery and chemotherapy for colon cancer demonstrated benefits in disease-free and overall survival using a combination of facility- and home-based programming.^54^ However, facility-based programs could also exacerbate barriers related to competing priorities. Future research is required to better understand what patient characteristics and preferences best align with home- vs facility-based programs, which could be informed by individual patient meta-analysis of our data and related trials.^15,16,55^ Further refinement of individual components, duration, and dosing for multimodal prehabilitation is also required and could be enhanced through use of wearable technology and subsequent analysis using emerging methods in targeting individualized treatment effects.^56,57^

### Limitations

Our trial has limitations. Control participants were only partially blinded, which could bias estimates to the null. In keeping with pragmatic design, all randomized participants were analyzed regardless of adherence, which improves internal validity but could dilute effect estimates. Improvements consistent with a clinically meaningful difference in disability score must be cautiously interpreted in the per-protocol population. Improvements in health-related quality of life across analysis populations, while consistent with prior knowledge,^9^ may be spurious due to multiple testing. We did not explicitly collect delirium data, which is a relevant outcome for older adults.

## Conclusions

In this multicenter randomized clinical trial, we found that assignment of older patients with frailty preparing for elective, noncardiac surgery to a home-based prehabilitation program did not result in improvements in patient-reported disability scores or reduced complication risk. Patient-centered outcomes may be meaningfully improved with adherent participation in prehabilitation; however, intervention design and delivery require further optimization to overcome barriers to adherence.

## Notes

Supplement 1. Trial Protocol

Supplement 2. Statistical Analysis Plan

Supplement 3. eFigure 1. PRagmatic Explanatory Continuum Indicator Summary (PRECIS)-2 eTable 1. Reporting Checklist (CONSORT With Patient-Reported Outcomes and Pragmatic Extensions) eTable 2. Reporting Checklist (TIDieR) eMethods 1. Patient Engagement (Description & GRIPP-2) eMethods 2. PREPARE Prehabilitation Program eMethods 3. Description of Adherence Optimization Coaching Strategies eMethods 4. Expanded Rationale for Sample Size Increase eMethods 5. Theoretical Domains Framework Survey eMethods 6. Statistical Analysis of Secondary Outcomes eMethods 7. Changes in Activity Levels eTable 3. Baseline Characteristics of Modified Intention to Treat Population eTable 4. Missing Data eTable 5. Baseline Characteristics of Adherent Population eTable 6. Incidence of Safety Outcomes From Randomization to Surgery eMethods 8. Patient Reported Barriers to Prehabilitation Adherence eFigure 2. Patient-Reported Barriers to Prehabilitation Adherence eTable 7. Interaction P Values for Subgroup Effect Modifier Analyses eTable 8. Complication Subtypes Within Primary Outcome eFigure 3. World Health Organization Disability Assessment Schedule Domain Scores at Baseline and 30 Days eTable 9. Results of Per-Protocol Analyses at 30-Day Follow-Up eTable 10. Results of Per-Protocol Analyses for Hospitalization Outcomes

Supplement 4. Data Sharing Statement

## References

[1] Weiser TG, Haynes AB, Molina G, . Estimate of the global volume of surgery in 2012: an assessment supporting improved health outcomes. Lancet. 2015;385(suppl 2):S11. doi:10.1016/S0140-6736(15)60806-6 26313057

[2] Wijeysundera DN, Pearse RM, Shulman MA, ; METS study investigators. Assessment of functional capacity before major non-cardiac surgery: an international, prospective cohort study. Lancet. 2018;391(10140):2631-2640. doi:10.1016/S0140-6736(18)31131-0 30070222

[3] McIsaac DI, Taljaard M, Bryson GL, . Frailty as a predictor of death or new disability after surgery: a prospective cohort study. Ann Surg. 2020;271(2):283-289. doi:10.1097/SLA.0000000000002967 30048320

[4] Shulman MA, Myles PS, Chan MTV, McIlroy DR, Wallace S, Ponsford J. Measurement of disability-free survival after surgery. Anesthesiology. 2015;122(3):524-536. doi:10.1097/ALN.0000000000000586 25689757

[5] Kim DH, Rockwood K. Frailty in older adults. N Engl J Med. 2024;391(6):538-548. doi:10.1056/NEJMra2301292 39115063 PMC11634188

[6] Aucoin SD, Hao M, Sohi R, . Accuracy and feasibility of clinically applied frailty instruments before surgery: a systematic review and meta-analysis. Anesthesiology. 2020;133(1):78-95. doi:10.1097/ALN.0000000000003257 32243326

[7] Fleurent-Grégoire C, Burgess N, McIsaac DI, . Towards a common definition of surgical prehabilitation: a scoping review of randomised trials. Br J Anaesth. 2024;133(2):305-315. doi:10.1016/j.bja.2024.02.035 38677949 PMC11282475

[8] McIsaac DI, Gill M, Boland L, ; Prehabilitation Knowledge Network. Prehabilitation in adult patients undergoing surgery: an umbrella review of systematic reviews. Br J Anaesth. 2022;128(2):244-257. doi:10.1016/j.bja.2021.11.014 34922735

[9] McIsaac DI, Kidd G, Gillis C, . Relative efficacy of prehabilitation interventions and their components: systematic review with network and component network meta-analyses of randomised controlled trials. BMJ. 2025;388:e081164. doi:10.1136/bmj-2024-081164 39843215 PMC11752451

[10] Shaw JF, Pilon S, Vierula M, McIsaac DI. Predictors of adherence to prescribed exercise programs for older adults with medical or surgical indications for exercise: a systematic review. Syst Rev. 2022;11(1):80. doi:10.1186/s13643-022-01966-9 35488307 PMC9052492

[11] Mullens CL, Collins RA, Kunnath N, Probst JC, Ibrahim AM. Trends in travel time to obtain surgical care for rural patients. JAMA. 2025;333(16):1453-1455. doi:10.1001/jama.2025.0447 39937470 PMC11822571

[12] Barbieri S, Jorm L. Travel times to hospitals in Australia. Sci Data. 2019;6(1):248. doi:10.1038/s41597-019-0266-4 31676758 PMC6825170

[13] Ng E, Wilkins R, Perras A. How far is it to the nearest hospital? calculating distances using the Statistics Canada Postal Code Conversion File. Health Rep. 1993;5(2):179-188. 8292757

[14] Bethell J, Puts MTE, Sattar S, . The Canadian Frailty Priority Setting Partnership: research priorities for older adults living with frailty. Can Geriatr J. 2019;22(1):23-33. doi:10.5770/cgj.22.336 31501680 PMC6707135

[15] McIsaac DI, Hladkowicz E, Bryson GL, . Home-based prehabilitation with exercise to improve postoperative recovery for older adults with frailty having cancer surgery: the PREHAB randomised clinical trial. Br J Anaesth. 2022;129(1):41-48. doi:10.1016/j.bja.2022.04.006 35589429

[16] Carli F, Bousquet-Dion G, Awasthi R, . Effect of multimodal prehabilitation vs postoperative rehabilitation on 30-day postoperative complications for frail patients undergoing resection of colorectal cancer: a randomized clinical trial. JAMA Surg. 2020;155(3):233-242. doi:10.1001/jamasurg.2019.5474 31968063 PMC6990653

[17] Barnes K, Hladkowicz E, Dorrance K, . Barriers and facilitators to participation in exercise prehabilitation before cancer surgery for older adults with frailty: a qualitative study. BMC Geriatr. 2023;23(1):356. doi:10.1186/s12877-023-03990-3 37280523 PMC10242997

[18] Atkins L, Francis J, Islam R, . A guide to using the Theoretical Domains Framework of behaviour change to investigate implementation problems. Implement Sci. 2017;12(1):77. doi:10.1186/s13012-017-0605-9 28637486 PMC5480145

[19] Loudon K, Treweek S, Sullivan F, Donnan P, Thorpe KE, Zwarenstein M. The PRECIS-2 tool: designing trials that are fit for purpose. BMJ. 2015;350:h2147. doi:10.1136/bmj.h2147 25956159

[20] Treweek S, Bevan S, Bower P, . Trial Forge Guidance 1: what is a Study Within A Trial (SWAT)? Trials. 2018;19(1):139. doi:10.1186/s13063-018-2535-5 29475444 PMC5824570

[21] McIsaac DI, Fergusson DA, Khadaroo R, ; PREPARE Investigators. PREPARE trial: a protocol for a multicentre randomised trial of frailty-focused preoperative exercise to decrease postoperative complication rates and disability scores. BMJ Open. 2022;12(8):e064165. doi:10.1136/bmjopen-2022-064165 35940835 PMC9364396

[22] McIsaac DI. Statistical analysis plan: PREPARE trial. OSF. https://osf.io/dxe7y/metadata/osf

[23] Schulz KF, Altman DG, Moher D; CONSORT Group. CONSORT 2010 statement: updated guidelines for reporting parallel group randomised trials. BMJ. 2010;340:c332. doi:10.1136/bmj.c332 20332509 PMC2844940

[24] Hoffmann TC, Glasziou PP, Boutron I, . Better reporting of interventions: template for intervention description and replication (TIDieR) checklist and guide. BMJ. 2014;348:g1687. doi:10.1136/bmj.g1687 24609605

[25] Staniszewska S, Brett J, Simera I, . GRIPP2 reporting checklists: tools to improve reporting of patient and public involvement in research. BMJ. 2017;358:j3453. doi:10.1136/bmj.j3453 28768629 PMC5539518

[26] Calvert M, Kyte D, Mercieca-Bebber R, ; the SPIRIT-PRO Group. Guidelines for inclusion of patient-reported outcomes in clinical trial protocols: the SPIRIT-PRO extension. JAMA. 2018;319(5):483-494. doi:10.1001/jama.2017.21903 29411037

[27] Zwarenstein M, Treweek S, Gagnier JJ, ; CONSORT group; Pragmatic Trials in Healthcare (Practihc) group. Improving the reporting of pragmatic trials: an extension of the CONSORT statement. BMJ. 2008;337:a2390. doi:10.1136/bmj.a2390 19001484 PMC3266844

[28] Searle SD, Mitnitski A, Gahbauer EA, Gill TM, Rockwood K. A standard procedure for creating a frailty index. BMC Geriatr. 2008;8(24):24. doi:10.1186/1471-2318-8-24 18826625 PMC2573877

[29] Perioperative care of people living with frailty. Centre for Perioperative Care. Accessed March 27, 2023. https://cpoc.org.uk/guidelines-resources-guidelines/perioperative-care-people-living-frailty

[30] Swarbrick CJ, Williams K, Evans B, ; SNAP-3 collaborators. Postoperative outcomes in older patients living with frailty and multimorbidity in the UK: SNAP-3, a snapshot observational study. Br J Anaesth. 2025;135(1):166-176. doi:10.1016/j.bja.2025.04.026 40425395 PMC12226742

[31] Haddad T, Mulpuru S, Salter I, . Development and evaluation of an evidence-based, theory-grounded online Clinical Frailty Scale tutorial. Age Ageing. 2022;51(2):afab258. doi:10.1093/ageing/afab258 35136898

[32] Canada’s food guide. Government of Canada. Accessed March 4, 2020. https://food-guide.canada.ca/en/

[33] World Health Organization. Global recommendations on physical activity for health. https://www.who.int/docs/default-source/physical-activity/information-sheet-global-recommendations-on-physical-activity-for-health/physical-activity-recommendations-65years.pdf 26180873

[34] Gillis C, Li C, Lee L, . Prehabilitation versus rehabilitation: a randomized control trial in patients undergoing colorectal resection for cancer. Anesthesiology. 2014;121(5):937-947. doi:10.1097/ALN.0000000000000393 25076007

[35] Borg GA. Psychophysical bases of perceived exertion. Med Sci Sports Exerc. 1982;14(5):377-381. doi:10.1249/00005768-198205000-00012 7154893

[36] Laporte M, Keller HH, Payette H, . Validity and reliability of the new Canadian Nutrition Screening Tool in the ‘real-world’ hospital setting. Eur J Clin Nutr. 2015;69(5):558-564. doi:10.1038/ejcn.2014.270 25514899

[37] Michie S, Johnston M, Francis J, Hardeman W, Eccles M. From theory to intervention: mapping theoretically derived behavioural determinants to behaviour change techniques. Appl Psychol. 2008;57(4):660-680. doi:10.1111/j.1464-0597.2008.00341.x

[38] Measuring health and disability: manual for WHO Disability Assessment Schedule (WHODAS 2.0). World Health Organization. https://www.who.int/publications/i/item/measuring-health-and-disability-manual-for-who-disability-assessment-schedule-(-whodas-2.0)/

[39] Shulman MA, Kasza J, Myles PS. Defining the minimal clinically important difference and patient-acceptable symptom state score for disability assessment in surgical patients. Anesthesiology. 2020;132(6):1362-1370. doi:10.1097/ALN.0000000000003240 32167984

[40] Grocott MPW, Browne JP, Van der Meulen J, . The Postoperative Morbidity Survey was validated and used to describe morbidity after major surgery. J Clin Epidemiol. 2007;60(9):919-928. doi:10.1016/j.jclinepi.2006.12.003 17689808

[41] Clavien PA, Strasberg SM. Severity grading of surgical complications. Ann Surg. 2009;250(2):197-198. doi:10.1097/SLA.0b013e3181b6dcab 19638901

[42] EuroQol Group. EuroQol–a new facility for the measurement of health-related quality of life. Health Policy. 1990;16(3):199-208. doi:10.1016/0168-8510(90)90421-9 10109801

[43] Xie F, Pullenayegum E, Gaebel K, ; Canadian EQ-5D-5L Valuation Study Group. A time trade-off-derived value set of the EQ-5D-5L for Canada. Med Care. 2016;54(1):98-105. doi:10.1097/MLR.0000000000000447 26492214 PMC4674140

[44] Goldberg A, Chavis M, Watkins J, Wilson T. The five-times-sit-to-stand test: validity, reliability and detectable change in older females. Aging Clin Exp Res. 2012;24(4):339-344. doi:10.1007/BF03325265 23238309

[45] Katz S, Ford AB, Moskowitz RW, Jackson BA, Jaffe MW. Studies of illness in the aged. the index of ADL: a standardized measure of biological and psychosocial function. JAMA. 1963;185:914-919. doi:10.1001/jama.1963.03060120024016 14044222

[46] Huijg JM, Gebhardt WA, Crone MR, Dusseldorp E, Presseau J. Discriminant content validity of a theoretical domains framework questionnaire for use in implementation research. Implement Sci. 2014;9(1):11. doi:10.1186/1748-5908-9-11 24423394 PMC3896680

[47] Kahan BC, Jairath V, Doré CJ, Morris TP. The risks and rewards of covariate adjustment in randomized trials: an assessment of 12 outcomes from 8 studies. Trials. 2014;15(1):139. doi:10.1186/1745-6215-15-139 24755011 PMC4022337

[48] Kahan BC. Accounting for centre-effects in multicentre trials with a binary outcome - when, why, and how? BMC Med Res Methodol. 2014;14(1):20. doi:10.1186/1471-2288-14-20 24512175 PMC3923100

[49] Molenaar CJL, Minnella EM, Coca-Martinez M, ; PREHAB Study Group. Effect of multimodal prehabilitation on reducing postoperative complications and enhancing functional capacity following colorectal cancer surgery: the PREHAB randomized clinical trial. JAMA Surg. 2023;158(6):572-581. doi:10.1001/jamasurg.2023.0198 36988937 PMC10061316

[50] Onerup A, Andersson J, Angenete E, . Effect of short-term homebased pre- and postoperative exercise on recovery after colorectal cancer surgery (PHYSSURG-C): a randomized clinical trial. Ann Surg. 2022;275(3):448-455. doi:10.1097/SLA.0000000000004901 33843798 PMC8820776

[51] Hatef E, Wilson RF, Hannum SM, . Use of Telehealth During the COVID-19 Era. Agency for Healthcare Research and Quality; 2023, doi:10.23970/AHRQEPCSRCOVIDTELEHEALTH.

[52] Minnella EM, Awasthi R, Loiselle SE, Agnihotram RV, Ferri LE, Carli F. Effect of exercise and nutrition prehabilitation on functional capacity in esophagogastric cancer surgery: a randomized clinical trial. JAMA Surg. 2018;153(12):1081-1089. doi:10.1001/jamasurg.2018.1645 30193337 PMC6583009

[53] Gillis C, Buhler K, Bresee L, . Effects of nutritional prehabilitation, with and without exercise, on outcomes of patients who undergo colorectal surgery: a systematic review and meta-analysis. Gastroenterology. 2018;155(2):391-410.e4. doi:10.1053/j.gastro.2018.05.012 29750973

[54] Courneya KS, Vardy JL, O’Callaghan CJ, ; CHALLENGE Investigators. Structured exercise after adjuvant chemotherapy for colon cancer. N Engl J Med. 2025;393(1):13-25. doi:10.1056/NEJMoa2502760 40450658

[55] Riley RD, Tierney JF, Stewart LA, eds. Individual Participant Data Meta-Analysis: A Handbook for Healthcare Research. Wiley; 2021. doi:10.1002/9781119333784

[56] Ke Y, Tay VY, Leong YH, . The role of wearable technology in home-based prehabilitation: a scoping review. Br J Anaesth. 2025;134(1):228-231. doi:10.1016/j.bja.2024.09.024 39505592

[57] Buell KG, Spicer AB, Casey JD, . Individualized treatment effects of oxygen targets in mechanically ventilated critically ill adults. JAMA. 2024;331(14):1195-1204. doi:10.1001/jama.2024.2933 38501205 PMC10951851

