# Supplementary material for: Home-Based Prehabilitation for Older Surgical Patients With Frailty: A Randomized Clinical Trial
Source: JAMA Surg. 2025 Dec 3;161(2):113–23. doi: 10.1001/jamasurg.2025.5288 (PMC12676472; doi:10.1001/jamasurg.2025.5288)
Supplement: Supplement 1. — Trial Protocol [file jamasurg-e255288-s001.pdf]

This supplement contains the following items:

1. Original protocol, final protocol, summary of changes.
2. Final statistical analysis plan.

THE PREPARE TRIAL

PREPARE Trial: a parallel arm multicenter randomized trial of frailty-focused PReoperative Exercise to decrease PostoperAtive complication Rates and disability scorEs

CLINICAL RESEARCH PROTOCOL

v.1.0

26-November-2019

**Sponsor Investigator/Principal Investigator:**	Daniel McIsaac, MD, MPH, FRCPC<br>Department of Anesthesiology and Pain Medicine<br>The Ottawa Hospital<br>1053 Carling Avenue<br>Ottawa ON K1Y 4E9
**Co-Principal Investigators:**	Dean Fergusson, PhD, MHA<br>Rachel Khadaroo, MD, PhD, FRCSC, FACS<br>John Muscedere, MD, FRCPC<br>Monica Taljaard, PhD
**Site Investigators:**	Husein Moloo, MD, MSc, FRCSC<br>Sylvain Gagne, MD, FRCPC<br>Rodney Breau, MD, MSc, FRCSC<br>Daniel Trottier, MD, FRCSC<br>Jean Michel Aubin, MD, FRCSC<br>Grace Ma, MD, FRCSC<br>Tarit Saha, MD, FRCPC<br>Antoine Eskander, MD, ScM, FRCSC<br>Rachel Khadaroo, MD, PhD, FRCSC, FACS<br>Ilun Yang, MD, FRCSC<br>Thomas Mutter, MD, FRCPC<br>Elijah Dixon, MD, BSc, MSc(Epi), FRCSC, FACS
**Funders:**	Canadian Institutes of Health Research (CIHR)<br>The Ottawa Hospital Academic Medical Organization (TOHAMO)
**Coordinating Centre:**	Department of Anesthesiology and Pain Medicine<br>The Ottawa Hospital<br>1053 Carling Avenue<br>Ottawa, ON K1Y 4E9

DOCUMENT HISTORY

Version	Version Date	Changes
1.0	26-NOVEMBER-2019	New document

PROTOCOL SYNOPSYS

**Study Title:**	PREPARE Trial: a parallel arm multicenter randomized trial of frailty-focused PReoperative Exercise to decrease PostoperAtive complication Rates and disability scorEs
**Protocol Short Title:**	The PREPARE Trial
**Protocol Version:**	1.0
**Protocol Date:**	26-NOVEMBER-2019
**Sponsor-Investigator:**	**Daniel McIsaac, MD, MPH, FRCPC**<br>Department of Anesthesiology and Pain Medicine<br>The Ottawa Hospital<br>1053 Carling Avenue<br>Ottawa ON K1Y 4E9
**Site Investigators:**	**Husein Moloo, MD, MSc, FRCSC**<br>The Ottawa Colorectal Group<br>The Ottawa Hospital<br>1053 Carling Avenue<br>Ottawa, ON K1Y 4E9<br><br>**Sylvain Gagne, MD, FRCPC**<br>Department of Anesthesiology and Pain Medicine<br>The Ottawa Hospital<br>501 Smyth Road,<br>Ottawa, ON K1H 8L6<br><br>**Rodney Breau, MD, MSc, FRCSC**<br>Department of Surgery, Urology<br>The Ottawa Hospital<br>501 Smyth Road,<br>Ottawa, ON K1H 8L6<br><br>**Daniel Trottier, MD, FRCSC**<br>Department of Surgery<br>Montfort Hospital<br>713 Montreal Road<br>Ottawa, ON K1K 0T2

	**Jean Michel Aubin, MD, FRCSC**<br>Department of Surgery<br>Queensway Carleton Hospital<br>3045 Baseline Road<br>Ottawa, ON K2H 8P4<br><br>**Grace Ma, MD, FRCSC**<br>Department of General Surgery<br>Health Sciences North<br>65 Larch Street<br>Sudbury, ON P3E 1B8<br><br>**Tarit Saha, MD, FRCPC**<br>Department of Anesthesia and Perioperative Medicine<br>Kingston Health Sciences Centre<br>76 Stuart Street<br>Kingston, ON K7L 2V7<br><br>**Antoine Eskander, MD, ScM, FRCSC**<br>Department of Otolaryngology<br>Sunnybrook Health Sciences Centre<br>2075 Bayview Drive<br>Toronto, ON M4N 3M5<br><br>**Rachel Khadaroo, MD, PhD, FRCSC, FACS**<br>Department of Surgery and Critical Care Medicine<br>University of Alberta Hospital<br>84409112th Street NW<br>Edmonton, AB T6G 2B7<br><br>**Ilun Yang, MD, FRCSC**<br>Department of Surgery<br>Juravinski Hospital<br>711 Concession Street<br>Hamilton, ON L8V 1C3<br><br>**Thomas Mutter, MD, FRCPC**<br>Department of Anesthesiology, Perioperative and Pain Medicine<br>Winnipeg Regional Health Authority<br>671 William Avenue<br>Winnipeg, MB R3E 0Z2<br><br>**Elijah Dixon, MD, BSc, MSc(Epi), FRCSC, FACS**<br>Department of Surgery<br>Alberta Health Services<br>1403-29th Street NW<br>Calgary, AB T2N 2T9
**Coordinating Centre:**	Department of Anesthesiology and Pain Medicine<br>The Ottawa Hospital<br>1053 Carling Avenue

	Ottawa ON, K1Y 4E9
**Funders:**	Canadian Institutes of Health Research (CIHR)<br>The Ottawa Hospital Medical Academic Organization (TOHAMO)
**Study Objectives:**	1. Improve patient-oriented disability scores (primary outcome)<br>2. Reduce complication rates, improve quality of life<br>3. Decrease healthcare resource use in older people with frailty having elective inpatient surgery
**Study Design:**	Assessor blinded multicenter individual patient parallel-arm randomized controlled trial conducted in 14 Canadian academic or community hospitals
**Number of Participants:**	750
**Study Population:**	Older adults aged ≥60 years who are undergoing major elective inpatient non-cardiac surgery
**Inclusion Criteria:**	1. Age ≥60 years<br>2. Elective surgery with expected post-surgery stay ≥2 days<br>3. Frailty present (CFS ≥4/9)<br>4. Surgery date between 3 and 12 weeks from enrollment
**Exclusion Criteria:**	1. Inability to communicate in English or French<br>2. Unreachable by telephone<br>3. Palliative surgery<br>4. Cardiac, neurological or orthopedic procedures
**Follow-up duration:**	One year

Table of Contents

1. BACKGROUND AND RATIONALE 8
1.1. The majority of Canadians who have surgery are older and many live with frailty 8
1.2. Frailty makes people vulnerable to adverse health outcomes 8
1.3. Findings from our group provide important insights into why frailty leads to adverse outcomes 8
1.4. Interventions to improve postoperative outcomes for older people with frailty are infrequently studied, however, exercise prehabilitation is a promising therapy 9
1.5. Care of older people having surgery and exercise before surgery are high-priority areas for patient-oriented research 9
1.6. Older people with frailty require tailored (i.e. type, location, duration) exercise prehabilitation programs to meet their unique needs 10
1.7. Results from our pilot trial of home-based exercise prehabilitation in cancer patients with frailty demonstrate feasibility 10
1.8. Serious limitations prevent generalizing the exercise prehabilitation literature for people *without* frailty to people *with* frailty 10
1.9. Currently registered studies will not address pertinent knowledge gaps 11
2. RESEARCH QUESTIONS 11
3. STUDY OBJECTIVES 11
4. STUDY DESIGN 11
4.2.1. Group 12
4.3. Allocation and Randomization 12
4.4. Protection Against Bias 13
4.5. Duration of Treatment 13
4.6. Inclusion/Exclusion Criteria 13
4.7. Justification of Criteria 13
4.8. Frequency and Duration to Follow-Up 14
4.9. Primary Outcomes 15
4.9.1. In-hospital complications 15
4.9.2. Patient-reported disability 30 days after surgery 15
4.10. Secondary Outcomes 16
4.10.1. Function 16
4.10.2. Health-related quality of life 16

4.10.3. All-cause mortality 16
4.10.4. Health System 16
4.10.5. Participant Feedback 16
4.10.6. Safety 16
4.11. Outcome Measurement at Follow-Up 16
4.12. Sample Size 17
4.13. Health Services Research Issues 17
4.14. Recruitment 17
4.15. Compliance 18
4.16. Loss to Follow-Up 18
4.17. Study Centres 18
5. DATA ANALYSIS 18
5.1. Primary Outcomes 18
5.2. Secondary Outcomes 19
5.3. Additional Analyses 19
5.4. Planned Subgroup Analyses 19
6. STUDY PROCEDURES 19
6.1. Informed Consent 19
6.2. Surgical Consultation Baseline Visit (between 3 and 12 weeks from date of surgery) 20
6.3. Weekly Adherence Calls 20
6.4. Day Before Surgery 20
6.5. In-Hospital Follow-Up (day 3, 5, 7 and discharge) 20
6.6. Post-Discharge Follow-Up 21
6.6.1. 30-Day Phone Follow-Up 21
6.6.2. 90-Day Phone Follow-up 21
6.6.3. One-Year Phone Follow-Up 21
6.7. Study Schedule of Events 21
7. DATA COLLECTION MANAGEMENT 22
7.1. Source Documents 22
7.2. Case Report Forms 23
7.3. Protocol Deviations 23
7.4. Record Retention 23

8. MANAGEMENT AND REPORTING OF ADVERSE EVENTS 23
9. FINANCING 24
REFERENCES 25
MAIN APPENDIX 34
SURVEY APPENDIX 44

1. BACKGROUND AND RATIONALE

The overarching problem to be addressed in the PREPARE Trial is to improve the substantial adverse postoperative outcome burden faced by older people with frailty. This will be accomplished by testing an intervention (exercise prehabilitation) which aligns with evidence-based causal pathways, in a manner relevant to patients, clinicians and the healthcare system.

1.1. The majority of Canadians who have surgery are older and many live with frailty

Most people who have major (e.g., intra-abdominal, thoracic or vascular) surgery are $\geq$65 years[1] and are part of Canada's fastest growing demographic.[2] Four out of ten older Canadians who have surgery also live with frailty,[3] a syndrome that develops due to age- and disease-related deficits that accumulate across the lifespan.[4,5]

1.2. Frailty makes people vulnerable to adverse health outcomes

Major surgery induces physiologic stress at least equivalent to sustained moderate intensity exercise.[6,7] Therefore, given the vulnerability to stressors inherent in having frailty,[5] it is not surprising that work from our group[3,8–11] and others demonstrates that frailty before surgery is associated with significant risk. Frailty predicts a 2- to 3-fold increase in rates of: new patient-reported disability;[3] major complications;[12] readmission;[13] and dying.[9,14–17] Approximately 20% of older people with frailty develop a new disability[3] and 60% experience a serious complication.[12] Half of community-dwelling older people with frailty who have major elective surgery are not discharged back home;[3,10] length of stay (LoS)[3,10,18] and costs of care are also significantly increased.[3,10]

1.3. Findings from our group provide important insights into why frailty leads to adverse outcomes

First, surgery induces substantial physical and physiologic stress, which is poorly tolerated by older people with frailty. In two population-based studies of older people having major surgery, we found that the timing of postoperative deaths was much earlier for people with frailty than without.[14,19] This suggests that the vulnerability to stressors inherent in frailty may be especially relevant after major surgical stress, findings consistent with other studies. Specifically, a recent systematic review identified frailty as the strongest predictor of postoperative complications in older people, a 2.2-fold increase.[12] Frailty is also a key risk factor for death after a complication. [20,21] These findings support a causal pathway: *frailty-related vulnerability leads to postoperative complications from which the older person with frailty cannot recover.*

Second, although most older people with frailty survive their surgery,[22] a multicenter cohort study from our group found that almost 20% of older people with preoperative frailty develop a new patient-reported disability after elective surgery, an adjusted 2.5-fold increase.[3] The ability to stand and to walk were the most severely affected domains of disability (Main Appendix),[23] which is consistent with evidence that decreased muscle mass, which is common in frailty, contributes to poor functional outcomes.[24] *This suggests that physical dysfunction is a substantial contributor to disability after surgery in older people.*

Therefore, accumulating data to support the hypothesis that to improve postoperative outcomes for older people with frailty, interventions should aim to: 1) decrease physical and physiologic vulnerability, which should translate into lower complication rates and subsequent deaths; and 2) address contributors to physical dysfunction, which should decrease disability and subsequent loss of independence.

1.4. Interventions to improve postoperative outcomes for older people with frailty are infrequently studied, however, exercise prehabilitation is a promising therapy

Despite the adverse outcome burden associated with frailty, a systematic review from our team identified only 11 studies (1 668 total participants) that tested interventions to improve the outcomes of older people with frailty having surgery.[25] Exercise prehabilitation (i.e., exercise therapy performed before surgery) was the most common and most promising of the limited number of interventions tested.[25–28] Four of five identified exercise studies found that functional outcomes were improved. Milder and colleagues, in a separate review of prehabilitation programs in people with frailty, identified three additional studies in different procedural areas where prehabilitation was associated with decreased length of stay (LoS) and mortality.[28] Two other randomized trials involving individuals with frailty characteristics (i.e., older age, higher baseline illness severity and decreased functional capacity) suggest that exercise prehabilitation may be ***most*** effective in this high-risk population. Minella and colleagues demonstrated that older people with frailty characteristics made functional gains 2-fold higher than people with high baseline function and low illness severity after participating in the same exercise prehabilitation program.[29] Barberan-Garcia found that postoperative complications were reduced in older people with frailty characteristics by 50% relative to control.[26]

1.5. Care of older people having surgery and exercise before surgery are high-priority areas for patient-oriented research

As outlined in 1. 1., the characteristics of people having surgery in Canada are changing. In the coming decades the number of Canadians $\geq$65 years is expected to double.[2] We have documented a 10% increase in the proportion of older people with frailty having surgery in the past 10 years.[9] Therefore, frailty will increasingly contribute to adverse patient and system outcomes after surgery. Accordingly, James Lind Alliance (JLA) priority setting partnerships[30,31] have defined key areas of research that must be addressed, including: 1) improving the care of older people having surgery; 2) the role of exercise in improving surgical outcomes; and 3) the role of exercise in managing frailty. However, currently available studies of exercise prehabilitation rarely include older people with frailty, lack patient-reported outcomes, evaluate resource-intensive hospital-based interventions (limiting access), have been small and single centered, and are at high risk of bias. This means that our proposed low risk of bias multicenter trial of an exercise prehabilitation program, tailored to the needs of older people with frailty, is urgently needed and directly aligns with patient- and community-centered priorities for research.

1.6. Older people with frailty require tailored (i.e. type, location, duration) exercise prehabilitation programs to meet their unique needs

Despite the limited number of studies describing exercise prehabilitation programs for people with frailty having surgery, a systematic review of 47 exercise studies for older people with frailty *not having surgery* describe the needs of this population.[32] First, exercise appears to be most effective in people with mild (as opposed to severe) frailty. Our data shows that 86% of elective surgery patients with frailty have a Clinical Frailty Scale score of 4 or 5 (i.e., vulnerable or mildly frail, the two lowest categories still considered as frail),[3,4] suggesting older people with frailty having surgery are an ideal population to benefit from exercise. Next, efficacious programs featured multicomponent training (i.e., combinations of different types of exercise), although resistance training appeared to be the most important component. Programs where exercise was performed at least three times per week for 30-45 minutes were more efficacious than lower frequency programs. While not addressed in the systematic review, priority setting partnerships demonstrate that older people with frailty prioritize home-based services,[31] which may reflect limited access to transport, decreased mobility, or other factors.[33] The need for home-based programming is also consistent with preliminary results from our ongoing systematic review of factors influencing compliance with exercise therapy in older people, which demonstrates that lack of proximity to facilities decreases program compliance.[34] Finally, in the surgical setting, systematic reviews suggest that two to four weeks of exercise prehabilitation are needed to improve post-operative outcomes.[35]

1.7. Results from our pilot trial of home-based exercise prehabilitation in cancer patients with frailty demonstrate feasibility

To date, we have recruited 189/200 participants.[36] Our results show that our home-based program, performed three times per week for at least three weeks before surgery, is feasible and acceptable to older people with frailty. We have met our weekly recruitment targets, have achieved 95% participation rates with prescribed exercise and 96% complete outcome follow up at 30 days. Feasibility results of our small trial in surgical oncology are encouraging for expanding our work to a multicenter trial across major surgical procedures (to enhance generalizability), appropriately powered for outcomes that matter most to older people (disability and complications).[37,38]

1.8. Serious limitations prevent generalizing the exercise prehabilitation literature for people *without* frailty to people *with* frailty

An alternative to conducting a multicenter trial of exercise prehabilitation in a population of older people with frailty would be to generalize findings from studies of exercise prehabilitation conducted in people *without* frailty. However, despite systematic review findings that exercise prehabilitation improves physical performance and function,[35,39–41] decreases complication rates[40,41] and reduces LoS[39,40] in younger patients (mean age <65 years) without frailty,[25,35,39–41] limitations exist that preclude generalizability. First, 87% of identified studies were at high or unclear risk of bias and 79% were single center studies with <100 participants. Second, interventions did not meet the needs of people with frailty, as 85% were not home-based, which may be especially relevant in the Canadian context where older adults are over-represented in rural areas and may need to travel long distances to hospital.[42]

Consistent with these limitations, five of six systematic reviews evaluating exercise prehabilitation (combining frail and non-frail populations) concluded that more data are needed, specifically from large, methodologically robust multicenter trials at low risk of bias.[25,28,35,39,41]

1.9. Currently registered studies will not address pertinent knowledge gaps

We have reviewed currently registered exercise prehabilitation trials. Only three trials, all with sample sizes <140, none powered for patient-reported outcomes, and all limited to organ-specific surgeries (e.g., colorectal or cardiac) are currently planned or underway. Therefore, results of ongoing trials are unlikely to address patient-important questions or provide generalizable findings applicable to older people with frailty.

2. RESEARCH QUESTIONS

In older people with frailty having major elective surgery, is participation in a structured home-based exercise program, compared to standard care, effective in:

- Decreasing patient-reported disability scores 30 days after surgery and rates of in-hospital complications?
- Improving health related quality of life and decreasing healthcare resource use?
- Reducing costs to the Canadian health care system (health services and economic outcomes)?

3. STUDY OBJECTIVES

The PREPARE Trial aims to provide randomized trial evidence evaluating whether a home-based exercise program, which we have already shown to be feasible and acceptable for older people living with frailty, can do the following:

1. Improve patient-oriented disability scores (primary outcome)
2. Reduce complication rates, improve quality of life
3. Decrease healthcare resource use in older people with frailty having elective inpatient surgery

4. STUDY DESIGN

4.1. Trial Design

The PREPARE Trial is an accessor blinded individual patient parallel-arm randomized controlled trial conducted in 14 Canadian academic or community hospitals.

4.2. Intervention

Our intervention is a structured, home-based, multimodal exercise prehabilitation program *already successfully implemented in our pilot trial.*[36] The protocol was developed with kinesiologists and exercise scientists (CSB, JN): 1) informed by a protocol with proven efficacy in improving function

for non-frail surgical patients,[45–47] 2) tailoring movements to the needs and safety of people with frailty, and 3) specific feedback from participants with frailty in our pilot trial (obtained through structured qualitative and quantitative assessment[36]). Our data demonstrate that the intervention can be feasibly implemented, that 95% of older people with frailty who are randomized to exercise participate in the program; 87% of patients report that it is: easy to follow, enjoyable, well-suited to their needs and that lack of experience with exercise was not a barrier to participation.

Exercise will be prescribed as one-hour sessions, performed a minimum of three times per week for three weeks, consisting of: 1) strength training; 2) aerobic exercise and 3) flexibility.

Strength training: 1 set of 10 repetitions of each exercise: 1) push-ups (modified to the individual's level of function as wall push-ups or knee push-ups); 2) seated row (elastic resistance band); 3) chest fly (elastic resistance band); 4) deltoid lift (elastic resistance band); 5) biceps curls (elastic resistance band); 6) triceps extensions (elastic resistance band); 7) quadricep exercises; 8) hamstring curls; 9) standing calf raises; 10) modified chair-seated abdominal crunches.

Aerobics: Participants are asked to walk for 20 minutes at moderate intensity. After the first week, the individual's average daily step count is used to recommend a 10% increase in daily step count each week. People with frailty typically have low baseline step counts,[48] and a 10% increase per week is considered to be a safe, meaningful and achievable method to personalize activity goals.[49]

Flexibility: Chest, arm, leg and trunk stretches; each stretch held for 20 seconds x 2 repetitions.

At the time of enrollment participants will be provided teaching on safe performance of prescribed exercises, a pamphlet describing the exercises with text and illustrations, an instruction video and a calendar to track progress. Equipment will be provided including resistance bands (clearly labeled for graded progression of resistance over time) and a pedometer. Nutritional advice will be provided to inform participants of proper caloric and protein requirements, should they screen positive for nutrition risk factors using the CNST. Participants will be supported by our experienced central team using weekly phone calls to monitor safety, encourage compliance and provide advice on exercise progression. Participants can call the study center at any time with questions or concerns. Based on preliminary results from our pilot trial, participants will be encouraged to identify an exercise partner or support person to increase the social aspects of exercise.

4.2.1. Group

To support blinding of control participants, they will receive the World Health Organization Recommendations for Physical Activity for People ≥65 Years pamphlet, as well as A Guide to Healthy Eating for Older Adults.[50]

4.3. Allocation and Randomization

The random allocation sequence will be computer-generated by an independent statistician at the Ottawa Methods Centre using permuted blocks of randomly varying lengths, stratified by center,

planned minimally invasive vs. open surgery (minimally invasive surgery is associated with decreased rates of complications and faster recovery[51,52]) and cancer surgery. Study personnel will access the randomization sequence via a central web-based application to ensure allocation concealment.

4.4. Protection Against Bias

Use of minimal exclusion criteria and inclusion of a wide variety of surgical procedures will enhance external validity. Randomization will be stratified by center and surgical approach to minimize the risk of confounding. Performance bias will be minimized by randomizing participants after meeting their surgeon, allowing clinicians and outcome assessors to remain blinded to treatment allocation. The risks of response bias (due to reduced ability to blind patients to their allocated interventions) will be minimized by informing participants in both arms that they are being enrolled in a trial to test activity interventions before surgery and by providing control participants with an activity guideline. Missing data bias will be reduced through measures to minimize loss to follow-up (centralization of telephone follow-up and collection of data in-hospital) and through adjustment of factors associated with missingness.

4.5. Duration of Treatment

Exercise prehabilitation ***will be prescribed for at least three weeks:*** 1) Two to four weeks of exercise appear to be necessary to improve postoperative outcomes;[35] 2) A relevant exercise program should be effective in less than four weeks (this a benchmark duration from decision to operate to surgery in oncology);[53,54] and 3) A similar protocol in people without frailty demonstrated efficacy in a median of 24 days.[45] We will take a pragmatic approach above the minimum to account for variable waits for surgery between hospitals and indications for surgery. Our preliminary data in people with frailty demonstrate an average of five weeks of preoperative participation.

4.6. Inclusion/Exclusion Criteria

Inclusion criteria	Exclusion criteria
≥60 years having elective surgery with eLoS ≥2 days	Unable to communicate in English/French
Frailty present (CFS≥4/9)	Unreachable by telephone
Surgery date between 3 and 12 weeks from enrollment	Palliative surgery
	Cardiac, neurological or orthopedic procedures

CFS: Clinical Frailty Scale; eLoS: expected length of stay

4.7. Justification of Criteria

1) Surgical procedures: Abdominal, thoracic, pelvic, head-and-neck, and vascular procedures represent >60% of procedures for older people.[55] Cardiac, orthopedic, and neurosurgical

procedures will be excluded as processes of care, recovery rates, and reasons for disability vary substantially from other major elective surgeries.

2) Frailty instrument: The Clinical Frailty Scale (CFS) is a clinically oriented instrument highly correlated with the Canadian Study of Health and Ageing Frailty Index (ρ=0.8)[4,56] and has high inter-rater reliability.[57,58] The accuracy of the CFS is similar to the Fried Phenotype (the most frequently used perioperative frailty instrument[12,59]) but the CFS is faster and easier to use.[3] A CFS score cut off of 4/9 maximizes sensitivity and specificity for predicting disability.[3]

3) Age: Inclusion of people 60-65 years will expand our participant pool and frailty is consistently associated with a greater impact on adverse outcomes in younger people than those in advanced age groups.[14,60]

4.8. Frequency and Duration to Follow-Up

A schedule of specific data elements for collection and timing of follow up is provided in the table below. Briefly, we will collect baseline demographic, comorbidity, function and surgical data. At the time of surgery full data on program compliance and safety events will be collected. While in hospital, complications, function, frailty and resource use outcomes will be collected. Telephone follow-up will collect disability, quality of life, step count, readmission and survival data. Wearable technology linked to a mobile device could facilitate follow up, however our data shows only 22% of older surgical patients have a cell phone. All participants will be linked to health administrative data collect health system and resource use outcomes (Ontario-Institute for Clinical Evaluative Sciences (ICES); Alberta-Alberta Data Integration, Measurement & Reporting (DIMR); Manitoba-Manitoba Center for Health Policy-MCHP).

Enrollment	Leading up to Surgery	Postop in-hospital	30 days postop	90 days postop	356 days postop	Admin data
CFS,[4] PHQ-2,[61] Demographics, WHODAS,[62] EQ-5D,[63] AD8,[64] [65]DASI,[66] TUG,[67] Katz,[68] Procedure, CNST	Step Counts (intervention group), Adverse Events, Adherence, TDF Participant program feedback, Self-reported change in exercise	POMS,[69] Falls, TUG, Katz, CFS, LoS, Disposition	WHODAS, EQ-5D, Falls, Readmission, Step Count, Survival	WHODAS, EQ-5D, Falls, Readmission, Survival	WHODAS, EQ-5D, Falls, Readmission, Survival	Costs, Readmissions, Days alive at home, ED visits, Survival

AD8: AD8 Cognitive Screen; CFS: Clinical Frailty Scale; DASI: Duke Activity Status Index; ED: Emergency Department; EQ-5D: EuroQuoL health related quality of life; LoS: Length of Stay; PHQ: Patient Health Questionnaire; POMS: Postoperative Morbidity Survey; TUG: Timed Up and Go Test; WHODAS: World Health Organization Disability Assessment Schedule; CNST: Canadian Nutrition Screening Tool

4.9. Primary Outcomes

The PREPARE Trial has co-primary outcomes (disability and complications), which were identified based on: high-priority outcomes for of older surgical patients (informed by our survey of older people and patient engagement in the protocol);[37,38] 2) proposed causal mechanisms between exercise prehabilitation and outcomes; and 3) systematic review efficacy data.[35,39–41]

4.9.1. In-hospital complications

The Postoperative Morbidity Survey (POMS), a prospectively administered instrument designed to identify significant in-hospital complications in key organ systems, will be used to define complications.[69,70] Individuals experiencing any POMS complication or dying in hospital will be said to have experienced a complication. The presence or absence of a complication is recorded based on objective criteria using items available from routine sources (i.e., charts, medication records, vital signs records, routine lab tests and direct questioning of the patient). Included items were generated directly from feedback by patients, nurses and physicians. The final survey was reviewed and approved by an international panel of surgeons and anesthesiologists[70] and contains 18 items addressing nine domains (i.e., pulmonary, infectious, renal, gastrointestinal, cardiovascular, neurological, hematological, wound, pain). In validation it had high inter-rater agreement (kappa=0.94-1.0), was acceptable to all patients and demonstrated construct validity.[69] Subsequent validation studies confirmed these findings.[71,72] The POMS tool has been used in many international studies.[36,72,81,73–80] To support secondary analyses, complication severity will be graded using the widely used updated Clavien-Dindo Classification,[82–88] a validated tool which grades severity based on the therapy used to treat the complication.[82,89,90] The American Society for Enhanced Recovery and Perioperative Quality Initiative have recently published an international joint statement recommending POMS for measurement of postoperative complications and the Clavien-Dindo Classification for grading of severity.[91]

4.9.2. Patient-reported disability 30 days after surgery

We will use the World Health Organization Disability Assessment Schedule 2.0 (WHODAS), a patient-reported disability scale that assesses limitations in six major life domains (i.e., cognition, mobility, self-care, social interaction, life activities, participation in society).[62,92] The WHODAS has been validated in surgical patients[93] (and other acute and chronic conditions[94–100]), was used by our group in a recent multicenter cohort study of older surgical patients[3] and was identified by older surgical patients as a high-priority outcome.[3,38] Each questionnaire item is scored on a Likert scale ranging from 0 to 4. The sum of the responses is the WHODAS Disability Score (range: 0 to 48), which is expressed as a percentage of the maximum possible score (Survey Appendix). People who die prior to follow up will be scored as completely disabled. Based on normative data, a mean difference of 8% for WHODAS scores is meaningful.[39] In extensive psychometric evaluation in the non-operative setting, it had high test-retest reliability, high internal consistency, good concurrent validity, and conformity to Rasch scaling properties.[62] In a cohort study of 510 surgical patients, it

had good to excellent clinical acceptability, internal consistency, scaling properties, responsiveness, criterion validity, and construct validity.[93] The WHODAS 2.0 is thus a feasible, valid, and reliable patient-reported instrument to measure disability in older surgical patients.

4.10. Secondary Outcomes

4.10.1. Function

Daily and total step counts, measured using a pedometer, predict adverse post-hospitalization outcomes and reflect physical recovery. These will be recorded daily for the duration of the exercise program (for those in the intervention group) and 30 days after surgery (for all participants) to evaluate functional recovery.[101–103] The Timed Up and Go Test is a validated test used for measuring the risk of falls in older adults.[67,104] The Katz Index measures function in activities of daily living.[68] Any falls will be documented.

4.10.2. Health-related quality of life

The EQ-5D-5L is a well-validated instrument with Canadian valuation statistics and national implementation,[63,105,106] used to measure health-related quality of life at baseline, 30, 90, and 365 days after surgery and to inform incremental cost per quality-adjusted life year gained.

4.10.3. All-cause mortality

All deaths and death dates will be identified in-hospital or through telephone follow-up.

4.10.4. Health System

Discharge disposition (home, home with support, rehabilitation, long term care) will be prospectively collected at discharge; readmissions by telephone. Linkage to ICES/DIMR/MCHP data will allow for collection of health system costs,[107] readmissions, emergency department visits and subsequent long-term care admissions in the year after surgery.[108]

4.10.5. Participant Feedback

A Theoretical Domains Framework[109,110] participant survey will identify barriers and facilitators to participation.

4.10.6. Safety

Falls, cardiac or respiratory complications and unplanned healthcare encounters (i.e. emergency department visits, hospital admissions) will be collected during the exercise treatment period for the intervention group. Furthermore, for the control group, participants will be asked if they experienced any falls since the time of enrollment up until the day before surgery. Unplanned healthcare encounters will also be collected during the time of enrollment to the day before surgery for the intervention group through chart review. Expert adjudication will determine whether they were study-attributable.

4.11. Outcome Measurement at Follow-Up

All participants will be followed up to 1 year after surgery or death, whichever occurs first. As described in section 4.9. and 4.10., outcomes will be measured using validated instruments either in-hospital or at regularly scheduled telephone follow-up from the central study team.

4.12. Sample Size

Our total sample size of 750 participants (375 per arm) is driven by the binary co-primary outcome (i.e., in-hospital complications). We have assumed a control arm complication rate of 55%, informed by data from our prospective cohort study (where complications were measured using POMS, see unpublished data Main Appendix)[3] and by systematic reviews of complications in older people with frailty having surgery.[12] Available effect size estimates, from a recent low risk of bias RCT[26] (RR=0.5, 95%CI 0.3-0.8) and a systematic review[40] (OR 0.41, 95%CI 0.28-0.62)), suggest exercise prehabilitation can reduce postoperative complications by a relative 50%.[26,40] We would consider a target difference of a 25% relative reduction to be both clinically important and plausible in a multicenter study including diverse surgical procedures and after accounting for imperfect compliance. In keeping with our intention-to-treat analysis, a sample size of 750 participants achieves 90% power to detect our target difference (i.e., a relative difference of 25% or absolute difference of 14%) using an unpooled Z-test with an alpha significance level of 0.025 (to account for our two pairwise comparisons for our two primary outcomes). This calculation accounts for a 10% non-compliance factor largely consisting of randomized participants not having their planned surgery to reflect the real world clinical scenario.[111] We did not account for missing complications data as we anticipate complete observation as they are measured in-hospital. For the continuous co-primary outcome (i.e., WHODAS Disability Score at 30 days) this sample size achieves 98% power to detect a minimum clinically important difference of 8 points on a 100-point scale (or a difference of 7 points with 90% power) using an ANCOVA analysis at the two-sided 2.5% significance level. This calculation assumes a common standard deviation of 25, a correlation between baseline and postoperative score of 0.4 (informed by our multicenter cohort study)[3] and accounts for 8% missing data at 30 days and 10% non-compliance (as was assumed for complications).

4.13. Health Services Research Issues

As listed in 4.10.4., health-related quality of life, healthcare resource use and economic outcomes will be measured prospectively and through data linkage. Description of cost-effectiveness, cost utility and other health economic analyses are provided in the appendix.

4.14. Recruitment

We have experience successfully recruiting older individuals with frailty. Based on our preliminary data[36] 40% of older surgical patients eligible for frailty screening were successfully enrolled (94% of screen-eligible participants were willing to be screened; 50% of screened people met inclusion criteria; 85% of fully eligible individuals enroll). Enrollment in the PREPARE multicenter trial (which will recruit individuals having surgery for benign and oncologic reasons) may be higher as our pilot trial was limited to cancer patients, who are less likely to enroll in trials due to their high burden of competing appointments and adjuvant treatments.[112] In our multicenter cohort study, 85% of screen-eligible individuals were enrolled.[3] *Based on surgical volumes of people ≥60 years*

over the past two years at confirmed study centers (Main Appendix), and estimating 40% successful enrollment, we anticipate that 30 patients per month will be recruited.

4.15. Compliance

Systematic reviews demonstrate high variation in compliance with exercise prehabilitation (16-100%),[41] and that dropout rates in exercise interventions typically exceed 20%.[113] Preliminary results from our pilot study demonstrate 95% participation (i.e., <5% dropout or non-participation after being randomized to exercise), and that participants complete 72% of all prescribed exercise, which is higher than the average completion rate from our systematic review of prescribed exercise in older people (mean=70%).[34] We have also achieved 83% compliance in a separate study from our group of exercise in older surgical patients with frailty.[114] Through weekly monitoring and support of participants by an experienced central team and enhancements of the PREPARE Trial intervention (personalization of goals and encouragement of social engagement with an exercise partner) we expect to meet or exceed the level of compliance found in our pilot study.

4.16. Loss to Follow-Up

Some randomized patients (11% in our preliminary data) will not have their planned surgery (this may occur due to cancellation or disease progression requiring emergency surgery), but will be included in the intention to treat (ITT) population to avoid introducing bias.[111] For example, if an exercise-related adverse event lead to cancellation of surgery this must be maintained in the analysis, as would a surgery that was cancelled due to exercise-related decreases in symptoms negating the need for surgery. These individuals will have their follow up dates set based on their initially planned surgery date. We have achieved 96% complete outcome data in our single center study, and >92% in a previous multicenter study.[3,36]

4.17. Study Centres

Our study will recruit patients from 14 centers in Ottawa, Toronto, Hamilton, Edmonton, Sudbury, Kingston, Calgary and Winnipeg. These study centers represent academic and community hospitals.

5. DATA ANALYSIS

Analysis will follow ITT principles. The ITT population will be defined as all randomized participants.[111] Descriptive statistics will compare study arms at baseline. Factors associated with missing outcome data will be examined using logistic regression.

5.1. Primary Outcomes

In-hospital complications will be analyzed using robust Poisson regression to yield relative risk estimates[115,116] and 97.5% confidence intervals. The analysis will include fixed terms for study arm, the stratification factor (surgical approach), and prespecified covariates (as adjustment for known prognostic factors can substantially increase power[117–119]): age, gender, surgery type, malignancy, frailty score, and factors associated with attrition,[120,121] and will account for the center effect. Absolute risk differences will also be reported.

WHODAS Disability score at 30 days will be analyzed using ANCOVA with the baseline measure entered as a covariate,[122] together with terms for the stratification factor and the prespecified covariates listed above. A random effect for center will account for the multicenter trial design. The intervention effect will be expressed as an adjusted mean difference with 97.5% confidence interval. Secondary repeated measures analyses of all disability score measurements (up to 365 days) will use restricted maximum likelihood estimation and model the covariance matrix to account for correlation in the four repeated measures over time. The model will constrain differences between the arms at baseline[122] by including fixed terms for time and arm by time interaction in addition to the covariates specified above and the random center effect. The difference between the treatment and control arms at 90 and 365 days will be estimated using adjusted least square mean differences.

5.2. Secondary Outcomes

All adjusted analyses will account for the covariates specified in the primary analysis and will account for center effects. Health-related quality of life measures will be analyzed as described for disability. Step counts will be analyzed using linear regression. Time to hospital discharge will be analyzed using Cox regression with in-hospital mortality as a competing risk. Overall survival will be analyzed using Cox regression. Discharge disposition will be analyzed using multinomial logistic regression. Health system outcomes (readmissions, emergency department visits and subsequent long-term care admissions) will be analyzed using robust Posisson regression. Binary safety outcomes will be analyzed as described for complications using Poisson regression or exact methods if event numbers are small. Costs analysis will use log-gamma regression.[123] From the perspective of Canada's healthcare system, we will conduct a cost-utility analysis to assess whether exercise prehabilitation offers value for money. Health care utilization and the efficacy of the intervention will be obtained from the trial. We expect attrition to be low; nevertheless, to account for any missing data in our ITT analyses, all eligible patients will be included in all analyses and baseline covariates associated with missing data will be included as covariates.

5.3. Additional Analyses

We will perform a per protocol analysis (individuals who actually had surgery and with >75% completion of prescribed exercise sessions considered as the per protocol population).

5.4. Planned Subgroup Analyses

The primary outcomes will be analyzed in pre-specified subgroups that we postulate may have differing responses to the intervention: gender, age (<75 vs $\geq$75[124]), cancer, depression, frailty (4 vs $\geq$5). Compliance rates will be compared by gender. These analyses will be conducted by including interaction terms between the subgroup indicator variables and the intervention.

6. STUDY PROCEDURES

6.1. Informed Consent

The site investigator must keep the original informed consent form signed by the patient and designee. The photocopy of the signed version must be given to the patient.

Eligible patients may only be included in the study after providing written consent, or, if incapable of doing so, after such consent has been provided by a legally acceptable representative of the patient. Informed consent must be collected prior to starting any study procedures. The process of obtaining informed consent should be documented in the patient source documents.

6.2. Surgical Consultation Baseline Visit (between 3 and 12 weeks from date of surgery)

After the patient has been deemed eligible and the ICF has been signed, the following will be assessed:

- Demographic Questionnaire
- World Health Organization Disability Assessment Survey (WHODAS 2.0)
- Timed Up and Go Test (TUG)
- EuroQuol Health Related Quality of Life (EQ5D5L)
- Patient Health Questionnaire (PHQ-2)
- Duke Activity Status Index (DASI)
- Katz Index of Independence in Activities of Daily Living (KATZ)
- AD8: Cognitive Screen
- Canadian Nutrition Screening Tool (CNST)
- If the participant is deemed at risk of malnutrition based on the CNST assessment, and is randomized into the intervention group, they will be given a pamphlet on recommended oral nutrition supplements and consumption guidelines

6.3. Weekly Adherence Calls

Throughout the duration of the prehabilitation program (3 to 12 weeks from surgery date), participants will receive weekly phone calls from the central coordinating site to gauge adherence to the exercise program, suggest any modifications to the program as needed, and provide overall support and track any adverse events and falls that may have taken place.

During the participant's final adherence call, they will be asked to complete the TDF questionnaire providing their feedback on the prehabilitation program.

6.4. Day Before Surgery

On the day before surgery, research personnel will document the following:

- Step count from enrollment to surgery (intervention group)
- Self-reported change in exercise
- Number of falls (if any)
- Adverse events (if any)

6.5. In-Hospital Follow-Up (day 3, 5, 7 and discharge)

- Tiered Postoperative Morbidity Survey (POMS) (patient reported, clinician/nurse reported, chart review)

- Timed Up and Go Test (TUG)*
- Katz Index of Independence in Activities of Daily Living (KATZ)
- Clinical Frailty Scale (CFS)
- Length of Stay (LoS)*
- Disposition (discharge location)*
- Number of falls (if any)

Provided they remain in hospital, research personnel will collect data on day 3, 5, 7 and discharge. In circumstances where the patient is discharged prior to post-op day 3, only data on the date of discharge will be collected.

*Denotes data is only to be collected at date of discharge.

6.6. Post-Discharge Follow-Up

Participants will be contacted by phone at 3 time-points after hospital discharge (e.g. 30 days, 90 days and one year after surgery for follow-up). To ensure high quality standardized follow-up, post-hospital discharge assessments will be performed solely by the central coordinating site.

6.6.1. 30-Day Phone Follow-Up

Participants will receive a telephone call from the central coordinating site to document the following:

- World Health Organization Disability Assessment Survey (WHODAS 2.0)
- EuroQuol health related quality of life (EQ5D5L)
- Readmission
- Step Count
- Survival

6.6.2. 90-Day Phone Follow-up

Participants will receive a telephone call from the central coordinating site to document the following:

- World Health Organization Disability Assessment Survey (WHODAS 2.0)
- EuroQuol health related quality of life (EQ5D5L)
- Readmission
- Survival

6.6.3. One-Year Phone Follow-Up

Participants will receive a telephone call from the central coordinating site to document the following:

- World Health Organization Disability Assessment Survey (WHODAS 2.0)
- EuroQuol health related quality of life (EQ5D5L)
- Readmission
- Survival

6.7. Study Schedule of Events

	Surgical Consult	Day Before Surgery	*In-Hospital Follow-Up	30-Day Phone Follow-Up	90-Day Phone Follow-Up	One-Year Phone Follow-Up
**Inclusion/Exclusion**	x					
**Informed Consent**	x					
**Demographics**	x					
**WHODAS 2.0**	x			x	x	x
**TUG****	x		x			
**EQ5D5L**	x			x	x	x
**PHQ-2**	x					
**DASI**	x					
**KATZ**	x		x			
**AD8**	x					
**CNST**	x					
**Step Count**		x		x		
**Self-reported change in exercise (pre-op questionnaire)**		x				
**Exercise Program Adherence**						
**Number of Falls**		x	x			
**Adverse Events**		x				
**POMS**			x			
**CFS**	x		x			
**LoS****			x			
**Disposition****			x			
**Readmission**				x	x	x
**Survival**				x	x	x

*In-hospital follow-up data collection takes place on day 3, 5, 7 and discharge. If patient is discharged prior to day 3, only discharge data is collected.

**Denotes in hospital data collection that only takes place on date of discharge.

7. DATA COLLECTION MANAGEMENT

7.1. Source Documents

Source documents are original documents, data and records, or certified copies of original records of clinical findings and/or observations. (e.g. hospital records, patient charts, participant files, etc.). All data entered into the eCRF must be supported by source documents. Source documents shall be made available at the request of the sponsor-investigator.

Sites will be provided with the eCRF which is to be used as a tool for data collection. Whenever possible, source data should be transcribed directly into the eCRF. However, the eCRF will serve as the source document for any assessments that are completed. Any changes made to data already entered into the eCRF shall be done by research personnel or the site investigator. These changes will automatically be recorded within user history of the EDC system.

7.2. Case Report Forms

An Electronic Data Capture (EDC) system will be developed by the Ottawa Methods Centre and will be primarily used for study data collection. Data collection will be completed by research personnel or the site investigator. Post-discharge data will be collected via telephone by authorized research staff at the central coordinating center and subsequently entered in the eCRF. Appropriate security measure will be taken to authorize study site personnel using unique usernames and passwords prior to entering any data in the EDC.

The study data will be housed on a secure in-house server at the Ottawa Methods Centre throughout the duration of the study and up to 7 years after study completion.

All eCRF corrections will be documented within the EDC. A history of changes as well as the user who made the change will be automatically recorded.

7.3. Protocol Deviations

A protocol deviation occurs when there is a departure from the approved protocol's procedures. This departure from approved research can be made by the patient, research staff, or site investigator. When a protocol deviation occurs, the site-investigator or authorized research personnel must document and discuss the deviation with the coordinating site.

7.4. Record Retention

The site must maintain adequate and accurate records to enable the conduct of the study to be fully documented and the study data to be subsequently verified. The Investigator Site File (ISF) will contain the study's essential documents.

Study records at each site should be stored as per local requirements. If there are no local requirements, they should be retained for 7 years after the completion of the trial.

8. MANAGEMENT AND REPORTING OF ADVERSE EVENTS

There are no expected Adverse Events (or Severe Adverse Events) associated with the prehabilitation program. Surgical complications, extended length of hospital stay, readmission, and death are all possible risks of surgery.

We will track (S)AEs during in-person data collection time points (baseline, and surgical follow-up visit), and during the time from enrollment to surgery (prehabilitation period).

Appendix A will provide a list of possible adverse events that could occur during the different stages of the PREPARE Trial (baseline, prehabilitation program, and follow-up visit). If participants report any of the mentioned discomforts or symptoms, or any other (S)AEs during these timepoints, the research study staff will document it in the (S)AE Log in the EDC and will notify the site-investigator. If the site-investigator concludes that the event is deemed serious and associated with participation in the study, they shall follow local REB guidelines regarding reporting SAEs.

9. FINANCING

This study is funded by the Canadian Institutes of Health Research (CIHR) and The Ottawa Hospital Academic Medical Organization (TOHAMO).

REFERENCES

1. Etzioni DA, Liu JH, Maggard MA, Ko CY. The aging population and its impact on the surgery workforce. Ann Surg. 2003;238(2):170-177. doi:10.1097/01.SLA.0000081085.98792.3d.
2. Statistics Canada. The Canadian Population in 2011: Age and Sex. Ottawa, ON; 2011. http://www12.statcan.gc.ca/census-recensement/2011/as-sa/98-311-x/98-311-x2011001-eng.cfm#a2.
3. McIsaac DI, Taljaard M, Bryson GL, et al. Frailty as a Predictor of Death or New Disability After Surgery: A Prospective Cohort Study. Ann Surg. 2018;accepted. doi:10.1097/SLA.0000000000002967.
4. Rockwood K, Song X, MacKnight C, et al. A global clinical measure of fitness and frailty in elderly people. CMAJ. 2005;173(5):489-495. doi:10.1503/cmaj.050051.
5. Fried LP, Ferruci L, Darer J, Williamson J, Anderson G. Untangling the concepts of disability, frailty and comorbidity: Implications for improved targeting and care. J Gerontol A Biol Sci Med Sci. 2004;59(3):M255-M263.
6. Fleisher LA, Fleischmann KE, Auerbach AD, et al. 2014 ACC/AHA guideline on perioperative cardiovascular evaluation and management of patients undergoing noncardiac surgery: a report of the American College of Cardiology/American Heart Association Task Force on practice guidelines. J Am Coll Cardiol. 2014;64(22):e77-137. doi:10.1016/j.jacc.2014.07.944.
7. Jetté M, Sidney K, Blümchen G. Metabolic equivalents (METS) in exercise testing, exercise prescription, and evaluation of functional capacity. Clin Cardiol. 1990;13(8):555-565. http://www.ncbi.nlm.nih.gov/pubmed/2204507.
8. McIsaac DI, Bryson GL, Van Walraven C. Association of frailty and 1-year postoperative mortality following major elective noncardiac surgery: A population-based cohort study. JAMA Surg. 2016;151(6). doi:10.1001/jamasurg.2015.5085.
9. McIsaac DI, Moloo H, Bryson GL, Van Walraven C. The association of frailty with outcomes and resource use after emergency general surgery: A population-based cohort study. Anesth Analg. 2017;124(5). doi:10.1213/ANE.0000000000001960.
10. McIsaac DI, Beaule PE, Bryson GL, van Walraven C. The impact of frailty on outcomes and healthcare resource utilization after total joint arthroplasty: a population-based cohort study. Bone Jt J. 2016;98:799-805.
11. McIsaac DI, Wong CA, Huang A, Moloo H, van Walraven C. Derivation and Validation of a Generalizable Preoperative Frailty Index Using Population-based Health Administrative Data. Ann Surg. 2018;epub. doi:10.1097/SLA.0000000000002769.
12. Watt J, Tricco AC, Talbot-Hamon C, et al. Identifying older adults at risk of harm following elective surgery: a systematic review and meta-analysis. BMC Med. 2018;16(1):2. doi:10.1186/s12916-017-0986-2.
13. Li Y, Pederson J, Churchill T, et al. Impact of frailty on outcomes after discharge in older surgical patients: a prospective cohort study. CMAJ. 2018;190(7):184-190. doi:10.1503/cmaj.161403.
14. McIsaac DI, Bryson GL, van Walraven C. Association of Frailty and 1-Year Postoperative Mortality Following Major Elective Noncardiac Surgery: A Population-Based Cohort Study. JAMA Surg. 2016;151(6):538-545. doi:10.1001/jamasurg.2015.5085.

15. Kim DH, Kim CA, Placide S, Lipsitz LA, Marcantonio ER. Preoperative Frailty Assessment and Outcomes at 6 Months or Later in Older Adults Undergoing Cardiac Surgical Procedures. Ann Intern Med. 2016. doi:10.7326/M16-0652.
16. Lin H-S, Watts JN, Peel NM, Hubbard RE. Frailty and post-operative outcomes in older surgical patients: a systematic review. BMC Geriatr. 2016;16(1):157. doi:10.1186/s12877-016-0329-8.
17. Wang J, Zou Y, Zhao J, et al. The Impact of Frailty on Outcomes of Elderly Patients After Major Vascular Surgery: A Systematic Review and Meta-analysis. Eur J Vasc Endovasc Surg. August 2018. doi:10.1016/j.ejvs.2018.07.012.
18. Cooper Z, Rogers SO, Ngo L, et al. Comparison of Frailty Measures as Predictors of Outcomes After Orthopedic Surgery. J Am Geriatr Soc. 2016. doi:10.1111/jgs.14387.
19. McIsaac DI, Moloo H, Bryson GL, van Walraven C. The Association of Frailty With Outcomes and Resource Use After Emergency General Surgery: A Population-Based Cohort Study. Anesth Analg. 2017;124(5):1653-1661. doi:10.1213/ANE.0000000000001960.
20. Shah R, Attwood K, Arya S, et al. Association of Frailty With Failure to Rescue After Low-Risk and High-Risk Inpatient Surgery. JAMA Surg. 2018;48202:e180214. doi:10.1001/jamasurg.2018.0214.
21. Arya S, Kim SI, Duwayri Y, et al. Frailty increases the risk of 30-day mortality, morbidity, and failure to rescue after elective abdominal aortic aneurysm repair independent of age and comorbidities. J Vasc Surg. 2015;61(2):324-331. doi:10.1016/j.jvs.2014.08.115.
22. McIsaac DI, Wijeysundera DN, Huang A, Bryson GL, van Walraven C. Association of the Hospital Volume of Frail Surgical Patients Cared for with Outcomes after Elective, Major Noncardiac Surgery: A Retrospective Population-based Cohort Study. Anesthesiology. 2017;126(4):602-613. doi:10.1097/ALN.0000000000001536.
23. World Health Organization. International Classification of Functioning, Disability and Health. Geneva
24. Han A, Bokshan SL, Marcaccio SE, DePasse JM, Daniels AH. Diagnostic Criteria and Clinical Outcomes in Sarcopenia Research: A Literature Review. J Clin Med. 2018;7(4). doi:10.3390/jcm7040070.
25. McIsaac DI, Jen T, Mookerji N, Patel A, Lalu MM. Interventions to improve the outcomes of frail people having surgery: A systematic review. Quinn TJ, ed. PLoS One. 2017;12(12):e0190071. doi:10.1371/journal.pone.0190071.
26. Barberan-Garcia A, Ubré M, Roca J, et al. Personalised Prehabilitation in High-risk Patients Undergoing Elective Major Abdominal Surgery : A Randomized Blinded Controlled Trial. Ann Surg. 2018;267(1):50-56. doi:10.1097/SLA.0000000000002293.
27. Scheede-Bergdahl C, Awasthi R, Munden J, Loiselle SE, Carli F. Multimodal prehabilitation in cancer patients. Who benefits? Can J Anaesth. 2015;62(1):1496-8975.
28. Milder DA, Pillinger NL, Kam PCA. The role of prehabilitation in frail surgical patients: A systematic review. Acta Anaesthesiol Scand. 2018;(July):1-11. doi:10.1111/aas.13239.
29. Minnella EM, Awasthi R, Gillis C, et al. Patients with poor baseline walking capacity are most likely to improve their functional status with multimodal prehabilitation. Surgery. 2016;160(4):1070-1079. doi:10.1016/j.surg.2016.05.036.
30. Boney O, Bell M, Bell N, et al. Identifying research priorities in anaesthesia and perioperative care: final report of the joint National Institute of Academic Anaesthesia/James Lind Alliance Research Priority Setting Partnership. BMJ Open. 2015;5(12):e010006. doi:10.1136/bmjopen-2015-010006.

31. Canadian Frailty Network (CFN). Top Ten Frailty Priorities. http://www.cfn-nce.ca/engagingcanadians/helping-to-set-frailty-priorities/top-ten-frailty-priorities-identified-by-citizens/. Published 2017. Accessed July 17, 2018.
32. Theou O, Stathokostas L, Roland KP, et al. The effectiveness of exercise interventions for the management of frailty: a systematic review. J Aging Res. 2011;2011:569194. doi:10.4061/2011/569194.
33. Frost R, Kharicha K, Jovicic A, et al. Identifying acceptable components for home-based health promotion services for older people with mild frailty: A qualitative study. Health Soc Care Community. 2018;26(3):393-403. doi:10.1111/hsc.12526.
34. Pilon S, Vierula M, McIsaac DI. Evaluation of factors that predict exercise compliance in older people with medical and surgical conditions. In: Candian Anesthesiology Society Annual Meeting. Montreal, QC; 2018.
35. Mainini C, Rebelo PF, Bardelli R, et al. Perioperative physical exercise interventions for patients undergoing lung cancer surgery: What is the evidence? SAGE open Med. 2016;4:2050312116673855. doi:10.1177/2050312116673855.
36. McIsaac DI, Saunders C, Hladkowicz E, et al. PREHAB study: a protocol for a prospective randomised clinical trial of exercise therapy for people living with frailty having cancer surgery. BMJ Open. 2018;8(6):e022057. doi:10.1136/bmjopen-2018-022057.
37. Fried TR, Bradley EH, Towle VR, Allore H. Understanding the treatment preferences of seriously ill patients. N Engl J Med. 2002;346(14):1061-1066. doi:10.1056/NEJMsa012528.
38. Shaw J, Beasley E, Hladkowicz E, et al. A survey of older people after major surgery: Prioritization of routine and patient-reported postoperative outcome measures. In: Ottawa Quaity & Patient Safety Conference. Ottawa, ON; 2018.
39. Santa Mina D, Clarke H, Ritvo P, et al. Effect of total-body prehabilitation on postoperative outcomes: a systematic review and meta-analysis. Physiotherapy. 2014;100(3):196-207. doi:10.1016/j.physio.2013.08.008.
40. Marmelo F, Rocha V, Gonçalves D. The impact of prehabilitation on post-surgical complications in patients undergoing non-urgent cardiovascular surgical intervention: Systematic review and metaanalysis. Eur J Prev Cardiol. January 2018:204748731775237. doi:10.1177/2047487317752373.
41. Luther A, Gabriel J, Watson RP, Francis NK. The Impact of Total Body Prehabilitation on PostOperative Outcomes After Major Abdominal Surgery: A Systematic Review. World J Surg. 2018. doi:10.1007/s00268-018-4569-y.
42. Laurent S. Rural Canada: Access to Health Care.; 2002. http://publications.gc.ca/CollectionR/LoPBdP/BP/prb0245-e.htm#Summary.
43. Straus SE, Tetroe J, Graham I. Defining knowledge translation. CMAJ. 2009;181(3-4):165-168. doi:10.1503/cmaj.081229.
44. Canadian Institutes of Health Research. Guide to Knowledge Translation Planning at CIHR: Integrated and End-of-Grant Approaches. http://www.cihr-irsc.gc.ca/e/45321.html#a3. Accessed June 8, 2018.
45. Gillis C, Li C, Lee L, et al. Prehabilitation versus rehabilitation: a randomized control trial in patients undergoing colorectal resection for cancer. Anesthesiology. 2014;121(5):937-947. doi:10.1097/ALN.0000000000000393.

46. Li C, Carli F, Lee L, et al. Impact of a trimodal prehabilitation program on functional recovery after colorectal cancer surgery: a pilot study. Surg Endosc. 2013;27(4):1072-1082. doi:10.1007/s00464012-2560-5.
47. Minnella EM, Awasthi R, Loiselle S-E, Agnihotram R V, Ferri LE, Carli F. Effect of Exercise and Nutrition Prehabilitation on Functional Capacity in Esophagogastric Cancer Surgery: A Randomized Clinical Trial. JAMA Surg. September 2018. doi:10.1001/jamasurg.2018.1645.
48. Tudor-Locke C, Craig CL, Aoyagi Y, et al. How many steps/day are enough? For older adults and special populations. Int J Behav Nutr Phys Act. 2011;8(1):80. doi:10.1186/1479-5868-8-80.
49. Stovitz SD, VanWormer JJ, Center BA, Bremer KL. Pedometers as a means to increase ambulatory activity for patients seen at a family medicine clinic. J Am Board Fam Pract. 18(5):335343. http://www.ncbi.nlm.nih.gov/pubmed/16148243.
50. World Health Organization. Global recommendations on physical activity for health 65 years and above. http://www.who.int/dietphysicalactivity/physical-activity-recommendations-65years.pdf?ua=1. Accessed July 17, 2018.
51. Vennix S, Pelzers L, Bouvy N, et al. Laparoscopic versus open total mesorectal excision for rectal cancer. Cochrane database Syst Rev. 2014;(4):CD005200. doi:10.1002/14651858.CD005200.pub3.
52. Fujii S, Tsukamoto M, Fukushima Y, et al. Systematic review of laparoscopic vs open surgery for colorectal cancer in elderly patients. World J Gastrointest Oncol. 2016;8(7):573-582. doi:10.4251/wjgo.v8.i7.573.
53. Ontario CC. Target Wait Times for Cancer Surgery in Ontario. https://www.cancercareontario.ca/en/guidelines-advice/types-of-cancer/3211. Accessed July 20, 2018.
54. NHS Cancer Plan. London, UK http://webarchive.nationalarchives.gov.uk/20130222181549/http://www.dh.gov.uk/prod_consum_d h/groups/dh_digitalassets/@dh/@en/documents/digitalasset/dh_4014513.pdf.
55. Redelmeier DA, Thiruchelvam D, Daneman N. Delirium after elective surgery among elderly patients taking statins. CMAJ. 2008;179(7):645-652. doi:10.1503/cmaj.080443.
56. Mitnitski a B, Mogilner a J, Rockwood K. Accumulation of deficits as a proxy measure of aging. ScientificWorldJournal. 2001;1:323-336. doi:10.1100/tsw.2001.58.
57. Grossman D, Rootenberg M, Perri G-A, et al. Enhancing communication in end-of-life care: a clinical tool translating between the Clinical Frailty Scale and the Palliative Performance Scale. J Am Geriatr Soc. 2014;62(8):1562-1567. doi:10.1111/jgs.12926.
58. Shears M, Takaoka A, Rochwerg B, et al. Assessing frailty in the intensive care unit: A reliability and validity study. J Crit Care. 2018;45:197-203. doi:10.1016/j.jcrc.2018.02.004.
59. Fried LP, Tangen CM, Walston J, et al. Frailty in Older Adults : Evidence for a Phenotype. J Gerontol Med Sci. 2001;56(3):146-157.
60. Bagshaw M, Majumdar SR, Rolfson DB, et al. A prospective multicenter cohort study of frailty in younger critically ill patients. Crit Care. 2016;20(1):175. doi:10.1186/s13054-016-1338-x.
61. Arroll B, Goodyear-Smith F, Crengle S, et al. Validation of PHQ-2 and PHQ-9 to Screen for Major Depression in the Primary Care. Ann Fam Med. 2010;8(4):348-354. doi:10.1370/afm.1139.INTRODUCTION.
62. Üstün TB, Chatterji S, Kostanjsek N, et al. Developing the World Health Organization Disability Assessment Schedule 2.0. Bull World Health Organ. 2010;88(11):815-823.

doi:10.2471/BLT.09.067231.

63. EuroQoL Group. About EQ-5D. http://www.euroqol.org/about-eq-5d.html. Accessed October 13, 2016.
64. Galvin JE, Roe CM, Powlishta KK, et al. The AD8: A brief informant interview to detect dementia. Neurology. 2005;65(4):559-564. doi:10.1212/01.wnl.0000172958.95282.2a.
65. Hlatky MA, Boineau RE, Higginbotham MB, et al. A brief self-administered questionnaire to determine functional capacity (the Duke Activity Status Index). Am J Cardiol. 1989;64(10):651-654. http://www.ncbi.nlm.nih.gov/pubmed/2782256.
66. Goldberg A, Chavis M, Watkins J, Wilson T. The five-times-sit-to-stand test: validity, reliability and detectable change in older females. Aging Clin Exp Res. 2012;24(4):339-344. http://www.ncbi.nlm.nih.gov/pubmed/23238309.
67. Podsiadlo D, Richardson S. The timed "up and go": a test of basic functional mobility for frail eldery persons. *JAGS* 1991; 39: 142-148.
68. Grocott MPW, Browne JP, Van der Meulen J, et al. The Postoperative Morbidity Survey was validated and used to describe morbidity after major surgery. J Clin Epidemiol. 2007;60(9):919-928.
doi:10.1016/j.jclinepi.2006.12.003.
69. Bennett-Guerrero E, Welsby I, Dunn TJ, et al. The use of a postoperative morbidity survey to evaluate patients with prolonged hospitalization after routine, moderate-risk, elective surgery. Anesth Analg. 1999;89(2):514-519. doi:10.1213/00000539-199908000-00050.
70. Davies SJ, Francis J, Dilley J, Wilson RJT, Howell SJ, Allgar V. Measuring outcomes after major abdominal surgery during hospitalization: reliability and validity of the Postoperative Morbidity Survey. Perioper Med (London, England). 2013;2(1):1. doi:10.1186/2047-0525-2-1.
71. Goodman BA, Batterham AM, Kothmann E, et al. Validity of the Postoperative Morbidity Survey after abdominal aortic aneurysm repair-a prospective observational study. Perioper Med (London, England). 2015;4:10. doi:10.1186/s13741-015-0020-1.
72. Wijeysundera DN, Pearse RM, Shulman MA, et al. Assessment of functional capacity before major non-cardiac surgery: an international, prospective cohort study. Lancet (London, England). 2018;391(10140):2631-2640. doi:10.1016/S0140-6736(18)31131-0.
73. Gilhooly DA, Cole M, Moonesinghe SR. The evaluation of risk prediction models in predicting outcomes after bariatric surgery: a prospective observational cohort pilot study. Perioper Med (London, England). 2018;7:6. doi:10.1186/s13741-018-0088-5.
74. McIsaac DI, Taljaard M, Bryson GL, et al. Comparative assessment of two frailty instruments for risk-stratification in elderly surgical patients: study protocol for a prospective cohort study. BMC Anesthesiol. 2016;16(1):111. doi:10.1186/s12871-016-0276-0.
75. Woodfield J, Zacharias M, Wilson G, et al. Protocol, and practical challenges, for a randomised controlled trial comparing the impact of high intensity interval training against standard care before major abdominal surgery: study protocol for a randomised controlled trial. Trials. 2018;19(1):331. doi:10.1186/s13063-018-2701-9.
76. Martos-Benítez FD, Gutiérrez-Noyola A, Echevarría-Víctores A. Postoperative complications and clinical outcomes among patients undergoing thoracic and gastrointestinal cancer surgery: A prospective cohort study. Rev Bras Ter intensiva. 28(1):40-48. doi:10.5935/0103-507X.20160012.
77. Patel ABU, Reyes A, Ackland GL. Non-inferiority of retrospective data collection for assessing perioperative morbidity. PeerJ. 2015;3:e1466. doi:10.7717/peerj.1466.

78. Richards T, Clevenger B, Keidan J, et al. PREVENTT: preoperative intravenous iron to treat anaemia in major surgery: study protocol for a randomised controlled trial. Trials. 2015;16:254. doi:10.1186/s13063-015-0774-2.
79. Kasivisvanathan R, Abbassi-Ghadi N, McLeod ADM, et al. Cardiopulmonary exercise testing for predicting postoperative morbidity in patients undergoing hepatic resection surgery. HPB (Oxford). 2015;17(7):637-643. doi:10.1111/hpb.12420.
80. Moonesinghe SR, Harris S, Mythen MG, et al. Survival after postoperative morbidity: a longitudinal observational cohort study. Br J Anaesth. 2014;113(6):977-984. doi:10.1093/bja/aeu224.
81. Dindo D, Demartines N, Clavien P-A. Classification of surgical complications: a new proposal with evaluation in a cohort of 6336 patients and results of a survey. Ann Surg. 2004;240(2):205-213. doi:10.1097/01.sla.0000133083.54934.ae.
82. Maggiori L, Rullier E, Lefevre JH, et al. Does a Combination of Laparoscopic Approach and Full Fast Track Multimodal Management Decrease Postoperative Morbidity?: A Multicenter Randomized Controlled Trial. Ann Surg. 2017;266(5):729-737. doi:10.1097/SLA.0000000000002394.
83. Palanivelu C, Senthilnathan P, Sabnis SC, et al. Randomized clinical trial of laparoscopic versus open pancreatoduodenectomy for periampullary tumours. Br J Surg. 2017;104(11):1443-1450. doi:10.1002/bjs.10662.
84. Kong S-H, Lee H-J, Na J-R, et al. Effect of perioperative oral nutritional supplementation in malnourished patients who undergo gastrectomy: A prospective randomized trial. Surgery. July 2018. doi:10.1016/j.surg.2018.05.017.
85. Lucot J-P, Cosson M, Bader G, et al. Safety of Vaginal Mesh Surgery Versus Laparoscopic Mesh Sacropexy for Cystocele Repair: Results of the Prosthetic Pelvic Floor Repair Randomized Controlled Trial. Eur Urol. 2018;74(2):167-176. doi:10.1016/j.eururo.2018.01.044.
86. Merki-Künzli C, Kerstan-Huber M, Switalla D, et al. Assessing the Value of Prehabilitation in Patients Undergoing Colorectal Surgery According to the Enhanced Recovery After Surgery (ERAS) Pathway for the Improvement of Postoperative Outcomes: Protocol for a Randomized Controlled Trial. JMIR Res Protoc. 2017;6(10):e199. doi:10.2196/resprot.7972.
87. Schultz JK, Yaqub S, Wallon C, et al. Laparoscopic Lavage vs Primary Resection for Acute Perforated Diverticulitis. JAMA. 2015;314(13):1364. doi:10.1001/jama.2015.12076.
88. Mitropoulos D, Artibani W, Biyani CS, Bjerggaard Jensen J, Rouprêt M, Truss M. Validation of the Clavien-Dindo Grading System in Urology by the European Association of Urology Guidelines Ad Hoc Panel. Eur Urol Focus. March 2017. doi:10.1016/j.euf.2017.02.014.
89. Téoule P, Bartel F, Birgin E, Rückert F, Wilhelm TJ. The Clavien-Dindo Classification in Pancreatic Surgery: A Clinical and Economic Validation. J Invest Surg. January 2018:1-7. doi:10.1080/08941939.2017.1420837.
90. Moonesinghe SR, Grocott MPW, Bennett-Guerrero E, et al. American Society for Enhanced Recovery (ASER) and Perioperative Quality Initiative (POQI) joint consensus statement on measurement to maintain and improve quality of enhanced recovery pathways for elective colorectal surgery. Perioper Med. 2017;6(1):6. doi:10.1186/s13741-017-0062-7.
91. WHO Disability Assessment Schedule 2.0 (WHODAS 2.0). World Health Organization: WHODAS 2.0. http://www.who.int/classifications/icf/whodasii/en/. Published 2014. Accessed April 13, 2014.

92. Shulman MA, Myles PS, Chan MT V., McIlroy DR, Wallace S, Ponsford J. Measurement of Disability-free Survival after Surgery. Anesthesiology. 2015;122(3):524-536. doi:10.1097/ALN.0000000000000586.

93. Wolf AC De, Tate RL, Lannin NA, Middleton J, Lane-brown A, Cameron ID. The World Health Organization Disability Assessment Scale , WHODAS II : reliability and validity in the measurement of activity and participation in a spinal cord injury population. J Rehabil Med. 2012;44(9):747-755. doi:10.2340/16501977-1016.

94. Schlote A, Richter M, Wunderlich MT, et al. WHODAS II with people after stroke and their relatives. Disabil Rehabil. 2009;31(11):855-864. doi:10.1080/09638280802355262.

95. Wolf A, Tate R, Lannin N, Middleton J, Lane-Brown A, Cameron I. The World Health Organization Disability Assessment Scale, WHODAS II: Reliability and validity in the measurement of activity and participation in a spinal cord injury population. J Rehabil Med. 2012;44(9):747-755. doi:10.2340/16501977-1016.

96. Kutlay Ş, Küçükdeveci AA, Elhan AH, Öztuna D, Koç N, Tennant A. Validation of the World Health Organization disability assessment schedule II (WHODAS-II) in patients with osteoarthritis. Rheumatol Int. 2011;31(3):339-346. doi:10.1007/s00296-009-1306-8.

97. Garin O, Ayuso-mateos JL, Almansa J, et al. Validation of the " World Health Organization Disability Assessment Schedule , WHODAS-2 " in patients with chronic diseases. Health Qual Life Outcomes. 2010;8(1):51.

98. Küçükdeveci AA, Kutlay Ş, Yıldızlar D, Öztuna D, Elhan AH, Tennant A. The reliability and validity of the World Health Organization Disability Assessment Schedule (WHODAS-II) in stroke. Disabil Rehabil. 2012;35(May 2012):1-7. doi:10.3109/09638288.2012.690817.

99. Soberg HL, Finset A, Roise O, Bautz-Holter E. The trajectory of physical and mental health from injury to 5 years after multiple trauma: A prospective, longitudinal cohort study. Arch Phys Med Rehabil. 2012;93(5):765-774. doi:10.1016/j.apmr.2011.08.050.

100. Takahashi T, Kumamaru M, Jenkins S, Saitoh M, Morisawa T, Matsuda H. In-patient step count predicts re-hospitalization after cardiac surgery. J Cardiol. 2015;66(4):286-291. doi:10.1016/j.jjcc.2015.01.006.

101. Fisher SR, Graham JE, Ottenbacher KJ, Deer R, Ostir G V. Inpatient Walking Activity to Predict Readmission in Older Adults. Arch Phys Med Rehabil. 2016;97(9 Suppl):S226-31. doi:10.1016/j.apmr.2015.09.029.

102. Low CA, Bovbjerg DH, Ahrendt S, et al. Fitbit step counts during inpatient recovery from cancer surgery as a predictor of readmission. Ann Behav Med. 2018;52(1):88-92. doi:10.1093/abm/kax022.

103. Applebaum E V., Breton D, Feng ZW, et al. Modified 30-second Sit to Stand test predicts falls in a cohort of institutionalized older veterans. Bowen M, ed. PLoS One. 2017;12(5):e0176946. doi:10.1371/journal.pone.0176946.

104. Bansback N, Tsuchiya A, Brazier J, Anis A. Canadian Valuation of EQ-5D Health States : Preliminary Value Set and Considerations for Future Valuation Studies. PLoS One. 2012;7(2). doi:10.1371/journal.pone.0031115.

105. Canadian Institute for Health Information (CIHI). Patient reported outcome measures. https://www.cihi.ca/en/patient-reported-outcome-measures. Accessed June 7, 2018.

106. Wodchis W, Bushmeneva K, Nikitovic M, McKillop I. Guidelines on Person-Level Costing Using Administrative Databases in Ontario. Toronto, ON; 2013. http://www.hsprn.ca/uploads/files/Guidelines_on_PersonLevel_Costing_May_2013.pdf.

107. Juurlink DN, Croxford R, Chong A, Austin P, Tu J, Laupacis A. Canadian Institute for Health Information Discharge Abstract Database : A Validation Study ICES Investigative Report June 2006 Canadian Institute for Health Information Discharge Abstract Database :; 2006.
108. Atkins L, Francis J, Islam R, et al. A guide to using the Theoretical Domains Framework of behaviour change to investigate implementation problems. Implement Sci. 2017;12(1):1-18. doi:10.1186/s13012-017-0605-9.

109. Huijg JM, Gebhardt WA, Crone MR, Dusseldorp E, Presseau J. Discriminant content validity of a theoretical domains framework questionnaire for use in implementation research. Implement Sci. 2014;9(1):1-16. doi:10.1186/1748-5908-9-11.
110. Fergusson D, Aaron SD, Guyatt G, Hébert P. Post-randomisation exclusions: the intention to treat principle and excluding patients from analysis. BMJ. 2002;325(7365):652-654. http://www.ncbi.nlm.nih.gov/pubmed/12242181.

111. Ross S, Grant A, Counsell C, Gillespie W, Russell I, Prescott R. Barriers to participation in randomised controlled trials: a systematic review. J Clin Epidemiol. 1999;52(12):1143-1156. http://www.ncbi.nlm.nih.gov/pubmed/10580777.
112. Kelley GA, Kelley KS. Dropouts and Compliance in Exercise Interventions Targeting Bone Mineral Density in Adults: A Meta-Analysis of Randomized Controlled Trials. J Osteoporos. 2013;2013:1-19. doi:10.1155/2013/250423.
113. McComb A, Warkentin LM, McNeely ML, Khadaroo RG. Development of a reconditioning program for elderly abdominal surgery patients: the Elder-friendly Approaches to the Surgical Environment-BEdside reconditioning for Functional ImprovemenTs (EASE-BE FIT) pilot study. World J Emerg Surg. 2018;13:21. doi:10.1186/s13017-018-0180-7.
114. Zou G. A Modified Poisson Regression Approach to Prospective Studies with Binary Data. Am J Epidemiol. 2004;159(7):702-706. doi:10.1093/aje/kwh090.
115. Austin PC, Laupacis A. A tutorial on methods to estimating clinically and policy-meaningful measures of treatment effects in prospective observational studies: a review. Int J Biostat. 2011;7(1):6. doi:10.2202/1557-4679.1285.
116. Kahan BC, Jairath V, Doré CJ, Morris TP. The risks and rewards of covariate adjustment in randomized trials: an assessment of 12 outcomes from 8 studies. Trials. 2014;15(1):139. doi:10.1186/1745-6215-15-139.

117. Hernández A V, Steyerberg EW, Habbema JDF. Covariate adjustment in randomized controlled trials with dichotomous outcomes increases statistical power and reduces sample size requirements.
J Clin Epidemiol. 2004;57(5):454-460. doi:10.1016/j.jclinepi.2003.09.014.

118. Thompson DD, Lingsma HF, Whiteley WN, Murray GD, Steyerberg EW. Covariate adjustment had similar benefits in small and large randomized controlled trials. J Clin Epidemiol. 2015;68(9):1068-1075. doi:10.1016/j.jclinepi.2014.11.001.

119. Groenwold RHH, Moons KGM, Vandenbroucke JP. Randomized trials with missing outcome data: How to analyze and what to report. Cmaj. 2014;186(15):1153-1157. doi:10.1503/cmaj.131353.

120. Groenwold RHH, Donders ART, Roes KCB, Harrell FE, Moons KGM. Dealing with missing outcome data in randomized trials and observational studies. Am J Epidemiol. 2012;175(3):210-
217. doi:10.1093/aje/kwr302.

121. Vickers AJ, Altman DG. Statistics Notes: Analysing controlled trials with baseline and follow up measurements. BMJ. 2001;323(7321):1123-1124. doi:10.1136/bmj.323.7321.1123.
122. Austin PC, Ghali WA, Tu J V. A comparison of several regression models for analysing cost of CABG surgery. Stat Med. 2003;22:2799-2815. doi:10.1002/sim.1442.
123. Bilimoria KY, Liu Y, Paruch JL, et al. Development and evaluation of the universal ACS NSQIP surgical risk calculator: a decision aid and informed consent tool for patients and surgeons. J Am Coll Surg. 2013;217(5):833-42.e1-3. doi:10.1016/j.jamcollsurg.2013.07.385.

MAIN APPENDIX

Section	Pages
Potential adverse events	35-37
WHODAS scores from cohort study	38
Table from PLOS SR	39
Economic Analysis	40-41
POMS data	42
Study timeline	43

POTENTIAL ADVERSE EVENTS (AE)

There are no expected Adverse Events (or Severe Adverse Events) associated with the prehabilitation program. Surgical complications, extended length of stay, readmission and death are all possible risks of surgery.

We will track (S)AEs during in-person data collection time points (baseline and surgical follow-up visit), and during the time from enrollment to surgery (prehabilitation period).

Below is a list of possible adverse events that could occur during the different stages of The PREPARE Trial (baseline, prehabilitation program, and follow-up visit). If participants report any of the below discomforts or symptoms, or any other (S)AEs during these timepoints, the research study staff will document it in the (S)AE Log, and will notify the Site Lead immediately. If the Site Lead reports that it is an SAE associated with participation in the study, he/she will follow their local REB reporting guidelines.

As death is a possible risk of having surgery, it will not be reported to the OHSN-REB unless the Principal Investigator deems the death related to the prehabilitation program. However, each site will follow-up the protocol of their own REB offices.

Baseline Assessment & Outcome Assessment (at enrollment and post-op visits)

- Physical assessment (TUG – Discharge visit)

Adverse Event	Preventative measures taken	If AE occurs, Action plan:
Fall	- A chair will be placed close by and to be moved by the administrator to allow the participant a break if necessary<br>- The administrator will be watching and ready to assist patient if they need<br>- If the participant is not comfortable doing the test, we will not force them to do so	If a participant falls during the TUG:<br>- Immediately stop the test<br>- Go over, and assess if they are injured<br>- If not, help them to a chair, give them water<br>- Assess severity of situation and call for a clinical professional
Chest pain	- Participants will be excluded if they:<br>- Have severe valvular heart disease that limits a patient's ability to ambulate on level ground, or is associated with synocope or dyspnea<br>- Has severe cardiac dysrhythmias that limit a patient's ability to ambulate on level ground, or is associated with synocope or dyspnea	- Stop test, assess severity of situation, act accordingly and call for a clinical professional

	- Recent myocardial infarcation (within the 6 weeks prior to enrollment) (based on the Heart and Stroke Foundation's Heart walk program)	
Light headed	- Ask questions like, did you drink water today? Have you eaten in the last couple hours? How do you feel about briefly walking today? Would you feel up for it?<br>- Explained that certain symptoms are abnormal and if they start feeling light headed, faint, dizzy to let us know	- Stop test, get participant chair and glass of water, assess severity of situation, act accordingly
Faint	- Explained that certain symptoms are abnormal and if they start feeling light headed, faint, dizzy to let us know<br>- Have a glass of water ready	- Help the participant sit down, get water, assess situation and act accordingly
Dizzy	- Explained that certain symptoms are abnormal and if they start feeling light headed, faint, dizzy to let us know	- Help the participant sit down, get water, assess situation and act accordingly
Severe shortness of breath	- Let the participant know that a little shortness in breath is normal<br>- Ask if participant has asthma, ask if they feel they would be able to complete the TUG test	- If very severe shortness of breath, stop test<br>- Assess severity of situation and act accordingly

During Prehabilitation Program (at home)

- Although unlikely, an adverse event may occur during their home-based trainings (i.e. walking, performing their resistance training with their theraband or during their flexibility training)

Adverse Event	Preventative measures taken
Fall	- Prehabilitation program should reduce the participants risk of falling<br>- Participant urged to go at own pace, if spouse or caretaker in room, exercise program will be explained to both parties, urged to have someone there with them when performing exercises<br>- Participants encouraged to wear proper shoes, exercise on a cushioned mat if possible
Chest pain	- Participant is explained that if mild chest pain occurs, contact study coordinators, if severe, talk with doctor or call 911.
Light headed	- Patient should be encouraged by study staff to make sure they are eating properly and drinking lots of water<br>- Urged to contact study team if unsure about symptoms experiencing

	- Explained that if experiencing any abnormal symptoms, stop exercising, evaluate how they feel, evaluate if it is normal, given number if have any questions, comments or concerns
Faint	- Participant is explained that if feelings of faintness occurs, to stop program, contact study coordinators, if severe, talk with doctor
Dizzy	- Participant is explained that if feelings of dizziness occurs, to stop program, contact study coordinators, if severe, talk with doctor
Severe shortness of breath	- Participant is explained that if shortness of breath occurs, to stop program, contact study coordinators, if severe, talk with doctor

World Health Organization Disability Assessment Schedule 2.0 Domain Severity Scores from a 700-participant Prospective Cohort Study

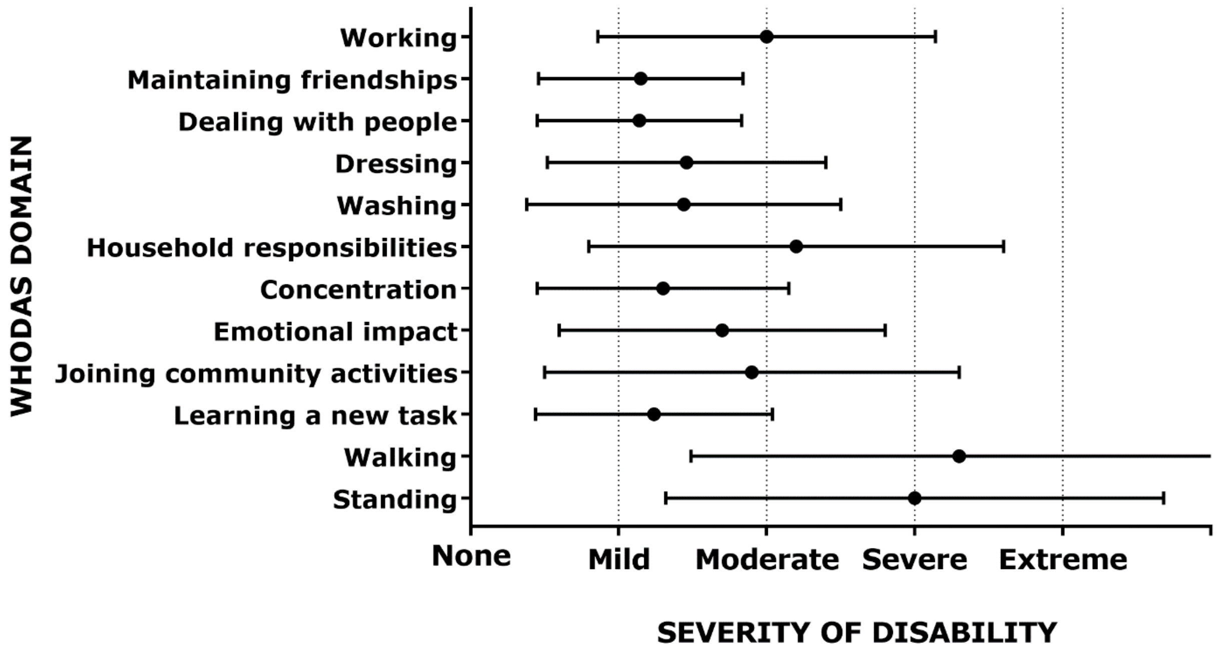

Studies evaluating exercise therapy specifically in older surgical patients with frailty or frailty characteristics

Table 1 - Existing studies of exercise before surgery in older people with frailty or frailty characteristics

Study	N	Design	Centers	Findings
Frailty-specific				
Binder, 2004	100	RCT	1	Improved functional status (Physical Performance Test)
Hoogeboom, 2010	21	RCT	1	Functional status not improved
Opasich, 2010	224	CBA	1	Improved functional status (Timed Up & Go), decreased LoS
Oosting, 2012	30	RCT	1	Improved functional status (6MWT)
Molino-Lova, 2013	99	RCT	1	Improved functional status (Short Physical Performance Battery)
Frailty characteristics				
Gillis, 2014	67	RCT	1	Improved functional status (6MWT); significanty greater improvements in older, sicker patients
Barberan-Garcia, 2018	125	RCT	1	Decreased in-hospital complications

CBA: controlled before-after; RCT: LoS: length of stay; randomized controlled trial; 6MWT: 6-minute walk test

Analytic Approach to Economic Analysis and Outcomes

Research question
The economic research question is whether a structured, home-based, multimodal exercise prehabilitation program is cost-effective compared to usual care from the perspective of Canada's health care system.

Study design
We will perform a cost-utility analysis of a structured, home-based, multimodal exercise prehabilitation program compared to usual care from the perspective of the publicly-funded healthcare system. The time period of the analysis will be restricted to one year. This is believed to be sufficient time to determine the impact of the prehabilitation program on resource use and quality of life. Costs and quality-adjusted life years (QALYs) in the second year will be discounted at a rate of 1.5% per annum as recommended by a national guideline.[1]

We will include all health services that are covered by provincial health insurance plans, including inpatient stays, outpatient attendances, day hospital treatment, medications rehabilitation and physician visits. Data on services used will be measured at baseline (randomization) and then every 6 months until one-year post-randomization. Unit costs for all resources will be obtained from relevant sources (e.g., Ontario Case Costing, Ontario Schedule of Benefits and Ontario Drug Benefit Formulary). We will use a micro-costing technique[2] to estimate the cost of the prehabilitation program, including variable costs (materials, supplies, personnel) and fixed costs (building, facilities, utilities, transportation, equipment). All costs will be expressed in 2018 Canadian Dollars.

We will obtain the efficacy of the planned intervention from the concurrent RCT. Health utility values will be measured using the EQ-5D-5L. Responses to the EQ-5D-5L will be scored using preference weights, which convert the five responses into a single summary index, where a score of 1 reflects perfect health and 0 is equivalent to dead.[3] QALYs will be estimated using a total area under the curve methods.[4]

Analysis
The statistical analysis will be conducted in accordance with current guidelines for clinical and cost-effectiveness analysis alongside RCTs.[5] We will adopt a statistical technique that accounts for the repeated nature of the cost and outcome data. The incremental cost and QALY will be estimated using generalized estimating equations (GEEs), a flexible multivariate regression framework that explicitly allows for the modelling of normal and non-normal distributional forms of repeated measure data.

The incremental cost per QALY gained will be obtained through the difference in average costs of the two strategies divided by the difference in average QALYs as denoted by the coefficient of the therapy indicator variables. As a scenario analysis, we will perform a cost-effectiveness analysis of the prehabilitation program compared to usual care and calculate the incremental cost per one one-unit decrease in WHODAS scale.

As we will have individual-level data on costs and outcomes for the period of trial follow-up, we will evaluate uncertainty of the cost-utility estimates using non-parametric bootstrapping. For this study,

we will obtain 5,000 estimates of costs and QALYs for each strategy. Results from the bootstrapping exercise will also be used to estimate 95% confidence intervals and depict cost effectiveness acceptability curves, which link the probability of the intervention being cost-effective to a range of potential threshold values that the health system may be willing to pay for an additional unit of effect.[6] The economic evaluation will be conducted using STATA version 15.0 and Microsoft Excel with Visual Basic Applications.

Economic Evaluation References

1. Lee K, McCarron C, Bryan S, Coyle D, Krahn M, McCabe C. *Guidelines for the Economic Evaluation of Health Technologies: Canada - 4th Edition*. Toronto, ON; 2017. https://www.cadth.ca/dv/guidelines-economic-evaluation-health-technologies-canada-4th-edition.
2. Gold M, Siegel J, Russell L, Weinstein M. *Cost-Effectiveness in Health and Medicine*. New York, NY: Oxford University Press; 1996.
3. Xie F, Pullenayegum E, Gaebel K, et al. A Time Trade-off-derived Value Set of the EQ-5D-5L for Canada. *Med Care*. 2016;54(1):98-105. doi:10.1097/MLR.0000000000000447.
4. Manca A, Hawkins N, Sculpher MJ. Estimating mean QALYs in trial-based cost-effectiveness analysis: the importance of controlling for baseline utility. *Health Econ*. 2005;14(5):487-496. doi:10.1002/hec.944.
5. Glick H, Doshi J, Sonnad S, Polsky D. *Economic Evaluation in Clinical Trials*. Oxford, UK: Oxford University Press; 2007.
6. Fenwick E, O'Brien BJ, Briggs A. Cost-effectiveness acceptability curves--facts, fallacies and frequently asked questions. *Health Econ*. 2004;13(5):405-415. doi:10.1002/hec.903.

Post-Operative Morbidity Survey Data from 700-Patient Multicenter Cohort Study

Domain	% with complication
Pulmonary	18%
Infectious	9%
Renal	10%
Gastrointestinal	13%
Cardiovascular	12%
Neurological	8%
Hematological	8%
Wound	5%
Pain	14%
**Any complication**	**57%**

Study Timeline: 3 years and 6 months (42 months)

Study Task	Study Month			
	1-5	6-22	23-30	31-42
Study Start-Up<br>(including initial start-up visit)				
Recruitment and data collection across 11 centers (25 months total)				
Monitoring/Recruitment Close-Up Visit				
Complete data collection for secondary outcomes				
Analysis				
Manuscript Preparation				

The study timeline reflects study start-up activities across 14 centers (first 5 months), recruitment based on monthly rates per center (30 patients a month x 25 months=750), a monitoring/recruitment close-up visit with all Site PIs, and the secondary outcome data collection at 365 days post-op. Resources for these study tasks are accounted for in the budget.

SURVEY APPENDIX

Section	Pages
WHODAS: World Health Organization Disability Assessment Schedule 2.O	45
POMS: The Postoperative Morbidity Survey	46-49
CFS: Clinical Frailty Scale	50
EQ-5D-5L	51-52
AD8: Cognitive Screen	53
DASI: Duke Activity Status Index	54
Timed Up and Go (TUG) Test	55
The Katz Index	56
TDF - Participant Program Feedback	57-60
CNST: Canadian Nutrition Screening Tool	61
PHQ-2: Patient Health Questionnaire-2	62
Pre-Operative Questionnaire	63-64
Demographic Questionnaire	65-68

World Health Organization Disability Assessment Schedule (WHODAS) 2.0

In the past 30 days, how much difficulty did you have in:		None	Mild	Moderate	Severe	Extreme or cannot do
S1	Standing for long periods such as 30 minutes?	1	2	3	4	5
S2	Taking care of your household responsibilities?	1	2	3	4	5
S3	Learning a new task, for example, learning how to get to a new place?	1	2	3	4	5
S4	How much of a problem did you have joining in community activities (for example, festivities, religious or other activities) in the same way as anyone else can?	1	2	3	4	5
S5	How much have you been emotionally affected by your health problems?	1	2	3	4	5

In the past 30 days, how much difficulty did you have in:		None	Mild	Moderate	Severe	Extreme or cannot do
S6	Concentrating on doing something for ten minutes?	1	2	3	4	5
S7	Walking a long distance such as a kilometre [or equivalent]?	1	2	3	4	5
S8	Washing your whole body?	1	2	3	4	5
S9	Getting dressed?	1	2	3	4	5
S10	Dealing with people you do not know?	1	2	3	4	5
S11	Maintaining a friendship?	1	2	3	4	5
S12	Your day-to-day work/school?	1	2	3	4	5

H1	Overall, in the past 30 days, how many days were these difficulties present?	***Record number of days ____***
H2	In the past 30 days, for how many days were you totally unable to carry out your usual activities or work because of any health condition?	***Record number of days ____***
H3	In the past 30 days, not counting the days that you were totally unable, for how many days did you cut back or reduce your usual activities or work because of any health condition?	***Record number of days ____***

POMS: The Postoperative Morbidity Survey

**Pulmonary**				
New requirement for oxygen?			☐ YES	☐ NO
If Yes:				
☐ Grade 1<br>No other treatment required (allowed drugs include: antiemetics, antipyretics, analgesics, diuretics and electrolytes)	☐ Grade 2<br>Treatment with drug (other than those listed in grade 1)	☐ Grade 3<br>N/A	☐ Grade 4<br>ICU/CCU/Stepdown	☐ Grade 5<br>Death
Ventilatory support (non-invasive or intubation) required?			☐ YES	☐ NO
If yes:				
☐ Grade 1<br>N/A	☐ Grade 2<br>N/A	☐ Grade 3<br>N/A	☐ Grade 4<br>If ventilatory support was required, automatically grade 4	☐ Grade 5<br>Death
**Infectious**				
New infection present?			☐ YES	☐ NO
If Yes:				
☐ Grade 1<br>N/A	☐ Grade 2<br>New/prolonged antibiotics	☐ Grade 3<br>Patient returned to OR for treatment or had interventional radiology procedure	☐ Grade 4<br>Sepsis/shock/ICU	☐ Grade 5<br>Death
**Gastrointestinal**				
Unable to tolerate an enteral diet?			☐ YES	☐ NO
If Yes:				
☐ Grade 1<br>N/A	☐ Grade 2<br>N/A	☐ Grade 3<br>Patient returned to OR for treatment	☐ Grade 4<br>ICU/CCU/Stepdown	☐ Grade 5<br>Death
Nausea/vomiting/abdominal distension?			☐ YES	☐ NO
If yes:				
☐ Grade 1<br>Antiemetic required	☐ Grade 2<br>N/A	☐ Grade 3<br>Patient returned to OR	☐ Grade 4<br>ICU/CCU/Stepdown	☐ Grade 5<br>Death
**Renal**				
Oliguria (<500cc/24hr)?			☐ YES	☐ NO
If Yes:				

☐ Grade 1<br>No treatment/fluid bolus/new diuretic	☐ Grade 2<br>N/A	☐ Grade 3<br>N/A	☐ Grade 4<br>ICU/CCU/Stepdown/ Dialysis	☐ Grade 5<br>Death
Serum creatinine level >30% of pre-op level?			☐ YES	☐ NO
If yes:				
☐ Grade 1<br>No treatment/fluid bolus/ new diuretic	☐ Grade 2<br>N/A	☐ Grade 3<br>Vasopressor/ Inotrope	☐ Grade 4<br>ICU/OR/Dialysis	☐ Grade 5<br>Death
**Cardiovascular**				
Has the patient experienced hypotension?			☐ YES	☐ NO
If Yes:				
☐ Grade 1<br>Treatment required (fluid bolus >200ml in an hour/held diuretic or anti-hypotensive)	☐ Grade 2<br>N/A	☐ Grade 3<br>Vasopressor	☐ Grade 4<br>ICU/CCU/Stepdown	☐ Grade 5<br>Death
Did the patient experience a Myocardial Ischemia or Infarction?			☐ YES	☐ NO
If yes:				
☐ Grade 1<br>No treatment (EKG only or Troponin only – no clinical symptoms)	☐ Grade 2<br>New statin/B-blocker/ASA/ Heparin	☐ Grade 3<br>Angiography/stent required	☐ Grade 4<br>ICU/CCU/Heart failure/CABG	☐ Grade 5<br>Death
Has the patient experienced an Thrombotic event?			☐ YES	☐ NO
If Yes:				
☐ Grade 1<br>N/A	☐ Grade 2<br>Treatment required	☐ Grade 3<br>N/A	☐ Grade 4<br>ICU/CCU/Stepdown/ Interventional radiology/Surgery	☐ Grade 5<br>Death
Has the patient experienced any Arrhythmias?			☐ YES	☐ NO
If Yes:				
☐ Grade 1<br>No new treatment required	☐ Grade 2<br>New rate/rhythm control	☐ Grade 3<br>Cardioversion	☐ Grade 4<br>CPR/ICU/CCU/ Stepdown	☐ Grade 5<br>Death
Has the patient experienced Cardiogenic Pulmonary Edema?			☐ YES	☐ NO
If Yes:				
☐ Grade 1<br>No new treatment required/Single diuretic dose	☐ Grade 2<br>Multiple diuretics or increased dose	☐ Grade 3<br>N/A	☐ Grade 4<br>ICU/CCU/ Stepdown	☐ Grade 5<br>Death
**Wound**				

Complication present?			☐ YES	☐ NO
If Yes:				
☐ Grade 1<br>Puss/swab/opened at bedside	☐ Grade 2<br>New treatment required	☐ Grade 3<br>OR/Radiological drainage	☐ Grade 4<br>ICU/CCU/Stepdown	☐ Grade 5<br>Death
**Hematological**				
Complication present?			☐ YES	☐ NO
If Yes:				
☐ Grade 1<br>N/A	☐ Grade 2<br>Transfusion (red blood, platelets, frozen plasma, albumin, cryoprecipitate	☐ Grade 3<br>Patient returned to OR for bleeding	☐ Grade 4<br>ICU/CCU/Stepdown	☐ Grade 5<br>Death
**Pain**				
Complication present?			☐ YES	☐ NO
If Yes:				
☐ Grade 1<br>Increase in current pain medication	☐ Grade 2<br>Addition of new medication	☐ Grade 3<br>Patient returned to OR	☐ Grade 4<br>ICU/CCU/Stepdown	☐ Grade 5<br>Death
**Neurological**				
Is there a new neurological deficit?			☐ YES	☐ NO
If Yes:				
☐ Grade 1<br>No treatment required	☐ Grade 2<br>Medical treatment required	☐ Grade 3<br>N/A	☐ Grade 4<br>Formal stroke diagnosis/ICU/ CCU/Stepdown/ Interventional radiology	☐ Grade 5<br>Death
Has the patient experienced any delirium/confusion?			☐ YES	☐ NO
If yes:				
☐ Grade 1<br>No treatment required	☐ Grade 2<br>Medical treatment required	☐ Grade 3<br>N/A	☐ Grade 4<br>ICU/CCU/ Stepdown	☐ Grade 5<br>Death
Sedative induced coma >24 hours after surgery?			☐ YES	☐ NO
If Yes:				
☐ Grade 1<br>N/A	☐ Grade 2<br>N/A	☐ Grade 3<br>N/A	☐ Grade 4<br>Automatically grade 4	☐ Grade 5<br>Death
Non-sedative induced coma?			☐ YES	☐ NO
If Yes:				

☐ Grade 1<br>N/A	☐ Grade 2<br>Automatically grade 2	☐ Grade 3<br>N/A	☐ Grade 4<br>ICU/CCU/<br>Stepdown	☐ Grade 5<br>Death

Clinical Frailty Scale

Clinical Frailty Scale*

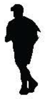

1 **Very Fit** – People who are robust, active, energetic and motivated. These people commonly exercise regularly. They are among the fittest for their age.

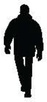

2 **Well** – People who have **no active disease symptoms** but are less fit than category 1. Often, they exercise or are very **active occasionally**, e.g. seasonally.

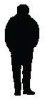

3 **Managing Well** – People whose **medical problems are well controlled,** but are **not regularly active** beyond routine walking.

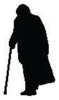

4 **Vulnerable** – While **not dependent** on others for daily help, often **symptoms limit activities.** A common complaint is being "slowed up", and/or being tired during the day.

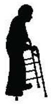

5 **Mildly Frail** – These people often have **more evident slowing,** and need help in **high order IADLs** (finances, transportation, heavy housework, medications). Typically, mild frailty progressively impairs shopping and walking outside alone, meal preparation and housework.

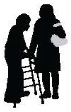

6 **Moderately Frail** – People need help with **all outside activities** and with **keeping house**. Inside, they often have problems with stairs and need **help with bathing** and might need minimal assistance (cuing, standby) with dressing.

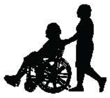

7 **Severely Frail** – **Completely dependent for personal care**, from whatever cause (physical or cognitive). Even so, they seem stable and not at high risk of dying (within ~ 6 months).

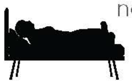

8 **Very Severely Frail** – Completely dependent, approaching the end of life. Typically, they could not recover even from a minor illness.

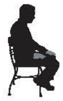

9. **Terminally Ill** - Approaching the end of life. This category applies to people with **a life expectancy <6 months**, who are **not otherwise evidently frail.**

Scoring frailty in people with dementia

The degree of frailty corresponds to the degree of dementia. Common **symptoms in mild dementia** include forgetting the details of a recent event, though still remembering the event itself, repeating the same question/story and social withdrawal.

In **moderate dementia**, recent memory is very impaired, even though they seemingly can remember their past life events well. They can do personal care with prompting.

In **severe dementia**, they cannot do personal care without help.

* 1. Canadian Study on Health & Aging, Revised 2008.
2. K. Rockwood et al. A global clinical measure of fitness and frailty in elderly people. CMAJ 2005;173:489-495.

© 2007-2009. Version 1.2. All rights reserved. Geriatric Medicine Research, Dalhousie University, Halifax, Canada. Permission granted to copy for research and educational purposes only.

DALHOUSIE UNIVERSITY Inspiring Minds

EQ-5D-5L

Under each heading, please tick the ONE box that best describes your health TODAY.

MOBILITY

I have no problems in walking about	❑
I have slight problems in walking about	❑
I have moderate problems in walking about	❑
I have severe problems in walking about	❑
I am unable to walk about	❑

SELF-CARE

I have no problems washing or dressing myself	❑
I have slight problems washing or dressing myself	❑
I have moderate problems washing or dressing myself	❑
I have severe problems washing or dressing myself	❑
I am unable to wash or dress myself	❑

USUAL ACTIVITIES *(e.g. work, study, housework, family or leisure activities)*

I have no problems doing my usual activities	❑
I have slight problems doing my usual activities	❑
I have moderate problems doing my usual activities	❑
I have severe problems doing my usual activities	❑
I am unable to do my usual activities	❑

PAIN / DISCOMFORT

I have no pain or discomfort	❑
I have slight pain or discomfort	❑
I have moderate pain or discomfort	❑
I have severe pain or discomfort	❑
I have extreme pain or discomfort	❑

ANXIETY / DEPRESSION

I am not anxious or depressed	❑
I am slightly anxious or depressed	❑
I am moderately anxious or depressed	❑
I am severely anxious or depressed	❑
I am extremely anxious or depressed	❑

Sample

- We would like to know how good or bad your health is TODAY.
- This scale is numbered from 0 to 100.
- 100 means the <u>best</u> health you can imagine.
  0 means the <u>worst</u> health you can imagine.
- Mark an X on the scale to indicate how your health is TODAY.
- Now, please write the number you marked on the scale in the box below.

YOUR HEALTH TODAY = ☐

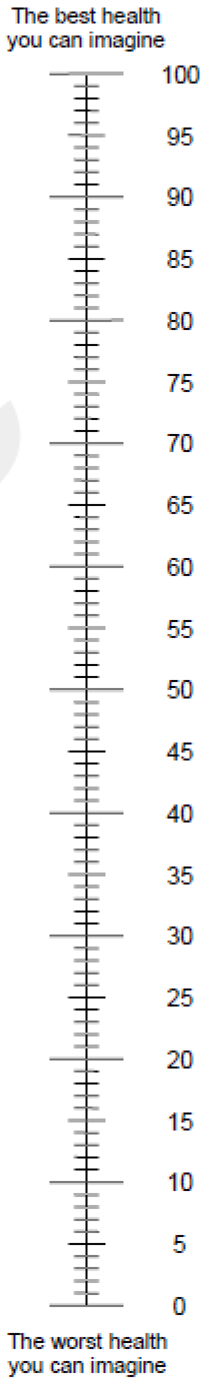

Sample

AD8: Cognitive Screen

The AD8: The Washington University Dementia Screening Test

("Eight-item Interview to Differentiate Aging and Dementia")

Administration

The questions are given to the respondent on a clipboard for self–administration or can be read aloud to the respondent either in person or over the phone. It is preferable to administer the AD8 to an informant, if available. If an informant is not available, the AD8 may be administered to the patient.

When administered to an informant, specifically ask the respondent to rate change in the patient.

When administered to the patient, specifically ask the patient to rate changes in his/her ability for each of the items, ***without*** attributing causality.

If read aloud to the respondent, it is important for the clinician to carefully read the phrase as worded and give emphasis to note changes due to cognitive problems (not physical problems).
There should be a one second delay between individual items.

No timeframe for change is required.

Scoring

The final score is a sum of the number items marked "Yes, A change".

Interpretation of Results

0-1: Normal cognition:
2 or greater: Impairment in cognition

Remember, "Yes, a change" indicates that there has been a change in the last several years caused by cognitive (thinking and memory) problems.	**YES, A change**	**NO, No change**	**N/A, Don't know**
**1.** Problems with judgment (e.g., problems making decisions, bad financial decisions, problems with thinking)			
**2.** Less interest in hobbies/activities			
**3.** Repeats the same things over and over (questions, stories, or statements)			
**4.** Trouble learning how to use a tool, appliance, or gadget (e.g., computer, micro-wave, remote control)			
**5.** Forgets correct month or year			
**6.** Trouble handling complicated financial affairs (e.g., balancing checkbook, income taxes, paying bills)			
**7.** Trouble remembering appointments			
**8.** Daily problems with thinking and/or memory			

**TOTAL AD8 SCORE**	

DASI: Duke Activity Status Index

DUKE ACTIVITY STATUS INDEX
Score sheet

Can you:	Scores are only given for 'yes' replies	
1. Take care of yourself, that is, eat, dress, bathe or use the toilet?	2.75	Yes/No
2. Walk indoors, such as around your house?	1.75	Yes/No
3. Walk a block or two on level ground?	2.75	Yes/No
4. Climb a flight of stairs or walk up a hill?	5.50	Yes/No
5. Run a short distance?	8.00	Yes/No
6. Do light work around the house like dusting or washing dishes?	2.70	Yes/No
7. Do moderate work around the house like vacuuming, sweeping floors or carrying groceries?	3.50	Yes/No
8. Do heavy work around the house like scrubbing floors or lifting or moving heavy furniture?	8.00	Yes/No
9. Do garden work like raking leaves, weeding or pushing a lawn mower?	4.50	Yes/No
10. Have sexual relations?	5.25	Yes/No
11. Participate in moderate recreational activities like golf, bowling, dancing, doubles tennis or throwing a ball?	6.00	Yes/No
12. Participate in strenuous sports like swimming, singles tennis, football, basketball or skiing?	7.50	Yes/No

(To be completed by staff) Duke Activity Status Index (DASI) = ☐

The higher the score is, the more physically active a person is according to this set of activities of daily living. The DASI score should be completed every three months and the score entered into the back of the exercise diary to monitor progress.

Timed Up and Go (TUG) Test

Instructions:

- Patients wear regular foot wear and can use a walking aid, if needed.
- Begin by having the patient sit with their hips all the way back (back touching the back of the chair) in a standard arm chair and identify a line 3 meters, or 10 feet away, on the floor.
- Instruct the patient: when I say "Go," I want you to stand up from the chair, walk to the marked line on the floor at your normal pace, turn around, walk back to the chair at your normal pace, and sit back down in the chair. You are allowed to use the arm rests during the sit to stand and stand to sit movements, if needed.
- Start timing on the word "Go." Stop timing after the patient sits back down, with their back resting on the chair.

1. **TUG time: ______ seconds**
2. **Walking aid used: ☐ No or ☐ Yes**
   If yes, what type of aid (walker, cane, etc.): ____________
3. **If participant did not attempt test or failed the test, indicate why:**

   Participant tried but unable

   Participant could not stand unassisted

   Test not attempted because research staff felt unsafe

   Test not attempted because participant felt unsafe

   Participant unable to understand instructions

   Participant refused

   Other (specify) __________________

The Katz Index

Katz Index of Independence in Activities of Daily Living

ACTIVITIES<br>POINTS (1 OR 0)	INDEPENDENCE:<br>(1 POINT)<br>**NO** supervision, direction or personal assistance	DEPENDENCE:<br>(0 POINTS)<br>**WITH** supervision, direction, personal assistance or total care
**BATHING**<br><br>POINTS:___________	**(1 POINT)** Bathes self completely or needs help in bathing only a single part of the body such as the back, genital area or disabled extremity.	**(0 POINTS)** Needs help with bathing more than one part of the body, getting in or out of the tub or shower. Requires total bathing.
**DRESSING**<br><br>POINTS:___________	**(1 POINT)** Gets clothes from closets and drawers and puts on clothes and outer garments complete with fasteners. May have help tying shoes.	**(0 POINTS)** Needs help with dressing self or needs to be completely dressed.
**TOILETING**<br><br>POINTS:___________	**(1 POINT)** Goes to toilet, gets on and off, arranges clothes, cleans genital area without help.	**(0 POINTS)** Needs help transferring to the toilet, cleaning self or uses bedpan or commode.
**TRANSFERRING**<br><br>POINTS:___________	**(1 POINT)** Moves in and out of bed or chair unassisted. Mechanical transferring aides are acceptable.	**(0 POINTS)** Needs help in moving from bed to chair or requires a complete transfer.
**CONTINENCE**<br><br>POINTS:___________	**(1 POINT)** Exercises complete self control over urination and defecation.	**(0 POINTS)** Is partially or totally incontinent of bowel or bladder.
**FEEDING**<br><br>POINTS:___________	**(1 POINT)** Gets food from plate into mouth without help. Preparation of food may be done by another person.	**(0 POINTS)** Needs partial or total help with feeding or requires parenteral feeding.

TOTAL POINTS = ______ 6 = High (*patient independent*) 0 = Low (*patient very dependent*)

Participant Program Feedback: Theoretical Domains Framework (TDF) Questionnaire

Knowledge:

○ Strongly Disagree	○ Disagree	○ Neutral	○ Agree	○ Strongly Agree

1-Strongly disagree 2-Disagree 3-Unsure 4-Agree 5-Strongly agree

1. I am aware of the content and goals of the exercise program: _______
2. I know how to complete the at-home exercise program: _______

Skills:

○ Strongly Disagree	○ Disagree	○ Neutral	○ Agree	○ Strongly Agree

1-Strongly disagree 2-Disagree 3-Unsure 4-Agree 5-Strongly agree

3. I have been trained how to do the exercises in the exercise program: _______
4. I have the skills and ability to perform the exercises in the program: _______
5. I have practiced the exercises in the program: _______

Belief about capabilities:

○ Strongly Disagree	○ Disagree	○ Neutral	○ Agree	○ Strongly Agree

1-Strongly disagree 2-Disagree 3-Unsure 4-Agree 5-Strongly agree

6. I am confident that I can do the exercises in the program: _______
7. I feel that I have control over the exercises in the program: _______
8. For me, doing the exercises in the program is easy: _______

Optimism:

○<br><br>Strongly Disagree	○<br><br>Disagree	○<br><br>Neutral	○<br><br>Agree	○<br><br>Strongly Agree

1-Strongly disagree 2-Disagree 3-Unsure 4-Agree 5-Strongly agree

9. With regard to the exercise program, I usually expect the best: ________

10. With regard to the exercise program, I hardly ever expect things to go my way: ________

Belief about consequences:

○<br><br>Strongly Disagree	○<br><br>Disagree	○<br><br>Neutral	○<br><br>Agree	○<br><br>Strongly Agree

1-Strongly disagree 2-Disagree 3-Unsure 4-Agree 5-Strongly agree

11. For me, completing the exercise program is useful: ________

12. If I complete the exercise program it will benefit **my** health: ________

Reinforcement:

13. Whenever I do the exercise program, I get acknowledgement from people who are important to me. ________

14. If I do the exercise program, I feel like I am doing something important:________

Goals:

15. Generally, other things in my life were a higher priority than completing the exercise program: ________

Memory, attention and decisional processes:

○<br><br>Strongly Disagree	○<br><br>Disagree	○<br><br>Neutral	○<br><br>Agree	○<br><br>Strongly Agree

1-Strongly disagree 2-Disagree 3-Unsure 4-Agree 5-Strongly agree

16. Doing the exercises in the program was easy to remember: _______

17. When I concentrate on doing the exercises in the program, I have no trouble focusing my attention. _______

Environmental context and resources:

○<br><br>Strongly Disagree	○<br><br>Disagree	○<br><br>Neutral	○<br><br>Agree	○<br><br>Strongly Agree

1-Strongly disagree 2-Disagree 3-Unsure 4-Agree 5-Strongly agree

18. I have the tools and support needed to complete the exercise program: _______

19. The exercise program has enough resources to help me to do the exercises: _______

Social influences:

○<br><br>Strongly Disagree	○<br><br>Disagree	○<br><br>Neutral	○<br><br>Agree	○<br><br>Strongly Agree

1-Strongly disagree 2-Disagree 3-Unsure 4-Agree 5-Strongly agree

20. Most people who are important to me think that I should do the exercise program: _______

21. Most people whose opinion I care about would approve me of doing the exercise program: _______

Emotion:

○<br><br>Never	○<br><br>Rarely	○<br><br>Sometimes	○<br><br>Very Often	○<br><br>Always

22. To what extent do you generally feel inspired to do the exercise program?: _______

23. To what extent do you generally feel nervous about the exercise program?: _______

Behavioural regulation:

○	○	○	○	○
Strongly Disagree	Disagree	Neutral	Agree	Strongly Agree

1-Strongly disagree 2-Disagree 3-Unsure 4-Agree 5-Strongly agree

24. I used my calendar to keep track of my exercise program: ________

25. I tend to notice my successes while working towards completing the exercise program. ________

Open-ended questions on patient experience:

26. What made it easy for you to do the exercise program?

27. What made it hard for you to do the exercise program?

28. Is there anything else you would like to tell us about the exercise program?

CANADIAN NUTRITION SCREENING TOOL (CNST)

Name:	Age:	Weight:	Room:

Identify patients who are at risk for malnutrition

	Date:		Date:	
	Admission		Rescreening	
Ask the patient the following questions*	Yes	No	Yes	No
Have you lost weight in the past 6 months **WITHOUT TRYING** to lose this weight?<br>If the patient reports a weight loss but gained it back, consider it as NO weight loss.				
Have you been eating less than usual **FOR MORE THAN A WEEK**?				
**Two "YES" answers indicate nutrition risk†**				

* If the patient is unable to answer the questions, a knowledgeable informant can be used to obtain the information. If the patient is uncertain regarding weight loss, ask if clothing is now fitting more loosely.

Patients at nutrition risk need an assessment to confirm malnutrition

Nutrition screening using a valid tool can generate a significant volume of requests for nutrition evaluation. Subjective Global Assessment (SGA) is a simple and efficient first-line assessment of nutritional status that can be used following a positive screening and to help prioritize cases.

If a patient is malnourished (SGA B or C), an in-depth nutrition assessment, along with treatment, is required by a registered dietitian.

The Canadian Nutrition Screening Tool was rigorously validated and tested for reliability in Canadian hospitals. Non-expert raters completed the tool and it was compared to the SGA conducted by a dietitian or trained nutrition researcher.

† If a patient is not at risk, rescreen within a week. Only consider weight change in the past week.

Validation and reliability testing of the Canadian Nutrition Screening Tool was funded by an unrestricted educational grant of Abbott Nutrition Canada.

Canadian Nutrition Society
Société canadienne de nutrition

Canadian Malnutrition Task Force | le Groupe de travail canadien sur la malnutrition

PHQ-2: Patient Health Questionnaire-2

Over the past 2 weeks how often have you been bothered by any of the following problems?	***Not at all***	***Several days***	***More than half of days***	***Nearly every day***
Have you been feeling down, depressed or hopeless?	☐	☐	☐	☐
Have you had little interest or pleasure in doing things?	☐	☐	☐	☐

Pre-Operative Questionnaire

Intervention Group:
While any falls in the intervention group will have already been recorded during weekly adherence calls, kindly ask this question again to ensure that this information has not been missed. It is suggested that you explain to the patient that while you are aware you have already discussed falls during the adherence calls, you are simply ensuring you have not missed any information.

Control Group:
For sites outside of Ottawa, please ensure a proper introduction is given to the participant as this will be the first time they will have been contacted by research from The Ottawa Hospital coordinating site.

1. Have you experienced a fall leading up to surgery, specifically, since you enrolled in The PREPARE Trial?

   ☐ No
   ☐ Yes

   How many times? __________
   If yes, details of the fall:
   ____
   ____
   ____
   ____

2. Thinking back to about a month before you enrolled in The PREPARE Trial, how much more would you say you exercised in the past 4 weeks compared to the month before enrollment?

○ No change at all	○ Slightly more	○ Moderately more	○ Significantly more	○ Biggest change possible

**For intervention group only*

3. Step count since previous (final) adherence call:____________

Demographic Information

This information is to be entered directly into the password protected Electronic Data Capturing (EDC) System stored on the secured TOH network. Note that this information will not be stored on the iPad, it is simply entered into the secured EDC system which houses data on the TOH secured network.

RA Initials |_____|_____| Date: _____________

Patient name	
Study ID	
Sex	
Age	
Phone #1	
Phone #2 / Email	
Clinical Frailty Score	
Surgery Type	
Surgery date	
Group Allocation	

Demographic Information Continued

Question	Responses		
	Yes	*No*	*Don't know*
Do you have people at home or nearby that you can count on for social/emotional support when you need it?			
Do you have any physical limitations that would prevent you from doing exercise?			
Are you currently consuming any oral nutrition supplements (i.e. protein/energy drinks, shakes) or vitamin/mineral supplements?			

Do you have or have you experienced the following medical conditions?	*Yes*	*No*	*Don't know/ prefer not to say*
Myocardial infarction (heart attack)			

Congestive heart failure			
Peripheral vascular disease			
CVA or TIA (stroke or mini-stroke)			
Connective tissue disease			
Peptic ulcer disease			
Chronic lung disease (ex. Chronic Obstructive Pulmonary Disease - COPD)			
Complications from your diabetes (eye, nerve, foot, kidney or other)			
Do you take insulin?			
Liver disease			
Hemiplegia (paralysis of one side of the body)			
Rheumatologic disease (i.e. Rheumatoid arthritis, Lupus or psoriasis)			
Kidney disease (of any kind)			

Cancer			
Cancer with metastases (spread to other areas of the body)			
Obstructive sleep apnea			
Do you use a continuous positive airway pressure (CPAP) machine to sleep?			
HIV or AIDS			
Visual (seeing) problems or glasses/contacts needed			
Hearing problems (corrected or uncorrected)			
Have you received radiation therapy in the past 6 months?	*Yes ☐ No ☐*	*Start date:*	*End date:*
Have you received chemotherapy in the past 6 months?	*Yes ☐ No ☐*	*Start date:*	*End date:*
Do you smoke or did you in the past?	*Yes, I currently smoke ☐*<br>*No, I do not smoke, but I did in the past ☐*<br>*No, I have never smoked ☐*		
If you smoke(d), how many cigarettes do you smoke in a day?	*1-10 ☐*	*11-20 ☐*	*More than 20 ☐*

THE PREPARE TRIAL

PREPARE Trial: a parallel arm multicenter randomized trial of frailty-focused PReoperative Exercise to decrease PostoperAtive complication Rates and disability scorEs

CLINICAL RESEARCH PROTOCOL

v.1.7

28-Aug-2023

**Sponsor Investigator/Principal Investigator:**	Daniel McIsaac, MD, MPH, FRCPC<br>Department of Anesthesiology and Pain Medicine<br>The Ottawa Hospital<br>1053 Carling Avenue<br>Ottawa ON K1Y 4E9
**Co-Principal Investigators:**	Dean Fergusson, PhD, MHA<br>Rachel Khadaroo, MD, PhD, FRCSC, FACS<br>John Muscedere, MD, FRCPC<br>Monica Taljaard, PhD
**Site Investigators:**	Husein Moloo, MD, MSc, FRCSC<br>Sylvain Gagne, MD, FRCPC<br>Rodney Breau, MD, MSc, FRCSC<br>Daniel Trottier, MD, FRCSC<br>Jean Michel Aubin, MD, FRCSC<br>Grace Ma, MD, FRCSC<br>Tarit Saha, MD, FRCPC<br>Antoine Eskander, MD, ScM, FRCSC<br>Rachel Khadaroo, MD, PhD, FRCSC, FACS<br>Ilun Yang, MD, FRCSC<br>Pablo E. Serrano, MD, MPH, MSc<br>Thomas Mutter, MD, FRCPC<br>Elijah Dixon, MD, BSc, MSc(Epi), FRCSC, FACS<br>Gregg Nelson, MD, PhD, FRCSC<br>Rosaleen Chun, MD, FRCPC<br>Susan Lee, MD, FRCPC, MAS<br>Sadeesh Srinathan, MD, MSc, FRCS(C)
**Funders:**	Canadian Institutes of Health Research (CIHR)<br>The Ottawa Hospital Academic Medical Organization (TOHAMO)

**Coordinating Centre:**	Department of Anesthesiology and Pain Medicine<br>The Ottawa Hospital<br>1053 Carling Avenue<br>Ottawa, ON K1Y 4E9

DOCUMENT HISTORY

Version	Version Date	Changes
1.7	28-AUGUST-2023	- Sample size has been increased to 850 participants.
1.6	30-JANUARY-2023	- Language has been added to the adverse events appendix to clarify that symptoms related to participants' pre-existing health conditions (i.e., comorbidities, side effects of cancer treatment, etc.) are expected for this population. The experience of such comorbidities and side effects are not considered to be a result of their participation in the prehabilitation program or trial overall.
1.5	26-APRIL-2022	- The processes for obtaining written informed consent and verbal informed consent have been clarified. Study procedures may begin as soon as a participant provides their written consent or their verbal consent to participate. When obtaining written consent, a photocopy of the fully signed Main Informed Consent Form will be provided to the participant for their records. When obtaining verbal consent, a copy of the Main Information Sheet (no signature pages) will be provided to the participant for their records by email or by mail.
1.4	11-NOVEMBER-2021	- For patients scheduled for surgery outside of the recruiting institution, only patient-reported data will be collected for the in-hospital follow-up (posteroperative days 3, 5, and 7 and discharge). Data collected by chart review will be documented as missing.
1.3	01-JULY-2021	- Addition of site investigators (Drs. Serrano, Nelson and Srinathan)<br>- Study objectives and inclusion/exclusion criteria clarified throughout

		- Addition of new cardio videos (standing and seated options) for intervention participants<br>- All intervention participants to receive nutrition pamphlet<br>- Exercise and adherence support provided by coordinating centre clarified to better reflect PREPARE Program approach<br>- COVID-19 status to be collected at all timepoints<br>- Study AE definitions clarified<br>- Program adherence and planned analysis defined<br>- Data collection and analysis plan for "Day 84" participants defined<br>- Follow-up windows included for all data collection timepoints<br>- Additional appendices (questionnaires, data collection tools, etc.) removed from document
1.2	02-SEPTEMBER-2020	- Addition of remote recruitment as needed<br>- Changes to data collection due to remote visits as needed
1.1	03-DECEMBER-2019	- Change in data collection time points for the Post-Operative Morbidity Survey (POMS)<br>- Time limit was placed on in-hospital data collection
1.0	26-NOVEMBER-2019	- New document

PROTOCOL SYNOPSYS

**Study Title:**	PREPARE Trial: a parallel arm multicenter randomized trial of frailty-focused PReoperative Exercise to decrease PostoperAtive complication Rates and disability scorEs
**Protocol Short Title:**	The PREPARE Trial
**Protocol Version:**	1.7
**Protocol Date:**	28-AUGUST-2023
**Clinical Trials Registration Number:**	NCT04221295
**Sponsor-Investigator:**	**Daniel McIsaac, MD, MPH, FRCPC**

	Department of Anesthesiology and Pain Medicine<br>The Ottawa Hospital<br>1053 Carling Avenue<br>Ottawa ON K1Y 4E9
**Site Investigators:**	**Husein Moloo, MD, MSc, FRCSC**<br>The Ottawa Colorectal Group<br>The Ottawa Hospital<br>1053 Carling Avenue<br>Ottawa, ON K1Y 4E9<br><br>**Sylvain Gagne, MD, FRCPC**<br>Department of Anesthesiology and Pain Medicine<br>The Ottawa Hospital<br>501 Smyth Road,<br>Ottawa, ON K1H 8L6<br><br>**Rodney Breau, MD, MSc, FRCSC**<br>Department of Surgery, Urology<br>The Ottawa Hospital<br>501 Smyth Road,<br>Ottawa, ON K1H 8L6<br><br>**Daniel Trottier, MD, FRCSC**<br>Department of Surgery<br>Montfort Hospital<br>713 Montreal Road<br>Ottawa, ON K1K 0T2<br><br>**Jean Michel Aubin, MD, FRCSC**<br>Department of Surgery<br>Queensway Carleton Hospital<br>3045 Baseline Road<br>Ottawa, ON K2H 8P4<br><br>**Grace Ma, MD, FRCSC**<br>Department of General Surgery<br>Health Sciences North<br>65 Larch Street<br>Sudbury, ON P3E 1B8<br><br>**Tarit Saha, MD, FRCPC**<br>Department of Anesthesia and Perioperative Medicine<br>Kingston Health Sciences Centre<br>76 Stuart Street<br>Kingston, ON K7L 2V7<br><br>**Antoine Eskander, MD, ScM, FRCSC**<br>Department of Otolaryngology<br>Sunnybrook Health Sciences Centre

	2075 Bayview Drive<br>Toronto, ON M4N 3M5<br><br>**Rachel Khadaroo, MD, PhD, FRCSC, FACS**<br>Department of Surgery and Critical Care Medicine<br>University of Alberta Hospital<br>84409112th Street NW<br>Edmonton, AB T6G 2B7<br><br>**Ilun Yang, MD, FRCSC**<br>Department of Surgery<br>Juravinski Hospital<br>711 Concession Street<br>Hamilton, ON L8V 1C3<br><br>**Pablo E. Serrano, MD, MPH, MSc**<br>Department of Surgery<br>Juravinski Hospital<br>711 Concession St., Rm B3-161<br>Hamilton, ON L8V 1C3<br><br>**Thomas Mutter, MD, FRCPC**<br>Department of Anesthesiology, Perioperative and Pain Medicine<br>Winnipeg Regional Health Authority<br>671 William Avenue<br>Winnipeg, MB R3E 0Z2<br><br>**Sadeesh Srinathan, MD, MSc, FRCS(C)**<br>Department of Surgery<br>GE611 - 820 Sherbrook Street<br>Health Sciences Centre<br>Winnipeg MB R3A 1R9<br><br>**Elijah Dixon, MD, BSc, MSc(Epi), FRCSC, FACS**<br>Department of Surgery<br>Alberta Health Services<br>1403-29th Street NW<br>Calgary, AB T2N 2T9<br><br>**Gregg Nelson, MD, PhD, FRCSC**<br>Department of Obstetrics and Gynaecology<br>University of Calgary<br>2500 University Drive NW<br>Calgary Alberta T2N 1N4<br><br>**Rosaleen Chun, MD, FRCPC**<br>Department of Anesthesiology, Perioperative and Pain Medicine<br>University of Calgaryx<br>Foothills Medical Center Division<br>1403-29th Street NW, Calgary, Alberta T2N 2T9

	**Susan Lee, MD, FRCPC, MAS**<br>Anesthesia Office<br>Royal Columbian Hospital<br>330 E Columbia St.<br>New Westminster, BC V3W 3L7
**Coordinating Centre:**	Department of Anesthesiology and Pain Medicine<br>The Ottawa Hospital<br>1053 Carling Avenue<br>Ottawa ON, K1Y 4E9
**Funders:**	Canadian Institutes of Health Research (CIHR)<br>The Ottawa Hospital Medical Academic Organization (TOHAMO)
**Study Objectives:**	1. Improve patient-oriented disability scores (co-primary outcome)<br>2. Reduce complication rates (co-primary outcome), improve quality of life<br>3. Decrease healthcare resource use in older people with frailty having elective inpatient surgery
**Study Design:**	Assessor blinded multicenter individual patient parallel-arm randomized controlled trial conducted in 14 Canadian academic or community hospitals
**Number of Participants:**	850
**Study Population:**	Older adults aged ≥60 years who are undergoing major elective inpatient non-cardiac surgery
**Inclusion Criteria:**	1. Patients aged ≥60 years<br>2. Scheduled, or on the pathway, for elective surgery eLOS ≥2 days<br>3. Expected surgery date between 3 and 12 weeks from enrollment<br>4. Clinical Frailty Scale (CFS) score ≥4/9
**Exclusion Criteria:**	1. Inability to speak English or French<br>2. Co-morbidity preventing assessment or understanding of questionnaires<br>3. Unable to be contacted by telephone<br>4. Unwilling to participate in exercise program<br>5. Cardiac, neurological or orthopedic procedure<br>6. Palliative surgery<br>7. Certain cardiovascular conditions (see Section 4.6)
**Follow-up duration:**	One year

Table of Contents

1. BACKGROUND AND RATIONALE 11

1.1. The majority of Canadians who have surgery are older and many live with frailty 11

1.2. Frailty makes people vulnerable to adverse health outcomes 11

1.3. Findings from our group provide important insights into why frailty leads to adverse outcomes 11

1.4. Interventions to improve postoperative outcomes for older people with frailty are infrequently studied, however, exercise prehabilitation is a promising therapy 12

1.5. Care of older people having surgery and exercise before surgery are high-priority areas for patient-oriented research 12

1.6. Older people with frailty require tailored (i.e. type, location, duration) exercise prehabilitation programs to meet their unique needs 12

1.7. Results from our pilot trial of home-based exercise prehabilitation in cancer patients with frailty demonstrate feasibility 13

1.8. Serious limitations prevent generalizing the exercise prehabilitation literature for people *without* frailty to people *with* frailty 13

1.9. Currently registered studies will not address pertinent knowledge gaps 14

2. RESEARCH QUESTIONS 14

3. STUDY OBJECTIVES 14

4. STUDY DESIGN 14

4.2.1. Control Group 16

4.3. Allocation and Randomization 16

4.4. Protection Against Bias 16

4.5. Duration of Treatment 16

4.6. Inclusion/Exclusion Criteria 16

4.7. Justification of Criteria 17

4.8. Frequency and Duration to Follow-Up 17

4.9. Primary Outcomes 18

4.9.1. In-hospital complications 18

4.9.2. Patient-reported disability 30 days after surgery 19

4.10. Secondary Outcomes 19

4.10.1. Function 19

4.10.2. Health-related quality of life 19

4.10.3. All-cause mortality 19
4.10.4. Health System 20
4.10.5. Participant Feedback 20
4.10.6. Safety 20
4.11. Outcome Measurement at Follow-Up 20
4.12. Sample Size 20
4.13. Health Services Research Issues 21
4.14. Recruitment 21
4.15. Adherence 21
4.16. Lost to Follow-Up 22
4.17. Study Centres 22
5. DATA ANALYSIS 23
5.1. Primary Outcomes 23
5.2. Secondary Outcomes 23
5.3. Additional Analyses 23
5.4. Planned Subgroup Analyses 24
6. STUDY PROCEDURES 24
6.1. Informed Consent 24
6.2. Surgical Consultation Baseline Visit (expected between 3 and 12 weeks from date of surgery) 24
6.3. Weekly Adherence Calls 25
6.4. Day Before Surgery 25
6.5. Day of Surgery 25
6.6. In-Hospital Follow-Up 25
6.7. Post-Discharge Follow-Up 26
6.7.1. 30-Day Phone Follow-Up 26
6.7.2. 90-Day Phone Follow-up 27
6.7.3. One-Year Phone Follow-Up 27
6.8. Study Schedule of Events 27
7. DATA COLLECTION MANAGEMENT 28
7.1. Source Documents 28
7.2. Case Report Forms 29

7.3. Protocol Deviations 29
7.4. Record Retention 29
8. MANAGEMENT AND REPORTING OF ADVERSE EVENTS 29
9. FINANCING 30
REFERENCES 30
APPENDIX – Potential Adverse Events (AEs) 38

1. BACKGROUND AND RATIONALE

The overarching problem to be addressed in the PREPARE Trial is to improve the substantial adverse postoperative outcome burden faced by older people with frailty. This will be accomplished by testing an intervention (exercise prehabilitation) which aligns with evidence-based causal pathways, in a manner relevant to patients, clinicians and the healthcare system.

1.1. The majority of Canadians who have surgery are older and many live with frailty

Most people who have major (e.g., intra-abdominal, thoracic or vascular) surgery are ≥65 years[1] and are part of Canada's fastest growing demographic.[2] Four out of ten older Canadians who have surgery also live with frailty,[3] a syndrome that develops due to age- and disease-related deficits that accumulate across the lifespan.[4,5]

1.2. Frailty makes people vulnerable to adverse health outcomes

Major surgery induces physiologic stress at least equivalent to sustained moderate intensity exercise.[6,7] Therefore, given the vulnerability to stressors inherent in having frailty,[5] it is not surprising that work from our group[3,8–11] and others demonstrates that frailty before surgery is associated with significant risk. Frailty predicts a 2- to 3-fold increase in rates of: new patient-reported disability;[3] major complications;[12] readmission;[13] and dying.[9,14–17] Approximately 20% of older people with frailty develop a new disability[3] and 60% experience a serious complication.[12] Half of community-dwelling older people with frailty who have major elective surgery are not discharged back home;[3,10] length of stay (LoS)[3,10,18] and costs of care are also significantly increased.[3,10]

1.3. Findings from our group provide important insights into why frailty leads to adverse outcomes

First, surgery induces substantial physical and physiologic stress, which is poorly tolerated by older people with frailty. In two population-based studies of older people having major surgery, we found that the timing of postoperative deaths was much earlier for people with frailty than without.[14,19] This suggests that the vulnerability to stressors inherent in frailty may be especially relevant after major surgical stress, findings consistent with other studies. Specifically, a recent systematic review identified frailty as the strongest predictor of postoperative complications in older people, a 2.2-fold increase.[12] Frailty is also a key risk factor for death after a complication. [20,21] These findings support a causal pathway: *frailty-related vulnerability leads to postoperative complications from which the older person with frailty cannot recover.*

Second, although most older people with frailty survive their surgery,[22] a multicenter cohort study from our group found that almost 20% of older people with preoperative frailty develop a new patient-reported disability after elective surgery, an adjusted 2.5-fold increase.[3] The ability to stand and to walk were the most severely affected domains of disability (Main Appendix),[23] which is consistent with evidence that decreased muscle mass, which is common in frailty, contributes to poor functional outcomes.[24] *This suggests that physical dysfunction is a substantial contributor to disability after surgery in older people.*

Therefore, accumulating data to support the hypothesis that to improve postoperative outcomes for older people with frailty, interventions should aim to: 1) decrease physical and physiologic vulnerability, which should translate into lower complication rates and subsequent deaths; and 2) address contributors to physical dysfunction, which should decrease disability and subsequent loss of independence.

1.4. Interventions to improve postoperative outcomes for older people with frailty are infrequently studied, however, exercise prehabilitation is a promising therapy

Despite the adverse outcome burden associated with frailty, a systematic review from our team identified only 11 studies (1 668 total participants) that tested interventions to improve the outcomes of older people with frailty having surgery.[25] Exercise prehabilitation (i.e., exercise therapy performed before surgery) was the most common and most promising of the limited number of interventions tested.[25–28] Four of five identified exercise studies found that functional outcomes were improved. Milder and colleagues, in a separate review of prehabilitation programs in people with frailty, identified three additional studies in different procedural areas where prehabilitation was associated with decreased length of stay (LoS) and mortality.[28] Two other randomized trials involving individuals with frailty characteristics (i.e., older age, higher baseline illness severity and decreased functional capacity) suggest that exercise prehabilitation may be ***most*** effective in this high-risk population. Minella and colleagues demonstrated that older people with frailty characteristics made functional gains 2-fold higher than people with high baseline function and low illness severity after participating in the same exercise prehabilitation program.[29] Barberan-Garcia found that postoperative complications were reduced in older people with frailty characteristics by 50% relative to control.[26]

1.5. Care of older people having surgery and exercise before surgery are high-priority areas for patient-oriented research

As outlined in 1. 1., the characteristics of people having surgery in Canada are changing. In the coming decades the number of Canadians ≥65 years is expected to double.[2] We have documented a 10% increase in the proportion of older people with frailty having surgery in the past 10 years.[9] Therefore, frailty will increasingly contribute to adverse patient and system outcomes after surgery. Accordingly, James Lind Alliance (JLA) priority setting partnerships[30,31] have defined key areas of research that must be addressed, including: 1) improving the care of older people having surgery; 2) the role of exercise in improving surgical outcomes; and 3) the role of exercise in managing frailty. However, currently available studies of exercise prehabilitation rarely include older people with frailty, lack patient-reported outcomes, evaluate resource-intensive hospital-based interventions (limiting access), have been small and single centered, and are at high risk of bias. This means that our proposed low risk of bias multicenter trial of an exercise prehabilitation program, tailored to the needs of older people with frailty, is urgently needed and directly aligns with patient- and community-centered priorities for research.

1.6. Older people with frailty require tailored (i.e. type, location, duration) exercise prehabilitation programs to meet their unique needs

Despite the limited number of studies describing exercise prehabilitation programs for people with frailty having surgery, a systematic review of 47 exercise studies for older people with frailty *not having surgery* describe the needs of this population.[32] First, exercise appears to be most effective in people with mild (as opposed to severe) frailty. Our data shows that 86% of elective surgery patients with frailty have a Clinical Frailty Scale score of 4 or 5 (i.e., vulnerable or mildly frail, the two lowest categories still considered as frail),[3,4] suggesting older people with frailty having surgery are an ideal population to benefit from exercise. Next, efficacious programs featured multicomponent training (i.e., combinations of different types of exercise), although resistance training appeared to be the most important component. Programs where exercise was performed at least three times per week for 30-45 minutes were more efficacious than lower frequency programs. While not addressed in the systematic review, priority setting partnerships demonstrate that older people with frailty prioritize home-based services,[31] which may reflect limited access to transport, decreased mobility, or other factors.[33] The need for home-based programming is also consistent with preliminary results from our ongoing systematic review of factors influencing compliance with exercise therapy in older people, which demonstrates that lack of proximity to facilities decreases program compliance.[34] Finally, in the surgical setting, systematic reviews suggest that two to four weeks of exercise prehabilitation are needed to improve post-operative outcomes.[35]

1.7. Results from our pilot trial of home-based exercise prehabilitation in cancer patients with frailty demonstrate feasibility

To date, we have recruited 189/200 participants.[36] Our results show that our home-based program, performed three times per week for at least three weeks before surgery, is feasible and acceptable to older people with frailty. We have met our weekly recruitment targets, have achieved 95% participation rates with prescribed exercise and 96% complete outcome follow up at 30 days. Feasibility results of our small trial in surgical oncology are encouraging for expanding our work to a multicenter trial across major surgical procedures (to enhance generalizability), appropriately powered for outcomes that matter most to older people (disability and complications).[37,38]

1.8. Serious limitations prevent generalizing the exercise prehabilitation literature for people *without* frailty to people *with* frailty

An alternative to conducting a multicenter trial of exercise prehabilitation in a population of older people with frailty would be to generalize findings from studies of exercise prehabilitation conducted in people *without* frailty. However, despite systematic review findings that exercise prehabilitation improves physical performance and function,[35,39–41] decreases complication rates[40,41] and reduces LoS[39,40] in younger patients (mean age <65 years) without frailty,[25,35,39–41] limitations exist that preclude generalizability. First, 87% of identified studies were at high or unclear risk of bias and 79% were single center studies with <100 participants. Second, interventions did not meet the needs of people with frailty, as 85% were not home-based, which may be especially relevant in the Canadian context where older adults are over-represented in rural areas and may need to travel long distances to hospital.[42]

Consistent with these limitations, five of six systematic reviews evaluating exercise prehabilitation (combining frail and non-frail populations) concluded that more data are needed, specifically from large, methodologically robust multicenter trials at low risk of bias.[25,28,35,39,41]

1.9. Currently registered studies will not address pertinent knowledge gaps

We have reviewed currently registered exercise prehabilitation trials. Only three trials, all with sample sizes <140, none powered for patient-reported outcomes, and all limited to organ-specific surgeries (e.g., colorectal or cardiac) are currently planned or underway. Therefore, results of ongoing trials are unlikely to address patient-important questions or provide generalizable findings applicable to older people with frailty.

2. RESEARCH QUESTIONS

In older people with frailty having major elective surgery, is participation in a structured home-based exercise program, compared to standard care, effective in:

- Decreasing patient-reported disability scores 30 days after surgery and rates of in-hospital complications?
- Improving health related quality of life and decreasing healthcare resource use?
- Reducing costs to the Canadian health care system (health services and economic outcomes)?

3. STUDY OBJECTIVES

The PREPARE Trial aims to provide randomized trial evidence evaluating whether a home-based exercise program, which we have already shown to be feasible and acceptable for older people living with frailty, can do the following:

1. Improve patient-oriented disability scores (co-primary outcome)
2. Reduce complication rates (co-primary outcome), improve quality of life
3. Decrease healthcare resource use in older people with frailty having elective inpatient surgery

4. STUDY DESIGN

4.1. Trial Design

The PREPARE Trial is an accessor blinded individual patient parallel-arm randomized controlled trial conducted in 14 Canadian academic or community hospitals.

4.2. Intervention

Our intervention is a structured, home-based, multimodal exercise prehabilitation program *already successfully implemented in our pilot trial.*[36] The protocol was developed with kinesiologists and exercise scientists (CSB, JN): 1) informed by a protocol with proven efficacy in improving function

for non-frail surgical patients,[45–47] 2) tailoring movements to the needs and safety of people with frailty, and 3) specific feedback from participants with frailty in our pilot trial (obtained through structured qualitative and quantitative assessment[36]). Our data demonstrate that the intervention can be feasibly implemented, that 95% of older people with frailty who are randomized to exercise participate in the program; 87% of patients report that it is: easy to follow, enjoyable, well-suited to their needs and that lack of experience with exercise was not a barrier to participation.

Exercise will be prescribed as one-hour sessions, performed a minimum of three times per week for three weeks, consisting of: 1) strength training; 2) aerobic exercise and 3) flexibility.

Strength training: 1 set of 10 repetitions of each exercise: 1) push-ups (modified to the individual's level of function as bicep or tricep wall push-ups); 2) seated row (elastic resistance band); 3) chest fly (elastic resistance band); 4) deltoid lift (elastic resistance band); 5) biceps curls (elastic resistance band); 6) triceps extensions (elastic resistance band); 7) quadricep exercises; 8) hamstring curls; 9) standing calf raises; 10) modified chair-seated abdominal exercises.

Aerobics: Aerobic exercises are tailored to each participants' needs and preferences. If a participant identifies walking as a preferred aerobic activity, it will be suggested that they walk for 20 minutes at moderate intensity. After the first week, the individual's average daily step count is used to recommend a 10% increase in daily step count each week. People with frailty typically have low baseline step counts,[48] and a 10% increase per week is considered to be a safe, meaningful and achievable method to personalize activity goals.[49] If a participant selects swimming or exercising using a stationary bike, for example, increases will be prescribed by increasing the time they engage in their aerobic activity if it suits their comfort and safety levels. To accommodate the challenges of getting outdoors during winter or inclement weather, and due to COVID-19 restrictions, participants will be provided with a video for an aerobic cardio session (standing and seated options) that is safe for older adults and that can be done indoors.

Flexibility: Chest, arm, leg and trunk stretches; each stretch held for 20 seconds x 2 repetitions.

At the time of enrollment participants will be provided teaching on safe performance of prescribed exercises, a booklet describing the exercises with text and illustrations, instructional videos, a calendar to track progress and a tip sheet for how to overcome common challenges to engaging in exercise. Equipment will be provided including a resistance band (with instruction for how to increase the resistance by shortening the band) and a pedometer. Nutritional status will be supported using a pamphlet to inform all intervention participants of proper caloric and protein requirements . Upon receipt of the materials, a trained research assistant will provide exercise teaching and will collaboratively discuss individual goals, values and possible challenges with each participant to support motivation, safety and adherence. Throughout the program, participants will be supported by our experienced central team using weekly phone calls to monitor safety, encourage adherence and provide advice on exercise progression and nutritional intake. Participants can call or email the study center at any time with questions or concerns. Based on

preliminary results from our pilot trial, participants will be encouraged to identify an exercise partner or support person to increase the social aspects of exercise.

4.2.1. Control Group

To support blinding of control participants, they will receive the World Health Organization Recommendations for Physical Activity for People ≥65 Years pamphlet, as well as A Guide to Healthy Eating for Older Adults.[50] Control group participants do not receive the pedometer to wear before surgery. They are only asked to track their steps for 30 days after surgery.

4.3. Allocation and Randomization

The random allocation sequence will be computer-generated by an independent statistician at the Ottawa Methods Centre using permuted blocks of randomly varying lengths, stratified by center, planned minimally invasive vs. open surgery (minimally invasive surgery is associated with decreased rates of complications and faster recovery[51,52]) and cancer surgery. Study personnel will access the randomization sequence via a central web-based application to ensure allocation concealment.

4.4. Protection Against Bias

Use of minimal exclusion criteria and inclusion of a wide variety of surgical procedures will enhance external validity. Randomization will be stratified by center and surgical approach to minimize the risk of confounding. Performance bias will be minimized by randomizing participants after meeting their surgeon, allowing clinicians and outcome assessors to remain blinded to treatment allocation. The risks of response bias (due to reduced ability to blind patients to their allocated interventions) will be minimized by informing participants in both arms that they are being enrolled in a trial to test activity interventions before surgery and by providing control participants with an activity guideline. Missing data bias will be reduced through measures to minimize loss to follow-up (centralization of telephone follow-up and collection of data in-hospital) and through adjustment of factors associated with missingness.

4.5. Duration of Treatment

Exercise prehabilitation ***will be prescribed for at least three weeks:*** 1) Two to four weeks of exercise appear to be necessary to improve postoperative outcomes;[35] 2) A relevant exercise program should be effective in less than four weeks (this a benchmark duration from decision to operate to surgery in oncology);[53,54] and 3) A similar protocol in people without frailty demonstrated efficacy in a median of 24 days.[45] We will take a pragmatic approach above the minimum to account for variable waits for surgery between hospitals and indications for surgery. Our preliminary data in people with frailty demonstrate an average of five weeks of preoperative participation.

4.6. Inclusion/Exclusion Criteria

Inclusion Criteria	Exclusion Criteria
• Patient aged ≥60 years<br>• Scheduled, or on the pathway, for elective surgery with eLoS* ≥2 days<br>• Expected surgery date between 3 and 12 weeks from enrollment<br>• Clinical Frailty Scale (CFS) score ≥4/9	• Inability to speak English/French<br>• Co-morbidity preventing assessment or understanding of questionnaires<br>• Unable to be contacted by telephone<br>• Unwilling to participate in exercise program<br>• Cardiac, neurological or orthopedic procedure<br>• Palliative surgery<br>• Any of the following cardiovascular conditions:<br>o Severe valvular heart disease that limits a patient's ability to ambulate on level ground, or is associated with syncope or dyspnea<br>o Severe cardiac dysrhythmias that limits a patient's ability to ambulate on level ground, or is associated with syncope or dyspnea<br>o Recent myocardial infarction (within the 6 weeks prior to enrollment, based on the Heart & Stroke Foundation's HeartWalk Program)

*eLoS = Expected length of stay

4.7. Justification of Criteria

1) Surgical procedures: Abdominal, thoracic, pelvic, head-and-neck, and vascular procedures represent >60% of procedures f32or older people.[55] Cardiac, orthopedic, and neurosurgical procedures will be excluded as processes of care, recovery rates, and reasons for disability vary substantially from other major elective surgeries.

2) Frailty instrument: The Clinical Frailty Scale (CFS) is a clinically oriented instrument highly correlated with the Canadian Study of Health and Ageing Frailty Index (ρ=0.8)[4,56] and has high inter-rater reliability.[57,58] The accuracy of the CFS is similar to the Fried Phenotype (the most frequently used perioperative frailty instrument[12,59]) but the CFS is faster and easier to use.[3] A CFS score cut off of 4/9 maximizes sensitivity and specificity for predicting disability.[3]

3) Age: Inclusion of people 60-65 years will expand our participant pool and frailty is consistently associated with a greater impact on adverse outcomes in younger people than those in advanced age groups.[14,60]

4.8. Frequency and Duration to Follow-Up

A schedule of specific data elements for collection and timing of follow up is provided in the table below. Briefly, we will collect baseline demographic, comorbidity, function and surgical data. At the time of surgery full data on program compliance and safety events will be collected. While in hospital, complications, function, frailty and resource use outcomes will be collected. Telephone

follow-up will collect disability, quality of life, step count, readmission and survival data. Wearable technology linked to a mobile device could facilitate follow up, however our data shows only 22% of older surgical patients have a cell phone. All participants will be linked to health administrative data collect health system and resource use outcomes (Ontario-Institute for Clinical Evaluative Sciences (ICES); Alberta-Alberta Data Integration, Measurement & Reporting (DIMR); Manitoba-Manitoba Center for Health Policy-MCHP).

Enrollment	Leading up to Surgery	Postop in-hospital	30 days postop	90 days postop	365 days postop	Admin data
CFS,[4] PHQ-2,[61] Demographics, WHODAS,[62] EQ-5D,[63] AD8,[64] [65]DASI,[66] 5TSTS,[67] Katz,[68] Procedure, CNST, COVID-19 status	Step Counts, Adherence, TDF Participant program feedback (intervention group), Adverse Events, Self-reported change in exercise, COVID-19 status	POMS,[69] Falls, 5TSTS, Katz, CFS, LoS, Disposition, COVID-19 status	WHODAS, EQ-5D, Falls, Readmission, Step Count, Survival, COVID-19 status	WHODAS, EQ-5D, Falls, Readmission, Survival, COVID-19 status	WHODAS, EQ-5D, Falls, Readmission, Survival, COVID-19 status	Costs, Readmissions, Days alive at home, ED visits, Survival

AD8: AD8 Cognitive Screen; CFS: Clinical Frailty Scale; DASI: Duke Activity Status Index; ED: Emergency Department; EQ-5D: EuroQuoL health related quality of life; LoS: Length of Stay; PHQ: Patient Health Questionnaire; POMS: Postoperative Morbidity Survey; 5TSTS: 5 Times Sit to Stand; WHODAS: World Health Organization Disability Assessment Schedule; CNST: Canadian Nutrition Screening Tool

4.9. Primary Outcomes

The PREPARE Trial has co-primary outcomes (disability and complications), which were identified based on: high-priority outcomes for of older surgical patients (informed by our survey of older people and patient engagement in the protocol);[37,38] 2) proposed causal mechanisms between exercise prehabilitation and outcomes; and 3) systematic review efficacy data.[35,39–41]

4.9.1. In-hospital complications

The Postoperative Morbidity Survey (POMS), a prospectively administered instrument designed to identify significant in-hospital complications in key organ systems, will be used to define complications.[69,70] Individuals experiencing any POMS complication or dying in hospital will be said to have experienced a complication. The presence or absence of a complication is recorded based on objective criteria using items available from routine sources (i.e., charts, medication records, vital signs records, routine lab tests and direct questioning of the patient). Included items were generated directly from feedback by patients, nurses and physicians. The final survey was reviewed and approved by an international panel of surgeons and anesthesiologists[70] and contains 18 items addressing nine domains (i.e., pulmonary, infectious, renal, gastrointestinal, cardiovascular,

neurological, hematological, wound, pain). In validation it had high inter-rater agreement (kappa=0.94-1.0), was acceptable to all patients and demonstrated construct validity.[69] Subsequent validation studies confirmed these findings.[71,72] The POMS tool has been used in many international studies.[36,72,81,73–80] To support secondary analyses, complication severity will be graded using the widely used updated Clavien-Dindo Classification,[82–88] a validated tool which grades severity based on the therapy used to treat the complication.[82,89,90] The American Society for Enhanced Recovery and Perioperative Quality Initiative have recently published an international joint statement recommending POMS for measurement of postoperative complications and the Clavien-Dindo Classification for grading of severity.[91]

4.9.2. Patient-reported disability 30 days after surgery

We will use the World Health Organization Disability Assessment Schedule 2.0 (WHODAS), a patient-reported disability scale that assesses limitations in six major life domains (i.e., cognition, mobility, self-care, social interaction, life activities, participation in society).[62,92] The WHODAS has been validated in surgical patients[93] (and other acute and chronic conditions[94–100]), was used by our group in a recent multicenter cohort study of older surgical patients[3] and was identified by older surgical patients as a high-priority outcome.[3,38] Each questionnaire item is scored on a Likert scale ranging from 0 to 4. The sum of the responses is the WHODAS Disability Score (range: 0 to 48), which is expressed as a percentage of the maximum possible score (Survey Appendix). People who die prior to follow up will be scored as completely disabled. Based on normative data, a mean difference of 8% for WHODAS scores is meaningful.[39] In extensive psychometric evaluation in the non-operative setting, it had high test-retest reliability, high internal consistency, good concurrent validity, and conformity to Rasch scaling properties.[62] In a cohort study of 510 surgical patients, it had good to excellent clinical acceptability, internal consistency, scaling properties, responsiveness, criterion validity, and construct validity.[93] The WHODAS 2.0 is thus a feasible, valid, and reliable patient-reported instrument to measure disability in older surgical patients.

4.10. Secondary Outcomes

4.10.1. Function

Daily and total step counts, measured using a pedometer, predict adverse post-hospitalization outcomes and reflect physical recovery. These will be recorded daily for the duration of the exercise program (for those in the intervention group) and 30 days after surgery (for all participants) to evaluate functional recovery.[101–103] The 5 Times Sit to Stand (5TSTS) is a validated test used for measuring the risk of falls and predicting disability in older adults.[67] The Katz Index measures function in activities of daily living.[68] Any falls will be documented.

4.10.2. Health-related quality of life

The EQ-5D-5L is a well-validated instrument with Canadian valuation statistics and national implementation,[63,104,105,106] used to measure health-related quality of life at baseline, 30, 90, and 365 days after surgery and to inform incremental cost per quality-adjusted life year gained.

4.10.3. All-cause mortality

All deaths and death dates will be identified in-hospital, by chart review or through telephone follow-up.

4.10.4. Health System

Discharge disposition (home, home with support, rehabilitation, long term care) will be prospectively collected at discharge; readmissions by telephone. Linkage to ICES/DIMR/MCHP data will allow for collection of health system costs,[107] readmissions, emergency department visits and subsequent long-term care admissions in the year after surgery.[108]

4.10.5. Participant Feedback

A Theoretical Domains Framework[109,110] participant survey will identify barriers and facilitators to participation.

4.10.6. Safety

Adverse events (AEs) that occur during or within 24 hours of engaging in any exercise specific to the prehabilitation intervention will be collected during the exercise treatment period for the intervention group. Participants in the intervention group will also be asked each week during the exercise treatment period if they experienced any falls. For the control group, participants will be asked if they experienced any falls since the time of enrollment up until the day before surgery. Expert adjudication will determine the need to report AEs to the Research Ethics Board (REB). Please note that all participating sites seeking approval through CTO Stream must follow the Board of Record's SOPs (i.e., OHSN-REB's SOPs).

4.11. Outcome Measurement at Follow-Up

All participants will be followed up to 1 year after surgery or death, whichever occurs first. As described in section 4.9. and 4.10., outcomes will be measured using validated instruments either in-hospital or at regularly scheduled telephone follow-up from the central study team.

4.12. Sample Size

Based on pre-COVID-19 pandemic sample size calculations, our total sample size of 750 participants (375 per arm) was driven by the binary co-primary outcome (i.e., in-hospital complications). We have assumed a control arm complication rate of 55%, informed by data from our prospective cohort study (where complications were measured using POMS, see unpublished data Main Appendix)[3] and by systematic reviews of complications in older people with frailty having surgery.[12] Available effect size estimates, from a recent low risk of bias RCT[26] (RR=0.5, 95%CI 0.3-0.8) and a systematic review[40] (OR 0.41, 95%CI 0.28-0.62)), suggest exercise prehabilitation can reduce postoperative complications by a relative 50%.[26,40] We would consider a target difference of a 25% relative reduction to be both clinically important and plausible in a multicenter study including diverse surgical procedures and after accounting for imperfect compliance. In keeping with our intention-to-treat analysis, a sample size of 750 participants achieves 90% power to detect our target difference (i.e., a relative difference of 25% or absolute difference of 14%) using an unpooled Z-test with an alpha significance level of 0.025 (to account for our two pairwise comparisons for our two primary outcomes). This calculation accounts for a 10% non-compliance factor largely consisting of randomized participants not having their planned

surgery to reflect the real world clinical scenario.[111] We did not account for missing complications data as we anticipate complete observation as they are measured in-hospital. For the continuous co-primary outcome (i.e., WHODAS Disability Score at 30 days) this sample size achieves 98% power to detect a minimum clinically important difference of 8 points on a 100-point scale (or a difference of 7 points with 90% power) using an ANCOVA analysis at the two-sided 2.5% significance level. This calculation assumes a common standard deviation of 25, a correlation between baseline and postoperative score of 0.4 (informed by our multicenter cohort study)[3] and accounts for 8% missing data at 30 days and 10% non-compliance (as was assumed for complications). Due to surgical delays and cancellations as a result of the COVID-19 pandemic, our trial sample size has seen more participants having surgery outside of their expected time to operation (i.e., following the 'Day 84' procedures (Section 4.16)) than originally predicted. To account for this, and to ensure adequate power for our analyses, our final sample size will be 850 participants.

4.13. Health Services Research Issues

As listed in 4.10.4., health-related quality of life, healthcare resource use and economic outcomes will be measured prospectively and through data linkage. Description of cost-effectiveness, cost utility and other health economic analyses are provided in the appendix.

4.14. Recruitment

We have experience successfully recruiting older individuals with frailty. Based on our preliminary data[36] 40% of older surgical patients eligible for frailty screening were successfully enrolled (94% of screen-eligible participants were willing to be screened; 50% of screened people met inclusion criteria; 85% of fully eligible individuals enroll). Enrollment in the PREPARE multicenter trial (which will recruit individuals having surgery for benign and oncologic reasons) may be higher as our pilot trial was limited to cancer patients, who are less likely to enroll in trials due to their high burden of competing appointments and adjuvant treatments.[112] In our multicenter cohort study, 85% of screen-eligible individuals were enrolled.[3] *Based on surgical volumes of people ≥60 years over the past two years at confirmed study centers (Main Appendix), and estimating 40% successful enrollment, we anticipate that 30 patients per month will be recruited.*

4.15. Adherence

Systematic reviews demonstrate high variation in compliance with exercise prehabilitation (16-100%),[41] and that dropout rates in exercise interventions typically exceed 20%.[113] Preliminary results from our pilot study demonstrate 95% participation (i.e., <5% dropout or non-participation after being randomized to exercise), and that participants complete 72% of all prescribed exercise, which is higher than the average completion rate from our systematic review of prescribed exercise in older people (mean=70%).[34] We have also achieved 83% compliance in a separate study from our group of exercise in older surgical patients with frailty.[114] Through weekly monitoring and support of participants by an experienced central team and enhancements of the PREPARE Trial intervention (personalization of goals and encouragement of social engagement with an exercise partner) we expect to meet or exceed the level of compliance found in our pilot study.

Adherence will be considered by the following criteria: a) any cardio attempted – 1 point, b) 5/10 strength exercises attempted – 1 point and c) 3/6 stretches attempted – 1 point. Because these components are to be completed 3 times a week, the total number of points in a week is 9 points. Therefore, in the final 4 weeks of the exercise intervention (or the final 4 weeks where there is adherence data for a participant), a participant has the possibility of obtaining 36 points. To be considered adherent (yes vs no), a participant must score 29/36 (80%). If a participant is only in the program for 2 or 3 weeks, the denominator will be 27. A secondary definition of moderate adherence will be defined as at least 25/36 (70%).

4.16. Lost to Follow-Up

Some randomized patients (11% in our preliminary data) will not have their planned surgery (this may occur due to cancellation or disease progression requiring emergency surgery), but will be included in the intention to treat (ITT) population to avoid introducing bias.[111] For example, if an exercise-related adverse event lead to cancellation of surgery this must be maintained in the analysis, as would a surgery that was cancelled due to exercise-related decreases in symptoms negating the need for surgery. These individuals will have follow up dates and data collection based on 12 weeks from enrollment, or 84 days from enrollment. The following data will be collected from this timepoint: discharge data (falls, 5TSTS, KATZ, CFS, COVID-19) on day 91 from enrollment, 30 day step-count from day 85 onward, and the 30, 90, 365 day follow-up calls. Of course, no POMS or hospital-level data will be collected as the surgery did not take place. In the event that a participant has their surgery rescheduled within 30 days from day 84, the following will be collected from their actual surgery date: in-hospital data collection (POD 3, 5, 7 and discharge), and the 30 day follow-up call. For those in the intervention group where this occurs, adherence data will only be captured leading up to the day 84 timepoint. We have achieved 96% complete outcome data in our single center study, and >92% in a previous multicenter study.[3,36]

If a participant ends up having surgery within 3 weeks of enrollment, they will remain in the trial and will be included in the ITT analysis. In the event that a participant does not have their planned surgery (i.e., due to disease progression), they will remain in the study and their surgical procedure will be indicated as "did not occur as planned".

In the event that a participant develops a cognitive impairment or an impairment that prevents them from being able to respond to study questionnaires, a proxy may complete the follow-up measures if the participant provided consent for this at enrollment. The EDC will distinguish between measures that were completed by the participant or their proxy.

4.17. Study Centres

Our study will recruit patients from 14 centers in Ottawa, Toronto, Hamilton, Edmonton, Sudbury, Kingston, Calgary, Vancouver and Winnipeg. These study centers represent academic and community hospitals.

5. DATA ANALYSIS

Analysis will follow ITT principles. The ITT population will be defined as all randomized participants.[111] Descriptive statistics will compare study arms at baseline. Factors associated with missing outcome data will be examined using logistic regression.

5.1. Primary Outcomes

In-hospital complications will be analyzed using robust Poisson regression to yield relative risk estimates[115,116] and 97.5% confidence intervals. The analysis will include fixed terms for study arm, the stratification factor (surgical approach), and prespecified covariates (as adjustment for known prognostic factors can substantially increase power[117–119]): age, gender, surgery type, malignancy, frailty score, and factors associated with attrition,[120,121] and will account for the center effect. Absolute risk differences will also be reported.

WHODAS Disability score at 30 days will be analyzed using ANCOVA with the baseline measure entered as a covariate,[122] together with terms for the stratification factor and the prespecified covariates listed above. A random effect for center will account for the multicenter trial design. The intervention effect will be expressed as an adjusted mean difference with 97.5% confidence interval. Secondary repeated measures analyses of all disability score measurements (up to 365 days) will use restricted maximum likelihood estimation and model the covariance matrix to account for correlation in the four repeated measures over time. The model will constrain differences between the arms at baseline[122] by including fixed terms for time and arm by time interaction in addition to the covariates specified above and the random center effect. The difference between the treatment and control arms at 90 and 365 days will be estimated using adjusted least square mean differences.

5.2. Secondary Outcomes

All adjusted analyses will account for the covariates specified in the primary analysis and will account for center effects. Health-related quality of life measures will be analyzed as described for disability. Step counts will be analyzed using linear regression. Time to hospital discharge will be analyzed using Cox regression with in-hospital mortality as a competing risk. Overall survival will be analyzed using Cox regression. Discharge disposition will be analyzed using multinomial logistic regression. Health system outcomes (readmissions, emergency department visits and subsequent long-term care admissions) will be analyzed using robust Posisson regression. Binary safety outcomes will be analyzed as described for complications using Poisson regression or exact methods if event numbers are small. Costs analysis will use log-gamma regression.[123] From the perspective of Canada's healthcare system, we will conduct a cost-utility analysis to assess whether exercise prehabilitation offers value for money. Health care utilization and the efficacy of the intervention will be obtained from the trial. We expect attrition to be low; nevertheless, to account for any missing data in our ITT analyses, all eligible patients will be included in all analyses and baseline covariates associated with missing data will be included as covariates.

5.3. Additional Analyses

We will perform a per protocol analysis (individuals who actually had surgery and with >80% completion of prescribed exercise sessions considered as the per protocol population).

5.4. Planned Subgroup Analyses

The primary outcomes will be analyzed in pre-specified subgroups that we postulate may have differing responses to the intervention: gender, age (<75 vs ≥75[124]), cancer, depression, frailty (4 vs ≥5). Compliance rates will be compared by gender. These analyses will be conducted by including interaction terms between the subgroup indicator variables and the intervention.

6. STUDY PROCEDURES

All study data, including questionnaires, will be captured electronically using an iPad or computer.

6.1. Informed Consent

Eligible patients may only be included in the study after providing written informed consent or verbal informed consent (i.e., remotely over the telephone) to participate. Informed consent must be obtained prior to starting any study procedures. The process of obtaining informed consent should be documented in the patient's source documents.

If the participant provides their written consent to participate in person, the site investigator must keep the fully signed original Main Informed Consent Form (i.e., signed by both the patient and individual obtaining consent) in the study file, providing a photocopy to the participant for their records. It is the site investigator and/or trained delegate's responsibility to document the details of the consent discussion in the participant notes.

If the participant provides their verbal consent to participate over the telephone, the individual obtaining consent is required to sign the Verbal Informed Consent Form. The signed Verbal Informed Consent Form will be saved in the study file and details of the consent discussion will be documented in the participant notes. A copy of the Main Information Sheet (no signature pages) will be provided to the participant for their records by email or by mail, depending on the participant's preference. The potential participant will also be provided with the opportunity of receiving a copy of the Main Information Sheet (by email or mail) to review before providing their consent to participate.

Regardless of the method of consent, participants will be given the option to name a proxy (family member, caregiver, or friend) who may be contacted to complete follow-up data collection (i.e., questionnaires) on their behalf, should they be incapable of doing so.

6.2. Surgical Consultation Baseline Visit (expected between 3 and 12 weeks from date of surgery)

The baseline visit will take place in person or by telephone. After the patient has been deemed eligible and the ICF has been signed or verbal informed consent has been obtained, the following will be assessed:

- Demographic Questionnaire

- World Health Organization Disability Assessment Survey (WHODAS 2.0)
- 5 Times Sit to Stand (5TSTS)
- EuroQuol Health Related Quality of Life (EQ5D5L)
- Patient Health Questionnaire (PHQ-2)
- Duke Activity Status Index (DASI)
- Katz Index of Independence in Activities of Daily Living (KATZ)
- AD8: Cognitive Screen
- Canadian Nutrition Screening Tool (CNST)
- COVID-19 status

6.3. Weekly Adherence Calls

Throughout the duration of the prehabilitation program, participants will receive weekly phone calls from the central coordinating site to gauge adherence to the exercise program, suggest any modifications to the program as needed, and provide overall support and track any adverse events and falls that may have taken place. If the coordinating centre is unable to reach a participant one week to complete their adherence call, the adherence data will be collected retrospectively at the next scheduled adherence call. If a participant does not have a surgery date booked by day 84, their prehabilitation program will end on day 84. If at day 84 from enrollment, a participant has not had surgery but has a known surgery date booked, their prehabilitation program extends until their surgery date.

During the participant's final adherence call, they will be asked to complete the TDF questionnaire providing their feedback on the prehabilitation program.

6.4. Day Before Surgery

Before the day of surgery (1-5 days), the coordinating centre will document the following either in person or over the telephone:

- Step count from enrollment to surgery (intervention group)
- Self-reported change in exercise
- Number of falls (if any)
- Adverse events (if any)
- COVID-19 status

6.5. Day of Surgery

The following data will be collected by chart review:

- Whether the surgery was performed, if the planned surgery took place, surgical procedure, surgery type (minimally or non-minimally invasive), surgery date and immediate postoperative disposition

Overall adherence and adherence during the final 2, 3 or 4 weeks of the prehabilitation program will be auto-calculated.

6.6. In-Hospital Follow-Up

For patients having surgery outside of the recruiting institution, only patient-reported data will be collected for the in-hospital follow-up (postoperative days 3, 5, and 7 and discharge). Data collected by chart review will be documented as missing.

Postoperative Days 3, 5 and 7:
If able, postoperative follow-up visits should be conducted in person. If an in-person visit is not feasible, data may be collected over the telephone.

- Postoperative Morbidity Survey (POMS) conducted via chart review and clinician/nurse reported
- Patient-reported Postoperative Morbidity Survey (POMS) **for neurological complications only**
- Katz Index of Independence in Activities of Daily Living (KATZ)
- Number of falls (if any)
- COVID-19 status

Day of Discharge:
If able, day of discharge data should be conducted in person. If an in-person visit is not feasible, data may be collected over the telephone and within 3 days of hospital discharge.

- Postoperative Morbidity Survey (POMS) conducted via chart review and clinician/nurse reported
- Patient-reported Postoperative Morbidity Survey (POMS) **for all complications**
- 5 Times Sit to Stand (5TSTS)
- Katz Index of Independence in Activities of Daily Living (KATZ)
- Clinical Frailty Scale (CFS)
- Length of Stay (LoS)
- Disposition (discharge location)
- Number of falls (if any)
- COVID-19 status

Provided the participant remains in hospital, research personnel will collect data on day 3, 5, 7 and discharge. In circumstances where the participant is discharged prior to post-op day 3, only data on the date of discharge will be collected.

If a participant is still admitted at postoperative day 30, their discharge data will be collected on postoperative day 30. The participant's actual length of stay and discharge date will be recorded.

6.7. Post-Discharge Follow-Up

Participants will be contacted by phone at 3 time-points after hospital discharge (e.g. 30 days (+/- 3 days), 90 days (+/- 7 days) and one year after surgery (+/- 7 days) for follow-up). To ensure high quality standardized follow-up, post-hospital discharge assessments will be performed solely by the central coordinating site.

6.7.1. 30-Day Phone Follow-Up

Participants will receive a telephone call from the central coordinating site to document the following:

- World Health Organization Disability Assessment Survey (WHODAS 2.0)
- EuroQuol health related quality of life (EQ5D5L)
- Readmission
- Step Count
- Survival
- COVID-19 status

6.7.2. 90-Day Phone Follow-up

Participants will receive a telephone call from the central coordinating site to document the following:

- World Health Organization Disability Assessment Survey (WHODAS 2.0)
- EuroQuol health related quality of life (EQ5D5L)
- Readmission
- Survival
- COVID-19 status

6.7.3. One-Year Phone Follow-Up

Participants will receive a telephone call from the central coordinating site to document the following:

- World Health Organization Disability Assessment Survey (WHODAS 2.0)
- EuroQuol health related quality of life (EQ5D5L)
- Readmission
- Survival
- COVID-19 status

6.8. Study Schedule of Events

	Time 0: Screening	Time 1: Baseline	Time 2: Weekly Phone Calls	Time 3: Day Before Surgery	Time 4: Day of Surgery	*Time 5: In-Hospital Follow-Up	Time 6: 30-Day Phone Follow-Up	Time 7: 90-Day Phone Follow-Up	Time 8: One-Year Phone Follow-Up
**Inclusion/Exclusion**	x								
**Informed Consent**	x								
**Demographics**		x							
**WHODAS 2.0**		x					x	x	x
**5TSTS****		x				x			
**EQ5D5L**		x					x	x	x

**PHQ-2**		x							
**DASI**		x							
**KATZ**		x				x			
**AD8**		x							
**CNST**		x							
**TDF Participant Feedback (Intervention group only)**				x					
**Step Count**					x		x		
**Pre-Operative Questionnaire**				x					
**Exercise Program Adherence (Intervention group only)**			x						
**Number of Falls**						x	x		
**POMS (chart review, patient-reported and clinician/nurse reported)**						x			
**CFS****	x					x			
**LoS****						x			
**Disposition****						x			
**Readmission**							x	x	x
**Survival**							x	x	x
**COVID-19 Status**		x	x	x		x	x	x	x

*In-hospital follow-up data collection takes place on day 3, 5, 7 and discharge. If patient is discharged prior to day 3, only discharge data is collected.

**Denotes in hospital data collection that only takes place on date of discharge.

Please refer to section **6.5 In-Hospital Follow-Up (day 3, 5, 7 and discharge)** for guidelines on POMS data collection.

7. DATA COLLECTION MANAGEMENT

7.1. Source Documents

Source documents are original documents, data and records, or certified copies of original records of clinical findings and/or observations. (e.g. hospital records, patient charts, participant files, etc.). All data entered into the eCRF must be supported by source documents. Source documents shall be made available at the request of the sponsor-investigator.

Sites will be provided with the eCRF which is to be used as a tool for data collection. Whenever possible, source data should be transcribed directly into the eCRF. However, the eCRF will serve as

the source document for any assessments that are completed. Any changes made to data already entered into the eCRF shall be done by research personnel or the site investigator. These changes will automatically be recorded within user history of the EDC system.

7.2. Case Report Forms

An Electronic Data Capture (EDC) system will be developed by the Ottawa Methods Centre and will be primarily used for study data collection. Data collection will be completed by research personnel or the site investigator. Post-discharge data will be collected via telephone by authorized research staff at the central coordinating center and subsequently entered in the eCRF. Appropriate security measure will be taken to authorize study site personnel using unique usernames and passwords prior to entering any data in the EDC.

The study data will be housed on a secure in-house server at the Ottawa Methods Centre throughout the duration of the study and up to 10 years after study completion.

All eCRF corrections will be documented within the EDC. A history of changes as well as the user who made the change will be automatically recorded.

7.3. Protocol Deviations

A protocol deviation occurs when there is a departure from the approved protocol's procedures. This departure from approved research can be made by the patient, research staff, or site investigator. When a protocol deviation occurs, the site-investigator or authorized research personnel must document and discuss the deviation with the coordinating site.

7.4. Record Retention

The site must maintain adequate and accurate records to enable the conduct of the study to be fully documented and the study data to be subsequently verified. The Investigator Site File (ISF) will contain the study's essential documents.

Study records at each site should be stored as per local requirements. If there are no local requirements, they should be retained for 10 years after the completion of the trial.

8. MANAGEMENT AND REPORTING OF ADVERSE EVENTS

There are many symptoms and events that can occur during the perioperative journey for older adults with frailty. For example, surgical complications, extended length of hospital stay, readmission, and death are all possible risks of surgery. Therefore, we will track (S)AEs that may occur during the physical measure of the 5TSTS and during or within 24 hours after engaging in the exercise intervention. Participants in both groups will be asked about any falls occurring between enrollment and surgery.

Appendix A will provide a list of possible expected adverse events that could occur during the different stages of the PREPARE Trial. If participants report any of the mentioned discomforts or symptoms, or any other (S)AEs during these timepoints, the research study staff will document it in the (S)AE Log and will notify the site-investigator. For example, all participant deaths occurring

between enrollment and date of surgery will be documented and assessed by the site investigator. Further, all falls occurring during or within 24 hours after exercise (intervention arm participants only) will also be documented and assessed. If the site-investigator concludes that the event is deemed serious, unexpected and may be associated with participation in the study, they shall notify the Coordinating Centre and follow their REB Board of Record's SAE reporting guidelines. Please note that participating sites seeking REB approval through CTO Stream must follow the Board of Record's SOPs (i.e., OHSN-REB's SOPs).

9. FINANCING

This study is funded by the Canadian Institutes of Health Research (CIHR) and The Ottawa Hospital Academic Medical Organization (TOHAMO).

REFERENCES

1. Etzioni DA, Liu JH, Maggard MA, Ko CY. The aging population and its impact on the surgery workforce. Ann Surg. 2003;238(2):170-177. doi:10.1097/01.SLA.0000081085.98792.3d.
2. Statistics Canada. The Canadian Population in 2011: Age and Sex. Ottawa, ON; 2011. http://www12.statcan.gc.ca/census-recensement/2011/as-sa/98-311-x/98-311-x2011001-eng.cfm#a2.
3. McIsaac DI, Taljaard M, Bryson GL, et al. Frailty as a Predictor of Death or New Disability After Surgery: A Prospective Cohort Study. Ann Surg. 2018;accepted. doi:10.1097/SLA.0000000000002967.
4. Rockwood K, Song X, MacKnight C, et al. A global clinical measure of fitness and frailty in elderly people. CMAJ. 2005;173(5):489-495. doi:10.1503/cmaj.050051.
5. Fried LP, Ferruci L, Darer J, Williamson J, Anderson G. Untangling the concepts of disability, frailty and comorbidity: Implications for improved targeting and care. J Gerontol A Biol Sci Med Sci. 2004;59(3):M255-M263.
6. Fleisher LA, Fleischmann KE, Auerbach AD, et al. 2014 ACC/AHA guideline on perioperative cardiovascular evaluation and management of patients undergoing noncardiac surgery: a report of the American College of Cardiology/American Heart Association Task Force on practice guidelines. J Am Coll Cardiol. 2014;64(22):e77-137. doi:10.1016/j.jacc.2014.07.944.
7. Jetté M, Sidney K, Blümchen G. Metabolic equivalents (METS) in exercise testing, exercise prescription, and evaluation of functional capacity. Clin Cardiol. 1990;13(8):555-565. http://www.ncbi.nlm.nih.gov/pubmed/2204507.
8. McIsaac DI, Bryson GL, Van Walraven C. Association of frailty and 1-year postoperative mortality following major elective noncardiac surgery: A population-based cohort study. JAMA Surg. 2016;151(6). doi:10.1001/jamasurg.2015.5085.
9. McIsaac DI, Moloo H, Bryson GL, Van Walraven C. The association of frailty with outcomes and resource use after emergency general surgery: A population-based cohort study. Anesth Analg. 2017;124(5). doi:10.1213/ANE.0000000000001960.
10. McIsaac DI, Beaule PE, Bryson GL, van Walraven C. The impact of frailty on outcomes and healthcare resource utilization after total joint arthroplasty: a population-based cohort study. Bone Jt J. 2016;98:799-805.
11. McIsaac DI, Wong CA, Huang A, Moloo H, van Walraven C. Derivation and Validation of a Generalizable Preoperative Frailty Index Using Population-based Health Administrative Data. Ann Surg. 2018;epub. doi:10.1097/SLA.0000000000002769.

12. Watt J, Tricco AC, Talbot-Hamon C, et al. Identifying older adults at risk of harm following elective surgery: a systematic review and meta-analysis. BMC Med. 2018;16(1):2. doi:10.1186/s12916-017-0986-2.
13. Li Y, Pederson J, Churchill T, et al. Impact of frailty on outcomes after discharge in older surgical patients: a prospective cohort study. CMAJ. 2018;190(7):184-190. doi:10.1503/cmaj.161403.
14. McIsaac DI, Bryson GL, van Walraven C. Association of Frailty and 1-Year Postoperative Mortality Following Major Elective Noncardiac Surgery: A Population-Based Cohort Study. JAMA Surg. 2016;151(6):538-545. doi:10.1001/jamasurg.2015.5085.
15. Kim DH, Kim CA, Placide S, Lipsitz LA, Marcantonio ER. Preoperative Frailty Assessment and Outcomes at 6 Months or Later in Older Adults Undergoing Cardiac Surgical Procedures. Ann Intern Med. 2016. doi:10.7326/M16-0652.
16. Lin H-S, Watts JN, Peel NM, Hubbard RE. Frailty and post-operative outcomes in older surgical patients: a systematic review. BMC Geriatr. 2016;16(1):157. doi:10.1186/s12877-016-0329-8.
17. Wang J, Zou Y, Zhao J, et al. The Impact of Frailty on Outcomes of Elderly Patients After Major Vascular Surgery: A Systematic Review and Meta-analysis. Eur J Vasc Endovasc Surg. August 2018. doi:10.1016/j.ejvs.2018.07.012.
18. Cooper Z, Rogers SO, Ngo L, et al. Comparison of Frailty Measures as Predictors of Outcomes After Orthopedic Surgery. J Am Geriatr Soc. 2016. doi:10.1111/jgs.14387.
19. McIsaac DI, Moloo H, Bryson GL, van Walraven C. The Association of Frailty With Outcomes and Resource Use After Emergency General Surgery: A Population-Based Cohort Study. Anesth Analg. 2017;124(5):1653-1661. doi:10.1213/ANE.0000000000001960.
20. Shah R, Attwood K, Arya S, et al. Association of Frailty With Failure to Rescue After Low-Risk and High-Risk Inpatient Surgery. JAMA Surg. 2018;48202:e180214. doi:10.1001/jamasurg.2018.0214.
21. Arya S, Kim SI, Duwayri Y, et al. Frailty increases the risk of 30-day mortality, morbidity, and failure to rescue after elective abdominal aortic aneurysm repair independent of age and comorbidities. J Vasc Surg. 2015;61(2):324-331. doi:10.1016/j.jvs.2014.08.115.
22. McIsaac DI, Wijeysundera DN, Huang A, Bryson GL, van Walraven C. Association of the Hospital Volume of Frail Surgical Patients Cared for with Outcomes after Elective, Major Noncardiac Surgery: A Retrospective Population-based Cohort Study. Anesthesiology. 2017;126(4):602-613. doi:10.1097/ALN.0000000000001536.
23. World Health Organization. International Classification of Functioning, Disability and Health. Geneva
24. Han A, Bokshan SL, Marcaccio SE, DePasse JM, Daniels AH. Diagnostic Criteria and Clinical Outcomes in Sarcopenia Research: A Literature Review. J Clin Med. 2018;7(4). doi:10.3390/jcm7040070.
25. McIsaac DI, Jen T, Mookerji N, Patel A, Lalu MM. Interventions to improve the outcomes of frail people having surgery: A systematic review. Quinn TJ, ed. PLoS One. 2017;12(12):e0190071. doi:10.1371/journal.pone.0190071.
26. Barberan-Garcia A, Ubré M, Roca J, et al. Personalised Prehabilitation in High-risk Patients Undergoing Elective Major Abdominal Surgery : A Randomized Blinded Controlled Trial. Ann Surg. 2018;267(1):50-56. doi:10.1097/SLA.0000000000002293.
27. Scheede-Bergdahl C, Awasthi R, Munden J, Loiselle SE, Carli F. Multimodal prehabilitation in cancer patients. Who benefits? Can J Anaesth. 2015;62(1):1496-8975.
28. Milder DA, Pillinger NL, Kam PCA. The role of prehabilitation in frail surgical patients: A systematic review. Acta Anaesthesiol Scand. 2018;(July):1-11. doi:10.1111/aas.13239.

29. Minnella EM, Awasthi R, Gillis C, et al. Patients with poor baseline walking capacity are most likely to improve their functional status with multimodal prehabilitation. Surgery. 2016;160(4):1070-1079. doi:10.1016/j.surg.2016.05.036.
30. Boney O, Bell M, Bell N, et al. Identifying research priorities in anaesthesia and perioperative care: final report of the joint National Institute of Academic Anaesthesia/James Lind Alliance Research Priority Setting Partnership. BMJ Open. 2015;5(12):e010006. doi:10.1136/bmjopen-2015-010006.
31. Canadian Frailty Network (CFN). Top Ten Frailty Priorities. http://www.cfn-nce.ca/engagingcanadians/helping-to-set-frailty-priorities/top-ten-frailty-priorities-identified-by-citizens/. Published 2017. Accessed July 17, 2018.
32. Theou O, Stathokostas L, Roland KP, et al. The effectiveness of exercise interventions for the management of frailty: a systematic review. J Aging Res. 2011;2011:569194. doi:10.4061/2011/569194.
33. Frost R, Kharicha K, Jovicic A, et al. Identifying acceptable components for home-based health promotion services for older people with mild frailty: A qualitative study. Health Soc Care Community. 2018;26(3):393-403. doi:10.1111/hsc.12526.
34. Pilon S, Vierula M, McIsaac DI. Evaluation of factors that predict exercise compliance in older people with medical and surgical conditions. In: Candian Anesthesiology Society Annual Meeting. Montreal, QC; 2018.
35. Mainini C, Rebelo PF, Bardelli R, et al. Perioperative physical exercise interventions for patients undergoing lung cancer surgery: What is the evidence? SAGE open Med. 2016;4:2050312116673855. doi:10.1177/2050312116673855.
36. McIsaac DI, Saunders C, Hladkowicz E, et al. PREHAB study: a protocol for a prospective randomised clinical trial of exercise therapy for people living with frailty having cancer surgery. BMJ Open. 2018;8(6):e022057. doi:10.1136/bmjopen-2018-022057.
37. Fried TR, Bradley EH, Towle VR, Allore H. Understanding the treatment preferences of seriously ill patients. N Engl J Med. 2002;346(14):1061-1066. doi:10.1056/NEJMsa012528.
38. Shaw J, Beasley E, Hladkowicz E, et al. A survey of older people after major surgery: Prioritization of routine and patient-reported postoperative outcome measures. In: Ottawa Quaity & Patient Safety Conference. Ottawa, ON; 2018.
39. Santa Mina D, Clarke H, Ritvo P, et al. Effect of total-body prehabilitation on postoperative outcomes: a systematic review and meta-analysis. Physiotherapy. 2014;100(3):196-207. doi:10.1016/j.physio.2013.08.008.
40. Marmelo F, Rocha V, Gonçalves D. The impact of prehabilitation on post-surgical complications in patients undergoing non-urgent cardiovascular surgical intervention: Systematic review and metaanalysis. Eur J Prev Cardiol. January 2018:204748731775237. doi:10.1177/2047487317752373.
41. Luther A, Gabriel J, Watson RP, Francis NK. The Impact of Total Body Prehabilitation on PostOperative Outcomes After Major Abdominal Surgery: A Systematic Review. World J Surg. 2018. doi:10.1007/s00268-018-4569-y.
42. Laurent S. Rural Canada: Access to Health Care.; 2002. http://publications.gc.ca/CollectionR/LoPBdP/BP/prb0245-e.htm#Summary.
43. Straus SE, Tetroe J, Graham I. Defining knowledge translation. CMAJ. 2009;181(3-4):165-168. doi:10.1503/cmaj.081229.
44. Canadian Institutes of Health Research. Guide to Knowledge Translation Planning at CIHR: Integrated and End-of-Grant Approaches. http://www.cihr-irsc.gc.ca/e/45321.html#a3. Accessed June 8, 2018.
45. Gillis C, Li C, Lee L, et al. Prehabilitation versus rehabilitation: a randomized control trial in patients undergoing colorectal resection for cancer. Anesthesiology. 2014;121(5):937-947.

doi:10.1097/ALN.0000000000000393.
46. Li C, Carli F, Lee L, et al. Impact of a trimodal prehabilitation program on functional recovery after colorectal cancer surgery: a pilot study. Surg Endosc. 2013;27(4):1072-1082. doi:10.1007/s00464012-2560-5.
47. Minnella EM, Awasthi R, Loiselle S-E, Agnihotram R V, Ferri LE, Carli F. Effect of Exercise and Nutrition Prehabilitation on Functional Capacity in Esophagogastric Cancer Surgery: A Randomized Clinical Trial. JAMA Surg. September 2018. doi:10.1001/jamasurg.2018.1645.
48. Tudor-Locke C, Craig CL, Aoyagi Y, et al. How many steps/day are enough? For older adults and special populations. Int J Behav Nutr Phys Act. 2011;8(1):80. doi:10.1186/1479-5868-8-80.
49. Stovitz SD, VanWormer JJ, Center BA, Bremer KL. Pedometers as a means to increase ambulatory activity for patients seen at a family medicine clinic. J Am Board Fam Pract. 18(5):335343. http://www.ncbi.nlm.nih.gov/pubmed/16148243.
50. World Health Organization. Global recommendations on physical activity for health 65 years and above. http://www.who.int/dietphysicalactivity/physical-activity-recommendations-65years.pdf?ua=1. Accessed July 17, 2018.
51. Vennix S, Pelzers L, Bouvy N, et al. Laparoscopic versus open total mesorectal excision for rectal cancer. Cochrane database Syst Rev. 2014;(4):CD005200. doi:10.1002/14651858.CD005200.pub3.
52. Fujii S, Tsukamoto M, Fukushima Y, et al. Systematic review of laparoscopic vs open surgery for colorectal cancer in elderly patients. World J Gastrointest Oncol. 2016;8(7):573-582. doi:10.4251/wjgo.v8.i7.573.
53. Ontario CC. Target Wait Times for Cancer Surgery in Ontario. https://www.cancercareontario.ca/en/guidelines-advice/types-of-cancer/3211. Accessed July 20, 2018.
54. NHS Cancer Plan. London, UK http://webarchive.nationalarchives.gov.uk/20130222181549/http://www.dh.gov.uk/prod_consum_d h/groups/dh_digitalassets/@dh/@en/documents/digitalasset/dh_4014513.pdf.
55. Redelmeier DA, Thiruchelvam D, Daneman N. Delirium after elective surgery among elderly patients taking statins. CMAJ. 2008;179(7):645-652. doi:10.1503/cmaj.080443.
56. Mitnitski a B, Mogilner a J, Rockwood K. Accumulation of deficits as a proxy measure of aging. ScientificWorldJournal. 2001;1:323-336. doi:10.1100/tsw.2001.58.
57. Grossman D, Rootenberg M, Perri G-A, et al. Enhancing communication in end-of-life care: a clinical tool translating between the Clinical Frailty Scale and the Palliative Performance Scale. J Am Geriatr Soc. 2014;62(8):1562-1567. doi:10.1111/jgs.12926.
58. Shears M, Takaoka A, Rochwerg B, et al. Assessing frailty in the intensive care unit: A reliability and validity study. J Crit Care. 2018;45:197-203. doi:10.1016/j.jcrc.2018.02.004.
59. Fried LP, Tangen CM, Walston J, et al. Frailty in Older Adults : Evidence for a Phenotype. J Gerontol Med Sci. 2001;56(3):146-157.
60. Bagshaw M, Majumdar SR, Rolfson DB, et al. A prospective multicenter cohort study of frailty in younger critically ill patients. Crit Care. 2016;20(1):175. doi:10.1186/s13054-016-1338-x.
61. Arroll B, Goodyear-Smith F, Crengle S, et al. Validation of PHQ-2 and PHQ-9 to Screen for Major Depression in the Primary Care. Ann Fam Med. 2010;8(4):348-354. doi:10.1370/afm.1139.INTRODUCTION.
62. Üstün TB, Chatterji S, Kostanjsek N, et al. Developing the World Health Organization Disability Assessment Schedule 2.0. Bull World Health Organ. 2010;88(11):815-823. doi:10.2471/BLT.09.067231.

63. EuroQoL Group. About EQ-5D. http://www.euroqol.org/about-eq-5d.html. Accessed October 13, 2016.
64. Galvin JE, Roe CM, Powlishta KK, et al. The AD8: A brief informant interview to detect dementia. Neurology. 2005;65(4):559-564. doi:10.1212/01.wnl.0000172958.95282.2a.
65. Hlatky MA, Boineau RE, Higginbotham MB, et al. A brief self-administered questionnaire to determine functional capacity (the Duke Activity Status Index). Am J Cardiol. 1989;64(10):651-654. http://www.ncbi.nlm.nih.gov/pubmed/2782256.
66. Goldberg A, Chavis M, Watkins J, Wilson T. The five-times-sit-to-stand test: validity, reliability and detectable change in older females. Aging Clin Exp Res. 2012;24(4):339-344. http://www.ncbi.nlm.nih.gov/pubmed/23238309.
67. Zhang, F., Ferrucci, L., Culham, E., Metter, E. J., Guralnik, J., & Deshpande, N. (2013). Performance on Five Times Sit-to-Stand Task as a Predictor of Subsequent Falls and Disability in Older Persons. *Journal of Aging and Health*, *25*(3), 478–492. https://doi.org/10.1177/0898264313475813
68. Grocott MPW, Browne JP, Van der Meulen J, et al. The Postoperative Morbidity Survey was validated and used to describe morbidity after major surgery. J Clin Epidemiol. 2007;60(9):919-928. doi:10.1016/j.jclinepi.2006.12.003.
69. Bennett-Guerrero E, Welsby I, Dunn TJ, et al. The use of a postoperative morbidity survey to evaluate patients with prolonged hospitalization after routine, moderate-risk, elective surgery. Anesth Analg. 1999;89(2):514-519. doi:10.1213/00000539-199908000-00050.
70. Davies SJ, Francis J, Dilley J, Wilson RJT, Howell SJ, Allgar V. Measuring outcomes after major abdominal surgery during hospitalization: reliability and validity of the Postoperative Morbidity Survey. Perioper Med (London, England). 2013;2(1):1. doi:10.1186/2047-0525-2-1.
71. Goodman BA, Batterham AM, Kothmann E, et al. Validity of the Postoperative Morbidity Survey after abdominal aortic aneurysm repair-a prospective observational study. Perioper Med (London, England). 2015;4:10. doi:10.1186/s13741-015-0020-1.
72. Wijeysundera DN, Pearse RM, Shulman MA, et al. Assessment of functional capacity before major non-cardiac surgery: an international, prospective cohort study. Lancet (London, England). 2018;391(10140):2631-2640. doi:10.1016/S0140-6736(18)31131-0.
73. Gilhooly DA, Cole M, Moonesinghe SR. The evaluation of risk prediction models in predicting outcomes after bariatric surgery: a prospective observational cohort pilot study. Perioper Med (London, England). 2018;7:6. doi:10.1186/s13741-018-0088-5.
74. McIsaac DI, Taljaard M, Bryson GL, et al. Comparative assessment of two frailty instruments for risk-stratification in elderly surgical patients: study protocol for a prospective cohort study. BMC Anesthesiol. 2016;16(1):111. doi:10.1186/s12871-016-0276-0.
75. Woodfield J, Zacharias M, Wilson G, et al. Protocol, and practical challenges, for a randomised controlled trial comparing the impact of high intensity interval training against standard care before major abdominal surgery: study protocol for a randomised controlled trial. Trials. 2018;19(1):331. doi:10.1186/s13063-018-2701-9.
76. Martos-Benítez FD, Gutiérrez-Noyola A, Echevarría-Víctores A. Postoperative complications and clinical outcomes among patients undergoing thoracic and gastrointestinal cancer surgery: A prospective cohort study. Rev Bras Ter intensiva. 28(1):40-48. doi:10.5935/0103-507X.20160012.
77. Patel ABU, Reyes A, Ackland GL. Non-inferiority of retrospective data collection for assessing perioperative morbidity. PeerJ. 2015;3:e1466. doi:10.7717/peerj.1466.
78. Richards T, Clevenger B, Keidan J, et al. PREVENTT: preoperative intravenous iron to treat anaemia in major surgery: study protocol for a randomised controlled trial. Trials. 2015;16:254. doi:10.1186/s13063-015-0774-2.

79. Kasivisvanathan R, Abbassi-Ghadi N, McLeod ADM, et al. Cardiopulmonary exercise testing for predicting postoperative morbidity in patients undergoing hepatic resection surgery. HPB (Oxford). 2015;17(7):637-643. doi:10.1111/hpb.12420.

80. Moonesinghe SR, Harris S, Mythen MG, et al. Survival after postoperative morbidity: a longitudinal observational cohort study. Br J Anaesth. 2014;113(6):977-984. doi:10.1093/bja/aeu224.

81. Dindo D, Demartines N, Clavien P-A. Classification of surgical complications: a new proposal with evaluation in a cohort of 6336 patients and results of a survey. Ann Surg. 2004;240(2):205-213. doi:10.1097/01.sla.0000133083.54934.ae.

82. Maggiori L, Rullier E, Lefevre JH, et al. Does a Combination of Laparoscopic Approach and Full Fast Track Multimodal Management Decrease Postoperative Morbidity?: A Multicenter Randomized Controlled Trial. Ann Surg. 2017;266(5):729-737. doi:10.1097/SLA.0000000000002394.

83. Palanivelu C, Senthilnathan P, Sabnis SC, et al. Randomized clinical trial of laparoscopic versus open pancreatoduodenectomy for periampullary tumours. Br J Surg. 2017;104(11):1443-1450. doi:10.1002/bjs.10662.

84. Kong S-H, Lee H-J, Na J-R, et al. Effect of perioperative oral nutritional supplementation in malnourished patients who undergo gastrectomy: A prospective randomized trial. Surgery. July 2018. doi:10.1016/j.surg.2018.05.017.

85. Lucot J-P, Cosson M, Bader G, et al. Safety of Vaginal Mesh Surgery Versus Laparoscopic Mesh Sacropexy for Cystocele Repair: Results of the Prosthetic Pelvic Floor Repair Randomized Controlled Trial. Eur Urol. 2018;74(2):167-176. doi:10.1016/j.eururo.2018.01.044.

86. Merki-Künzli C, Kerstan-Huber M, Switalla D, et al. Assessing the Value of Prehabilitation in Patients Undergoing Colorectal Surgery According to the Enhanced Recovery After Surgery (ERAS) Pathway for the Improvement of Postoperative Outcomes: Protocol for a Randomized Controlled Trial. JMIR Res Protoc. 2017;6(10):e199. doi:10.2196/resprot.7972.

87. Schultz JK, Yaqub S, Wallon C, et al. Laparoscopic Lavage vs Primary Resection for Acute Perforated Diverticulitis. JAMA. 2015;314(13):1364. doi:10.1001/jama.2015.12076.

88. Mitropoulos D, Artibani W, Biyani CS, Bjerggaard Jensen J, Rouprêt M, Truss M. Validation of the Clavien-Dindo Grading System in Urology by the European Association of Urology Guidelines Ad Hoc Panel. Eur Urol Focus. March 2017. doi:10.1016/j.euf.2017.02.014.

89. Téoule P, Bartel F, Birgin E, Rückert F, Wilhelm TJ. The Clavien-Dindo Classification in Pancreatic Surgery: A Clinical and Economic Validation. J Invest Surg. January 2018:1-7. doi:10.1080/08941939.2017.1420837.

90. Moonesinghe SR, Grocott MPW, Bennett-Guerrero E, et al. American Society for Enhanced Recovery (ASER) and Perioperative Quality Initiative (POQI) joint consensus statement on measurement to maintain and improve quality of enhanced recovery pathways for elective colorectal surgery. Perioper Med. 2017;6(1):6. doi:10.1186/s13741-017-0062-7.

91. WHO Disability Assessment Schedule 2.0 (WHODAS 2.0). World Health Organization: WHODAS 2.0. http://www.who.int/classifications/icf/whodasii/en/. Published 2014. Accessed April 13, 2014.

92. Shulman MA, Myles PS, Chan MT V., McIlroy DR, Wallace S, Ponsford J. Measurement of Disability-free Survival after Surgery. Anesthesiology. 2015;122(3):524-536. doi:10.1097/ALN.0000000000000586.

93. Wolf AC De, Tate RL, Lannin NA, Middleton J, Lane-brown A, Cameron ID. The World Health Organization Disability Assessment Scale , WHODAS II : reliability and validity in the

measurement of activity and participation in a spinal cord injury population. J Rehabil Med. 2012;44(9):747-755. doi:10.2340/16501977-1016.

94. Schlote A, Richter M, Wunderlich MT, et al. WHODAS II with people after stroke and their relatives. Disabil Rehabil. 2009;31(11):855-864. doi:10.1080/09638280802355262.

95. Wolf A, Tate R, Lannin N, Middleton J, Lane-Brown A, Cameron I. The World Health Organization Disability Assessment Scale, WHODAS II: Reliability and validity in the measurement of activity and participation in a spinal cord injury population. J Rehabil Med. 2012;44(9):747-755. doi:10.2340/16501977-1016.

96. Kutlay Ş, Küçükdeveci AA, Elhan AH, Öztuna D, Koç N, Tennant A. Validation of the World Health Organization disability assessment schedule II (WHODAS-II) in patients with osteoarthritis. Rheumatol Int. 2011;31(3):339-346. doi:10.1007/s00296-009-1306-8.

97. Garin O, Ayuso-mateos JL, Almansa J, et al. Validation of the " World Health Organization Disability Assessment Schedule , WHODAS-2 " in patients with chronic diseases. Health Qual Life Outcomes. 2010;8(1):51.

98. Küçükdeveci AA, Kutlay Ş, Yıldızlar D, Öztuna D, Elhan AH, Tennant A. The reliability and validity of the World Health Organization Disability Assessment Schedule (WHODAS-II) in stroke. Disabil Rehabil. 2012;35(May 2012):1-7. doi:10.3109/09638288.2012.690817.

99. Soberg HL, Finset A, Roise O, Bautz-Holter E. The trajectory of physical and mental health from injury to 5 years after multiple trauma: A prospective, longitudinal cohort study. Arch Phys Med Rehabil. 2012;93(5):765-774. doi:10.1016/j.apmr.2011.08.050.

100. Takahashi T, Kumamaru M, Jenkins S, Saitoh M, Morisawa T, Matsuda H. In-patient step count predicts re-hospitalization after cardiac surgery. J Cardiol. 2015;66(4):286-291. doi:10.1016/j.jjcc.2015.01.006.

101. Fisher SR, Graham JE, Ottenbacher KJ, Deer R, Ostir G V. Inpatient Walking Activity to Predict Readmission in Older Adults. Arch Phys Med Rehabil. 2016;97(9 Suppl):S226-31. doi:10.1016/j.apmr.2015.09.029.

102. Low CA, Bovbjerg DH, Ahrendt S, et al. Fitbit step counts during inpatient recovery from cancer surgery as a predictor of readmission. Ann Behav Med. 2018;52(1):88-92. doi:10.1093/abm/kax022.

103. Applebaum E V., Breton D, Feng ZW, et al. Modified 30-second Sit to Stand test predicts falls in a cohort of institutionalized older veterans. Bowen M, ed. PLoS One. 2017;12(5):e0176946. doi:10.1371/journal.pone.0176946.

104. Bansback N, Tsuchiya A, Brazier J, Anis A. Canadian Valuation of EQ-5D Health States : Preliminary Value Set and Considerations for Future Valuation Studies. PLoS One. 2012;7(2). doi:10.1371/journal.pone.0031115.

105. Canadian Institute for Health Information (CIHI). Patient reported outcome measures. https://www.cihi.ca/en/patient-reported-outcome-measures. Accessed June 7, 2018.

106. Wodchis W, Bushmeneva K, Nikitovic M, McKillop I. Guidelines on Person-Level Costing Using Administrative Databases in Ontario. Toronto, ON; 2013. http://www.hsprn.ca/uploads/files/Guidelines_on_PersonLevel_Costing_May_2013.pdf.

107. Juurlink DN, Croxford R, Chong A, Austin P, Tu J, Laupacis A. Canadian Institute for Health Information Discharge Abstract Database : A Validation Study ICES Investigative Report June 2006 Canadian Institute for Health Information Discharge Abstract Database :; 2006.

108. Atkins L, Francis J, Islam R, et al. A guide to using the Theoretical Domains Framework of behaviour change to investigate implementation problems. Implement Sci. 2017;12(1):1-18. doi:10.1186/s13012-017-0605-9.

109. Huijg JM, Gebhardt WA, Crone MR, Dusseldorp E, Presseau J. Discriminant content validity of a theoretical domains framework questionnaire for use in implementation research. Implement Sci. 2014;9(1):1-16. doi:10.1186/1748-5908-9-11.

110. Fergusson D, Aaron SD, Guyatt G, Hébert P. Post-randomisation exclusions: the intention to treat principle and excluding patients from analysis. BMJ. 2002;325(7365):652-654. http://www.ncbi.nlm.nih.gov/pubmed/12242181.
111. Ross S, Grant A, Counsell C, Gillespie W, Russell I, Prescott R. Barriers to participation in randomised controlled trials: a systematic review. J Clin Epidemiol. 1999;52(12):1143-1156. http://www.ncbi.nlm.nih.gov/pubmed/10580777.
112. Kelley GA, Kelley KS. Dropouts and Compliance in Exercise Interventions Targeting Bone Mineral Density in Adults: A Meta-Analysis of Randomized Controlled Trials. J Osteoporos. 2013;2013:1-19. doi:10.1155/2013/250423.
113. McComb A, Warkentin LM, McNeely ML, Khadaroo RG. Development of a reconditioning program for elderly abdominal surgery patients: the Elder-friendly Approaches to the Surgical Environment-BEdside reconditioning for Functional ImprovemenTs (EASE-BE FIT) pilot study. World J Emerg Surg. 2018;13:21. doi:10.1186/s13017-018-0180-7.
114. Zou G. A Modified Poisson Regression Approach to Prospective Studies with Binary Data. Am J Epidemiol. 2004;159(7):702-706. doi:10.1093/aje/kwh090.
115. Austin PC, Laupacis A. A tutorial on methods to estimating clinically and policy-meaningful measures of treatment effects in prospective observational studies: a review. Int J Biostat. 2011;7(1):6. doi:10.2202/1557-4679.1285.
116. Kahan BC, Jairath V, Doré CJ, Morris TP. The risks and rewards of covariate adjustment in randomized trials: an assessment of 12 outcomes from 8 studies. Trials. 2014;15(1):139. doi:10.1186/1745-6215-15-139.
117. Hernández A V, Steyerberg EW, Habbema JDF. Covariate adjustment in randomized controlled trials with dichotomous outcomes increases statistical power and reduces sample size requirements.
J Clin Epidemiol. 2004;57(5):454-460. doi:10.1016/j.jclinepi.2003.09.014.
118. Thompson DD, Lingsma HF, Whiteley WN, Murray GD, Steyerberg EW. Covariate adjustment had similar benefits in small and large randomized controlled trials. J Clin Epidemiol. 2015;68(9):1068-1075. doi:10.1016/j.jclinepi.2014.11.001.
119. Groenwold RHH, Moons KGM, Vandenbroucke JP. Randomized trials with missing outcome data: How to analyze and what to report. Cmaj. 2014;186(15):1153-1157. doi:10.1503/cmaj.131353.
120. Groenwold RHH, Donders ART, Roes KCB, Harrell FE, Moons KGM. Dealing with missing outcome data in randomized trials and observational studies. Am J Epidemiol. 2012;175(3):210-
217. doi:10.1093/aje/kwr302.
121. Vickers AJ, Altman DG. Statistics Notes: Analysing controlled trials with baseline and follow up measurements. BMJ. 2001;323(7321):1123-1124. doi:10.1136/bmj.323.7321.1123.
122. Austin PC, Ghali WA, Tu J V. A comparison of several regression models for analysing cost of CABG surgery. Stat Med. 2003;22:2799-2815. doi:10.1002/sim.1442.
123. Bilimoria KY, Liu Y, Paruch JL, et al. Development and evaluation of the universal ACS NSQIP surgical risk calculator: a decision aid and informed consent tool for patients and surgeons. J Am Coll Surg. 2013;217(5):833-42.e1-3. doi:10.1016/j.jamcollsurg.2013.07.385.

APPENDIX – Potential Adverse Events (AEs)

Surgical complications, extended length of stay, readmission and death are all possible risks of surgery.

We will track (S)AEs during data collection of physical measurements (5TSTS) (baseline and discharge visit/phone call), and during the time from enrollment to surgery (prehabilitation period).

Below is a list of possible expected adverse events that could occur during the different stages of The PREPARE Trial (baseline, prehabilitation program, and discharge visit/phone call). If participants report any of the below discomforts or symptoms, or any other (S)AEs during these timepoints, the research study staff will document it in the (S)AE Log, and will notify the site-investigator immediately. For example, all participant deaths occurring between enrollment and date of surgery will be documented and assessed by the site investigator. Further, all falls occurring during or within 24 hours after exercise (intervention arm participants only) will also be documented and assessed. If the site-investigator concludes that the event is deemed serious, unexpected and may be associated with participation in the study, they shall notify the Coordinating Centre and follow their REB Board of Record's SAE reporting guidelines. Please note that participating sites seeking REB approval through CTO Stream must follow the Board of Record's SOPs (i.e., OHSN-REB's SOPs).

Baseline Assessment & Outcome Assessment: Physical assessment (5TSTS)

Adverse Event	Preventative measures taken	If AE occurs, Action plan:
Light-headed	- Ask questions like, did you drink water today? Have you eaten in the last couple hours? How do you feel about briefly walking today? Would you feel up for it?<br>- Explained that certain symptoms are abnormal and if they start feeling light headed, faint, dizzy to let us know	- Stop test and if in person, get participant chair and glass of water, assess severity of situation, act accordingly
Dizzy	- Explained that certain symptoms are abnormal and if	- If in-person, help the participant sit down, get water, assess situation and act accordingly

	they start feeling light headed, faint, dizzy to let us know	
Mild shortness of breath	- Let the participant know that a little shortness in breath is normal<br>- Ask if participant has asthma, ask if they feel they would be able to complete the 5TSTS test	- If very severe shortness of breath, stop test<br>- Assess severity of situation and act accordingly

During Prehabilitation Program (at home)

- Although unlikely, an adverse event may occur during their home-based trainings (i.e. walking, performing their resistance training with their theraband or during their flexibility training)

Adverse Event	Preventative measures taken
Light-headed	- Patient should be encouraged by study staff to make sure they are eating properly and drinking lots of water<br>- Urged to contact study team if unsure about symptoms experiencing<br>- Explained that if experiencing any abnormal symptoms, stop exercising, evaluate how they feel, evaluate if it is normal, given number if have any questions, comments or concerns
Dizzy	- Participant is explained that if feelings of dizziness occurs, to stop program, contact study coordinators, if severe, talk with doctor
Mild shortness of breath	- Participant is explained that if shortness of breath occurs, to stop program, contact study coordinators, if severe, talk with doctor
Mild muscle pain	- Participants will be reminded to stretch and to only engage in exercises that feel right for them. Modifications will be provided.

Comorbidities and Cancer-Related Side Effects

Participants enrolled in the trial are frail older adults who may experience symptoms resulting from their pre-existing health condition(s), including cancer diagnoses. Such comorbidities and cancer-related side effects (including side effects from cancer-related treatment), would be expected for this population and not a result of their participation in the prehabilitation program or the trial overall. As with all possible SAEs, events that may be deemed serious, unexpected and/or may be associated with participation in the study will be documented and assessed by the site-investigator.

Protocol Summary of Changes

**Protocol Number**<br>*Version Date*	**REB Approval Date**	**Summary of Changes**
**Protocol 1.0**<br>*November 26, 2019*	December 2, 2019	- New document
**Protocol 1.1**<br>*December 3, 2019*	January 21, 2020	- Change in data collection time points for the Post-Operative Morbidity Survey (POMS)<br>- Time limit was placed on in-hospital data collection
**Protocol 1.2**<br>*September 2, 2020*	September 2, 2020	- Addition of remote recruitment as needed<br>- Changes to data collection due to remote visits as needed
**Protocol 1.3**<br>*July 1, 2021*	July 21, 2021	- Addition of site investigators (Drs. Serrano, Nelson and Srinathan)<br>- Study objectives and inclusion/exclusion criteria clarified throughout<br>- Addition of new cardio videos (standing and seated options) for intervention participants<br>- All intervention participants to receive nutrition pamphlet<br>- Exercise and adherence support provided by coordinating centre clarified to better reflect PREPARE Program approach<br>- COVID-19 status to be collected at all timepoints<br>- Study AE definitions clarified<br>- Program adherence and planned analysis defined<br>- Data collection and analysis plan for "Day 84" participants defined<br>- Follow-up windows included for all data collection timepoints<br>- Additional appendices (questionnaires, data collection tools, etc.) removed from document for ease of REB amendments/review
**Protocol 1.4**<br>*November 11, 2021*	November 25, 2021	- For patients scheduled for surgery outside of the recruiting institution, only patient-reported data will be collected for the in-hospital follow-up (postoperative days 3, 5, and 7 and discharge). Data collected by chart review will be documented as missing.
**Protocol 1.5**<br>*April 26, 2022*	May 3, 2022	- The processes for obtaining written informed consent and verbal informed consent have been clarified. Study procedures may begin as soon as a participant provides their written consent or their verbal consent to participate. When obtaining written consent, a photocopy of the

		fully signed Main Informed Consent Form will be provided to the participant for their records. When obtaining verbal consent, a copy of the Main Information Sheet (no signature pages) will be provided to the participant for their records by email or by mail.
**Protocol 1.6**<br>*January 30, 2023*	June 19, 2023	- Language has been added to the adverse events appendix to clarify that symptoms related to participants' pre-existing health conditions (i.e., comorbidities, side effects of cancer treatment, etc.) are expected for this population. The experience of such comorbidities and side effects are not considered to be a result of their participation in the prehabilitation program or trial overall.
**Protocol 1.7**<br>*August 28, 2023*	October 26, 2023	- Sample size has been increased to 850 participants.

Statistical Analysis Plan

1 Administrative Information

Date	October 31, 2024
Study Title	PREPARE Trial: a parallel arm multicentre randomised trial of frailty-focused preoperative exercise to decrease postoperative complication rates and disability scores
Study Registration Number	NCT04221295
SAP Version Number	2
Protocol Version and Date	1.7, August 28, 2023
Trial Statistician	Dr. Monica Taljaard
Trial Principal Investigator	Dr. Daniel McIsaac
SAP Author(s)	Caroline Lee

Revision Control

Protocol Version	Updated SAP version number	Section number changed	Description of change	Date changed
1.7	2	9.4	Removed step count from imputation model as the value was highly missing due to poor pedometer function amongst older adults.	2024/10/31; prior to any outcome analyses
1.7	2	9.4	Removed consent method from the imputation model to reflect pre-specified removal of consent method as a stratification factor due to COVID-19 related changes to telephone vs. in-person consent	2024/10/31; prior to any outcome analyses

Roles and Responsibilities

Name	Role	Institution
Dr. Daniel McIsaac	Principal Investigator	University of Ottawa
Dr. Dean Fergusson	Lead Epidemiologist	Ottawa Hospital Research Institute
Dr. Monica Taljaard	Lead Biostatistician	Ottawa Hospital Research Institute
Dr. Rachel Khadaroo	Lead Surgeon	University of Alberta
Dr. John Muscedere	Co-Principal Investigator and Knowledge User	Queen's University
Amanda Meliambro	Co-Principal Investigator and Patient Partner	Memorial University of Newfoundland

2 SAP Signatures

I give my approval for the attached SAP entitled PREPARE TRIAL dated October 31st, 2024

Statistician (Author)

Name: Monica Taljaard

Signature: ______________________

Date: 11/25/2024

Statistician Reviewer (As applicable)

Name:

Signature: ______________________

Date: ______________________

Principal Investigator

Name: Daniel I McIsaac

Signature: ______________________

Date: 2024/11/12

3 Table of Contents

1 Administrative Information 1
2 SAP Signatures 2
3 Table of Contents 3
4 Abbreviations and Definitions 5
5 Introduction 5
6 Study Objectives, Endpoints, and Estimands 6
6.1 Study Objectives 6
6.2 Endpoints 6
6.3 Intercurrent Events 8
6.4 Target Estimands 9
7 Study Methods 9
7.1 General Study Design and Plan 9
7.2 Inclusion-Exclusion Criteria and General Study Population 11
7.3 Randomization and Blinding 11
7.4 Study Assessments 11
8 Sample Size 12
9 General Analysis Considerations 13

9.1 Timing of Analyses 13
9.2 Analysis Populations 13
9.2.1 Full Analysis Population 13
9.2.2 Per Protocol Population 14
9.2.3 Safety Population 14
9.3 Covariates and Subgroups 14
9.4 Missing Data 14
9.5 Interim Analyses and Data Monitoring (as applicable) 15
9.5.1 Purpose of Interim Analyses 15
9.5.2 Planned Schedule of Interim Analyses 15
9.5.3 Scope of Adaptations 15
9.5.4 Stopping Rules 15
9.5.5 Adjustment of Confidence Intervals and P-values 15
10 Summary of Study Data 16
10.1 Subject Disposition 16
10.2 Derived variables 17
10.3 Protocol Deviations 17
10.4 Demographic and Baseline Variables 17
10.5 Treatment Compliance 18
11 Effectiveness Analyses 19
11.1 Primary Effectiveness Analysis 19
11.2 Secondary Effectiveness Analyses 19
11.2.1 Secondary Analyses of Primary Effectiveness Endpoint 19
11.2.2 Analyses of Secondary Endpoints 19
11.3 Exploratory Effectiveness Analyses 20
12 Safety Analyses 20
13 Cost-Effectiveness Data 21
14 Reporting Conventions 21
15 Summary of Changes to the Protocol and/or SAP 21
16 References 21
17 Listing of Tables and Figures 24

4 Abbreviations and Definitions

5TSTS	5-Times-Sit-To-Stand
AD8	8-item Informant Interview to Differentiate Aging and Dementia
CFS	Clinical Frailty Scale
CI	Confidence Interval
CNST	Canadian Nutrition Screening Tool
DASI	Duke Activity Status Index
DIMR	Alberta-Alberta Data Integration, Measurement & Reporting
DSMB	Data Safety Monitoring Board
EQ-5D-5L	5-level EuroQoL health related quality of life
KATZ	Katz Index of Independence in Activities of Daily Living
HRQoL	Health-related Quality of Life
ICES	Institute of Clinical Evaluative Sciences
ITT	Intention-to-Treat
LoS	Length of Stay
MCHP	Manitoba-Manitoba Center for Health Policy
PHQ	Personal Health Questionnaire
POMS	Postoperative Morbidity Questionnaire
SD	Standard Deviation
WHODAS	World Health Organization Disability Assessment Schedule 2.0

5 Introduction

Frailty is a multidimensional state of vulnerability arising from age- and disease-related deficits accumulating throughout a patient's life[1–5]. In cases where frailty is present prior to surgery, the rates of new patient-reported disability, major complications, hospital readmission, non-home discharge, and death increase by more than twofold[1,6–11]. Improving physical and physiological status prior to surgery could mitigate adverse postoperative outcomes for older individuals with frailty[11,12]. One approach to enhance patients' physical and physiological well-being is prehabilitation, which actively readies patients for surgery using exercise, nutrition, psychocognitive interventions or a combination of these methods[13]. Recent research shows that prehabilitation could offer advantages in terms of reducing complication rates, decreasing non-home discharge, shortening length of stay, and promoting functional recovery[13]. However, there have been few perioperative trials that include older people with frailty[14,15]. Furthermore, the existing trials have relatively small sample sizes and are single centre[14,15]. Therefore, addressing knowledge gaps through a multicenter trial specifically focused on individuals with frailty is required to evaluate the effectiveness of home-based prehabilitation in reducing patient-reported disability and postoperative complications. [13].

6 Study Objectives, Endpoints, and Estimands

6.1 Study Objectives

This trial was designed to evaluate the effectiveness of a home-based multimodal prehabilitation intervention in decreasing patient-reported disability and postoperative complications in older people with frailty having major surgery. The intervention consists of a structured, home-based, multimodal prehabilitation program that provides personalized exercise and nutritional recommendations. The exercise regimen entails 1-hour sessions three times a week for at least three weeks, incorporating strength training, aerobic exercises, and flexibility exercises. Participants will receive a telephone-assisted education session on the exercise program and are further supported by an experienced central team that will conduct weekly phone calls to ensure safety, encourage adherence, and provide guidance on exercise progression. Participants in the control group receive static activity and healthy eating recommendations but do not receive active support, logs, or regular contact.

The primary objective is to determine if the prehabilitation program will reduce patient-reported disability 30-days after surgery and/or in-hospital complications. The secondary objectives will assess the effectiveness of the program on patient-centered outcomes (i.e., discharge home, survival, 1-year disability scores, quality of life, frailty, function) and system-relevant outcomes (i.e., length of stay readmissions). An additional objective of this trial relates to evaluating cost-effectiveness of the program.

6.2 Endpoints

The trial has two primary outcomes, defined at the patient-level: an index of patient-reported disability at 30 days after surgery (continuous) and presence of any in-hospital complications (binary). These outcomes were selected based on priorities for older surgical patients, proposed causal mechanisms between exercise prehabilitation and outcomes, and systematic review efficacy data.

The continuous co-primary outcome, patient reported disability 30 days after surgery, will be measured using the World Health Organization Disability Assessment Schedule 2.0

(WHODAS)[1]. The WHODAS is a patient-reported disability scale that assesses limitations in six major life domains, namely cognition, mobility, self-care, social interaction, life activities, and participation in society. The WHODAS contains questionnaire items that are scored on a Likert scale from 0 to 4. The WHODAS Disability Score ranges from 0 to 48 and is expressed as a percentage (0 to 100) of the maximum possible score. If a participant dies before follow-up, they will be considered completely disabled (i.e., assigned a score of 100).

In-hospital complications will be measured using the Postoperative Morbidity Questionnaire (POMS), which is a prospectively administered instrument designed to identify significant in-hospital complications in key organ systems[1]. The POMS consists of 18 items addressing nine domains: pulmonary, infectious, renal, gastrointestinal, cardiovascular, neurological, hematological, and wound pain; the pain domain will not be included in the PREPARE study composite definition as pre-specified based on causal mechanisms. The POMS uses medical charts, medications records, vital signs records, routine lab tests, and direct questioning of the patient to determine the presence or absence of a complication. Any POMS complication or death in hospital will be recorded as a complication (i.e., a composite outcome).

The secondary outcomes are reflected by the following five domains: (1) function, (2) health-related quality of life, (3) all-cause mortality, (4) health system outcomes, (5) safety.

Function will be measured using three different outcomes: (1) total step counts, (2) Five-Times sit to stand test (5TSTS), and (3) the Katz Index. Step count will be recorded daily for 30 days after surgery, using a pedometer, and will be expressed as a daily average over all available days. The 5TSTS measures the lower-extremity strength and balance of a patient[16]. The Katz Index measures the independence level of daily living activities, on a scale from 0 to 6, where 0 indicates the highest level of dependence and 6 indicates the highest level of independence[17].

Health-related quality of life will be measured using the 5-level EuroQoL health related quality of life (EQ-5D-5L). The EQ-5D-5L measures quality of life on the following five dimensions: (1) mobility, (2) self-care, (3) usual activities, (4) pain/discomfort, and (5) anxiety/depression[18]. Postoperative pain will be captured from the pain domain of the POMS instrument.

All-cause mortality will be identified in-hospital or through telephone follow-up up at 30 days, 90 days, and 1-year post-surgery. The severity of in-hospital complications will also be captured using the Clavien-Dindo scale as an ordinal secondary outcome.

Discharge disposition (home, home with support, rehabilitation, long term care) will be prospectively collected at discharge by telephone. Where possible, trial data will be linked to repositories of routinely collected health data. Linkage to these databases will allow for collection of (1) health system costs, (2) readmissions, (3) emergency department visits, and (4) subsequent long-term care admissions in the year after surgery.

Lastly, patient safety will be measured through (1) falls, musculoskeletal (MSK) injury, and head injury, and (2) unplanned health encounters (emergency room visits and hospital admissions). This information will be measured during the post-randomization pre-surgery period. These measures will be collected in both arms. Additional safety measures that will only be measured in the intervention arm are new or worsened symptoms and adherence.

6.3 Intercurrent Events

Intercurrent events (ICEs) are events that occur after treatment that may affect the interpretation of the endpoints[19]. We anticipate and have accounted for six potential types of intercurrent events in our analysis plan: (1) patients who do not have a surgery performed during the trial (for a reason other than death), (2) patients who die post-randomization, but pre-surgery, (3) patients who die outside of hospital post-surgery, (4) patients who withdraw from treatment but still complete data collection, (5) patients who do not adhere to treatment but still complete data collection, and (6) patients who are lost to follow-up (do not complete data collection). There are five potential strategies for dealing with intercurrent events[19]: (1) *treatment policy strategy* in which the measured value of the outcome variable is used in the analysis regardless of occurrence of the intercurrent event (i.e., consistent with a traditional intention-to-treat approach), (2) *hypothetical strategy* in which the value of the outcome variable is estimated in the hypothetical scenario in which the intercurrent event would not have occurred, (3) *composite variable strategy* in which the intercurrent event is incorporated into the definition of the variable, (4) *while-on-treatment strategy* in which the response to treatment prior to occurrence of the intercurrent event is of interest, and (5) *principal stratum strategy* in which the target population is defined to be a "principal stratum" in which an intercurrent event would or would not occur. Our chosen strategy for dealing with each of these intercurrent events in the primary analysis for each co-primary outcome is summarized in the table.

PRIMARY ANALYSIS

**Intercurrent event**	**POMS**	**WHODAS at 30 days**
Do not have surgery performed for reason other than death	Exclude (*principal stratum strategy)* assuming the reason for not having surgery is unrelated to trial arm	Include (*treatment policy strategy*)
Die before surgery	Exclude (*principal stratum strategy)* assuming the reason for death before surgery is unrelated to trial arm	Include (*composite variable strategy*): assigned the worst possible outcome
Die outside of hospital after surgery	Not applicable: patients who are discharged from the hospital can no longer experience an in-hospital complication	Include (*composite variable strategy*): assigned the worst possible outcome
Withdraw from treatment	Include (*treatment policy strategy*)	Include (*treatment policy strategy*)
Do not adhere to treatment	Include (*treatment policy strategy*)	Include (*treatment policy strategy*)
Lost to follow-up or withdraw from trial	Include (*hypothetical strategy*)*:* Participants with missing data are included in the analysis using a multiple imputation model	Include *(hypothetical strategy):* Participants with missing data are included in the analysis using a multiple imputation model under the assumption that reasons for

	under the assumption that reasons for attrition are related only to observed data	attrition are related only to observed data

For the co-primary outcome of patient-reported disability at 30 days (WHODAS), all patients will be included in the analysis; thus, we will adopt the treatment policy strategy by using the measured WHODAS (at day 114 post-randomization) for patients who do not have surgery and those who withdraw from treatment. Patients who die outside of hospital post-randomisation will be considered fully disabled and assigned WHODAS scores of 100 (composite variable strategy).

For the co-primary outcome of in-hospital complications, patients who do not have surgery and patients who die post-randomisation but prior to having surgery cannot be considered to be in the risk set for a postoperative complication; therefore, these individuals will be excluded from the analysis. In addition, POMS will still be recorded for individuals who withdraw from the intervention or do not adhere to treatment (treatment policy strategy).

6.4 Target Estimands

The target estimand for the co-primary outcome of patient-reported disability is the mean difference in the WHODAS score at day 30 in patients with frailty planning to undergo elective inpatient surgery and allocated to the prehabilitation program versus those allocated to usual care.

The target estimand for the co-primary outcome of in-hospital complications is the relative odds of in-hospital complications for patients with frailty undergoing elective inpatient surgery and allocated to the prehabilitation program versus those allocated to usual care.

7 Study Methods

7.1 General Study Design and Plan

This study is a multicenter, parallel arm individually randomized controlled trial conducted across 11 Canadian community and academic hospitals, with the Ottawa Methods Centre serving as the coordinating center.

Participants will be recruited from surgery or anesthesia clinic lists by telephone. Eligible individuals who consent to research contact, are expected to undergo surgery in 3-12 weeks, and are 60 years or older will be assessed for frailty using the Clinical Frailty Scale (CFS). Those scoring ≥ 4/9 on the CFS will be given the opportunity to provide written or verbal informed consent to participate in the trial. Baseline data will be collected from all participants before randomization. Subsequently, participants will be randomly assigned to either the intervention or control group.

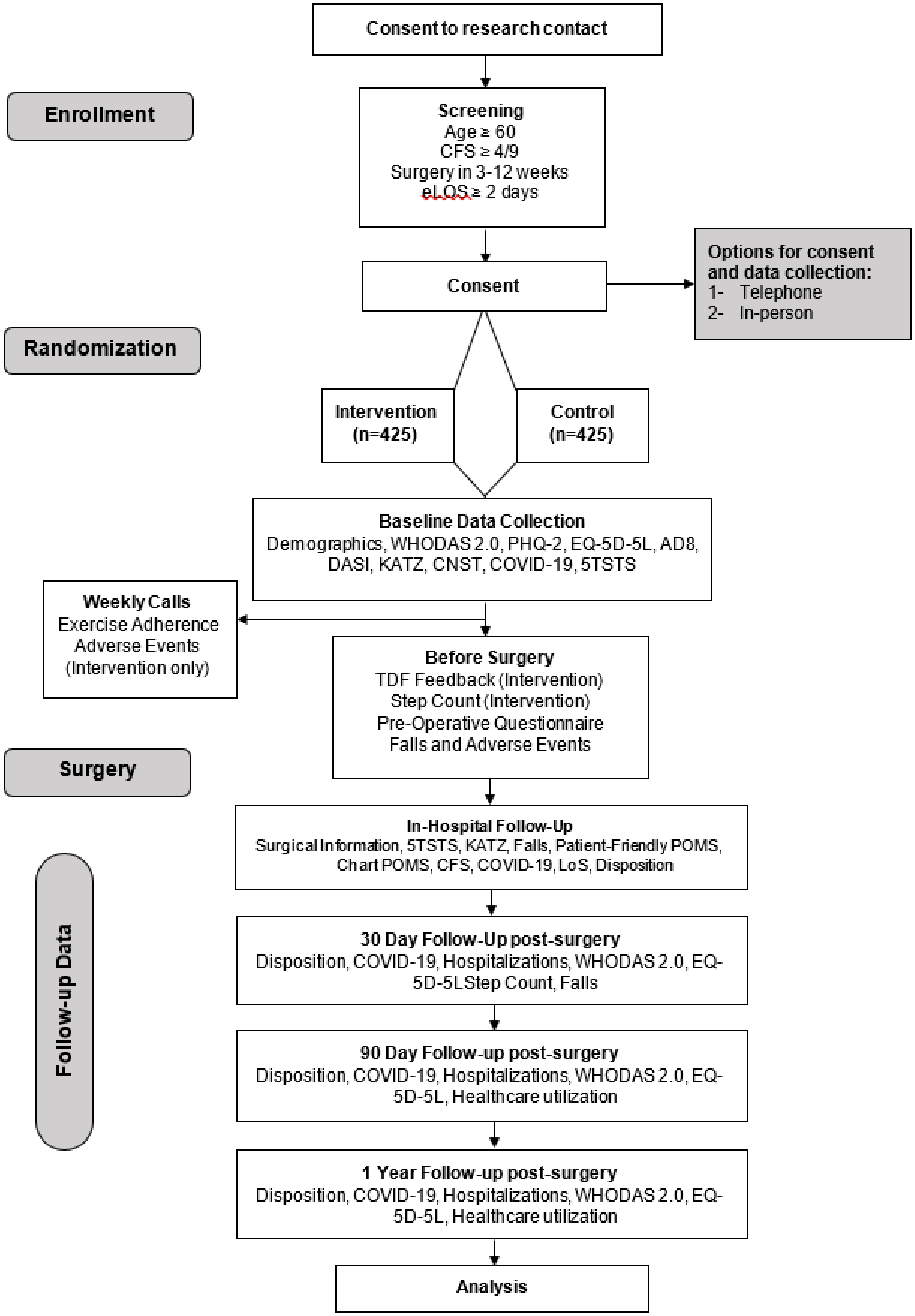
Consent to research contact
Enrollment
Screening
Age ≥ 60
CFS ≥ 4/9
Surgery in 3-12 weeks
eLOS ≥ 2 days
Consent
Options for consent and data collection:
1- Telephone
2- In-person
Randomization
Intervention (n=425)
Control (n=425)
Baseline Data Collection
Demographics, WHODAS 2.0, PHQ-2, EQ-5D-5L, AD8, DASI, KATZ, CNST, COVID-19, 5TSTS
Weekly Calls
Exercise Adherence
Adverse Events
(Intervention only)
Before Surgery
TDF Feedback (Intervention)
Step Count (Intervention)
Pre-Operative Questionnaire
Falls and Adverse Events
Surgery
In-Hospital Follow-Up
Surgical Information, 5TSTS, KATZ, Falls, Patient-Friendly POMS, Chart POMS, CFS, COVID-19, LoS, Disposition
Follow-up Data
30 Day Follow-Up post-surgery
Disposition, COVID-19, Hospitalizations, WHODAS 2.0, EQ-5D-5LStep Count, Falls
90 Day Follow-up post-surgery
Disposition, COVID-19, Hospitalizations, WHODAS 2.0, EQ-5D-5L, Healthcare utilization
1 Year Follow-up post-surgery
Disposition, COVID-19, Hospitalizations, WHODAS 2.0, EQ-5D-5L, Healthcare utilization
Analysis

7.2 Inclusion-Exclusion Criteria and General Study Population

The inclusion criteria of the study are: (1) participants aged ≥ 60 years, (2) elective inpatient surgery, (3) the expected surgery date is between 3-12 weeks from enrollment, and (4) a Clinical Frailty Scale (CFS) score greater or equal to 4/9.

The exclusion criteria of the study are: (1) the inability to speak English or French, (2) the presence of comorbidities preventing assessment or the ability to understand the questionnaires, (3) the inability to be contacted by telephone, (4) the unwillingness to participate in the exercise programme, (5) cardiac, neurological, or orthopaedic procedure, (6) palliative surgery, and (7) any of the following cardiovascular conditions: (i) severe valvular heart disease, (ii) severe cardiac dysrhythmias, or (iii) myocardial infraction within the 6 weeks prior to enrolment.

7.3 Randomization and Blinding

The allocation sequence will be computer-generated by an independent biostatistician using permuted blocks of randomly varying lengths, stratified by centre, cancer vs non-cancer surgery, and consent method (in-person vs telephone). The randomisation sequence will be accessed through a central secure internet-based application to ensure allocation concealment. After informed consent is obtained, the researcher will log onto the central secure internet-based application with a password, obtained by the Ottawa Methods Center.

The clinicians and outcome assessors will be fully blinded to potential treatment allocation groups. The participants will be partially blinded as they will be informed that they are being enrolled in a study to evaluate activity interventions before surgery.

7.4 Study Assessments

The study assessment schedule is provided in Table 1. The co-primary outcome of the WHODAS will be assessed at baseline, 30 days, 90 days, and 1-year post-surgery with 30 days being specified as the primary endpoint. The co-primary outcome POMS will be assessed postoperatively during the index surgical hospitalization.

The following baseline characteristics will be measured: demographics, Clinical Frailty Scale (CFS), Patient Health Questionnaire (PHQ-2), AD8 Cognitive Screen (AD8), Duke Activity Status Index (DASI), Canadian Nutrition Screening Tool (CNST), cancer status, neoadjuvant therapy status.

To assess intervention safety, falls and unplanned healthcare encounters will be identified during the intervention period but prior to surgery. During this period, prespecified intervention attributable events and intervention compliance will also be measured in the intervention arm.

The secondary outcome measuring function, the 5TSTS, will be measured at baseline and postoperatively at the time of hospital discharge. Additionally, step counts, which also measure function, will be assessed, and averaged over the available days in the 30 days post surgery period. Health-related quality of life, measured through the EQ-5D-5L, will be assessed at baseline, 30 days, 90 days, and 1 year post surgery. Discharge disposition, CFS and length of stay (LoS) will be measured postoperatively at hospital discharge. Postoperative falls will

be identified within 30 days post-surgery. Any deaths, and their date, will be identified up to 1-year post-surgery.

**Table 1. Timing of Study Assessments**							
**Domain**	**Assessment**	**Baseline**	**Surgery**	**Postop in-hospital**	**30 days postop**	**90 days postop**	**365 days postop**
Co-Primary Outcomes	WHODAS	X			X	X	X
	POMS			X			
Baseline characteristics	Demographics	X					
	CFS	X		X			
	PHQ-2	X					
	AD8	X					
	DASI	X					
	CNST	X					
	Cancer/Chemo	X					
	COVID-19	X			X	X	X
Secondary Outcome: Function	Step Counts				X		
	5TSTS	X		X			
	Katz	X		X			
Secondary Outcome: HRQoL	EQ-5D-5L	X			X	X	X
Secondary Outcome: All-cause mortality	Survival				X	X	X
Secondary Outcome: Health System	Disposition			X			
	LoS			X			
Secondary Outcome: Safety	Adjudicated Adverse Events		X				
	Falls			X	X	X	X
	Readmissions			X	X	X	X

8 Sample Size

The sample size is driven by the binary primary outcome of in-hospital post-operative complications (POMS). Our initial target sample size was 750 participants (375 participants in each arm). After publication of the protocol, the trial steering committee approved an increase in the target sample size to 850 participants (425 in each arm) to allow for the following assumptions:

1) A 55% complication rate among those undergoing surgery in the control arm, which is informed by data from our prior trial and related systematic reviews[20,21];
2) A 28% relative reduction in complications in the intervention arm among those undergoing surgery and being adherent to the intervention;
3) 15% non-adherence in the intervention arm (i.e., patients not doing any exercise);

4) 15% attrition, which accounts for patients not having surgery and 5% of patients being lost to follow-up.

With these assumptions, the target of 850 participants achieves 90% power to detect a relative difference of 23.8% in the intention to treat analysis (a control arm event rate of 0.55 versus an attenuated intervention arm event rate of 0.4191) using a pooled Z-test with a two-sided alpha of 0.025.

For the continuous co-primary outcome (WHODAS Disability Score), a sample size of 850 achieves 90% power to detect a difference of 5 points on a 100-point scale using an analysis of covariance (ANCOVA) at the two-sided alpha level of 0.025 in the intention to treat analysis. This difference is smaller than the minimum clinically important difference of 8 points used in the study protocol, and accounts for our conservative strategy of dealing with intercurrent events[22]. It is also in line with subsequent publications suggesting a minimally important difference of 5 specifically for surgical patients. We assumed a common SD of 20 (consistent with previous publications[20]), a correlation between baseline and postoperative score of 0.4, and accounted for 5% attrition. A Bonferroni correction was used to maintain the overall type I error rate across the two primary outcomes at 5%.

9 General Analysis Considerations

9.1 Timing of Analyses

The trial is due to finish once the 1-year follow-up post-surgery for the last recruited patient has been obtained. As the primary outcome data will be complete approximately 30-days after the last recruited patient has surgery, the data will be cleaned, verified, and locked for the initial analyses at 30 days after the last participant has surgery. Final analysis will commence once the final lock has been confirmed by the Principal Investigator.

9.2 Analysis Populations

9.2.1 Full Analysis Population

The full analysis population differs between the two co-primary outcomes. For WHODAS, the full analysis population will consist of all participants who were randomized (as prehabilitation can influence disability status regardless of having surgery). In the case of patients who receive the intervention, but do not undergo surgery, WHODAS will still be collected 114 days post-randomization. Furthermore, individuals who die post-randomization will be recorded as fully disabled on WHODAS (i.e., they will receive a score of 100). Therefore, this analysis population will consist of all participants who where randomized regardless of whether they received the intervention (i.e., intention to treat) or had surgery.

For POMS, the full analysis population will consist of individuals who are part of the risk set for postoperative complications (i.e., patients who were randomized and underwent surgery, as people who do not have surgery cannot be at risk of a postoperative complication). Participants who don’t have surgery or who die prior to surgery will not be included in the analysis. POMS will be recorded regardless of whether participants withdraw from treatment or do not adhere to the intervention.

9.2.2 Per Protocol Population

The per protocol analysis population will consist of all individuals in the control arm who had their planned surgery, and all individuals in the intervention arm who had their planned surgery and who completed > 75% of their prescribed exercise sessions.

9.2.3 Safety Population

The safety population will consist of all the study participants. Furthermore, there will be an additional intervention-attributable safety analysis performed only on patients who were allocated to the intervention.

9.3 Covariates and Subgroups

Primary and secondary analyses will be adjusted for stratification factors and postulated predictors of outcome (to enhance power). In the original study protocol, it was specified that randomization would be stratified on the consent method (in-person vs. telephone). However, due to the COVID-19 pandemic, and after only 20 patients had already been recruited in-person, the use of in-person consent was no longer permitted. For that reason, the analysis will only adjust for cancer versus non-cancer surgery (binary), and center (as a random intercept) as stratification factors.[23] Other covariates, postulated to be predictive of outcome that will be included in outcome regression models are age (continuous), sex (binary), malnutrition risk (binary), and frailty score (binary, 4 vs. $\geq$5 on the CFS).

Preplanned subgroup analyses for the two primary outcomes will be conducted based on sex, age (<75 vs ≥75), presence of cancer, presence of depression, and frailty status (4 vs ≥ 5). Subgroup analyses will be conducted using an effect modifier approach where the subgroup indicator will be tested as an interaction term with treatment allocation.

9.4 Missing Data

Since ITT analyses are being used in this trial, all eligible patients will be included. To account for missing data due to attrition, a multiple imputation model will be used to maintain power and attenuate missing data bias. The multiple imputation model will be used to impute missing outcome data for patients who withdrew entirely from the study or for individuals lost to follow up. Multiple imputation will not be used to assign outcome values for patients who have died, or who did not receive surgery. Instead, these intercurrent events will be managed using the strategies described in Section 6.3.

Prior to any statistical analysis being conducted, multiple imputation using the fully conditional specification method will be performed to create a dataset with no missing observations. In fully conditional specification multiple imputation, the missing variables are imputed by creating an imputation model for each missing variable, given the other available variables[24]. The multiple imputation model will include the following variables: (1) WHODAS, (2) POMS, (3) age, (4) sex, (5) surgery type, (6) malnutrition risk (CNST), (7) frailty score (CFS), (8) centre, (9) cancer surgery, (10) 5TSTS, (11) Katz Index, (12) EQ-5D-5L, (13) survival, (14) disposition, and (15) length of stay.

Since the primary and secondary outcomes are measured at multiple time points throughout the trial, the multiple imputation model will use all the data present until the participant withdrew from the trial. The multiple imputation analysis will be repeated at least 10 times (depending on the fraction of missing information) and Rubin's rule will be used to determine the average value for each missing value.

9.5 Interim Analyses and Data Monitoring (as applicable)

9.5.1 Purpose of Interim Analyses

An interim analysis for safety was completed and reviewed by the DSMB on August 9th, 2022, after enrollment of 50% of the sample size.

9.5.2 Planned Schedule of Interim Analyses

The interim safety analysis was performed on the safety population when they completed their 30-day follow up.

9.5.3 Scope of Adaptations

During the interim analysis, baseline data, compliance data, all outcome data related to complications, safety, and adverse events was collected, while masking the treatment allocation.

9.5.4 Stopping Rules

The DSMB recommended that the trial continue following the interim safety analysis.

9.5.5 Adjustment of Confidence Intervals and P-values

Since there are two primary outcomes and an effect on at least one of the outcomes is desired, a multiplicity adjustment is required. For both primary outcomes, a two-sided alpha value of 0.025 will be used for the hypothesis tests for the primary outcomes and 97.5% confidence intervals (CIs) will be reported. No adjustment will be applied for prespecified secondary outcomes, which will be reported using 95% CIs.

10 Summary of Study Data

10.1 Subject Disposition

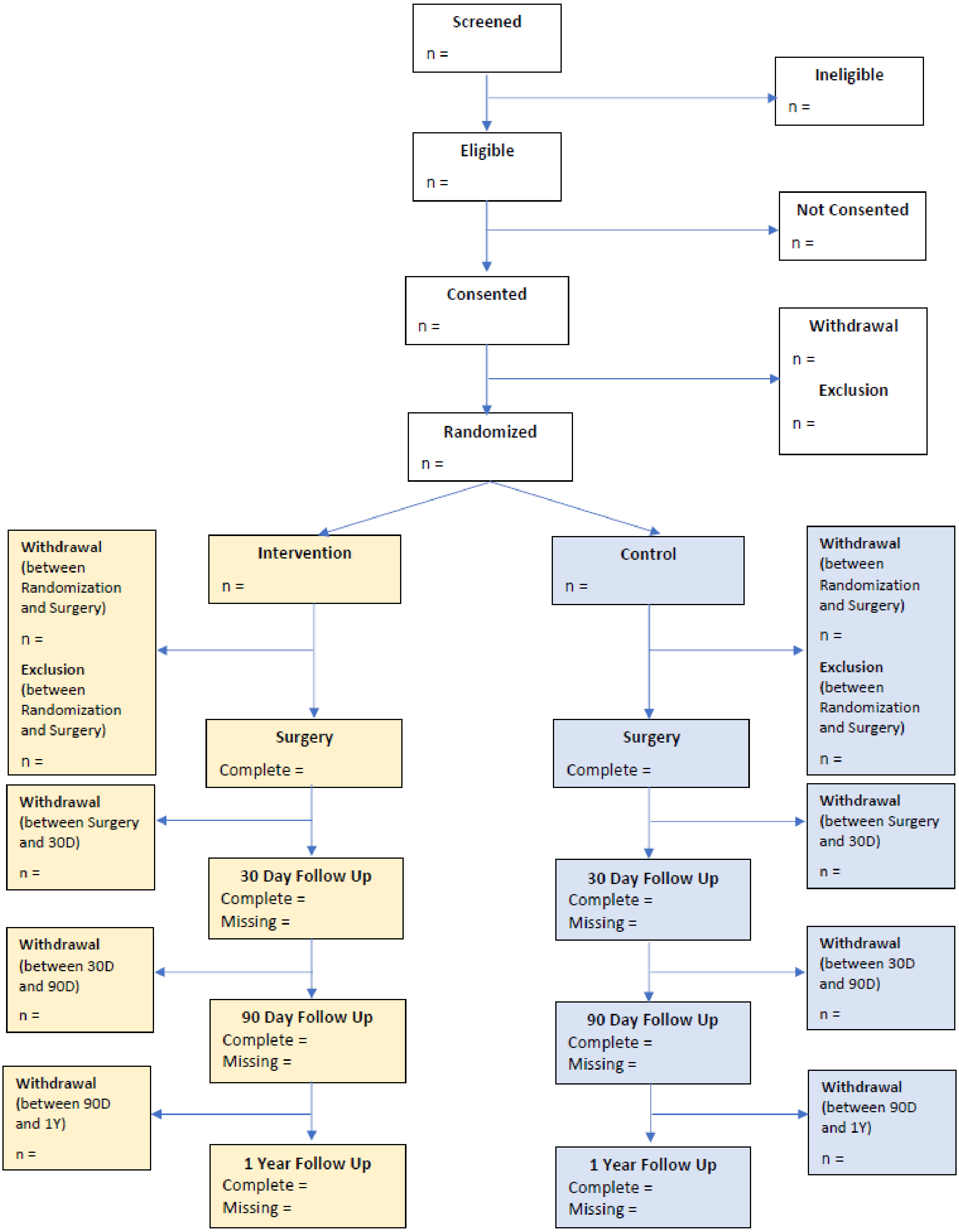

10.2 Derived variables

The following 12 components are measured to determine the WHODAS score: (1) standing for long periods such as 30 min, (2) taking care of household responsibilities, (3) learning a new task, for example learning how to get a new place, (4) how much of a problem have you had joining in community activities, (5) how much have you been emotionally affected by your recent surgery, (6) concentrating on doing something for 10 mins, (7) walking a long distance (like a kilometer), (8) washing your whole body independently, (9) getting dressed independently. (10) dealing with people you don't know, (11) maintaining a friendship, and (12) doing your day-to-day work. Each component is measured on a scale from 0 to 4 where 0=None, 1=Mild, 2=Moderate, 3=Severe, 4=Extreme or cannot do, and not applicable. This results in a range of WHODAS scores from 0 to 48, which is then presented as percentage of the maximum possible score (i.e., 0 to 100).

In addition, we will also record the answers to the following questions: (1) in the last 30 days, how many days were these difficulties present, (2) in the last 30 days, how many days were you totally unable to carry out your usual activities because of your health, (3) not counting the days you were unable, in the last 30 days how many days did you have to cut back or reduce your usual activities because of your health, and (4) How much of an impact did the COVID-19 pandemic have on your ability to perform activities described in the previous survey. The last question will be answered using the following scale: major impact, moderate impact, minor impact, no impact, and too soon to tell.

10.3 Protocol Deviations

The following protocol deviations could occur: wrong consent version used, data collected outside protocol defined time points, no surgery performed within 84 days of randomization, no program orientation for intervention participant (typically because surgery would be scheduled sooner than anticipated), participant unblinded for baseline data collection, surgery less than 3 weeks from enrollment, wrong version of materials sent to participants, the randomization of a patient to the incorrect site, patients opting out of the exercise program, surgery at another hospital.

10.4 Demographic and Baseline Variables

Baseline characteristics of participants in each arm will be reported using descriptive statistics. Specifically, continuous variables will be presented as means with standard deviation (SD) or median with the interquartile range (IQR) for skewed distributions. Additionally, categorical variables will be reported using frequency and proportion. The following baseline characteristics will be tabulated by arm and overall: age, sex, weeks from enrollment to surgery, Clinical Frailty Score, consent method, and COVID-19 status (Table 2).

Table 2. Baseline Characteristics

	**Overall (n=)**	**Control (n=)**	**Intervention (n=)**
No.			
Age, yr (mean, SD)			
Sex			
Female			

Male			
Weeks from enrollment to surgery (median, IQR)			
Clinical Frailty Score (median, IQR)			
Surgery Type			
Colorectal			
Hepatobiliary			
Thoracic			
Urologic or gynecologic			
Other			
Cancer			
Duke Activity Status Index Score (mean, SD)			
Katz Index (median, IQR)			
History of myocardial infarction			
Congestive heart failure			
History of stroke or transient ischemic attack			
Chronic pulmonary disease			
Diabetes with complications			
Liver disease			
Kidney disease			
Receipt of radiation in the last 6 months			
History of chemotherapy in last 6 months			
Current smoker			
At risk of malnutrition			

10.5 Treatment Compliance

In order to measure treatment compliance, 1 point will be given based on the following criteria: (1) any cardio attempted, (2) 5/10 strength exercises attempted, and (3) 3/6 stretches attempted. Since these components are to be completed 3 times a week and treatment compliance will be measured weekly, the maximum number of compliance points that can be given in a week is 9. Compliance is defined as the proportion of possible compliance points achieved. The final 4 weeks of the exercise intervention are used to calculate compliance; therefore, the maximum points that can be obtained is 36. However, if a participant was in the study for less than 4 weeks, the available data will be used to determine compliance. For example, if a participant is in the study for 3 weeks, they would have a maximum of 27 possible points. Participants who achieve a compliance score of greater than 75% and enrollment of 3 weeks of more will be included in the per protocol analysis.

11 Effectiveness Analyses

11.1 Primary Effectiveness Analysis

Mixed-effects logistic regression will be used to analyse the co-primary outcome of in-hospital complications; the treatment effect estimate will be expressed as an adjusted odds ratio (OR) with 97.5% confidence interval (CI). The fixed terms in the analytical model will be study arm and the stratification factors (cancer vs. non-cancer surgery) along with prespecified baseline covariates: age, sex, surgery type, malnutrition risk (CNST), and frailty score (CFS). To account for the centre effect (stratification factor), a random intercept will be added to the analysis. In addition to the adjusted ORs, absolute risk differences and 97.5% CIs will also be reported. To obtain absolute risk differences, marginal standardization will be used. Marginal standardization is the preferred method of obtaining absolute risk differences as logistic regression with an identity link can lead to issues such as producing potentially impossible predicted values[25]. To obtain the standard errors, bootstrapping will be used with at least 1000 replications[25].

ANCOVA will be used to analyse the co-primary outcome WHODAS Disability score at 30 days. The covariates included in this analysis will be the baseline value for the WHODAS Disability score, along with the fixed terms of study arm, stratification factors (cancer surgery, consent method), and prespecified covariates (age, sex, surgery type, malnutrition risk, frailty score). To account for the multicentre trial design, a random effect of centre will be used. The model will be estimated using Restricted Maximum Likelihood. The intervention effect will be reported as an adjusted mean difference with 97.5% CI.

11.2 Secondary Effectiveness Analyses

11.2.1 Secondary Analyses of Primary Effectiveness Endpoint

Secondary repeated measures of all WHODAS disability scores (up to 365 days) will employ restricted maximum likelihood estimation. These analyses will also consider the correlation between the four repeated measurements over time by modeling the covariance matrix. The best-fitting model will be selected using information criteria. To account for differences between the groups at baseline, the model will incorporate fixed terms for time, an interaction between time and group, along with the specified covariates and the random center effect. The difference between the treatment and control arms at the 90-day and 365-day marks will be estimated using adjusted least square mean differences along with their associated confidence intervals.

11.2.2 Analyses of Secondary Endpoints

All analyses of secondary outcomes will take into consideration the stratification factors and the specified covariates from the primary analysis, along with accounting for center effects. Health-related quality of life (HRQoL) and 5STS will undergo the same analytic approach as disability scores. Linear regression will be employed for analyzing step counts, while Cox regression will be used to analyse time to hospital discharge, with in-hospital mortality as a competing risk. The marginal probability of the competing events will be estimated using Cumulative Incidence Functions and statistically compared using subdistributional hazard functions. Overall survival will also be analyzed using Cox regression. Discharge disposition

and complication severity will be analysed using ordinal logistic regression. Health system outcomes such as readmissions, emergency department visits, and subsequent long-term care admissions will be analysed with logistic regression. Binary safety outcomes will follow a similar approach as complications, with potential adjustments for small event numbers using exact methods. Differences in costs of care will be analyzed using generalized linear methods with log link and gamma distribution.

11.3 Exploratory Effectiveness Analyses

Additional analyses will involve a per-protocol analysis, focusing on individuals who underwent their planned surgery, had at least 3 weeks of enrollment in the intervention arm and completed more than 75% of the prescribed exercise sessions as the per-protocol population. Primary outcomes will be examined in pre-defined subgroups, that we hypothesize may exhibit different responses to the intervention, including sex, age (<75 vs. ≥75), cancer diagnosis, and frailty level (4 vs. ≥ 5). We will also compare compliance rates between sexes by incorporating interaction terms between subgroup indicators and the intervention.

Using the same regression modelling approach as the primary analysis of the WHODAS score, for individuals who did not have their planned surgery within 84 days of allocation, but who went on to have their planned surgery within 114 days of allocation, an exploratory effectiveness analysis will be conducted replacing their WHODAS score at day 114 with the WHODAS score recorded 30 days after their delayed surgery.

Using the same regression modelling approach as the primary analysis of complications, a sensitivity analysis will be performed for the POMS outcome, where: (1) individuals who do not have surgery are assigned a POMS complication status of 'no complication'; (2) individuals who had their planned surgery more than 84 days after allocation, but on or before day 114 post-allocation are assigned a POMS complication status based on their hospitalization for their delayed surgery; or (3) those who die prior to surgery are assigned a POMS complication status of 'complication present'.

12 Safety Analyses

On the last day before surgery, patients in both arms will be asked if they have experienced any of the following adverse events (AEs) between randomization and surgery: (1) fall, (2) serious musculoskeletal (MSK) injury, (3) head injury, (4) emergency room visit, or (5) hospital admission. These outcomes will be analysed using Poisson regression or exact methods if event numbers are small. This analysis will be used to compare the incidence of falls, serious injuries and unexpected health care visits between the control and intervention arms.

For individuals in the intervention arm, the following intervention-attributable AEs will be measured: (1) light-headedness, (2) dizziness, (3) mild shortness of breath (SoB), (4) muscle pain, (5) mild worsening of pre-existing health conditions, and (6) other. The proportion of AEs and 95% CIs experienced by intervention arm participants will be reported. These AEs will be identified and rated using the following standard assessment:

Table 3. Standard Assessment for Adverse Events

**Severity**<br>(1=Mild, 2=Moderate, 3=Severe)
**Deemed Serious?**<br>(No=1,<br>If Yes, assign code: 1=Death, or 2=Life-threatening, or 3=Hospitalization (initial or prolonged), or 4=Disability or incapacity, or 5=Required intervention to prevent permanent impairment, 6=Other (as per PI))
**Unexpected as per Protocol?**<br>(0=Expected (light-headed, dizzy, mild SoB, mild, muscle pain, pre-existing health conditions); 1=Unexpected)
**Causality/Relatedness**<br>(1=Definite (clearly related to intervention), 2=Probable (likely related to intervention), 3=Possible (potentially related to intervention), 4=Unlikely (improbably but not impossible related to intervention), 5=Unrelated (clearly not related to intervention), or 6=Unable to determine at this time)

13 Cost-Effectiveness Data

From the perspective of Canada's healthcare system, we will conduct a cost-utility analysis to evaluate whether exercise prehabilitation represents a cost-effective approach. Data on healthcare utilization and the effectiveness of the intervention will be derived from the trial. Micro costing will capture intervention-specific costs. The results of the cost-effectiveness analysis will be reported in the future, not part of the primary report.

14 Reporting Conventions

P-values will be reported to 3 decimal places (p-values less than 0.001 will be reported as <0.001). The mean, standard deviation, and any other statistics other than quantiles, will be reported to one decimal place greater than the original data. Quantiles, such as median, or minimum and maximum will use the same number of decimal places as the original data. Estimated parameters, not on the same scale as raw observations (e.g., regression coefficients) will be reported to 3 significant figures.

15 Summary of Changes to the Protocol and/or SAP

In the protocol, centre was listed as both a fixed and random effect in the analysis. To resolve this issue, we determined that a random center effect would be the most appropriate method[23]. In addition, we have clarified how intercurrent events will be handled and revised the wording of the objective in order to adhere with the new guidance regarding estimands[19]. Furthermore, changes were made to the sample size (it has increased from 750 to 850) to accommodate the strategies for dealing with intercurrent events. Lastly, due to changes related to the COVID-19 pandemic, method of consent (telephone vs. in-person) was done only by phone for the last 830 patients, so this will not be adjusted for in the statistical models.

16 References

1. McIsaac DI, Fergusson DA, Khadaroo R, et al. PREPARE trial: a protocol for a multicentre randomised trial of frailty-focused preoperative exercise to decrease postoperative complication rates and disability scores. *BMJ Open*. 2022;12(8):e064165. doi:10.1136/bmjopen-2022-064165

2. McIsaac DI, Taljaard M, Bryson GL, et al. Frailty as a Predictor of Death or New Disability After Surgery: A Prospective Cohort Study. *Ann Surg*. 2020;271(2):283. doi:10.1097/SLA.0000000000002967

3. Rockwood K. A global clinical measure of fitness and frailty in elderly people. *Can Med Assoc J*. 2005;173(5):489-495. doi:10.1503/cmaj.050051

4. Fried LP, Ferrucci L, Darer J, Williamson JD, Anderson G. Untangling the Concepts of Disability, Frailty, and Comorbidity: Implications for Improved Targeting and Care. *J Gerontol Ser A*. 2004;59(3):M255-M263. doi:10.1093/gerona/59.3.M255

5. McIsaac DI, Wong CA, Huang A, Moloo H, van Walraven C. Derivation and validation of a generalizable preoperative frailty index using population-based health administrative data. *Ann Surg*. 2019;270(1):102-108.

6. McIsaac DI, Bryson GL, van Walraven C. Association of Frailty and 1-Year Postoperative Mortality Following Major Elective Noncardiac Surgery: A Population-Based Cohort Study. *JAMA Surg*. 2016;151(6):538-545. doi:10.1001/jamasurg.2015.5085

7. McIsaac DI, Moloo H, Bryson GL, van Walraven C. The Association of Frailty With Outcomes and Resource Use After Emergency General Surgery: A Population-Based Cohort Study. *Anesth Analg*. 2017;124(5):1653-1661. doi:10.1213/ANE.0000000000001960

8. Kim DH, Kim CA, Placide S, Lipsitz LA, Marcantonio ER. Preoperative Frailty Assessment and Outcomes at 6 Months or Later in Older Adults Undergoing Cardiac Surgical Procedures. *Ann Intern Med*. 2016;165(9):650-660. doi:10.7326/M16-0652

9. Lin HS, Watts JN, Peel NM, Hubbard RE. Frailty and post-operative outcomes in older surgical patients: a systematic review. *BMC Geriatr*. 2016;16(1):157. doi:10.1186/s12877-016-0329-8

10. Wang J, Zou Y, Zhao J, et al. The Impact of Frailty on Outcomes of Elderly Patients After Major Vascular Surgery: A Systematic Review and Meta-analysis. *Eur J Vasc Endovasc Surg*. 2018;56(4):591-602. doi:10.1016/j.ejvs.2018.07.012

11. Aucoin SD, Hao M, Sohi R, et al. Accuracy and Feasibility of Clinically Applied Frailty Instruments before Surgery: A Systematic Review and Meta-analysis. *Anesthesiology*. 2020;133(1):78-95. doi:10.1097/ALN.0000000000003257

12. Alvarez-Nebreda ML, Bentov N, Urman RD, et al. Recommendations for Preoperative Management of Frailty from the Society for Perioperative Assessment and Quality Improvement (SPAQI). *J Clin Anesth*. 2018;47:33-42. doi:10.1016/j.jclinane.2018.02.011

13. McIsaac DI, Gill M, Boland L, et al. Prehabilitation in adult patients undergoing surgery:

an umbrella review of systematic reviews. *Br J Anaesth*. 2022;128(2):244-257. doi:10.1016/j.bja.2021.11.014

14. McIsaac DI, Jen T, Mookerji N, Patel A, Lalu M. Interventions to improve the outcomes of frail people having surgery: a systematic review. *PLOS ONE*. 2017;12(e0190071). Accessed September 19, 2023. https://onlinelibrary.wiley.com/doi/full/10.1111/aas.13239?casa_token=6GF-Aog4igcAAAAA%3AAtj1jOXnAeq9O6loIEJ4jtFnPotNOrPQzqQ9eX4Klq5bCDOKnc9J5xVem9yjBOsykPTrBs8_o_11mQ

15. Milder DA, Pillinger NL, Kam PCA. The role of prehabilitation in frail surgical patients: A systematic review. *Acta Anaesthesiol Scand*. 2018;62(10):1356-1366. doi:10.1111/aas.13239

16. Whitney SL, Wrisley DM, Marchetti GF, Gee MA, Redfern MS, Furman JM. Clinical Measurement of Sit-to-Stand Performance in People With Balance Disorders: Validity of Data for the Five-Times-Sit-to-Stand Test. *Phys Ther*. 2005;85(10):1034-1045. doi:10.1093/ptj/85.10.1034

17. Katz S, Ford AB, Moskowitz RW, Jackson BA, Jaffe MW. STUDIES OF ILLNESS IN THE AGED. THE INDEX OF ADL: A STANDARDIZED MEASURE OF BIOLOGICAL AND PSYCHOSOCIAL FUNCTION. *JAMA*. 1963;185:914-919. doi:10.1001/jama.1963.03060120024016

18. Pleyer L, Heibl S, Tinchon C, et al. Health-Related Quality of Life as Assessed by the EQ-5D-5L Predicts Outcomes of Patients Treated with Azacitidine—A Prospective Cohort Study by the AGMT. *Cancers*. 2023;15(5):1388. doi:10.3390/cancers15051388

19. International Council for Harmonisation (ICH). Addendum on estimands and sensitivity analysis in clinical trials to the guideline on statistical principles for clinical trials E9 (R1). *Fed Regist*. Published online 2019:1-19.

20. McIsaac DI, Hladkowicz E, Bryson GL, et al. Home-based prehabilitation with exercise to improve postoperative recovery for older adults with frailty having cancer surgery: the PREHAB randomised clinical trial. *Br J Anaesth*. 2022;129(1):41-48. doi:10.1016/j.bja.2022.04.006

21. Watt J, Tricco AC, Talbot-Hamon C, et al. Identifying Older Adults at Risk of Delirium Following Elective Surgery: A Systematic Review and Meta-Analysis. *J Gen Intern Med*. 2018;33(4):500-509. doi:10.1007/s11606-017-4204-x

22. Shulman MA, Kasza J, Myles PS. Defining the Minimal Clinically Important Difference and Patient-acceptable Symptom State Score for Disability Assessment in Surgical Patients. *Anesthesiology*. 2020;132(6):1362-1370. doi:10.1097/ALN.0000000000003240

23. Kahan BC. Accounting for centre-effects in multicentre trials with a binary outcome – when, why, and how? *BMC Med Res Methodol*. 2014;14(1):20. doi:10.1186/1471-2288-14-20

24. Liu Y, De A. Multiple Imputation by Fully Conditional Specification for Dealing with

Missing Data in a Large Epidemiologic Study. *Int J Stat Med Res*. 2015;4(3):287-295. doi:10.6000/1929-6029.2015.04.03.7

25. Muller CJ, MacLehose RF. Estimating predicted probabilities from logistic regression: different methods correspond to different target populations. *Int J Epidemiol*. 2014;43(3):962-970. doi:10.1093/ije/dyu029

17 Listing of Tables and Figures

Table 1: Baseline characteristics by allocation

Table 2: Primary and secondary outcomes (Intervention/Control/Adjusted effect measure/P-value

Figure 1: CONSORT diagram

Figure 2 : Forest plot of subgroup estimates for the two primary outcomes

File 2C: Statistical Analysis Plan Summary of Changes

Statistical Analysis Plan Summary of Changes

Statistical Analysis Plan Version	Summary of Changes
SAP 1.0 – May 10, 2024	
SAP 2.0 – October 31, 2024	- As COVID-19 arose immediately after study start up, almost all participants were consented by telephone, therefore strata for in-person consent are essentially empty so adjustment in outcome and imputation models will not include consent method.<br><br>- Pedometer readings were unreliably captured by older participants; therefore, values were highly missing across the full population. As such, this value will not be included in the imputation model or analyzed as a secondary outcome.
