# Supplementary material for: Home-Based Prehabilitation for Older Surgical Patients With Frailty: A Randomized Clinical Trial
Source: JAMA Surg. 2025 Dec 3;161(2):113–23. doi: 10.1001/jamasurg.2025.5288 (PMC12676472; doi:10.1001/jamasurg.2025.5288)
Supplement: Supplement 2. — Statistical Analysis Plan [file jamasurg-e255288-s002.pdf]

Statistical Analysis Plan

1 Administrative Information

Date	2024/10/31
Study Title	PREPARE Trial: a parallel arm multicentre randomised trial of frailty-focused preoperative exercise to decrease postoperative complication rates and disability scores
Study Registration Number	NCT04221295
SAP Version Number	2
Protocol Version and Date	2, 2024/10/31
Trial Statistician	Dr. Monica Taljaard
Trial Principal Investigator	Dr. Daniel McIsaac
SAP Author(s)	Caroline Lee

Revision Control

Protocol Version	Updated SAP version number	Section number changed	Description of change	Date changed
2	2	9.4	Removed step count from imputation model as the value was highly missing due to poor pedometer function amongst older adults.	2024/10/31; prior to any outcome analyses
2	2	9.4	Removed consent method from the imputation model to reflect pre-specified removal of consent method as a stratification factor due to COVID-19 related changes to telephone vs. in-person consent	2024/10/31; prior to any outcome analyses

Roles and Responsibilities

**Name**	**Role**	**Institution**
Dr. Daniel McIsaac	Principal Investigator	University of Ottawa
Dr. Dean Fergusson	Lead Epidemiologist	Ottawa Hospital Research Institute

Dr. Monica Taljaard	Lead Biostatistician	Ottawa Hospital Research Institute
Dr. Rachel Khadaroo	Lead Surgeon	University of Alberta
Dr. John Muscedere	Co-Principal Investigator and Knowledge User	Queen's University
Amanda Meliambro	Co-Principal Investigator and Patient Partner	Memorial University of Newfoundland

2 Abbreviations and Definitions

5TSTS	5-Times-Sit-To-Stand
AD8	8-item Informant Interview to Differentiate Aging and Dementia
CFS	Clinical Frailty Scale
CI	Confidence Interval
CNST	Canadian Nutrition Screening Tool
DASI	Duke Activity Status Index
DIMR	Alberta-Alberta Data Integration, Measurement & Reporting
DSMB	Data Safety Monitoring Board
EQ-5D-5L	5-level EuroQoL health related quality of life
KATZ	Katz Index of Independence in Activities of Daily Living
HRQoL	Health-related Quality of Life
ICES	Institute of Clinical Evaluative Sciences
ITT	Intention-to-Treat
LoS	Length of Stay
MCHP	Manitoba-Manitoba Center for Health Policy
PHQ	Personal Health Questionnaire
POMS	Postoperative Morbidity Questionnaire
SD	Standard Deviation
WHODAS	World Health Organization Disability Assessment Schedule 2.0

3 Introduction

Frailty is a multidimensional state of vulnerability arising from age- and disease-related deficits accumulating throughout a patient's life[1–5]. In cases where frailty is present prior to surgery, the rates of new patient-reported disability, major complications, hospital readmission, non-home discharge, and death increase by more than twofold[1,6–11]. Improving physical and physiological status prior to surgery could mitigate adverse postoperative outcomes for older individuals with frailty[11,12]. One approach to enhance patients' physical and physiological well-being is prehabilitation, which actively readies patients for surgery using exercise, nutrition, psychocognitive interventions or a combination of these methods[13]. Recent research shows that prehabilitation could offer advantages in terms of reducing complication rates, decreasing non-home discharge, shortening length of stay, and promoting functional recovery[13]. However, there have been few perioperative trials that include older people with frailty[14,15]. Furthermore, the existing trials have relatively small sample sizes and are single centre[14,15]. Therefore, addressing knowledge gaps through a multicenter trial specifically focused on individuals with frailty is required to evaluate the effectiveness of home-based prehabilitation in reducing patient-reported disability and postoperative complications. [13].

4 Study Objectives, Endpoints, and Estimands

4.1 Study Objectives

This trial was designed to evaluate the effectiveness of a home-based multimodal prehabilitation intervention in decreasing patient-reported disability and postoperative complications in older people with frailty having major surgery. The intervention consists of a structured, home-based, multimodal prehabilitation program that provides personalized exercise and nutritional recommendations. The exercise regimen entails 1-hour sessions three times a week for at least three weeks, incorporating strength training, aerobic exercises, and flexibility exercises. Participants will receive a telephone-assisted education session on the exercise program and are further supported by an experienced central team that will conduct weekly phone calls to ensure safety, encourage adherence, and provide guidance on exercise progression. Participants in the control group receive static activity and healthy eating recommendations but do not receive active support, logs, or regular contact.

The primary objective is to determine if the prehabilitation program will reduce patient-reported disability 30-days after surgery and/or in-hospital complications. The secondary objectives will assess the effectiveness of the program on patient-centered outcomes (i.e., discharge home, survival, 1-year disability scores, quality of life, frailty, function) and system-relevant outcomes (i.e., length of stay readmissions). An additional objective of this trial relates to evaluating cost-effectiveness of the program.

4.2 Endpoints

The trial has two primary outcomes, defined at the patient-level: an index of patient-reported disability at 30 days after surgery (continuous) and presence of any in-hospital complications (binary). These outcomes were selected based on priorities for older surgical patients, proposed causal mechanisms between exercise prehabilitation and outcomes, and systematic review efficacy data.

The continuous co-primary outcome, patient reported disability 30 days after surgery, will be measured using the World Health Organization Disability Assessment Schedule 2.0 (WHODAS)[1]. The WHODAS is a patient-reported disability scale that assesses limitations in six major life domains, namely cognition, mobility, self-care, social interaction, life activities, and participation in society. The WHODAS contains questionnaire items that are scored on a Likert scale from 0 to 4. The WHODAS Disability Score ranges from 0 to 48 and is expressed as a percentage (0 to 100) of the maximum possible score. If a participant dies before follow-up, they will be considered completely disabled (i.e., assigned a score of 100).

In-hospital complications will be measured using the Postoperative Morbidity Questionnaire (POMS), which is a prospectively administered instrument designed to identify significant in-hospital complications in key organ systems[1]. The POMS consists of 18 items addressing nine domains: pulmonary, infectious, renal, gastrointestinal, cardiovascular, neurological, hematological, and wound pain; the pain domain will not be included in the PREPARE study composite definition as pre-specified based on causal mechanisms. The POMS uses medical charts, medications records, vital signs records, routine lab tests, and direct questioning of the patient to determine the presence or absence of a complication. Any POMS complication or death in hospital will be recorded as a complication (i.e., a composite outcome).

The secondary outcomes are reflected by the following five domains: (1) function, (2) health-related quality of life, (3) all-cause mortality, (4) health system outcomes, (5) safety.

Function will be measured using three different outcomes: (1) total step counts, (2) Five-Times sit to stand test (5TSTS), and (3) the Katz Index. Step count will be recorded daily for 30 days after surgery, using a pedometer, and will be expressed as a daily average over all available days. The 5TSTS measures the lower-extremity strength and balance of a patient[16]. The Katz Index measures the independence level of daily living

activities, on a scale from 0 to 6, where 0 indicates the highest level of dependence and 6 indicates the highest level of independence[17].

Health-related quality of life will be measured using the 5-level EuroQoL health related quality of life (EQ-5D-5L). The EQ-5D-5L measures quality of life on the following five dimensions: (1) mobility, (2) self-care, (3) usual activities, (4) pain/discomfort, and (5) anxiety/depression[18]. Postoperative pain will be captured from the pain domain of the POMS instrument.

All-cause mortality will be identified in-hospital or through telephone follow-up up at 30 days, 90 days, and 1-year post-surgery. The severity of in-hospital complications will also be captured using the Clavien-Dindo scale as an ordinal secondary outcome.

Discharge disposition (home, home with support, rehabilitation, long term care) will be prospectively collected at discharge by telephone. Where possible, trial data will be linked to repositories of routinely collected health data. Linkage to these databases will allow for collection of (1) health system costs, (2) readmissions, (3) emergency department visits, and (4) subsequent long-term care admissions in the year after surgery.

Lastly, patient safety will be measured through (1) falls, musculoskeletal (MSK) injury, and head injury, and (2) unplanned health encounters (emergency room visits and hospital admissions). This information will be measured during the post-randomization pre-surgery period. These measures will be collected in both arms. Additional safety measures that will only be measured in the intervention arm are new or worsened symptoms and adherence.

4.3 Intercurrent Events

Intercurrent events (ICEs) are events that occur after treatment that may affect the interpretation of the endpoints[19]. We anticipate and have accounted for six potential types of intercurrent events in our analysis plan: (1) patients who do not have a surgery performed during the trial (for a reason other than death), (2) patients who die post-randomization, but pre-surgery, (3) patients who die outside of hospital post-surgery, (4) patients who withdraw from treatment but still complete data collection, (5) patients who do not adhere to treatment but still complete data collection, and (6) patients who are lost to follow-up (do not complete data collection). There are five potential strategies for dealing with intercurrent events[19]: (1) *treatment policy strategy* in which the measured value of the outcome variable is used in the analysis regardless of occurrence of the intercurrent event (i.e., consistent with a traditional intention-to-treat approach), (2) *hypothetical strategy* in which the value of the outcome variable is estimated in the hypothetical scenario in which the intercurrent event would not have occurred, (3) *composite variable strategy* in which the intercurrent event is incorporated into the definition of the variable, (4) *while-on-treatment strategy* in which the response to treatment prior to occurrence of the intercurrent event is of interest, and (5) *principal stratum strategy* in which the target population is defined to be a "principal stratum" in which an intercurrent event would or would not occur. Our chosen strategy for dealing with each of these intercurrent events in the primary analysis for each co-primary outcome is summarized in the table.

PRIMARY ANALYSIS

Intercurrent event	POMS	WHODAS at 30 days
Do not have surgery performed for reason other than death	Exclude (*principal stratum strategy)* assuming the reason for not having surgery is unrelated to	Include (*treatment policy strategy*)

	trial arm	
Die before surgery	Exclude (*principal stratum strategy)* assuming the reason for death before surgery is unrelated to trial arm	Include (*composite variable strategy*): assigned the worst possible outcome
Die outside of hospital after surgery	Not applicable: patients who are discharged from the hospital can no longer experience an in-hospital complication	Include (*composite variable strategy*): assigned the worst possible outcome
Withdraw from treatment	Include (*treatment policy strategy*)	Include (*treatment policy strategy*)
Do not adhere to treatment	Include (*treatment policy strategy*)	Include (*treatment policy strategy*)
Lost to follow-up or withdraw from trial	Include (*hypothetical strategy*): Participants with missing data are included in the analysis using a multiple imputation model under the assumption that reasons for attrition are related only to observed data	Include *(hypothetical strategy):* Participants with missing data are included in the analysis using a multiple imputation model under the assumption that reasons for attrition are related only to observed data

For the co-primary outcome of patient-reported disability at 30 days (WHODAS), all patients will be included in the analysis; thus, we will adopt the treatment policy strategy by using the measured WHODAS (at day 114 post-randomization) for patients who do not have surgery and those who withdraw from treatment. Patients who die outside of hospital post-randomisation will be considered fully disabled and assigned WHODAS scores of 100 (composite variable strategy).

For the co-primary outcome of in-hospital complications, patients who do not have surgery and patients who die post-randomisation but prior to having surgery cannot be considered to be in the risk set for a postoperative complication; therefore, these individuals will be excluded from the analysis. In addition, POMS will still be recorded for individuals who withdraw from the intervention or do not adhere to treatment (treatment policy strategy).

4.4 Target Estimands

The target estimand for the co-primary outcome of patient-reported disability is the mean difference in the WHODAS score at day 30 in patients with frailty planning to undergo elective inpatient surgery and allocated to the prehabilitation program versus those allocated to usual care.

The target estimand for the co-primary outcome of in-hospital complications is the relative odds of in-hospital complications for patients with frailty undergoing elective inpatient surgery and allocated to the prehabilitation program versus those allocated to usual care.

5 Study Methods

5.1 General Study Design and Plan

This study is a multicenter, parallel arm individually randomized controlled trial conducted across 11 Canadian community and academic hospitals, with the Ottawa Methods Centre serving as the coordinating center.

Participants will be recruited from surgery or anesthesia clinic lists by telephone. Eligible individuals who consent to research contact, are expected to undergo surgery in 3-12 weeks, and are 60 years or older will be assessed for frailty using the Clinical Frailty Scale (CFS). Those scoring $\geq$ 4/9 on the CFS will be given the opportunity to provide written or verbal informed consent to participate in the trial. Baseline data will be collected from all participants before randomization. Subsequently, participants will be randomly assigned to either the intervention or control group.

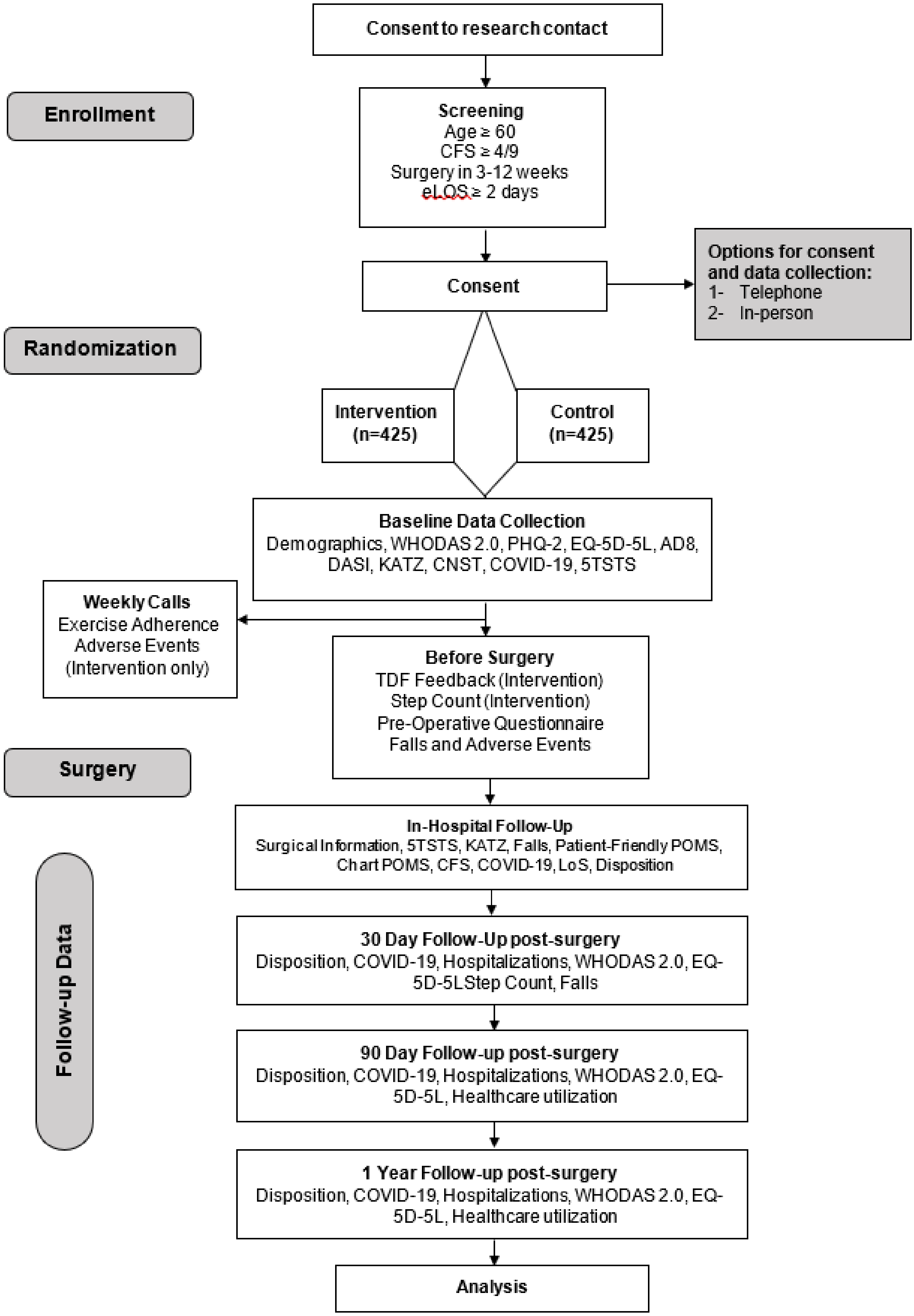
Consent to research contact
Enrollment
Screening
Age ≥ 60
CFS ≥ 4/9
Surgery in 3-12 weeks
eLOS ≥ 2 days
Consent
Options for consent and data collection:
1- Telephone
2- In-person
Randomization
Intervention (n=425)
Control (n=425)
Baseline Data Collection
Demographics, WHODAS 2.0, PHQ-2, EQ-5D-5L, AD8, DASI, KATZ, CNST, COVID-19, 5TSTS
Weekly Calls
Exercise Adherence
Adverse Events
(Intervention only)
Before Surgery
TDF Feedback (Intervention)
Step Count (Intervention)
Pre-Operative Questionnaire
Falls and Adverse Events
Surgery
In-Hospital Follow-Up
Surgical Information, 5TSTS, KATZ, Falls, Patient-Friendly POMS, Chart POMS, CFS, COVID-19, LoS, Disposition
Follow-up Data
30 Day Follow-Up post-surgery
Disposition, COVID-19, Hospitalizations, WHODAS 2.0, EQ-5D-5LStep Count, Falls
90 Day Follow-up post-surgery
Disposition, COVID-19, Hospitalizations, WHODAS 2.0, EQ-5D-5L, Healthcare utilization
1 Year Follow-up post-surgery
Disposition, COVID-19, Hospitalizations, WHODAS 2.0, EQ-5D-5L, Healthcare utilization
Analysis

132

5.2 Inclusion-Exclusion Criteria and General Study Population

The inclusion criteria of the study are: (1) participants aged ≥ 60 years, (2) elective inpatient surgery, (3) the expected surgery date is between 3-12 weeks from enrollment, and (4) a Clinical Frailty Scale (CFS) score greater or equal to 4/9.

The exclusion criteria of the study are: (1) the inability to speak English or French, (2) the presence of comorbidities preventing assessment or the ability to understand the questionnaires, (3) the inability to be contacted by telephone, (4) the unwillingness to participate in the exercise programme, (5) cardiac, neurological, or orthopaedic procedure, (6) palliative surgery, and (7) any of the following cardiovascular conditions: (i) severe valvular heart disease, (ii) severe cardiac dysrhythmias, or (iii) myocardial infraction within the 6 weeks prior to enrolment.

5.3 Randomization and Blinding

The allocation sequence will be computer-generated by an independent biostatistician using permuted blocks of randomly varying lengths, stratified by centre, cancer vs non-cancer surgery, and consent method (in-person vs telephone). The randomisation sequence will be accessed through a central secure internet-based application to ensure allocation concealment. After informed consent is obtained, the researcher will log onto the central secure internet-based application with a password, obtained by the Ottawa Methods Center.

The clinicians and outcome assessors will be fully blinded to potential treatment allocation groups. The participants will be partially blinded as they will be informed that they are being enrolled in a study to evaluate activity interventions before surgery.

5.4 Study Assessments

The study assessment schedule is provided in Table 1. The co-primary outcome of the WHODAS will be assessed at baseline, 30 days, 90 days, and 1-year post-surgery with 30 days being specified as the primary endpoint. The co-primary outcome POMS will be assessed postoperatively during the index surgical hospitalization.

The following baseline characteristics will be measured: demographics, Clinical Frailty Scale (CFS), Patient Health Questionnaire (PHQ-2), AD8 Cognitive Screen (AD8), Duke Activity Status Index (DASI), Canadian Nutrition Screening Tool (CNST), cancer status, neoadjuvant therapy status.

To assess intervention safety, falls and unplanned healthcare encounters will be identified during the intervention period but prior to surgery. During this period, prespecified intervention attributable events and intervention compliance will also be measured in the intervention arm.

The secondary outcome measuring function, the 5TSTS, will be measured at baseline and postoperatively at the time of hospital discharge. Additionally, step counts, which also measure function, will be assessed, and averaged over the available days in the 30 days post surgery period. Health-related quality of life, measured through the EQ-5D-5L, will be assessed at baseline, 30 days, 90 days, and 1 year post surgery. Discharge disposition, CFS and length of stay (LoS) will be measured postoperatively at hospital discharge. Postoperative falls will be identified within 30 days post-surgery. Any deaths, and their date, will be identified up to 1-year post-surgery.

Table 1. Timing of Study Assessments

Domain	Assessment	Baseline	Surgery	Postop in-hospital	30 days postop	90 days postop	365 days postop

Co-Primary Outcomes	WHODAS	X			X	X	X
	POMS			X			
Baseline characteristics	Demographics	X					
	CFS	X		X			
	PHQ-2	X					
	AD8	X					
	DASI	X					
	CNST	X					
	Cancer/Chemo	X					
	COVID-19	X			X	X	X
Secondary Outcome: Function	Step Counts				X		
	5TSTS	X		X			
	Katz	X		X			
Secondary Outcome: HRQoL	EQ-5D-5L	X			X	X	X
Secondary Outcome: All-cause mortality	Survival				X	X	X
Secondary Outcome: Health System	Disposition			X			
	LoS			X			
Secondary Outcome: Safety	Adjudicated Adverse Events		X				
	Falls			X	X	X	X
	Readmissions			X	X	X	X

6 Sample Size

The sample size is driven by the binary primary outcome of in-hospital post-operative complications (POMS). Our initial target sample size was 750 participants (375 participants in each arm). After publication of the protocol, the trial steering committee approved an increase in the target sample size to 850 participants (425 in each arm) to allow for the following assumptions:

1) A 55% complication rate among those undergoing surgery in the control arm, which is informed by data from our prior trial and related systematic reviews[20,21];
2) A 28% relative reduction in complications in the intervention arm among those undergoing surgery and being adherent to the intervention;
3) 15% non-adherence in the intervention arm (i.e., patients not doing any exercise);
4) 15% attrition, which accounts for patients not having surgery and 5% of patients being lost to follow-up.

With these assumptions, the target of 850 participants achieves 90% power to detect a relative difference of 23.8% in the intention to treat analysis (a control arm event rate of 0.55 versus an attenuated intervention arm event rate of 0.4191) using a pooled Z-test with a two-sided alpha of 0.025.

For the continuous co-primary outcome (WHODAS Disability Score), a sample size of 850 achieves 90% power to detect a difference of 5 points on a 100-point scale using an analysis of covariance (ANCOVA) at the two-sided alpha level of 0.025 in the intention to treat analysis. This difference is smaller than the minimum clinically important difference of 8 points used in the study protocol, and accounts for our conservative strategy

of dealing with intercurrent events[22]. It is also in line with subsequent publications suggesting a minimally important difference of 5 specifically for surgical patients. We assumed a common SD of 20 (consistent with previous publications[20]), a correlation between baseline and postoperative score of 0.4, and accounted for 5% attrition. A Bonferroni correction was used to maintain the overall type I error rate across the two primary outcomes at 5%.

7 General Analysis Considerations

7.1 Timing of Analyses

The trial is due to finish once the 1-year follow-up post-surgery for the last recruited patient has been obtained. As the primary outcome data will be complete approximately 30-days after the last recruited patient has surgery, the data will be cleaned, verified, and locked for the initial analyses at 30 days after the last participant has surgery. Final analysis will commence once the final lock has been confirmed by the Principal Investigator.

7.2 Analysis Populations

7.2.1 Full Analysis Population

The full analysis population differs between the two co-primary outcomes. For WHODAS, the full analysis population will consist of all participants who were randomized (as prehabilitation can influence disability status regardless of having surgery). In the case of patients who receive the intervention, but do not undergo surgery, WHODAS will still be collected 114 days post-randomization. Furthermore, individuals who die post-randomization will be recorded as fully disabled on WHODAS (i.e., they will receive a score of 100). Therefore, this analysis population will consist of all participants who where randomized regardless of whether they received the intervention (i.e., intention to treat) or had surgery.

For POMS, the full analysis population will consist of individuals who are part of the risk set for postoperative complications (i.e., patients who were randomized and underwent surgery, as people who do not have surgery cannot be at risk of a postoperative complication). Participants who don’t have surgery or who die prior to surgery will not be included in the analysis. POMS will be recorded regardless of whether participants withdraw from treatment or do not adhere to the intervention.

7.2.2 Per Protocol Population

The per protocol analysis population will consist of all individuals in the control arm who had their planned surgery, and all individuals in the intervention arm who had their planned surgery and who completed > 75% of their prescribed exercise sessions.

7.2.3 Safety Population

The safety population will consist of all the study participants. Furthermore, there will be an additional intervention-attributable safety analysis performed only on patients who were allocated to the intervention.

7.3 Covariates and Subgroups

Primary and secondary analyses will be adjusted for stratification factors and postulated predictors of outcome (to enhance power). In the original study protocol, it was specified that randomization would be stratified on the consent method (in-person vs. telephone). However, due to the COVID-19 pandemic, and after only 20 patients had already been recruited in-person, the use of in-person consent was no longer permitted. For

that reason, the analysis will only adjust for cancer versus non-cancer surgery (binary), and center (as a random intercept) as stratification factors.[23] Other covariates, postulated to be predictive of outcome that will be included in outcome regression models are age (continuous), sex (binary), malnutrition risk (binary), and frailty score (binary, 4 vs. ≥5 on the CFS).

Preplanned subgroup analyses for the two primary outcomes will be conducted based on sex, age (<75 vs ≥75), presence of cancer, presence of depression, and frailty status (4 vs ≥ 5). Subgroup analyses will be conducted using an effect modifier approach where the subgroup indicator will be tested as an interaction term with treatment allocation.

7.4 Missing Data

Since ITT analyses are being used in this trial, all eligible patients will be included. To account for missing data due to attrition, a multiple imputation model will be used to maintain power and attenuate missing data bias. The multiple imputation model will be used to impute missing outcome data for patients who withdrew entirely from the study or for individuals lost to follow up. Multiple imputation will not be used to assign outcome values for patients who have died, or who did not receive surgery. Instead, these intercurrent events will be managed using the strategies described in Section 6.3.

Prior to any statistical analysis being conducted, multiple imputation using the fully conditional specification method will be performed to create a dataset with no missing observations. In fully conditional specification multiple imputation, the missing variables are imputed by creating an imputation model for each missing variable, given the other available variables[24]. The multiple imputation model will include the following variables: (1) WHODAS, (2) POMS, (3) age, (4) sex, (5) surgery type, (6) malnutrition risk (CNST), (7) frailty score (CFS), (8) centre, (9) cancer surgery, (10) 5TSTS, (11) Katz Index, (12) EQ-5D-5L, (13) survival, (14) disposition, and (15) length of stay.

Since the primary and secondary outcomes are measured at multiple time points throughout the trial, the multiple imputation model will use all the data present until the participant withdrew from the trial. The multiple imputation analysis will be repeated at least 10 times (depending on the fraction of missing information) and Rubin's rule will be used to determine the average value for each missing value.

7.5 Interim Analyses and Data Monitoring (as applicable)

7.5.1 Purpose of Interim Analyses

An interim analysis for safety was completed and reviewed by the DSMB on August 9th, 2022, after enrollment of 50% of the sample size.

7.5.2 Planned Schedule of Interim Analyses

The interim safety analysis was performed on the safety population when they completed their 30-day follow up.

7.5.3 Scope of Adaptations

During the interim analysis, baseline data, compliance data, all outcome data related to complications, safety, and adverse events was collected, while masking the treatment allocation.

7.5.4 Stopping Rules

The DSMB recommended that the trial continue following the interim safety analysis.

7.5.5 Adjustment of Confidence Intervals and P-values

Since there are two primary outcomes and an effect on at least one of the outcomes is desired, a multiplicity adjustment is required. For both primary outcomes, a two-sided alpha value of 0.025 will be used for the hypothesis tests for the primary outcomes and 97.5% confidence intervals (CIs) will be reported. No adjustment will be applied for prespecified secondary outcomes, which will be reported using 95% CIs.

8 Summary of Study Data

8.1 Subject Disposition

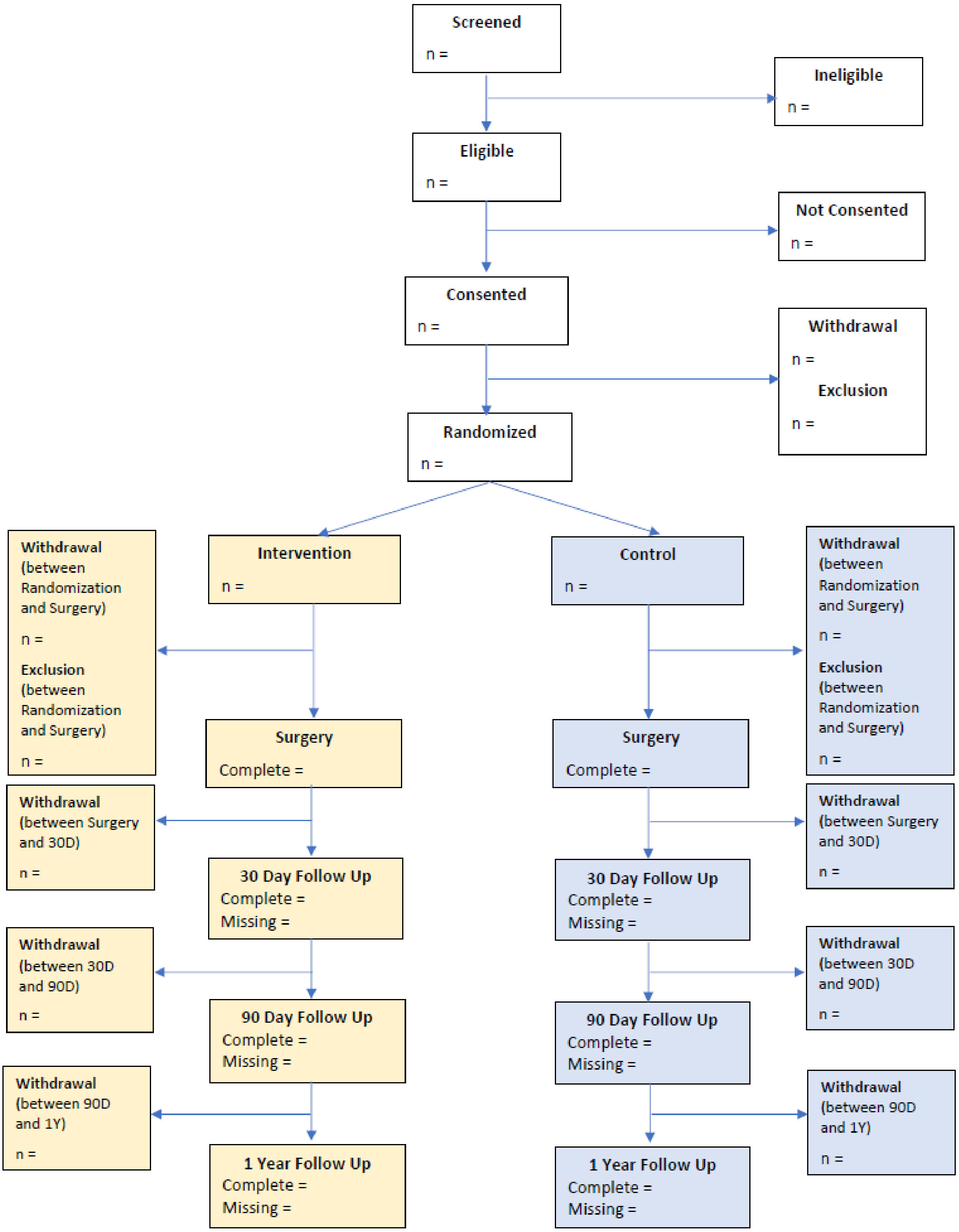

8.2 Derived variables

The following 12 components are measured to determine the WHODAS score: (1) standing for long periods such as 30 min, (2) taking care of household responsibilities, (3) learning a new task, for example learning how to get a new place, (4) how much of a problem have you had joining in community activities, (5) how much have you been emotionally affected by your recent surgery, (6) concentrating on doing something for 10 mins, (7) walking a long distance (like a kilometer), (8) washing your whole body independently, (9) getting dressed independently. (10) dealing with people you don't know, (11) maintaining a friendship, and (12) doing your day-to-day work. Each component is measured on a scale from 0 to 4 where 0=None, 1=Mild, 2=Moderate, 3=Severe, 4=Extreme or cannot do, and not applicable. This results in a range of WHODAS scores from 0 to 48, which is then presented as percentage of the maximum possible score (i.e., 0 to 100).

In addition, we will also record the answers to the following questions: (1) in the last 30 days, how many days were these difficulties present, (2) in the last 30 days, how many days were you totally unable to carry out your usual activities because of your health, (3) not counting the days you were unable, in the last 30 days how many days did you have to cut back or reduce your usual activities because of your health, and (4) How much of an impact did the COVID-19 pandemic have on your ability to perform activities described in the previous survey. The last question will be answered using the following scale: major impact, moderate impact, minor impact, no impact, and too soon to tell.

8.3 Protocol Deviations

The following protocol deviations could occur: wrong consent version used, data collected outside protocol defined time points, no surgery performed within 84 days of randomization, no program orientation for intervention participant (typically because surgery would be scheduled sooner than anticipated), participant unblinded for baseline data collection, surgery less than 3 weeks from enrollment, wrong version of materials sent to participants, the randomization of a patient to the incorrect site, patients opting out of the exercise program, surgery at another hospital.

8.4 Demographic and Baseline Variables

Baseline characteristics of participants in each arm will be reported using descriptive statistics. Specifically, continuous variables will be presented as means with standard deviation (SD) or median with the interquartile range (IQR) for skewed distributions. Additionally, categorical variables will be reported using frequency and proportion. The following baseline characteristics will be tabulated by arm and overall: age, sex, weeks from enrollment to surgery, Clinical Frailty Score, and COVID-19 status (Table 2).

Table 2. Baseline Characteristics

	**Overall (n=)**	**Control (n=)**	**Intervention (n=)**
No.			
Age, yr (mean, SD)			
Sex			
Female			
Male			
Weeks from enrollment to surgery (median, IQR)			
Clinical Frailty Score (median, IQR)			

Surgery Type			
Colorectal			
Hepatobiliary			
Thoracic			
Urologic or gynecologic			
Other			
Cancer			
Duke Activity Status Index Score (mean, SD)			
Katz Index (median, IQR)			
History of myocardial infarction			
Congestive heart failure			
History of stroke or transient ischemic attack			
Chronic pulmonary disease			
Diabetes with complications			
Liver disease			
Kidney disease			
Receipt of radiation in the last 6 months			
History of chemotherapy in last 6 months			
Current smoker			
At risk of malnutrition			

8.5 Treatment Compliance

In order to measure treatment compliance, 1 point will be given based on the following criteria: (1) any cardio attempted, (2) 5/10 strength exercises attempted, and (3) 3/6 stretches attempted. Since these components are to be completed 3 times a week and treatment compliance will be measured weekly, the maximum number of compliance points that can be given in a week is 9. Compliance is defined as the proportion of possible compliance points achieved. The final 4 weeks of the exercise intervention are used to calculate compliance; therefore, the maximum points that can be obtained is 36. However, if a participant was in the study for less than 4 weeks, the available data will be used to determine compliance. For example, if a participant is in the study for 3 weeks, they would have a maximum of 27 possible points. Participants who achieve a compliance score of greater than 75% and enrollment of 3 weeks of more will be included in the per protocol analysis.

9 Effectiveness Analyses

9.1 Primary Effectiveness Analysis

Mixed-effects logistic regression will be used to analyse the co-primary outcome of in-hospital complications; the treatment effect estimate will be expressed as an adjusted odds ratio (OR) with 97.5% confidence interval (CI). The fixed terms in the analytical model will be study arm and the stratification factors (cancer vs. non-cancer surgery) along with prespecified baseline covariates: age, sex, surgery type, malnutrition risk (CNST), and frailty score (CFS). To account for the centre effect (stratification factor), a random intercept will be added to the analysis. In addition to the adjusted ORs, absolute risk differences and 97.5% CIs will also be reported. To obtain absolute risk differences, marginal standardization will be used. Marginal

standardization is the preferred method of obtaining absolute risk differences as logistic regression with an identity link can lead to issues such as producing potentially impossible predicted values[25]. To obtain the standard errors, bootstrapping will be used with at least 1000 replications[25].

ANCOVA will be used to analyse the co-primary outcome WHODAS Disability score at 30 days. The covariates included in this analysis will be the baseline value for the WHODAS Disability score, along with the fixed terms of study arm, stratification factors (cancer surgery), and prespecified covariates (age, sex, surgery type, malnutrition risk, frailty score). To account for the multicentre trial design, with stratification by centre, a random effect of centre will be used. The model will be estimated using Restricted Maximum Likelihood. The intervention effect will be reported as an adjusted mean difference with 97.5% CI.

9.2 Secondary Effectiveness Analyses

9.2.1 Secondary Analyses of Primary Effectiveness Endpoint

Secondary repeated measures of all WHODAS disability scores (up to 365 days) will employ restricted maximum likelihood estimation. These analyses will also consider the correlation between the four repeated measurements over time by modeling the covariance matrix. The best-fitting model will be selected using information criteria. To account for differences between the groups at baseline, the model will incorporate fixed terms for time, an interaction between time and group, along with the specified covariates and the random center effect. The difference between the treatment and control arms at the 90-day and 365-day marks will be estimated using adjusted least square mean differences along with their associated confidence intervals.

9.2.2 Analyses of Secondary Endpoints

All analyses of secondary outcomes will take into consideration the stratification factors and the specified covariates from the primary analysis, along with accounting for center effects. Health-related quality of life (HRQoL) and 5STS will undergo the same analytic approach as disability scores. Linear regression will be employed for analyzing step counts, while Cox regression will be used to analyse time to hospital discharge, with in-hospital mortality as a competing risk. The marginal probability of the competing events will be estimated using Cumulative Incidence Functions and statistically compared using subdistributional hazard functions. Overall survival will also be analyzed using Cox regression. Discharge disposition and complication severity will be analysed using ordinal logistic regression. Health system outcomes such as readmissions, emergency department visits, and subsequent long-term care admissions will be analysed with logistic regression. Binary safety outcomes will follow a similar approach as complications, with potential adjustments for small event numbers using exact methods. Differences in costs of care will be analyzed using generalized linear methods with log link and gamma distribution.

9.3 Exploratory Effectiveness Analyses

Additional analyses will involve a per-protocol analysis, focusing on individuals who underwent their planned surgery, had at least 3 weeks of enrollment in the intervention arm and completed more than 75% of the prescribed exercise sessions as the per-protocol population. Primary outcomes will be examined in pre-defined subgroups, that we hypothesize may exhibit different responses to the intervention, including sex, age (<75 vs. ≥75), cancer diagnosis, and frailty level (4 vs. ≥ 5). We will also compare compliance rates between sexes by incorporating interaction terms between subgroup indicators and the intervention.

Using the same regression modelling approach as the primary analysis of the WHODAS score, for individuals who did not have their planned surgery within 84 days of allocation, but who went on to have their planned surgery within 114 days of allocation, an exploratory effectiveness analysis will be conducted replacing their WHODAS score at day 114 with the WHODAS score recorded 30 days after their delayed surgery.

Using the same regression modelling approach as the primary analysis of complications, a sensitivity analysis will be performed for the POMS outcome, where: (1) individuals who do not have surgery are assigned a POMS complication status of ‘no complication’; (2) individuals who had their planned surgery more than 84 days after allocation, but on or before day 114 post-allocation are assigned a POMS complication status based on their hospitalization for their delayed surgery; or (3) those who die prior to surgery are assigned a POMS complication status of ‘complication present’.

10 Safety Analyses

On the last day before surgery, patients in both arms will be asked if they have experienced any of the following adverse events (AEs) between randomization and surgery: (1) fall, (2) serious musculoskeletal (MSK) injury, (3) head injury, (4) emergency room visit, or (5) hospital admission. These outcomes will be analysed using Poisson regression or exact methods if event numbers are small. This analysis will be used to compare the incidence of falls, serious injuries and unexpected health care visits between the control and intervention arms.

For individuals in the intervention arm, the following intervention-attributable AEs will be measured: (1) light-headedness, (2) dizziness, (3) mild shortness of breath (SoB), (4) muscle pain, (5) mild worsening of pre-existing health conditions, and (6) other. The proportion of AEs and 95% CIs experienced by intervention arm participants will be reported. These AEs will be identified and rated using the following standard assessment:

Table 3. Standard Assessment for Adverse Events
**Severity**<br>(1=Mild, 2=Moderate, 3=Severe)
**Deemed Serious?**<br>(No=1,<br>If Yes, assign code: 1=Death, or 2=Life-threatening, or 3=Hospitalization (initial or prolonged), or 4=Disability or incapacity, or 5=Required intervention to prevent permanent impairment, 6=Other (as per PI))
**Unexpected as per Protocol?**<br>(0=Expected (light-headed, dizzy, mild SoB, mild, muscle pain, pre-existing health conditions); 1=Unexpected)
**Causality/Relatedness**<br>(1=Definite (clearly related to intervention), 2=Probable (likely related to intervention), 3=Possible (potentially related to intervention), 4=Unlikely (improbably but not impossible related to intervention), 5=Unrelated (clearly not related to intervention), or 6=Unable to determine at this time)

11 Cost-Effectiveness Data

From the perspective of Canada's healthcare system, we will conduct a cost-utility analysis to evaluate whether exercise prehabilitation represents a cost-effective approach. Data on healthcare utilization and the

effectiveness of the intervention will be derived from the trial. Micro costing will capture intervention-specific costs. The results of the cost-effectiveness analysis will be reported in the future, not part of the primary report.

12 Reporting Conventions

P-values will be reported to 3 decimal places (p-values less than 0.001 will be reported as <0.001). The mean, standard deviation, and any other statistics other than quantiles, will be reported to one decimal place greater than the original data. Quantiles, such as median, or minimum and maximum will use the same number of decimal places as the original data. Estimated parameters, not on the same scale as raw observations (e.g., regression coefficients) will be reported to 3 significant figures.

13 Summary of Changes to the Protocol and/or SAP

In the protocol, centre was listed as both a fixed and random effect in the analysis. To resolve this issue, we determined that a random center effect would be the most appropriate method[23]. In addition, we have clarified how intercurrent events will be handled and revised the wording of the objective in order to adhere with the new guidance regarding estimands[19]. Furthermore, changes were made to the sample size (it has increased from 750 to 850) to accommodate the strategies for dealing with intercurrent events. Lastly, due to changes related to the COVID-19 pandemic, method of consent (telephone vs. in-person) was done only by phone for the last 830 patients, so this will not be adjusted for in the statistical models.

14 References

1. McIsaac DI, Fergusson DA, Khadaroo R, et al. PREPARE trial: a protocol for a multicentre randomised trial of frailty-focused preoperative exercise to decrease postoperative complication rates and disability scores. *BMJ Open*. 2022;12(8):e064165. doi:10.1136/bmjopen-2022-064165

2. McIsaac DI, Taljaard M, Bryson GL, et al. Frailty as a Predictor of Death or New Disability After Surgery: A Prospective Cohort Study. *Ann Surg*. 2020;271(2):283. doi:10.1097/SLA.0000000000002967

3. Rockwood K. A global clinical measure of fitness and frailty in elderly people. *Can Med Assoc J*. 2005;173(5):489-495. doi:10.1503/cmaj.050051

4. Fried LP, Ferrucci L, Darer J, Williamson JD, Anderson G. Untangling the Concepts of Disability, Frailty, and Comorbidity: Implications for Improved Targeting and Care. *J Gerontol Ser A*. 2004;59(3):M255-M263. doi:10.1093/gerona/59.3.M255

5. McIsaac DI, Wong CA, Huang A, Moloo H, van Walraven C. Derivation and validation of a generalizable preoperative frailty index using population-based health administrative data. *Ann Surg*. 2019;270(1):102-108.

6. McIsaac DI, Bryson GL, van Walraven C. Association of Frailty and 1-Year Postoperative Mortality Following Major Elective Noncardiac Surgery: A Population-Based Cohort Study. *JAMA Surg*. 2016;151(6):538-545. doi:10.1001/jamasurg.2015.5085

7. McIsaac DI, Moloo H, Bryson GL, van Walraven C. The Association of Frailty With Outcomes and Resource Use After Emergency General Surgery: A Population-Based Cohort Study. *Anesth Analg*. 2017;124(5):1653-1661. doi:10.1213/ANE.0000000000001960

8. Kim DH, Kim CA, Placide S, Lipsitz LA, Marcantonio ER. Preoperative Frailty Assessment and Outcomes at 6 Months or Later in Older Adults Undergoing Cardiac Surgical Procedures. *Ann Intern Med*. 2016;165(9):650-660. doi:10.7326/M16-0652

9. Lin HS, Watts JN, Peel NM, Hubbard RE. Frailty and post-operative outcomes in older surgical patients: a systematic review. *BMC Geriatr*. 2016;16(1):157. doi:10.1186/s12877-016-0329-8

10. Wang J, Zou Y, Zhao J, et al. The Impact of Frailty on Outcomes of Elderly Patients After Major Vascular Surgery: A Systematic Review and Meta-analysis. *Eur J Vasc Endovasc Surg*. 2018;56(4):591-602. doi:10.1016/j.ejvs.2018.07.012

11. Aucoin SD, Hao M, Sohi R, et al. Accuracy and Feasibility of Clinically Applied Frailty Instruments before Surgery: A Systematic Review and Meta-analysis. *Anesthesiology*. 2020;133(1):78-95. doi:10.1097/ALN.0000000000003257

12. Alvarez-Nebreda ML, Bentov N, Urman RD, et al. Recommendations for Preoperative Management of Frailty from the Society for Perioperative Assessment and Quality Improvement (SPAQI). *J Clin Anesth*. 2018;47:33-42. doi:10.1016/j.jclinane.2018.02.011

13. McIsaac DI, Gill M, Boland L, et al. Prehabilitation in adult patients undergoing surgery: an umbrella review of systematic reviews. *Br J Anaesth*. 2022;128(2):244-257. doi:10.1016/j.bja.2021.11.014

14. McIsaac DI, Jen T, Mookerji N, Patel A, Lalu M. Interventions to improve the outcomes of frail people having surgery: a systematic review. *PLOS ONE*. 2017;12(e0190071). Accessed September 19, 2023. https://onlinelibrary.wiley.com/doi/full/10.1111/aas.13239?casa_token=6GF-Aog4igcAAAAA%3AAtj1jOXnAeq9O6loIEJ4jtFnPotNOrPQzqQ9eX4Klq5bCDOKnc9J5xVem9yjBOsykPTrBs8_o_11mQ

15. Milder DA, Pillinger NL, Kam PCA. The role of prehabilitation in frail surgical patients: A systematic review. *Acta Anaesthesiol Scand*. 2018;62(10):1356-1366. doi:10.1111/aas.13239

16. Whitney SL, Wrisley DM, Marchetti GF, Gee MA, Redfern MS, Furman JM. Clinical Measurement of Sit-to-Stand Performance in People With Balance Disorders: Validity of Data for the Five-Times-Sit-to-Stand Test. *Phys Ther*. 2005;85(10):1034-1045. doi:10.1093/ptj/85.10.1034

17. Katz S, Ford AB, Moskowitz RW, Jackson BA, Jaffe MW. STUDIES OF ILLNESS IN THE AGED. THE INDEX OF ADL: A STANDARDIZED MEASURE OF BIOLOGICAL AND PSYCHOSOCIAL FUNCTION. *JAMA*. 1963;185:914-919. doi:10.1001/jama.1963.03060120024016

18. Pleyer L, Heibl S, Tinchon C, et al. Health-Related Quality of Life as Assessed by the EQ-5D-5L Predicts Outcomes of Patients Treated with Azacitidine—A Prospective Cohort Study by the AGMT. *Cancers*. 2023;15(5):1388. doi:10.3390/cancers15051388

19. International Council for Harmonisation (ICH). Addendum on estimands and sensitivity analysis in clinical trials to the guideline on statistical principles for clinical trials E9 (R1). *Fed Regist*. Published online 2019:1-19.

20. McIsaac DI, Hladkowicz E, Bryson GL, et al. Home-based prehabilitation with exercise to improve postoperative recovery for older adults with frailty having cancer surgery: the PREHAB randomised clinical trial. *Br J Anaesth*. 2022;129(1):41-48. doi:10.1016/j.bja.2022.04.006

21. Watt J, Tricco AC, Talbot-Hamon C, et al. Identifying Older Adults at Risk of Delirium Following Elective Surgery: A Systematic Review and Meta-Analysis. *J Gen Intern Med*. 2018;33(4):500-509. doi:10.1007/s11606-017-4204-x

22. Shulman MA, Kasza J, Myles PS. Defining the Minimal Clinically Important Difference and Patient-acceptable Symptom State Score for Disability Assessment in Surgical Patients. *Anesthesiology*. 2020;132(6):1362-1370. doi:10.1097/ALN.0000000000003240

23. Kahan BC. Accounting for centre-effects in multicentre trials with a binary outcome – when, why, and how? *BMC Med Res Methodol*. 2014;14(1):20. doi:10.1186/1471-2288-14-20

24. Liu Y, De A. Multiple Imputation by Fully Conditional Specification for Dealing with Missing Data in a Large Epidemiologic Study. *Int J Stat Med Res*. 2015;4(3):287-295. doi:10.6000/1929-6029.2015.04.03.7

25. Muller CJ, MacLehose RF. Estimating predicted probabilities from logistic regression: different methods correspond to different target populations. *Int J Epidemiol*. 2014;43(3):962-970. doi:10.1093/ije/dyu029

15 Listing of Tables and Figures

Table 1: Baseline characteristics by allocation

Table 2: Primary and secondary outcomes (Intervention/Control/Adjusted effect measure/P-value

Figure 1: CONSORT diagram

Figure 2 : Forest plot of subgroup estimates for the two primary outcomes
