# Supplementary material for: Home-Based Prehabilitation for Older Surgical Patients With Frailty: A Randomized Clinical Trial
Source: JAMA Surg. 2025 Dec 3;161(2):113–23. doi: 10.1001/jamasurg.2025.5288 (PMC12676472; doi:10.1001/jamasurg.2025.5288)
Supplement: Supplement 3. — eFigure 1. PRagmatic Explanatory Continuum Indicator Summary (PRECIS)-2 eTable 1. Reporting Checklist (CONSORT With Patient-Reported Outcomes and Pragmatic Extensions) eTable 2. Reporting Checklist (TIDieR) eMethods 1. Patient Engagement (Description & GRIPP-2) eMethods 2. PREPARE Prehabilitation Program eMethods 3. Description of Adherence Optimization Coaching Strategies eMethods 4. Expanded Rationale for Sample Size Increase eMethods 5. Theoretical Domains Framework Survey eMethods 6. Statistical Analysis of Secondary Outcomes eMethods 7. Changes in Activity Levels eTable 3. Baseline Characteristics of Modified Intention to Treat Population eTable 4. Missing Data eTable 5. Baseline Characteristics of Adherent Population eTable 6. Incidence of Safety Outcomes From Randomization to Surgery eMethods 8. Patient Reported Barriers to Prehabilitation Adherence eFigure 2. Patient-Reported Barriers to Prehabilitation Adherence eTable 7. Interaction P Values for Subgroup Effect Modifier Analyses eTable 8. Complication Subtypes Within Primary Outcome eFigure 3. World Health Organization Disability Assessment Schedule Domain Scores at Baseline and 30 Days eTable 9. Results of Per-Protocol Analyses at 30-Day Follow-Up eTable 10. Results of Per-Protocol Analyses for Hospitalization Outcomes [file jamasurg-e255288-s003.pdf]

Supplemental Online Content

McIsaac DI, Lee S, Fergusson D, et al; PREPARE Trial Investigator Group. Home-based prehabilitation for older surgical patients with frailty: a randomized clinical trial. *JAMA Surg*. Published online December 3, 2025. doi:10.1001/jamasurg.2025.5288

eFigure 1. PRagmatic Explanatory Continuum Indicator Summary (PRECIS)-2

eTable 1. Reporting Checklist (CONSORT With Patient-Reported Outcomes and Pragmatic Extensions)

eTable 2. Reporting Checklist (TIDieR)

eMethods 1. Patient Engagement (Description & GRIPP-2)

eMethods 2. PREPARE Prehabilitation Program

eMethods 3. Description of Adherence Optimization Coaching Strategies

eMethods 4. Expanded Rationale for Sample Size Increase

eMethods 5. Theoretical Domains Framework Survey

eMethods 6. Statistical Analysis of Secondary Outcomes

eMethods 7. Changes in Activity Levels

eTable 3. Baseline Characteristics of Modified Intention to Treat Population

eTable 4. Missing Data

eTable 5. Baseline Characteristics of Adherent Population

eTable 6. Incidence of Safety Outcomes From Randomization to Surgery

eMethods 8. Patient Reported Barriers to Prehabilitation Adherence

eFigure 2. Patient-Reported Barriers to Prehabilitation Adherence

eTable 7. Interaction *P* Values for Subgroup Effect Modifier Analyses

eTable 8. Complication Subtypes Within Primary Outcome

eFigure 3. World Health Organization Disability Assessment Schedule Domain Scores at Baseline and 30 Days

© 2025 McIsaac DI et al; PREPARE Trial Investigator Group. *JAMA Surgery*.

eTable 9. Results of Per-Protocol Analyses at 30-Day Follow-Up

eTable 10. Results of Per-Protocol Analyses for Hospitalization Outcomes

This supplemental material has been provided by the authors to give readers additional information about their work.

© 2025 McIsaac DI et al; PREPARE Trial Investigator Group. *JAMA Surgery*.

eFigure 1. PRagmatic Explanatory Continuum Indicator Summary (PRECIS)-2

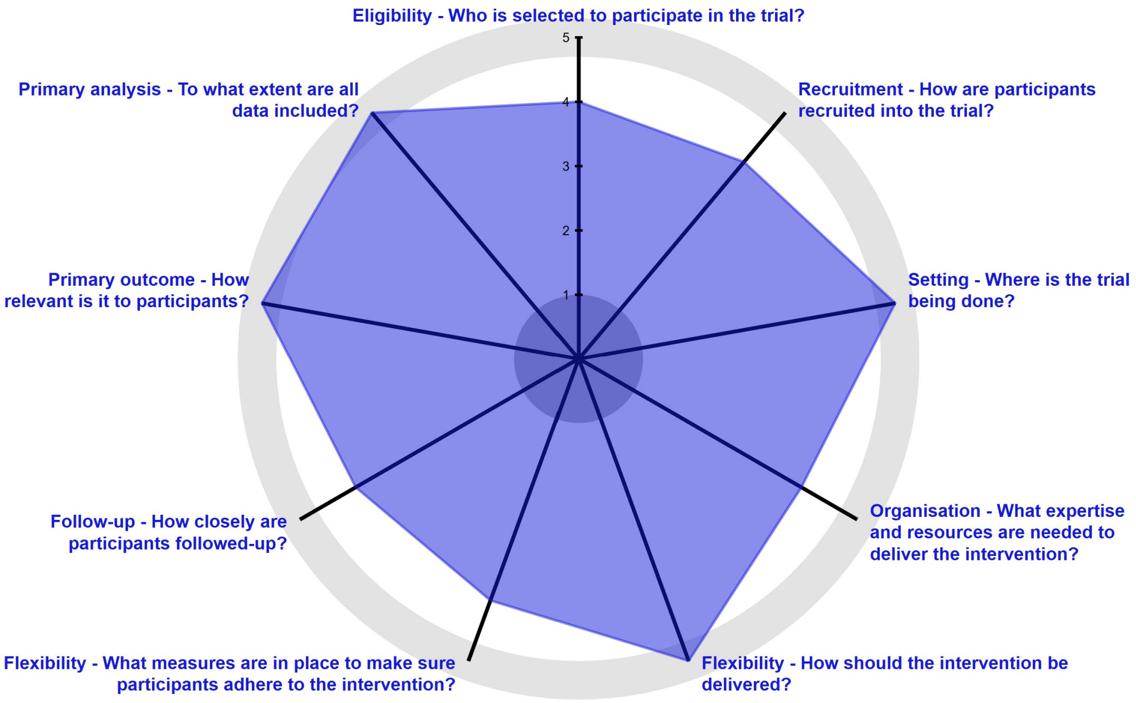

© 2025 McIsaac DI et al; PREPARE Trial Investigator Group. *JAMA Surgery*.

eTable 1. Reporting Checklist (CONSORT With Patient-Reported Outcomes and Pragmatic Extensions)

© 2025 McIsaac DI et al; PREPARE Trial Investigator Group. *JAMA Surgery*.

PRECIS-2 Domain	Description	Score	Justification
Eligibility	Who is selected to participate in the trial?	4	All participants having an eligible surgery, identified with frailty using a guideline-recommended and clinically feasible frailty instrument. While many surgical procedures are included, not all types of inpatient surgeries are eligible
Recruitment	How are participants recruited into the trial?	4	Patients are recruited from surgeons' offices. No extra visits or contacts are required. Clinic lists are screened by research staff to identify potential candidates
Setting	Where is the trial being done?	5	The trial is conducted at 13 different hospitals across Canada, including academic and community centers.
Organisation	What expertise and resources are needed to deliver the intervention?	4	The intervention is delivered as a home-based program, with the coaching support that would be required in a real-world setting. Materials are delivered by courier, so no extra patient contacts or visits are required.
Flexibility: delivery	How should the intervention be delivered?	5	The intervention is delivered as it would be in real world settings. Duration is based on expected surgical wait but is flexible based on actual surgical booking date. Participation has no impact on surgical scheduling. All non-prehabilitation care is at the discretion of treating clinicians.
Flexibility: adherence	What measures are in place to make sure participants adhere to the intervention?	4	Adherence optimization strategies are integrated into the program and into weekly coaching calls. Where adherence is imperfect in a given week, coaches leverage theory-based strategies to improve adherence.
Follow-up	How closely are participants followed-up?	4	No extra patient visits are required. One co-primary, patient-reported outcome (disability) is collected by telephone. In person visits by research staff during hospitalization are used to identify complications, including medical record review.
Primary outcome	How relevant is it to participants?	5	Co-primary outcomes are highly relevant to participants. Patient-reported disability is highly rated by participants and reflects each participant's experience of recovery after surgery. Complications are identified by older surgical patients as their most highly prioritized outcome in surgical research.

© 2025 McIsaac DI et al; PREPARE Trial Investigator Group. *JAMA Surgery*.

Primary analysis	To what extent are all data included?	5	All randomized participants at risk of a given outcome are included in relevant intention to treat analyses, regardless of adherence or other behaviors during the study. For patient-reported disability and non-hospitalization outcomes, this means that all participants, even those who did not have their planned surgeries are included (as even those not having surgery could benefit from prehabilitation or theoretically experience harm). For complications and other hospitalization outcomes, all randomized participants who had surgery are included, as only people who had surgery are at risk of a hospitalization outcome.

© 2025 McIsaac DI et al; PREPARE Trial Investigator Group. *JAMA Surgery*.

eTable 2. Reporting Checklist (TIDieR)

Section/topic	No	CONSORT 2025 checklist item description	Reported on page no.
**Title and abstract**			
Title and structured abstract	1a	Identification as a randomised trial	1
	1b	Structured summary of the trial design, methods, results, and conclusions	3
**Open science**			
Trial registration	2	Name of trial registry, identifying number (with URL) and date of registration	4
Protocol and statistical analysis plan	3	Where the trial protocol and statistical analysis plan can be accessed	6
Data sharing	4	Where and how the individual de-identified participant data (including data dictionary), statistical code and any other materials can be accessed	NA
Funding and conflicts of interest	5a	Sources of funding and other support (eg, supply of drugs), and role of funders in the design, conduct, analysis and reporting of the trial	6
	5b	Financial and other conflicts of interest of the manuscript authors	
**Introduction**			
Background and rationale	6	Scientific background and rationale	5
Objectives	7	Specific objectives related to benefits and harms	5
**Methods**			
Patient and public involvement	8	Details of patient or public involvement in the design, conduct and reporting of the trial	6 & Appendix 2
Trial design	9	Description of trial design including type of trial (eg, parallel group, crossover), allocation ratio, and framework (eg, superiority, equivalence, non-inferiority, exploratory)	6
Changes to trial protocol	10	Important changes to the trial after it commenced including any outcomes or analyses that were not prespecified, with reason	Sample size 9 & Appendix 6
Trial setting	11	Settings (eg, community, hospital) and locations (eg, countries, sites) where the trial was conducted	6 to 8
Eligibility criteria	12a	Eligibility criteria for participants	6
	12b	If applicable, eligibility criteria for sites and for individuals delivering the interventions (eg, surgeons, physiotherapists)	NA

© 2025 McIsaac DI et al; PREPARE Trial Investigator Group. *JAMA Surgery*.

Intervention and comparator	13	Intervention and comparator with sufficient details to allow replication. If relevant, where additional materials describing the intervention and comparator (eg, intervention manual) can be accessed	7 to 8 and Appendices 3 & 4
Outcomes	14	Prespecified primary and secondary outcomes, including the specific measurement variable (eg, systolic blood pressure), analysis metric (eg, change from baseline, final value, time to event), method of aggregation (eg, median, proportion), and time point for each outcome	8 to 9
Harms	15	How harms were defined and assessed (eg, systematically, non-systematically)	8 to 9
	16a	How sample size was determined, including all assumptions supporting the sample size calculation	9
Sample size	16b	Explanation of any interim analyses and stopping guidelines	NA
Randomisation:			
	17a	Who generated the random allocation sequence and the method used	7
Sequence generation	17b	Type of randomisation and details of any restriction (eg, stratification, blocking and block size)	6 to 7
Allocation concealment mechanism	18	Mechanism used to implement the random allocation sequence (eg, central computer/telephone; sequentially numbered, opaque, sealed containers), describing any steps to conceal the sequence until interventions were assigned	6 to 7
Implementation	19	Whether the personnel who enrolled and those who assigned participants to the interventions had access to the random allocation sequence	6 to 7
	20a	Who was blinded after assignment to interventions (eg, participants, care providers, outcome assessors, data analysts)	6 to 7
Blinding	20b	If blinded, how blinding was achieved and description of the similarity of interventions	6 to 8
	21a	Statistical methods used to compare groups for primary and secondary outcomes, including harms	10 to 11
	21b	Definition of who is included in each analysis (eg, all randomised participants), and in which group	10
	21c	How missing data were handled in the analysis	10
Statistical methods	21d	Methods for any additional analyses (eg, subgroup and sensitivity analyses), distinguishing prespecified from post hoc	10 to 11
**Results**			
Participant flow, including flow diagram	22a	For each group, the numbers of participants who were randomly assigned, received intended intervention, and were analysed for the primary outcome	Figure 1
	22b	For each group, losses and exclusions after randomisation, together with reasons	Figure 1
Recruitment	23a	Dates defining the periods of recruitment and follow-up for outcomes of benefits and harms	12

© 2025 McIsaac DI et al; PREPARE Trial Investigator Group. *JAMA Surgery*.

	23b	If relevant, why the trial ended or was stopped	NA
Intervention and comparator delivery	24a	Intervention and comparator as they were actually administered (eg, where appropriate, who delivered the intervention/comparator, how participants adhered, whether they were delivered as intended (fidelity))	11, Appendix 9
	24b	Concomitant care received during the trial for each group	8
Baseline data	25	A table showing baseline demographic and clinical characteristics for each group	T1, Appendix 8
Numbers analysed, outcomes and estimation	26	For each primary and secondary outcome, by group:<br>● the number of participants included in the analysis<br>● the number of participants with available data at the outcome time point<br>● result for each group, and the estimated effect size and its precision (such as 95% confidence interval)<br>● for binary outcomes, presentation of both absolute and relative effect size	13 to 14
Harms	27	All harms or unintended events in each group	12
Ancillary analyses	28	Any other analyses performed, including subgroup and sensitivity analyses, distinguishing pre-specified from post hoc	NA
**Discussion**			
Interpretation	29	Interpretation consistent with results, balancing benefits and harms, and considering other relevant evidence	14 to 16
Limitations	30	Trial limitations, addressing sources of potential bias, imprecision, generalisability, and, if relevant, multiplicity of analyses	16

CONSORT-PRO Patient-reported outcome extension items

1b-Page 4

2a-Pages 6-7

2b-Page 7

4a- N/A

6a-Page 10

© 2025 McIsaac DI et al; PREPARE Trial Investigator Group. *JAMA Surgery*.

7a-Page 11, eMethods

12a-Page 13

15-Table 1

16-Page 16

17a-eTable 8

18-Page 16

20-Page 19

21-Page 18

22-N/A

CONSORT Pragmatic trials extension items

2-Page 6

3-Page 8

4-Page 9, eMethods

6-Page 10

7-Page 11, eMethods

11-Page 10

13-Figure 1

22-Page 17-20

© 2025 McIsaac DI et al; PREPARE Trial Investigator Group. *JAMA Surgery*.

eTable 2. Reporting Checklist (TIDieR)

The TIDieR (Template for Intervention Description and Replication) Checklist				
**Section**	**Item #**	**Item Description**	**Primary paper (page/appendix)**	**Other details**
**BRIEF NAME**	1	Provide the name or a phrase that describes the intervention.	1,3, 7	
**WHY**	2	Describe any rationale, theory, or goal of the elements essential to the intervention.	3, 7	Appendix 3, 4
**WHAT**				
**Materials**	3	Describe any physical or informational materials used in the intervention, including those provided to participants or used in intervention delivery or in training of intervention providers. Provide information on where the materials can be accessed (e.g. online appendix, URL).	7	Appendix 3, 4
**Procedures**	4	Describe each of the procedures, activities, and/or processes used in the intervention, including any enabling or support activities.	7 to 8	Appendix 3, 4
**WHO PROVIDED**	5	For each category of intervention provider (e.g. psychologist, nursing assistant), describe their expertise, background and any specific training given.	7	Appendix 3, 4
**HOW**	6	Describe the modes of delivery (e.g. face-to-face or by some other mechanism, such as internet or telephone) of the intervention and whether it was provided individually or in a group.	7 to 8	Appendix 3, 4
**WHERE**	7	Describe the type(s) of location(s) where the intervention occurred, including any necessary infrastructure or relevant features.	7 to 8	Appendix 3, 4
**WHEN and HOW MUCH**	8	Describe the number of times the intervention was delivered and over what period of time including the number of sessions, their schedule, and their duration, intensity or dose.	7	Appendix 3
**TAILORING**	9	If the intervention was planned to be personalised, titrated or adapted, then describe what, why, when, and how.	7	Appendix 3

© 2025 McIsaac DI et al; PREPARE Trial Investigator Group. *JAMA Surgery*.

**MODIFICATIONS**	10	If the intervention was modified during the course of the study, describe the changes (what, why, when, and how).	NA	NA
**HOW WELL**				
**Planned**	11	If intervention adherence or fidelity was assessed, describe how and by whom, and if any strategies were used to maintain or improve fidelity, describe them.	7 to 8	Appendix 3, 4
**Actual**	12	If intervention adherence or fidelity was assessed, describe the extent to which the intervention was delivered as planned.	12	Appendix 9

© 2025 McIsaac DI et al; PREPARE Trial Investigator Group. *JAMA Surgery*.

eMethods 1. Patient Engagement (Description & GRIPP-2)

Our trial was informed by patient-centered and community-centered priorities for research, and used an integrated knowledge translation approach where we partnered with patients and knowledge users from conception of our protocol. Priorities for perioperative research identified through James Lind Alliance partnerships were also addressed, including: (1) improving the care of older people having surgery; (2) the role of exercise in improving surgical outcomes; (3) the role of exercise in managing frailty and (4) improving home-based care for older people with frailty. Our trial was led by an executive committee that included a patient partner with lived experience caring for an older surgical patient with frailty. Our patient partner helped to inform the definition of our intention to treat population (i.e., all randomized participants), as we postulated that prehabilitation could improve patient-reported disability status even if planned surgeries did not occur, and our modified intention to treat population (i.e., all randomized participants who had surgery), as only individuals who had surgery could experience a postoperative complication or hospitalization-specific outcome.

Guidance for Reporting Involvement of Patients and the Public (GRIPP)-2 Checklist

**Section and topic**	**Reported on page #**
1: Abstract of paper	
1a: Aim	3
1b: Methods	3
1c: Results	3
1d: Conclusions	3
1e: Keywords	1
2: Background to paper	
2a: Definition	5
2b: Theoretical underpinnings	5
2c: Concepts and theory development	5
3: Aims of paper	
3: Aim	5
4: Methods of paper	
4a: Design	6
4b: People involved	6
4c: Stages of involvement	6
4d: Level or nature of involvement	6
5: Capture or measurement of PPI impact	
5a: Qualitative evidence of impact	NA
5b: Quantitative evidence of impact	NA
5c: Robustness of measure	NA
6: Economic assessment	
6: Economic assessment	NA
7: Study results	
7a: Outcomes of PPI	8 to 9
7b: Impacts of PPI	8 to 9

© 2025 McIsaac DI et al; PREPARE Trial Investigator Group. *JAMA Surgery*.

7c: Context of PPI	6
7d: Process of PPI	6
7ei: Theory development	NA
7eii: Theory development	NA
7f: Measurement	NA
7g: Economic assessment	NA
8: Discussion and conclusions	
8a: Outcomes	14 to 15
8b: Impacts	16
8c: Definition	NA
8d: Theoretical underpinnings	NA
8e: Context	NA
8f: Process	NA
8g: Measurement and capture of PPI impact	NA
8h: Economic assessment	NA
8i: Reflections/critical perspective	16

© 2025 McIsaac DI et al; PREPARE Trial Investigator Group. *JAMA Surgery*.

eMethods 2. PREPARE Prehabilitation Program

Rationale and overview

The PREPARE intervention is a structured, home-based, multimodal prehabilitation program. The prehabilitation program was developed with kinesiologists, exercise scientists and nutrition experts, and was informed by a protocol with proven efficacy in improving function both for surgical participants without frailty and in adherent participants with frailty. This program allows for tailoring movements to the needs and safety of people with frailty, and integrated specific feedback from participants with frailty. Nutritional advice was personalized based on individual-level malnutrition risk. Structured theory- and evidence-based strategies were used to support participant adherence at program initiation and throughout enrollment.

Program description – exercise component

At the time of enrollment participants were provided teaching on safe performance of prescribed exercises by a kinesiology-trained coach. Their prehabilitation package included a booklet describing the exercises with text and illustrations, instructional videos, a calendar to track progress and a tip sheet for how to overcome common challenges to engaging in exercise. Equipment provided included a resistance band (with instruction for how to increase the resistance by shortening the band) and a pedometer. Equipment was sent by overnight courier immediately after randomization.

Exercise was prescribed as one-hour sessions, performed a minimum of three times per week for at least three weeks up to a maximum of 12 weeks) before surgery, consisting of: 1) strength training; 2) aerobic exercise and 3) flexibility. A kinesiologist worked with each participant to provide individualized approaches to each exercise by tailoring movements so that individuals with pre-existing pain or other physical limitations could complete the program

<u>Strength training</u>: 1 set of 10 repetitions of each exercise: 1) wall push-ups (modified to the individual's level of function; 2) seated row (elastic resistance band); 3) chest fly (elastic resistance band); 4) deltoid lift (elastic resistance band); 5) biceps curls (elastic resistance band); 6) triceps extensions (elastic resistance band); 7) quadricep exercises; 8) hamstring curls; 9) standing calf raises; 10) modified chair-seated abdominal exercises.

<u>Aerobics</u>: Aerobic exercises were tailored to each participant's needs and preferences. If a participant identified walking as a preferred aerobic activity, it was prescribed that they walk for 20 minutes at moderate intensity. After the first week, the individual's average daily step count was used to recommend a 10% increase in daily step count each week. A 10% increase per week was considered to be a safe, meaningful and achievable method to personalize activity goals. If a participant preferred swimming or exercising using a stationary bike, for example, increases were prescribed by increasing the time they engage in their aerobic activity if it suited their comfort and safety levels. Participants were coached to achieve moderate intensity using a Borg scale, which was provided as part of their prehabilitation package. To accommodate the challenges of getting outdoors during winter or inclement weather, and due to COVID-19 restrictions, participants were provided with a video for an in-home aerobic exercise session (standing and seated options) designed to be safe and achievable for older adults.

<u>Flexibility</u>: Chest, arm, leg and trunk stretches; each stretch held for 20 seconds x 2 repetitions.

Program description – nutrition component

Following screening for malnutrition risk using the Canadian Nutrition Screening Tool (CNST), malnutrition risk status was used to guide nutritional advice. The nutrition component was supported by a pamphlet to inform all intervention participants of proper caloric and protein requirements. The pamphlet also included participant-suggested recipes for protein smoothies. Coupons (from a variety of companies with no commercial connection to the trial, its

© 2025 McIsaac DI et al; PREPARE Trial Investigator Group. *JAMA Surgery*.

investigators, or participants) for protein enhanced beverages were provided to all participants in the program. Participants identified as at risk of malnutrition were provided personalized advice regarding achieving individual protein goals from a coach with expertise in nutrition science, which included identification of individual barriers and strategies to overcome identified barriers. For all prehabilitation participants, protein intake and nutritional goals were reviewed during weekly coaching calls.

Coaching

Upon receipt of the study program materials, a trained coach provided exercise teaching and collaboratively discussed individual goals, values and possible challenges with each participant to support motivation, safety and adherence.

Throughout the program, participants were supported by the central team using weekly phone calls to monitor safety, encourage adherence and provide advice on exercise progression and nutritional intake. A single coach was assigned to each participant throughout their time in the program to provide consistency and a longitudinal relationship. Participants were able to call or email the study center at any time with questions or concerns.

© 2025 McIsaac DI et al; PREPARE Trial Investigator Group. *JAMA Surgery*.

eMethods 3. Description of Adherence Optimization Coaching Strategies

Supporting exercise adherence: At each coaching call adherence was assessed by the coach based on participant self-report and review of the program logbook for the previous week. The program included adherence strategies globally applied for all participants, as well as strategies employed for participants who reported less than 100% adherence in a given week. Adherence strategies were based on the Theoretical Domains Framework (TDF: a leading theory focused on health-related behavior change), as well as self-determination theory, which states that competence, autonomy and relatedness promote behavior change. Global strategies aimed to promote competence by emphasizing participant successes; they promoted autonomy by encouraging the participants to schedule their own program and complete the level that is suitable to their abilities and needs; and they promoted relatedness by encouraging participants to find a friend or partner to complete the program with.

Where participants were identified to have achieved less than 100% adherence to their prescribed exercises, TDF-informed strategies were employed to target commonly identified barriers to program participation (e.g., illness severity, weather, guilt or frustration). These strategies were informed by previous qualitative research done with older surgical participants with frailty engaged in prehabilitation in preparation for surgery, as well as through engagement with participant partners. Examples, organized by TDF domain, are provided below. Each coach was oriented to, and trained on, program adherence strategies as part of their coach training.

**Strategies for low adherence**		
**PREPARE Strategies**	**TDF Domain**	**Example**
**Personalization**	Physical Skills	Exercise regressions (modify exercise, split cardio sessions (e.g., 10 mins cardio x2), reduced repetitions)
**Goal setting**	Goals	Review and promote previous week's goal
**Promote self-efficacy**	Beliefs about capabilities	Congratulate on successes, small or big; congratulate on successes, despite what they are going through (illness, fatigue, symptom burden)
**Self-monitoring (calendar)**	Behavioural regulation	Coach can go through week with participant and ask them to circle days they are planning on completing the program
**Discussion of previous successes with exercise**	Optimism	Coach to discuss any previous success the participant has had with exercises to promote self-efficacy
**Education/Instruction**	Knowledge	Answer questions about specific exercises to ensure the participant is completing the movement correctly
**Social support**	Social influences	Coaches recommend finding a partner to exercise with
**Signed Consent Form**	Intentions	Kindly remind the participant they agreed to try to do the exercises at point of consent
**Equipment, materials, and DVDs**	Environmental context and resources	Encourage participants to hang tip sheet on their fridge and refer to it when they need some extra motivation
**Strategies from Tip Sheet: Relaxation strategies (breathing, stretching)**	Emotion	Relaxation strategies such as instruction of deep breathing or square breathing and encouragement of stretches to cope with pain/stress/illness severity
**Strategies from Tip Sheet: Activation strategies (self-talk, music, values)**	Beliefs about consequences	Coach to discuss personal values with participants and what matters to them so that motivation can be derived from these values

© 2025 McIsaac DI et al; PREPARE Trial Investigator Group. *JAMA Surgery*.

eMethods 4. Expanded Rationale for Sample Size Increase

Original sample size

The initial sample size for the PREPARE Trial was 750 participants (375 per arm), was estimated to achieve 90% power to detect a relative difference of 25% in the incidence of the co-primary outcome of complications using an unpooled Z-test with a two-sided alpha level of 0.025. This calculation accounted for a 10% non-compliance factor largely consisting of randomized participants not having their planned surgery, and assumed a control group event rate of 55%. For the continuous co-primary outcome (i.e., WHODAS Disability Score at 30 days) this sample size was estimated to provide 98% power to detect a minimum clinically important difference of 8 points on a 100-point scale using an ANCOVA analysis at the two-sided alpha level of 0.025. This calculation assumed a common standard deviation of 25, a correlation between baseline and postoperative score of 0.4 and accounted for 8% missing data at 30 days and 10% intervention non-compliance. A Bonferroni-correction was applied to maintain the overall type I error rate across the two primary outcomes at 5%, hence the two-sided alpha=0.025 for each co-primary outcome.

Trial executive committee recommends increase in sample size

In early 2023, the trial executive team noted a number of important general and trial-specific considerations that the team, inclusive of patient partners, thought would influence the power of the PREPARE Trial to estimate a meaningful difference between arms. None of these considerations were based on knowledge of trial or group level outcome data, which remained blinded to the team throughout the trial and final analysis by an independent biostatistician.

First, as the trial was conducted during the COVID-19 pandemic (1st patient enrolled in March, 2020), changes and disruptions to surgical scheduling had substantially impacted expected versus actual time to surgery for enrolled participants. Specifically, due to efforts made in Canada to target surgical oncology cases during the post-lockdown recovery periods, oncology surgeries (~75% of PREPARE cases) received expedited, but unpredictable, operating room access. This led to many PREPARE participants having an expected surgical wait of >3 weeks at the time of enrollment, but actually receiving operating room access in <3 weeks from enrollment. This structurally limited potential adherence for patients who achieved earlier OR access than expected. Concurrently, non-oncology cases faced lengthy and often permanent delays, which led to many planned non-oncology participants not having their planned surgery.

Next, after estimation and registration of our original sample size, which included a generic minimally important difference of 8-points on a 100-point scale for the WHODAS (Andrews G, et. al. PLoS ONE, 2009. doi: 10.1371/journal.pone.0008343), a surgery-specific minimally important difference of 5 points on the 100-point scale was published (Shulman et. al. Anesthesiology, 2020. doi: 10.1097/ALN.0000000000003240). Additionally, new data were published that informed the expected common SD for the WHODAS score in this population specifically (20, as opposed to the 25 originally assumed: McIsaac et. al. BJA, 2022. doi: 10.1016/j.bja.2022.04.006). Lastly, as a relatively pragmatic trial, our executive committee re-confirmed that intention to treat populations for each co-primary outcome should include all randomized participants at risk of prespecified outcomes. Specifically, patient-reported and non-hospitalization outcomes (including the co-primary WHODAS) should be analyzed in all randomized participants, while hospitalization outcomes (including the co-primary complications outcome) should be analyzed in all randomized participants who had surgery. Therefore, re-estimated sample size calculations would need to account for different attrition rates for each co-primary outcome.

Key changes to the sample size estimation, which is described in detail in the manuscript, included the following:

<u>WHODAS disability score</u>: target effect size 5 points, common SD 20, 5% attrition.

<u>Incidence of any complication</u>: same target effect size, 15% non-adherence, 15% attrition.

New sample size estimate

A Bonferroni correction (two-sided alpha=0.025) was used to maintain the overall type I error rate across co-primary outcomes at 0.05. With 850 participants, the trial had 90% power to detect a minimum clinically important

© 2025 McIsaac DI et al; PREPARE Trial Investigator Group. *JAMA Surgery*.

absolute mean difference of 5 points on the 100-point WHODAS disability score[33] using an analysis of covariance (ANCOVA), assuming a common standard deviation (SD) of 20 and correlation between baseline and follow-up of 0.4 with 5% attrition. For the co-primary outcome of any complication, we had 90% power to detect an absolute difference of 15% in complications using a pooled Z-test, assuming a control arm complication rate of 0.55 and accounting for 15% non-adherence to the exercise intervention and 15% attrition (including participants not having surgery).

© 2025 McIsaac DI et al; PREPARE Trial Investigator Group. *JAMA Surgery*.

eMethods 5. Theoretical Domains Framework Survey

On the day before surgery, participants in the prehabilitation arm were asked to complete a survey based on a validated survey construct for the Theoretical Domains Framework (Huijg KM, et. al., Implementation Science, 2014. doi: 10.1186/1748-5908-9-11). The survey for PREPARE prehabilitation participants was adapted to the current context based on previous qualitative assessment of barriers and facilitators to prehabilitation for older adults with frailty (Barnes K, et. al. BMC Geriatrics, 2023. doi: 10.1186/s12877-023-03990-3) and input from patient partners to ensure relevant content, clear wording and to minimize respondent burden. Each response was scored on a 5-point Likert scale (questions 10 and 23 were reverse coded for analysis).

Knowledge:

Strongly Disagree	Disagree	Neutral	Agree	Strongly Agree

1. I am aware of the content and goals of the exercise program.
2. I know how to complete the at-home exercise program.

Skills:

Strongly Disagree	Disagree	Neutral	Agree	Strongly Agree

3. I have been trained how to do the exercises in the exercise program.
4. I have the skills and ability to perform the exercises in the program.
5. I have practiced the exercises in the program.

Belief About Capabilities:

Strongly Disagree	Disagree	Neutral	Agree	Strongly Agree

6. I am confident that I can do the exercises in the program.
7. I feel that I have control over the exercises in the program.
8. For me, doing the exercises in the program is easy.

Optimism:

Strongly Disagree	Disagree	Neutral	Agree	Strongly Agree

9. With regard to the exercise program, I usually expect the best.
10. With regard to the exercise program, I hardly ever expect things to go my way.

Belief About Consequences:

© 2025 McIsaac DI et al; PREPARE Trial Investigator Group. *JAMA Surgery*.

Strongly Disagree	Disagree	Neutral	Agree	Strongly Agree

11. For me, completing the exercise program is useful.
12. If I complete the exercise program it will benefit my health.

Reinforcement:

Strongly Disagree	Disagree	Neutral	Agree	Strongly Agree

13. Whenever I do the exercise program, I get acknowledgement from people who are important to me.
14. If I do the exercise program, I feel like I am doing something important.

Goals:

Strongly Disagree	Disagree	Neutral	Agree	Strongly Agree

15. Generally, other things in my life were a higher priority than completing the exercise program.

Memory, Attention and Decisional Processes:

Strongly Disagree	Disagree	Neutral	Agree	Strongly Agree

16. Doing the exercises in the program was easy to remember.
17. When I concentrate on doing the exercises in the program, I have no trouble focusing my attention.

Environmental Context and Resources:

Strongly Disagree	Disagree	Neutral	Agree	Strongly Agree

18. I have the tools and support needed to complete the exercise program.
19. The exercise program has enough resources to help me to do the exercises.

Social Influences:

Strongly Disagree	Disagree	Neutral	Agree	Strongly Agree

20. Most people who are important to me think that I should do the exercise program.
21. Most people whose opinion I care about would approve me of doing the exercise program.

Emotion:

© 2025 McIsaac DI et al; PREPARE Trial Investigator Group. *JAMA Surgery*.

Never	Rarely	Sometimes	Very Often	Always

22. To what extent do you generally feel inspired to do the exercise program?
23. To what extent do you generally feel nervous about the exercise program?

Behavioral Regulation:

Strongly Disagree	Disagree	Neutral	Agree	Strongly Agree

24. I used my calendar to keep track of my exercise program.
25. I tend to notice my successes while working towards completing the exercise program.

© 2025 McIsaac DI et al; PREPARE Trial Investigator Group. *JAMA Surgery*.

eMethods 6. Statistical Analysis of Secondary Outcomes

In keeping with methods applied to analyses of co-primary outcomes, all secondary outcomes were analyzed using multiple imputation via fully conditional specification under a missing at random assumption to account for any missing data, as well as prespecified adjustment for stratification and baseline prognostic factors: a random intercept for each study site, cancer surgery (vs. not), age, sex, surgery type, malnutrition risk, and frailty score.

Health-related quality of life, health utility and 5-times sit to stand time were analyzed using ANCOVA, via mixed effects linear regression models that included terms for allocation, prespecified stratification and prognostic factors and additional adjustment for each relevant baseline measure. These analyses yielded an adjusted mean difference with 95% confidence interval.

Mixed effects ordinal logistic regression was used for Katz index, discharge disposition and Clavien-Dindo complication severity. These analyses yielded an adjusted generalized odds ratio with 95% confidence interval.

Mixed effects Cox proportional hazards regression was used for overall survival and length of stay (as time to hospital discharge (accounting for death as a competing risk)). These analyses yielded an adjusted hazard ratio with 95% confidence interval.

Mixed effects binary logistic regression was used to compare the proportion of participants with postoperative falls and readmissions. These analyses yielded an adjusted odds ratio with 95% confidence interval.

Step count outcomes were not analyzed as participants in both arms struggled to use provided pedometers and reliably record pedometer outcome (69% missing values in control, 73% missing values in intervention).

© 2025 McIsaac DI et al; PREPARE Trial Investigator Group. *JAMA Surgery*.

eMethods 7. Changes in Activity Levels

Activity levels

Methods

On the day prior to surgery, participants in both trial arms were contacted by telephone and asked to rate their perceived change in physical activity level:

Thinking back to about a month before you enrolled in The PREPARE Trial, how much more would you say you exercised since you joined the trial compared to the month before enrollment?				
No change at all<br>1	Slightly more<br>2	Moderately more<br>3	Significantly more<br>4	Biggest change possible<br>5

Participant responses were descriptively analyzed using medians and interquartile ranges, stratified by allocation group (prehabilitation vs. usual care).

Ratings were compared between arms using ordinal logistic regression, adjusted for stratification factors and baseline factors prespecified to be prognostic: a random intercept for each study site, cancer surgery (vs. not), age, sex, surgery type, malnutrition risk, and frailty score. This analysis yielded a generalized odds ratio with 95% confidence interval.

Results

Control group participants reported no median change (0/5) in physical activity prior to surgery (IQR no change (0/5) to slight change (1/5)), while intervention participants reported a moderate (3/5) increase in physical activity (IQR slight (1/5) to significant (4/5)).

Following adjusted ordinal regression, prehabilitation participants reported an 8-fold increase in the odds that their activity levels were higher versus baseline compared to control (generalized odds ratio (OR) 8.24, 95%CI 6.15 to 11.03; *P*<0.001).

© 2025 McIsaac DI et al; PREPARE Trial Investigator Group. *JAMA Surgery*.

eTable 3. Baseline Characteristics of Modified Intention to Treat Population

Characteristics of the Patients Who Underwent Randomization and Had Planned Surgery*		
**Characteristic**	**Prehabilitation n=353**	**Usual care n=352**
Age at randomization - yr	71.9 (7.0)	71.7 (7.0)
Female sex - no. (%)	199 (56.4)	177 (50.3)
Weeks from enrollment to surgery (median, IQR)	34 (22, 51)	34 (22,49)
Frailty and functional indicators		
Clinical Frailty Scale Score (median, IQR)†	4 (4,4)	4 (4,4)
Duke Activity Status Index Score	33.0 ± 13.3	33.0 ± 13.3
Katz Index (median, IQR)	6 (6,6)	6 (6,6)
Surgery Type - no. (%)		
Colorectal	77 (21.8)	74 (21.0)
Head and neck	9 (2.6)	16 (4.6)
Hepatobiliary	48 (13.6)	37 (10.5)
Thoracic	63 (17.9)	62 (17.6)
Vascular	46 (13.0)	44 (12.5)
Urologic or gynecologic	77 (21.8)	85 (24.2)
Other general surgery	33 (9.4)	34 (9.7)
Coexisting conditions - no. (%)		
History of myocardial infarction	27 (7.7)	37 (10.5)
Congestive heart failure	14 (4.0)	16 (4.6)
History of stroke or transient ischemic attack	29 (8.2)	36 (10.2)
Chronic pulmonary disease	52 (14.7)	64 (18.2)
Diabetes with complications	33 (9.4)	51 (14.5)
Liver disease	33 (9.4)	30 (8.5)
Kidney disease	54 (15.3)	55 (15.6)
Current smoker	49 (11.6)	48 (11.3)
At risk of malnutrition	88 (20.8)	78 (18.4)
Oncologic variables - no. (%)		
Cancer	279 (79.0)	272 (77.3)
Receipt of radiation in the last 6 months	41 (11.6)	44 (12.5)
History of chemotherapy in last 6 months	69 (19.6)	69 (19.6)

Participants represented in this table are those who were randomized and underwent their planned surgery

© 2025 McIsaac DI et al; PREPARE Trial Investigator Group. *JAMA Surgery*.

eTable 4. Missing Data

eTable 4a- Characteristics of the Patients With and Without Missing WHODAS Data*		
**Characteristic**	**Missing n=67**	**Not missing n=780**
Age at randomization - yr	73.4 (7.0)	71.6 (7.0)
Female sex - no. (%)	40 (59.7)	412 (52.8)
Days from enrollment to surgery (median, IQR)	39.5 (25,63)	37 (24, 62)
Frailty and functional indicators		
Clinical Frailty Scale Score (median, IQR)†	4 (4,5)	4 (4,4)
Duke Activity Status Index Score	26.2 ± 14.5	32.9 ± 13.3
Katz Index (median, IQR)	6 (6,6)	6 (6,6)
Surgery Type - no. (%)		
Colorectal	9 (13.4)	172 (22.1)
Head and neck	3 (4.5)	26 (3.3)
Hepatobiliary	15 (22.4)	91 (11.7)
Thoracic	8 (11.9)	131 (16.8)
Vascular	46 (13.0)	44 (12.5)
Urologic or gynecologic	16 (23.9)	181 (23.2)
Other general surgery	6 (9.0)	81 (10.4)
Coexisting conditions - no. (%)		
History of myocardial infarction	6 (9.0)	70 (9.0)
Congestive heart failure	5 (7.5)	30 (3.9)
History of stroke or transient ischemic attack	14 (20.9)	70 (9.0)
Chronic pulmonary disease	10 (14.9)	127 (16.3)
Diabetes with complications	9 (13.4)	87 (11.2)
Liver disease	8 (11.9)	70 (9.0)
Kidney disease	10 (14.9)	123 (15.8)
Current smoker	9 (13.4)	88 (11.3)
At risk of malnutrition	20 (29.9)	146 (18.7)
Oncologic variables - no. (%)		
Cancer	49 (73.1)	592 (75.9)
Receipt of radiation in the last 6 months	3 (4.5)	52 (6.7)
History of chemotherapy in last 6 months	7 (10.5)	96 (12.3)

© 2025 McIsaac DI et al; PREPARE Trial Investigator Group. *JAMA Surgery*.

eTable 4b-Missing Data Prevalence by Allocation for Patients Who Underwent Randomization		
**Characteristic**	**Prehabilitation n=423**	**Usual care n=424**
Age at randomization - yr	0 (0%)	0 (0%)
Female sex - no. (%)	0 (0%)	0 (0%)
Weeks from enrollment to surgery (median, IQR)	0 (0%)	0 (0%)
Frailty and functional indicators		
Clinical Frailty Scale Score (median, IQR)†	0 (0%)	0 (0%)
Duke Activity Status Index Score	0 (0%)	0 (0%)
Katz Index (median, IQR)	0 (0%)	0 (0%)
Surgery Type - no. (%)		
Colorectal	0 (0%)	0 (0%)
Head and neck	0 (0%)	0 (0%)
Hepatobiliary	0 (0%)	0 (0%)
Thoracic	0 (0%)	0 (0%)
Vascular	0 (0%)	0 (0%)
Urologic or gynecologic	0 (0%)	0 (0%)
Other general surgery	0 (0%)	0 (0%)
Coexisting conditions - no. (%)		
History of myocardial infarction	0 (0%)	0 (0%)
Congestive heart failure	0 (0%)	0 (0%)
History of stroke or transient ischemic attack	0 (0%)	0 (0%)
Chronic pulmonary disease	0 (0%)	0 (0%)
Diabetes with complications	0 (0%)	0 (0%)
Liver disease	0 (0%)	0 (0%)
Kidney disease	0 (0%)	0 (0%)
Current smoker	0 (0%)	0 (0%)
At risk of malnutrition		
Oncologic variables - no. (%)		
Cancer	0 (0%)	0 (0%)
Receipt of radiation in the last 6 months	0 (0%)	0 (0%)
History of chemotherapy in last 6 months	0 (0%)	0 (0%)
**30-day Outcomes**	**Prehabilitation n=423**	**Usual care n=424**
WHODAS Disability score	36 (8.5%)	31 (7.3%)
EuroQoL visual analogue scale score	38 (8.9%)	37 (8.7%)
EuroQoL utility index	36 (8.5%)	34 (8.0%)
Death	0 (0%)	0 (0%)
Falls	23 (5.8%)	14 (3.3%)
**Hospital Discharge Outcomes**	**Prehabilitation n=353**	**Usual care n=352**
Any postoperative complication	0 (0%)	0 (0%)
5 Times Sit to Stand	12 (3.4%)	22 (6.3%)

© 2025 McIsaac DI et al; PREPARE Trial Investigator Group. *JAMA Surgery*.

Katz Index of Activities of Daily Living	0 (0%)	0 (0%)
Discharge Disposition	1 (0.3%)	4 (1.1%)
Time to discharge	1 (0.3%)	3 (0.9%
Readmission	3 (0.8%)	10 (2.8%)

© 2025 McIsaac DI et al; PREPARE Trial Investigator Group. *JAMA Surgery*.

eTable 5. Baseline Characteristics of Adherent Population

Characteristics of Per Protocol Adherent vs. Non-Adherent Prehabilitation Patients		
**Characteristic**	**Adherent** n=152**	**Non-Adherent n=201**
Age at randomization - yr	72.0 + 7.4	71.7 + 6.6
Female sex - no. (%)	82 (54.0)	117 (58.2)
Days from enrollment to surgery (median, IQR)	38 (30,52)	27 (21,47)
Frailty and functional indicators		
Clinical Frailty Scale Score (median, IQR)†	4 (4,4)	4 (4,4)
Duke Activity Status Index Score	34.6 (13.2)	31.7 (13.2)
Katz Index (median, IQR)	6 (6,6)	6 (6,6)
Surgery Type - no. (%)		
Colorectal	33 (21.7)	44 (21.9)
Head and neck	3 (2.0)	6 (3.0)
Hepatobiliary	25 (16.5)	23 (11.4)
Thoracic	22 (14.5)	41 (20.4)
Vascular	17 (11.2)	29 (14.4)
Urologic or gynecologic	39 (25.7)	38 (18.9)
Other general surgery	13 (8.6)	20 (10.0)
Coexisting conditions - no. (%)		
History of myocardial infarction	15 (7.5)	12 (6.0)
Congestive heart failure	6 (3.0)	8 (4.0)
History of stroke or transient ischemic attack	9 (4.5)	20 (10.0)
Chronic pulmonary disease	16 (8.0)	36 (17.9)
Diabetes with complications	14 (7.0)	19 (9.5)
Liver disease	17 (8.5)	16 (8.0)
Kidney disease	18 (9.0)	36 (17.9)
Current smoker	11 (5.5)	30 (14.9)
At risk of malnutrition	27 (13.4)	42 (20.9)
Oncologic variables - no. (%)		
Cancer	123 (80.9)	156 (77.6)
Receipt of radiation in the last 6 months	12 (6.0)	11 (5.5)
History of chemotherapy in last 6 months	26 (12.9)	21 (10.5)

*Plus-minus values are means + standard deviation

**Denotes having planned surgery and completing >75% of prescribed exercises for ≥3 weeks prior to surgery

†Clinical Frailty Scale scores were assigned by research staff trained in frailty assessment using a theory and evidence-based online training module

© 2025 McIsaac DI et al; PREPARE Trial Investigator Group. *JAMA Surgery*.

eTable 6. Incidence of Safety Outcomes From Randomization to Surgery

**Incidence of adverse events from randomization to surgery***				
**Adverse event****	**Prehabilitation n=273**	**Usual care n=278**	**Risk difference (95%CI)**	**P-value**
Any fall - no. (%)	11/273 (4.0)	9/278 (3.2)	0.8% (-2.3 to 3.9)	0.62
Any musculoskeletal injury - no. (%)	10/273 (3.7)	4/273 (1.4)	2.2% (-0.04 to 4.9)	0.10
Any hospitalization - no. (%)	11/273 (4.0)	9/278 (3.2)	0.8% (-2.3 to 3.9)	0.62
Any head injury - no. (%)	3/273 (1.1)	1/273 (0.4)	0.7% (-0.7 to 2.2)	0.31

*Group level adverse events were only systematically ascertained for the final 551 participants as this safety indicator was not included in the original protocol.

**Adverse events were ascertained by patient report on the last follow up call on the day prior to surgery.

© 2025 McIsaac DI et al; PREPARE Trial Investigator Group. *JAMA Surgery*.

eMethods 8. Patient Reported Barriers to Prehabilitation Adherence

Methods

For each weekly coaching call, coaches identified whether prehabilitation participants had been 100% adherent to aerobic exercise, and separately strength exercises. Where a given participant's weekly adherence was <100%, the coach determined whether the primary reason for non-adherence was medical, behavioural or other (a coach rated a reason as 'other' if a medical or behavioural explanation was not applicable, so this was a heterogeneous category). With each non-adherent week as the denominator, we then estimated the proportion of reasons for non-adherence using methods appropriate for repeated measures per participant (PROC SURVEYFREQ, with participant number as the cluster variable).

On the day before surgery, prehabilitation participants were asked to complete a validated TDF survey (see Appendix 5 for numerically matched TDF questions). Each of the 25 TDF survey responses were descriptively analyzed to generate medians and interquartile ranges for each response. Any item with a 1st quartile (i.e., lower limit of the interquartile range) value <4 was rated as a strong barrier. This rating was applied post hoc based on the team's review of descriptive data, where consistently high ratings were apparent for 22 of 25 questions. Questions 10 and 23 were reverse coded for analysis.

Results

There were 623 non-adherent participant weeks for aerobic exercises and 1050 non-adherent participant weeks for strength exercises. Reasons for non-adherence along with proportions and 95%CIs are presented in the following tables.

**Aerobic exercise non-adherence**			
**Reason for non-adherence**	**Number of non-adherent weeks**	**%**	**95% CI (%)**
Medical	281	45.1%	36.6 to 53.6
Behavioural	332	53.3%	44.6 to 61.9
Other	10	1.6%	0.1 to 3.1

**Strength exercise non-adherence**			
**Reason for non-adherence**	**Number of non-adherent weeks**	**%**	**95% CI (%)**
Medical	402	38.3%	32.0 – 44.6
Behavioural	627	59.7%	53.3 – 66.1
Other	21	2.0%	0.9 – 3.1

Box and whisker plots for each TDF survey response are provided in eFigure 2 (below).

Strong barriers, with corresponding TDF domain, included:
Question 13-Whenever I do the exercise program, I get acknowledgement from people who are important to me. (Domain: Reinforcement)
Question 15- Generally, other things in my life were a higher priority than completing the exercise program. (Domain: Goals)
Question 22-To what extent do you generally feel inspired to do the exercise program? (Domain: Emotion)

© 2025 McIsaac DI et al; PREPARE Trial Investigator Group. *JAMA Surgery*.

eFigure 2. Patient-Reported Barriers to Prehabilitation Adherence

See eMethods 5 & 8 for wording of specific questions pertaining to each domain and response.

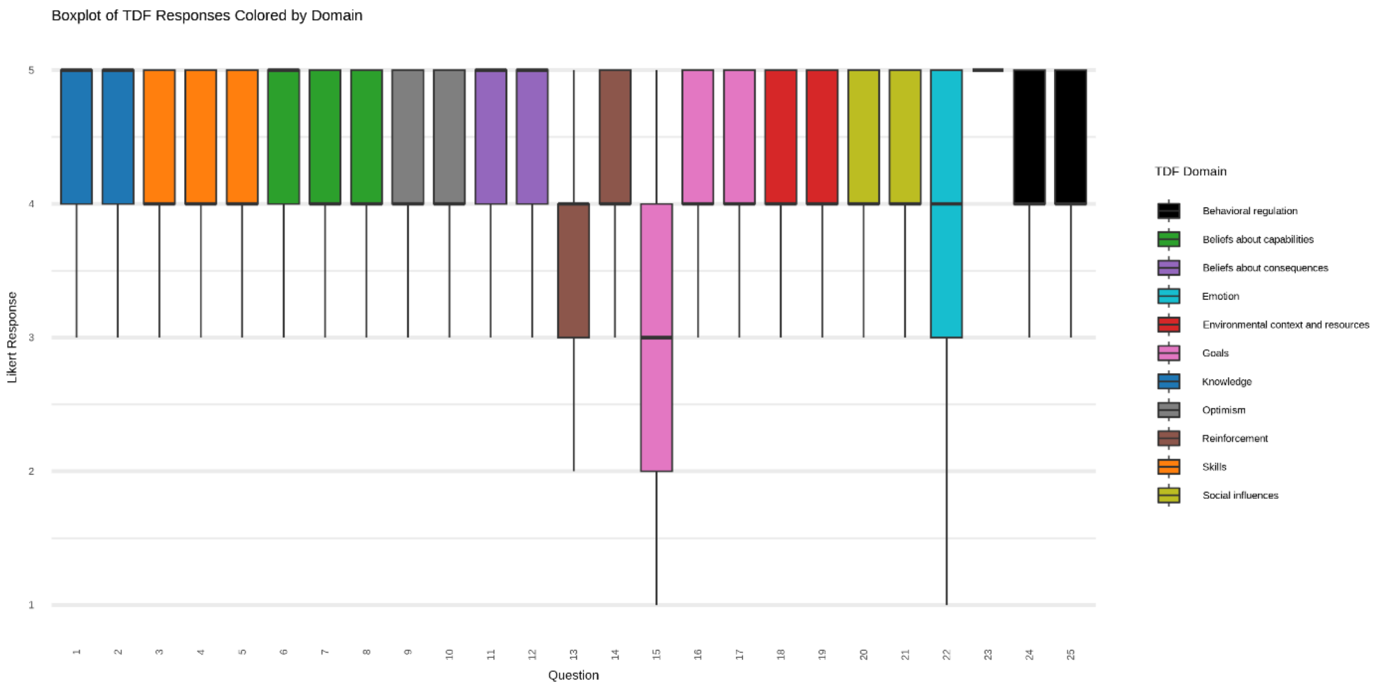

© 2025 McIsaac DI et al; PREPARE Trial Investigator Group. *JAMA Surgery*.

eTable 7. Interaction *P* Values for Subgroup Effect Modifier Analyses

Interaction p-values for subgroup effect modifier analyses	
	**Interaction P-value**
**WHODAS Disability Score**	
Male vs. Female	0.12
<75 years vs. ≥75 years	0.85
Cancer surgery vs. not	0.85
Depression vs. not	0.21
Clinical Frailty Scale 4 vs. ≥5	0.95
Surgery type	0.98
**Any complication**	
Male vs. Female	0.21
<75 years vs. ≥75 years	0.09
Cancer surgery vs. not	0.47
Depression vs. not	0.94
Clinical Frailty Scale 4 vs. ≥5	0.11
Surgery type	0.63

All effect modifiers were prespecified except for surgery type

© 2025 McIsaac DI et al; PREPARE Trial Investigator Group. *JAMA Surgery*.

eTable 8. Complication Subtypes Within Primary Outcome

Incidence of complication subtypes		
Outcome	Prehabilitation n=353	Usual care n=352
Pulmonary - no. %	44/353 (12.5)	45/352 (12.8)
Gastrointestinal - no. %	31/353 (8.9)	28/353 (8.0)
Renal - no. %	60/353 (17.0)	72/352 (20.5)
Cardiovascular - no. %	101/353 (28.6)	81 (352 (23.0)
Wound - no. %	25/353 (7.1)	27/352 (7.7)
Infection - no. %	44/353 (12.5)	53/352 (15.1)
Hematologic - no. %	33/353 (9.4)	32/352 (9.1)
Neurologic - no. %	36/353 (10.2)	44/352 (12.5)

© 2025 McIsaac DI et al; PREPARE Trial Investigator Group. *JAMA Surgery*.

eFigure 3. World Health Organization Disability Assessment Schedule Domain Scores at Baseline and 30 Days

This figure displays the mean and 95%CI for responses in each of the 12 WHODAS domains for participants assigned to prehabilitation and usual care (control) at baseline and at 30-day follow up. Each WHODAS domain is collected on a 5-point Likert scale from 0 (no difficulty) to 4 (extreme difficulty/cannot do).

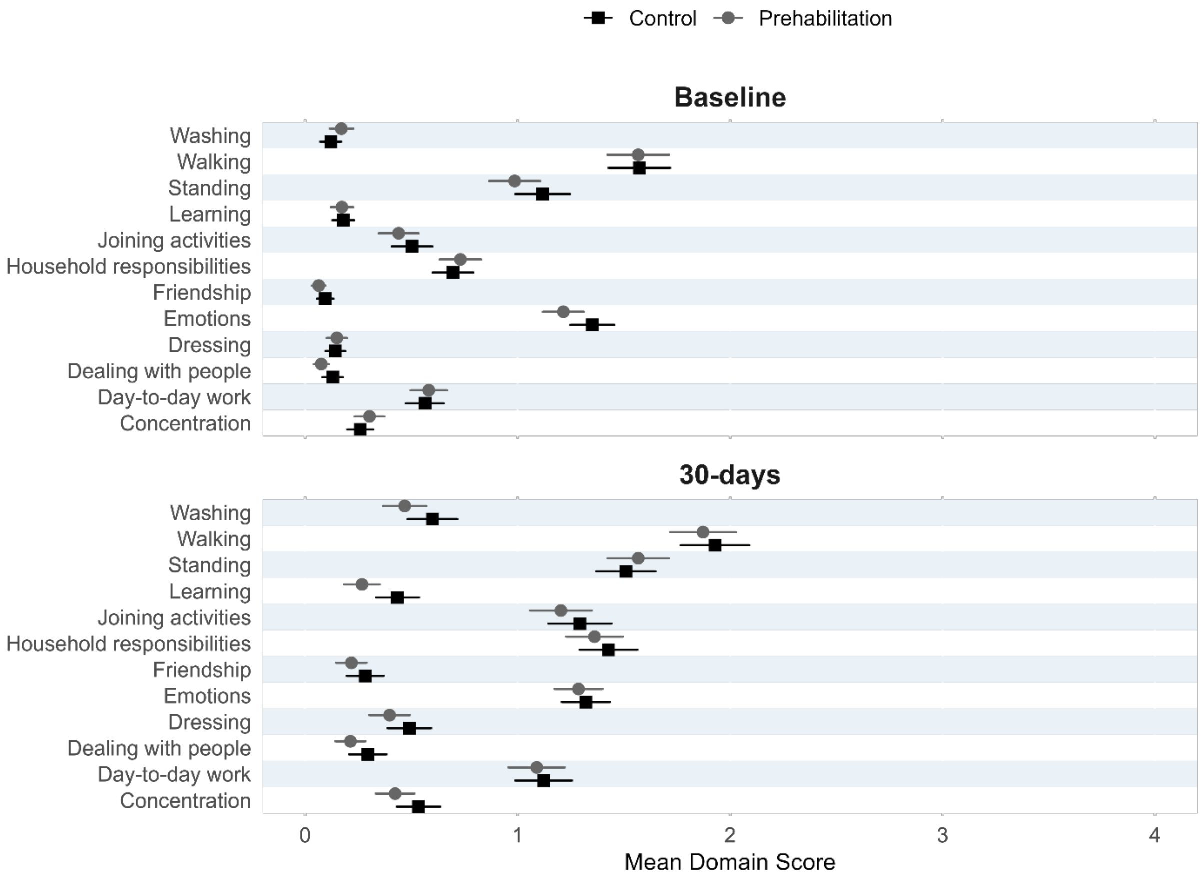

© 2025 McIsaac DI et al; PREPARE Trial Investigator Group. *JAMA Surgery*.

eTable 9. Results of Per-Protocol Analyses at 30-Day Follow-Up

**Per Protocol Primary Outcome and Prespecified Secondary Outcomes at 30-day Follow Up**				
**Outcome**	**Prehabilitation n=171**	**Usual care n=424**	**Intervention effect (CI)**	**P-value**
Primary outcome				
**WHODAS Disability Score - mean ± SD**	**19.7 ± 19.3**	**24.7 + 23.8**	**-4.9 (-9.8 to -0.01)***	**0.0247**
Secondary outcomes				
**EuroQol visual analogue scale score - mean + SD (0 to 100 scale)**	**68.4 (21.8)**	**63.1 (24.4)**	**5.5 (1.3 to 9.8)****	**0.011**
**EuroQol utility index - mean + SD (-1 to 1.59 scale)**	**0.8 (0.2)**	**0.8 (0.3)**	**0.06 (0.007 to 0.1)****	**0.0254**
Deaths - no. (%)	1/171 (1.4)	7/424 (1.7)	0.43 (0.05 to 3.78)†	0.445
Falls - no. (%)	14/169 (7.7)	31/410 (7.6)	0.91 (0.44 to 1.86)‡	0.795

*represents a mean difference with 97.5% confidence interval.

**represents a mean difference with 95% confidence interval.

† represents a hazard ratio with 95% confidence interval.

‡ represents an odds ratio from mixed-effects binary logistic regression with 95% confidence interval.
Bolding highlights statistically significant results

© 2025 McIsaac DI et al; PREPARE Trial Investigator Group. *JAMA Surgery*.

eTable 10. Results of Per-Protocol Analyses for Hospitalization Outcomes

Primary Outcome and Prespecified Secondary Outcomes at Hospital Discharge				
**Outcome**	**Prehabilitation n=149**	**Usual care n=352**	**Intervention effect (CI)**	**P-value**
Primary outcome				
Any postoperative complication - no. %	74/149 (49.7)	168/353 (47.7)	1.06 (0.67 to 1.67)*	0.785
Secondary outcomes				
Five times sit to stand time -mean + SD in seconds	45.5 (18.6)	42.4 (19.8)	1.9 (-1.7 to 5.4)**	0.305
Katz Index of Activities of Daily living-median (IQR) (0 to 6)	6 (5 to 6)	6 (5 to 6)	0.84 (0.58 to 1.23)***	0.372
Discharge disposition - median (IQR) (range died in hospital to home without support)	4 (4 to 4)	4 (4 to 4)	1.11 (0.97 to 1.81)‡	0.693
Time to discharge - mean + SD in days	5.5 + 9.4	5.6 + 8.5	1.06 (0.89 to 1.25)†	0.529
Postoperative complication severity - median (IQR) (range 0 to 5)	0 (0 to 2)	0 (0 to 2)	0.97 (0.67 to 1.41)***	0.874
Readmission - no. (%)	15/149 (10.1)	40/347 (11.7)	0.94 (0.49 to 1.80)¶	0.855

*represents an odds ratio from mixed-effects binary logistic regression with 97.5% confidence interval.

**represents a mean difference with 95% confidence interval.

***represents a generalized odds ratio from mixed-effects ordinal logistic regression with 95% confidence interval where values <1 denote better outcome with intervention.

† represents a subdistributional hazard ratio with 95% confidence interval, accounting for death as a competing risk where values >1 denote shorter time to discharge with intervention.

‡ represents a generalized odds ratio from mixed-effects ordinal logistic regression with 95% confidence interval where values >1 denote better outcome with intervention.

¶ represents an odds ratio from mixed-effects binary logistic regression with 95% confidence interval.

© 2025 McIsaac DI et al; PREPARE Trial Investigator Group. *JAMA Surgery*.

© 2025 McIsaac DI et al; PREPARE Trial Investigator Group. *JAMA Surgery*.
