# Supplementary material for: Home-Based Prehabilitation for Older Surgical Patients With Frailty: A Randomized Clinical Trial
Source: JAMA Surg. 2025 Dec 3;161(2):113–23. doi: 10.1001/jamasurg.2025.5288 (PMC12676472; doi:10.1001/jamasurg.2025.5288)
Supplement: Supplement 4. — Data Sharing Statement [file jamasurg-e255288-s004.pdf]

Data Sharing Statement

McIsaac. Home-Based Prehabilitation for Older Surgical Patients With Frailty. *JAMA Surg*. Published December 03, 2025. doi:10.1001/jamasurg.2025.5288

Data
Additional Information: NCT04221295
Data available: Yes
Data types: Other (please specify)
Additional Information: Trial data will be available for data sharing through reasonable request to the corresponding author. This includes a data dictionary and de-identified participant level data.
How to access data: Data access requests can be made to Daniel I McIsaac (dmcisaac@toh.ca_
When available: With publication

Supporting Documents
Document types: None

Additional Information
Who can access the data: Reasonable requests for data sharing will be reviewed by the trial executive committee and sharing will be conducted with researchers whose proposed use of the data has been approved.
Types of analyses: Data sharing will be available for specified purposes.
Mechanisms of data availability: Data sharing will be available after approval of a proposal and with signed data access agreements.
